# CLL Cells Respond to B-Cell Receptor Stimulation with a MicroRNA/mRNA Signature Associated with MYC Activation and Cell Cycle Progression

**DOI:** 10.1371/journal.pone.0060275

**Published:** 2013-04-01

**Authors:** Valerie Pede, Ans Rombout, Jolien Vermeire, Evelien Naessens, Pieter Mestdagh, Nore Robberecht, Hanne Vanderstraeten, Nadine Van Roy, Jo Vandesompele, Frank Speleman, Jan Philippé, Bruno Verhasselt

**Affiliations:** 1 Department of Clinical Chemistry, Microbiology and Immunology; Faculty of Medicine and Health Sciences, Ghent University, Ghent, Belgium; 2 Department of Medical Genetics, Faculty of Medicine and Health Sciences, Ghent University, Ghent, Belgium; University of Thessaly, Greece

## Abstract

Chronic lymphocytic leukemia (CLL) is a disease with variable clinical outcome. Several prognostic factors such as the immunoglobulin heavy chain variable genes (*IGHV*) mutation status are linked to the B-cell receptor (BCR) complex, supporting a role for triggering the BCR *in vivo* in the pathogenesis. The miRNA profile upon stimulation and correlation with *IGHV* mutation status is however unknown. To evaluate the transcriptional response of peripheral blood CLL cells upon BCR stimulation *in vitro*, miRNA and mRNA expression was measured using hybridization arrays and qPCR. We found both *IGHV* mutated and unmutated CLL cells to respond with increased expression of MYC and other genes associated with BCR activation, and a phenotype of cell cycle progression. Genome-wide expression studies showed hsa-miR-132-3p/hsa-miR-212 miRNA cluster induction associated with a set of downregulated genes, enriched for genes modulated by BCR activation and amplified by Myc. We conclude that BCR triggering of CLL cells induces a transcriptional response of genes associated with BCR activation, enhanced cell cycle entry and progression and suggest that part of the transcriptional profiles linked to *IGHV* mutation status observed in isolated peripheral blood are not cell intrinsic but rather secondary to *in vivo* BCR stimulation.

## Introduction

Chronic lymphocytic leukemia (CLL) patients show a highly variable clinical course: some patients have an almost normal life expectancy without need for treatment, while other patients die of drug-resistant disease within 2 years after initial diagnosis [1]. Currently, clinical consensus recommends not to rely exclusively on clinical staging systems such as the Rai or Binet score for prognostic assessment of CLL patients, but to take into account other prognostic parameters to predict clinical outcome, even in low stage disease [2]. Besides genetic markers, other markers were demonstrated to be of prognostic value such as mutation status of the variable region of the immunoglobulin heavy chain gene (*IGHV*) and the expression of CD38, lipoprotein lipase (LPL) and zeta-chain associated protein kinase 70 kDa, ZAP-70 [3] (reviewed in [2]). Remarkably, all of them relate to the B-cell receptor (BCR) directly or indirectly. Additionally, the similarity in BCR structure and reactivity between some CLL cases suggest that CLL B cells may typically recognize specific antigens [4], [5]. The BCR plays an important role in the interaction of B cells with the micro-environment in germinal centers, needed for proliferation and survival. In CLL, *in vivo* triggering of the BCR is believed to contribute to pathogenesis and clinical evolution of the disease [6]. Indeed, antigen recognition by the BCR would result in activation of transcription factors, such as nuclear factor-kappaB (NFκB) complex, nuclear factor of activated T cells (NFAT) complex and FOS [7]. Cross-linking the surface IgM receptor with the use of anti-IgM antibodies *in vitro* results in a heterogeneous response among CLL cases, as assessed by tyrosine phosphorylation, Ca^2+^ mobilization or even by measuring survival after Ig cross-linking [8]. The heterogeneous response was found to correlate with several prognostic indicators of progressive disease, including CD38, ZAP-70 and *IGHV* mutation status [8]–[11]. However, whether this reflects an intrinsic defect of the BCR signaling pathway remains unresolved. Controversial data have been reported on the transcriptional response of CLL upon BCR stimulation [6], [12]. Moreover, micro-RNA expression signatures correlating with prognostic subgroups have been published [13]–[15]. How microRNA expression is affected by BCR triggering and how it relates to mRNA signatures is at present unknown.

We report here that both *IGHV* mutated and unmutated CLL cells respond to BCR ligation *in vitro* with prominent MYC expression and changes in the miRNA profile, typically showing an induction of the hsa-miR-132-3p/hsa-miR-212 miRNA cluster. Transcriptome analysis further shows induction of FOS, NFAT5, DUSP2, EGR1 and ELK1. All these are part of a larger induced profile of genes associated with cell cycle initiation and progression, further confirmed phenotypically. This transcriptional response upon BCR triggering cluster probably supports a MYC amplified proliferative response that allows CLL cells to multiply in suitable niches *in vivo*.

## Materials and Methods

### Ethics statement

The study protocol was approved by the Ghent University Hospital Ethical Committee. Patient samples were obtained after informed consent.

### Patients sample collection and characterization

Twenty-one patients newly diagnosed with CLL in Ghent University Hospital were included in the present study after informed consent. Peripheral blood mononuclear cells (PBMC) were isolated on a Lymphoprep (Nycomed, Oslo, Norway) layer, and contained as expected for the majority CD19^+^CD5^+^ CLL cells (Table S1, median 94%, range 36–99). Cryopreservation did not compromise functional experiments after thawing. Characteristics of the patients included are summarized in Table S2. Determination of *IGHV* mutation status and intracellular ZAP-70 expression was performed as previously described [16]. Protein membrane expression was analyzed by flow cytometry after labeling with anti-CD19 (PE or allophycocyanin, APC), anti-CD3-fluorescein isothiocyanate, (FITC); both from BD Biosciences, San José, California, USA) and anti-CXCR4-PE (BD Pharmingen, San Diego, California, USA). Data acquisition and analysis were performed using BD FACSDiva software.

### Cytogenetic analysis

Detection of copy number aberrations was done either by fluorescence in situ hybridization (FISH) (cases CLL-1,-4 and 13–21) or by array comparative genomic hybridization (array-CGH) (all other cases). FISH was performed as previously described [17] using the following probes: LSI 13 (RB1)+LSI D13S319 (13q14.3) (detection of 13q deletion) and LSI TP53 (17p13) (detection of 17p deletion), both from Abbott Laboratories, Wavre, Belgium, and BAC clone RP11-241D13 (detection of 11q deletion), BAC PAC resource center, CHORI, Oakland, CA, USA.

Array-CGH was performed using a 60K SurePrint G3 unselected oligonucleotide array (Agilent Technologies, Amstelveen, The Netherlands). For the hybridization of the arrays 200 ng of tumor DNA and reference DNA were labelled with Cy3 and Cy5, respectively (BioPrime ArrayCGH Genomic Labeling System, Invitrogen, Merelbeke, Belgium). Further processing was done according to the manufacturers' instructions. Features were extracted using the feature extraction v10.1.0.0.0 software program and processed with an in-house developed visualization software arrayCGHbase (http://medgen.ugent.be/arrayCGHbase) [18], including circular binary segmentation for scoring of DNA copy number alterations [19].

### Cell culture and BCR stimulation

Cells were cultured as described before [20]. BCR stimulation was performed as described by Kofler et al. [21] Anti-IgM-polyacrylamid immunobead (anti-IgM) reagent (Irvine Scientific, Santa Ana, CA, USA) was added to the PBMC cultures at a concentration of 100 µg/mL for 3 or 24 hours. Anti-IgA-immunobeads (anti-IgA, Irvine Scientific) served as a negative control. In the CD19+ cells (before purification) the annexin negative fraction remained stable over a period of 24 hours: average 63% at 3 hours and 66% at 24 hours after initiation of BCR stimulation.

CLL cell purification was performed after stimulation by negative depletion using EasySep technology (Stem Cell Technologies, Vancouver, Canada). The percentage of viable CD19^+^ cells was assessed by flow cytometry and was at least 98.2% (data not shown).

To measure cell cycle progression, stimulated CLL cells were labelled with CD-19-PE, CD3-FITC and DRAQ5 (Biostatus Limited, Leicestershire, U.K.) after 48 hours of stimulation and analyzed by flow cytometry.

### Real-time quantitative PCR (qPCR)

Total cellular RNA was extracted using the miRNeasy kit (Qiagen, Hilden, Germany), cDNA was synthesized and LPL measured with qPCR as published before [22]. All other genes were measured using a calibration curve of 8, four-fold dilutions of cDNA made from stimulated CLL cells. These assays were either SybrGreen based with primers described before for MYC [23] and ACTB [24], or probe hydrolysis based using either published primers and probes (for ABL1 [25]), or Assay-on-Demand® gene expression assays (Applied Biosystems, for ELK1, NFAT5, FOS, DUSP2, EGR1, EP400, ZBTB5, CXCR4), used according to the manufacturer's instructions. All reactions were performed in duplicate on a LightCycler 480 (Roche, Basel, Switzerland) or on ABI Prism 7300 Real Time PCR System (Applied Biosystems, Foster City, CA, USA). ABL1 and ACTB reference genes were sufficient to normalize gene expression as assessed with geNorm software [26]. Expression of selected miRNAs was confirmed on the same RNA used in the genome-wide miRNA expression analysis and on an additional patient samples, all without cDNA amplification before quantification. For normalization, the three most stable small RNA controls (RNU 48, RNU 24, RNU 44) were used [27]. All qPCR reactions for microRNAs were performed in duplicate on a LightCycler 480 or on ABI Prism 7900 HT cycler.

### Genome-wide expression analysis

750 ng of total RNA from freshly isolated CLL cells was used for Illumina microarray analysis in an external facility (ServiceXS, Leiden, The Netherlands). Quality and integrity of the RNA samples was analyzed with the Agilent Bioanalyzer (Agilent Technologies). The Illumina TotalPrep RNA Amplification Kit (Ambion, Austin, TX, USA) was used to synthesize biotine labeled cRNA. Biotinylated cRNA (750 ng) was hybridized onto the HumanHT-12 v3 Expression BeadChip. Illumina's GenomeStudio v1 software with the default settings was used for Gene Expression analysis.

The Illumina mRNA expression data were normalized using quantile normalization from the ‘affy’ package using Rgui statistical language (www.bioconductor.org). Bead summary data were log2-transformed and normalized by quantile normalization using the bead array package [28]. mRNA differential expression analysis was performed by Rank Product analysis using the RankProd package [29] in Bioconductor. Pfp (percentage of false positive predictions) values were calculated from 100 permutations and a cut-off value of 5% (0.05) pfp was applied to define differentially expressed genes. Fold change in expression was calculated as the average of the expression ratios of IgM stimulation/IgA stimulation.

Normalized gene expression values were analysed using Gene Set Enrichment Analysis (according to Subramanian et al. [30]) with phenotype labels representing the different sample subgroups (i.e. stimulated and unstimulated). The following gene set collections (version 3.1) were analyzed: Gene Ontology Biological Process, KEGG and Transcription Factor Targets. Significant gene set associations were selected based on the FDR q-value (FDR q<0.05), obtained by 1,000 permutations of phenotype labels.

Genome wide miRNA expression was measured as described previously [31]. Briefly, miRNA specific cDNA synthesis for 636 different human miRNAs was followed by pre-amplification by means of a 14-cycle PCR reaction with a pool of miRNA specific forward primers and universal reverse primers to increase detection sensitivity. Finally, pre-amplified miRNA cDNA was used as input for arrayed qPCR reaction with miRNA specific hydrolysis probes and forward primer and universal reverse primer (Applied Biosystems). All reactions were performed on the 7900 HT cycler (Applied Biosystems). Prior to miRNA expression normalization, Cq-values>35 were excluded from the analysis. miRNA expression data were normalized using the global mean [27]. Only miRNAs that were detected in at least 80% of the samples within one of the defined subgroups were included in the differential expression analysis. Differentially expressed miRNAs were identified using the Rank Products algorithm [29] as described above. Fold change in expression of a miRNA was calculated as the average of the expression ratios of anti-IgM stimulation/anti-IgA stimulation.

### Correlation mRNA/miRNA

To identify putative functions associated with the miRNAs of interest, we performed an integrative mRNA – miRNA expression analysis according to Mestdagh et al. [32]. Briefly, matching mRNA and miRNA expression levels were correlated using Spearman's Rank statistics. For each miRNA, mRNAs were ranked according to their correlation coefficient and miRNA associated functions were identified using Gene Set Enrichment Analysis [33] on the ranked mRNA list. Inferred functions were uploaded to the miRNA body map webtool (www.miRNAbodymap.org).

Ingenuity Pathways Analysis software version 9.0 application 2011-07-23, content 2011-05-18 (Ingenuity Systems,Redwood City, CA) was used to identify and visualize modulated pathways (http://www.ingenuity.com/products/pathways_analysis.html).

### Statistics

All statistical analyses on genome-wide expression data were performed using the R statistical programming language (version 2.11).

For comparing stimulated versus unstimulated samples, the Wilcoxon matched pairs test and for comparing unmutated versus mutated samples the Mann-Whitney U test was applied using the GraphPad Prism 5 statistical software (GraphPad Software, La Jolla, CA, USA).

## Results

### Both *IGHV* mutated and unmutated CLL cells transcriptionally respond to B-cell receptor stimulation

**Figure 1:**
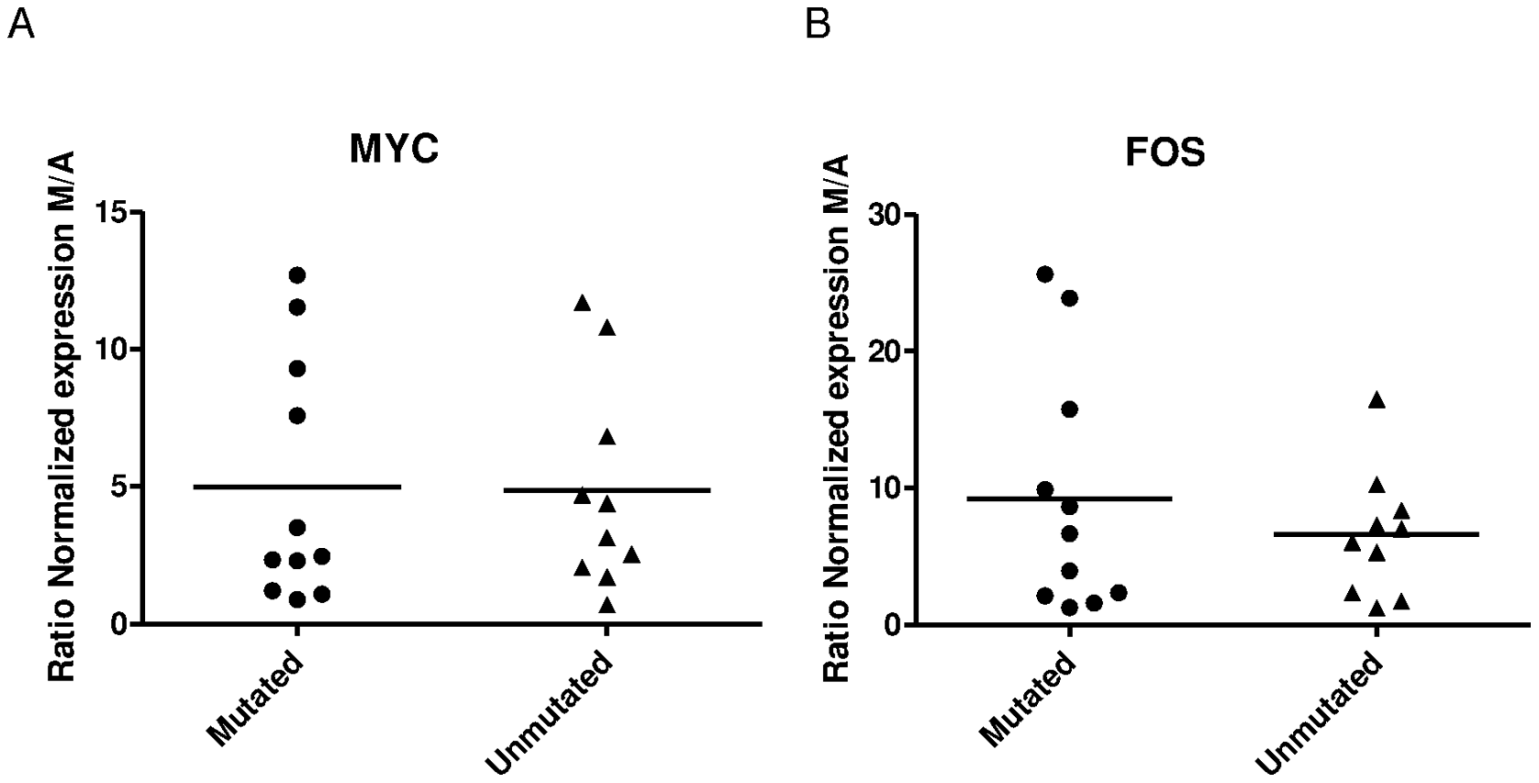
BCR stimulation of both *IGHV* mutated and *IGHV* unmutated CLL cells induces gene expression. Expression of MYC (A) and FOS (B) in CLL cells stimulated with anti-IgA or anti-IgM beads for 24 hours (MYC) or 3 hours (FOS). Scatter plots show normalized mRNA expression for *IGHV* mutated (M, • ; N = 11) and *IGHV* unmutated cases (U, ▴; N = 10), horizontal line represent average value. Significant induction of both MYC and FOS (p<0.05), however not significantly different between *IGHV* mutated and *IGHV* unmutated cases. (C) Kinetics of expression of ELK1, NFAT5, FOS, DUSP2, EGR1 and MYC. Scatter plots show normalized mRNA expression for *IGHV* mutated (M, • ; N = 4) and *IGHV* unmutated cases (U, ▴; N = 4), horizontal lines represent average values. Significant differences are indicated (*) (p<0.05).

Previous studies showed that expression profiles of CLL cells freshly isolated from peripheral blood show considerable overlap between unmutated and mutated samples [34], [35]. While Herishanu et al. [6] and recently Krysov et al. [36] show that both *IGHV* mutated and *IGHV* unmutated CLL cells transcriptionally respond to BCR ligation *in vitro*, other studies reported that *IGHV* mutated CLL cells poorly respond to IgM stimulation, in contrast to *IGHV* unmutated CLL cells which do respond [12], [37]–[40]. We stimulated CLL cells with anti-IgM beads or control anti-IgA beads. After 24 hours of stimulation, both *IGHV* mutated and *IGHV* unmutated CLL cells induced *MYC* expression to the same level (Fig. 1A). Similarly, no significant difference was seen in induction of *FOS* expression upon 3 hours of BCR stimulation (Fig. 1B). Collectively, these results show a clear response of CLL cells to BCR triggering, but no significant difference in stimulation efficiency between mutated (N = 11) and unmutated (N = 10) cases, measured by *FOS* or *MYC* expression. By contrast, expression of *LPL* increased on average to levels six times higher in unmutated compared to mutated cases upon BCR ligation (data not shown). This illustrates that our samples are inherently different according to mutational status, since previous reports [6], [41] showed LPL to increase specifically in unmutated CLL cells upon BCR stimulation.

A peak in MYC expression was reached already after 3 hours (median about 13-fold induction), but expression remained high up to 24 hours of stimulation (Fig. 1C). To further analyze the response we performed kinetic measurement of mRNA expression of genes downstream of the BCR (transcription factors ELK1, EGR1, FOS and NFAT5, and of DUSP2, a negative regulator of ERK) after stimulation of CLL cells. EGR1, FOS and to a lesser extent ELK1 were induced soon after stimulation, but returned to control levels within 6 to 24 hours, again without statistical significant differences between *IGHV* mutated and *IGHV* unmutated CLL samples. Interestingly, expression of DUSP2, a negative regulator of ERK that drives expression of these transcription factors, was induced simultaneously in these samples, suggesting a negative feedback. In addition, expression of NFAT5 was induced in both *IGHV* mutated and *IGHV* unmutated CLL samples reaching a peak after 3 hours (Fig. 1C), indicating that also the p38 MAPK pathway was activated in our samples [42]. We did observe that irrespective of mutational status, the magnitude of induction of these genes within one donor correlated (data not shown).

Since especially MYC is an amplifier associated with cell cycle entry in B cells [43], we determined if the stimulated cells did show phenotypic signs of proliferation. As shown in Fig. S1, DNA staining revealed that a small fraction of the cells was in S/G2 phase, in IgM stimulated but hardly any in control IgA stimulated cells. This response was seen both in *IGHV* mutated and unmutated CLL cells, in 6 of the 8 samples tested.

### B-cell receptor triggering of CLL cells results in a transcriptional response enriched for genes involved in proliferation

Genome-wide transcriptome analysis was performed on samples stimulated for 3 and 24 hours, two time points that are fit to discriminate the kinetic profiles we observed with the selected genes observed above in an independent series of samples (overview of samples in Table S1). Rank-product analysis detected 984 and 1192 differentially expressed genes with an increase in expression (percentage false positive <0.05), after 3 and 24 hours of stimulation respectively, and 1095 (3 hours) and 1190 (24 hours) genes with decreased expression (percentage false positive <0.05). Of these, 239 (3 hours) and 164 (24 hours) of the upregulated genes and 140 (3 hours) and 102 (24 hours) of the downregulated genes showed a fold change of at least 2 (Table S3). The most significantly modulated genes with a fold change of 3 or more are listed in Table 1. As expected, DUSP2, FOS, EGR1 and MYC were part of the most prominently upregulated transcripts 3 hours after BCR stimulation, while of these only MYC was more than 3 fold upregulated 24 hours after stimulation. On the other hand, ELK1 and NFAT5 were found to be only modestly upregulated by BCR triggering, scoring below the 2 fold induction threshold (1.6 and 1.5 respectively, Table S3). The array data are therefore remarkably in line with the qPCR results in part obtained in an independent series of samples.

**Table 1:** Change in mRNAs expression after 3 hours or 24 hours of BCR stimulation.

3 hours of stimulation		24 hours of stimulation	
Upregulated			
Gene	FC	Gene	FC
CCL3L1	6.49	CCL4L2	8.74
CCL4L2	6.04	CCL4L1	7.69
DUSP2	6.02	DDIT4	5.59
FOS	5.42	TRIB3	5.18
CKS2	5.34	RGS1	5.08
CCL3	5.27	SLC7A5	4.57
UBTD1	5.21	CCL3	4.14
CCL4L1	5.07	GZMB	4.07
MYC	4.82	MTHFD2	4.02
NR4A3	4.78	CCL3L1	3.84
C13ORF15	4.64	CCL3L3	3.80
FOSB	4.61	MGC4677	3.69
PHLDB1	4.60	MYC	3.42
EGR1	4.58	C20ORF100	3.37
MGC4677	4.49	IGSF3	3.33
NR4A2	4.46	PSAT1	3.27
CCL3L3	4.32	FAM152B	3.26
RCAN1	4.20	CD1C	3.20
SERPINE2	4.02	DBN1	3.20
CHRNA1	3.95	RCAN1	3.19
HOMER1	3.93	MTHFD1L	3.19
TRIB3	3.93	OAS3	3.15
EGR2	3.92	ADM	3.11
MYCN	3.90	LRRC32	3.08
TRK1	3.87	BATF3	3.07
EGR3	3.83	SLC1A5	3.04
LOC143666	3.81		
HS.562534	3.71		
DDIT4	3.59		
RNF19A	3.54		
SERTAD1	3.51		
PTGER4	3.51		
LOC653506	3.49		
PIM3	3.49		
TRQ1	3.45		
CHRNA1	3.42		
HS.538259	3.41		
GRAMD4	3.39		
HS.543887	3.38		
RNF19A	3.36		
ATF3	3.33		
KLF10	3.26		
RHOB	3.25		
BTG3	3.23		
C17ORF91	3.22		
MAPK6	3.22		
C10ORF54	3.18		
PDCD1	3.17		
CD200	3.13		
EIF2AK3	3.07		

Downregulated			
Gene	FC	Gene	FC
TXNIP	0.20	LTB	0.18
CD79B	0.20	VPREB3	0.22
CXCR4	0.22	CD24	0.23
CYBASC3	0.29	TXNIP	0.28
SEMA4B	0.29	CECR1	0.29
PRICKLE1	0.31	TMEM71	0.30
BCL11A	0.32	ALOX5	0.31
NUAK2	0.33	SNORD13	0.31
FLOT2	0.33	GNG7	0.31
CTDSP2	0.33	CXCR4	0.32
		C1ORF162	0.33
		TGFBI	0.33
		SPOCK2	0.33

Table shows three-fold up- or downregulated genes after 3 or 24 hours of BCR stimulation. Fold change (FC) is indicated, all entries percentage of false positives <0.0001.

To validate some of these observations in independent samples, we performed additional stimulation experiments. The increased expression at the mRNA level translated into secretion of CCL3 and CCL4 by BCR stimulated CLL cells as measured with ELISA (Fig. S2). Reduction of CXCR4 (confirmed by qPCR on these and independent samples, as shown in Fig. S3) and CD19 mRNA expression was accompanied by a reduction of cell surface expression, as shown in Fig. S4.

**Figure 2:**
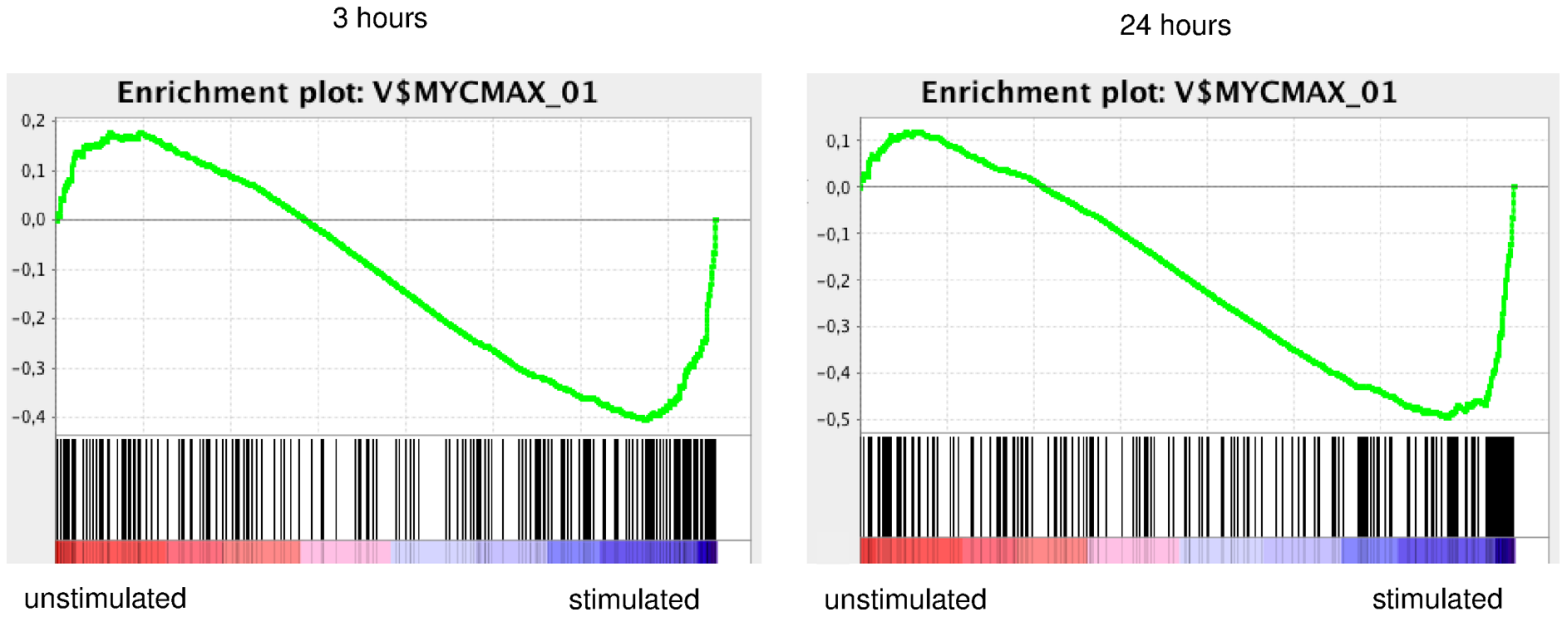
BCR stimulation induces an expression profile enriched for MYC induced genes. Figure shows Gene Set Enrichment Analysis enrichment plot of MYCMAX_01 gene set from Transcription Factor Targets collection (version 3.1) of data obtained after 3 hours or 24 hours of stimulation as indicated. Bottom shows location of the genes in MYCMAX_01 set in the ranked list of differentially expressed genes: highest in unstimulated samples left (red zone) to highest in stimulated samples right (blue zone)). Upper part shows profile the running enrichment score (green line), showing maximum enrichment score (negative value) in stimulated samples. For both time points, FDR q value was below 0.01.

To better understand the biological significance of our data we performed Gene Set Enrichment Analysis using different gene set collections [30]. We compared stimulated samples to unstimulated samples, and found in both Gene Ontology Biological Process and KEGG gene set collections an enrichment for gene sets involved in cell cycle and metabolic processes. In the Transcription Factor Targets collection, most enriched sets were genes associated with MYC activation. (Table S4). As shown in Fig. 2, the profile of the running enrichment score for the MYC gene set shows a peak in the ranking region of those genes in expression most correlated to stimulated samples. This was highly significant both after 3 hours and 24 hours of stimulation.

### B-cell receptor stimulation affects miRNA profiles in B-CLL cells

**Figure 3:**
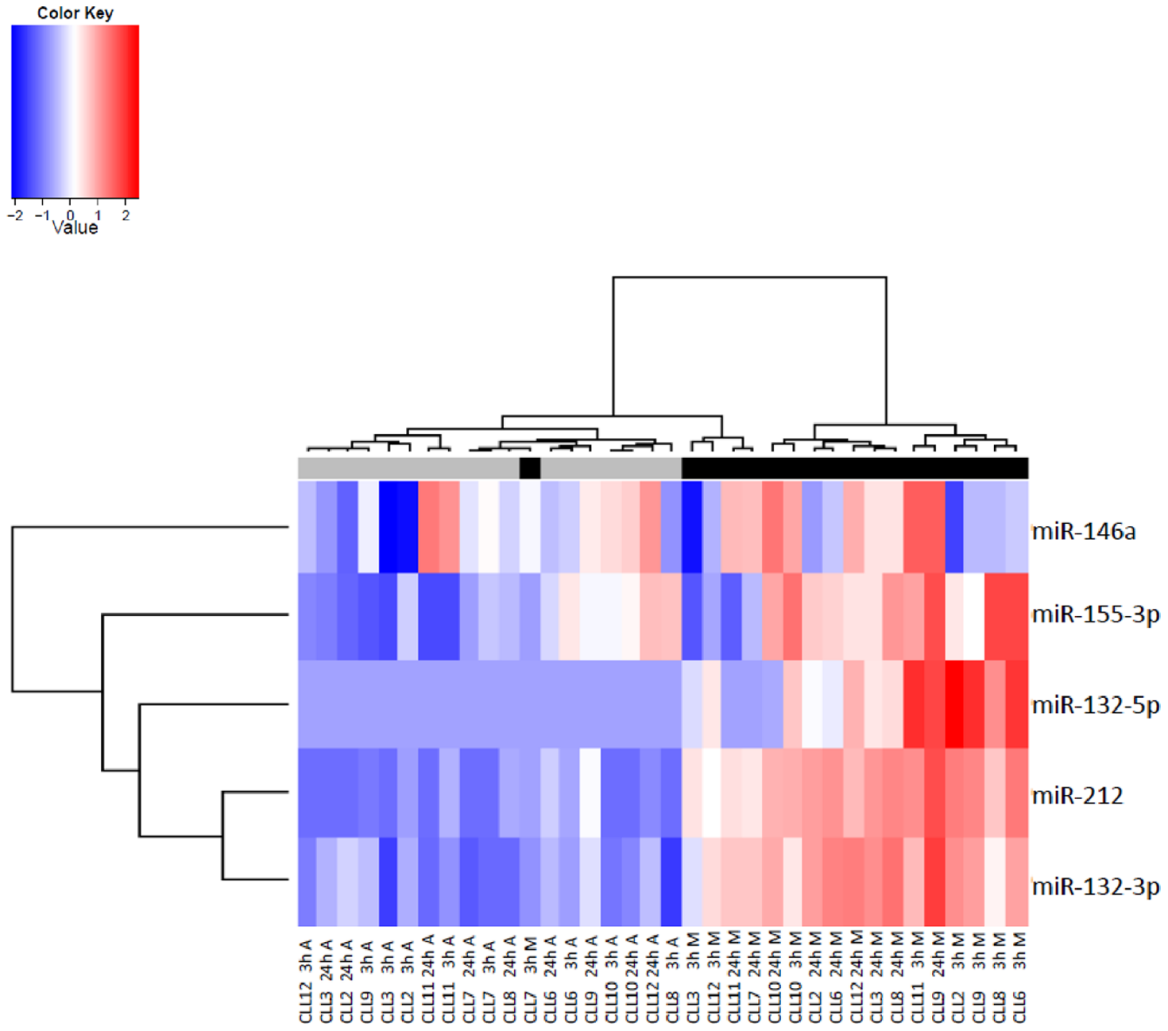
A selected set of miR characterizes BCR stimulated CLL cells. Heat-map shows unsupervised clustering of samples (anti-IgM stimulated black tag, control IgA stimulated grey tag) according to expression of hsa-miR-146a, hsa-miR-155-3p, hsa-miR-132-5p, hsa-miR212 and hsa-miR-132-3p. Code from blue (−2 log2 normalized expression) to red (+2 log2 normalized expression) indicates miR expression levels.

**Figure 4:**
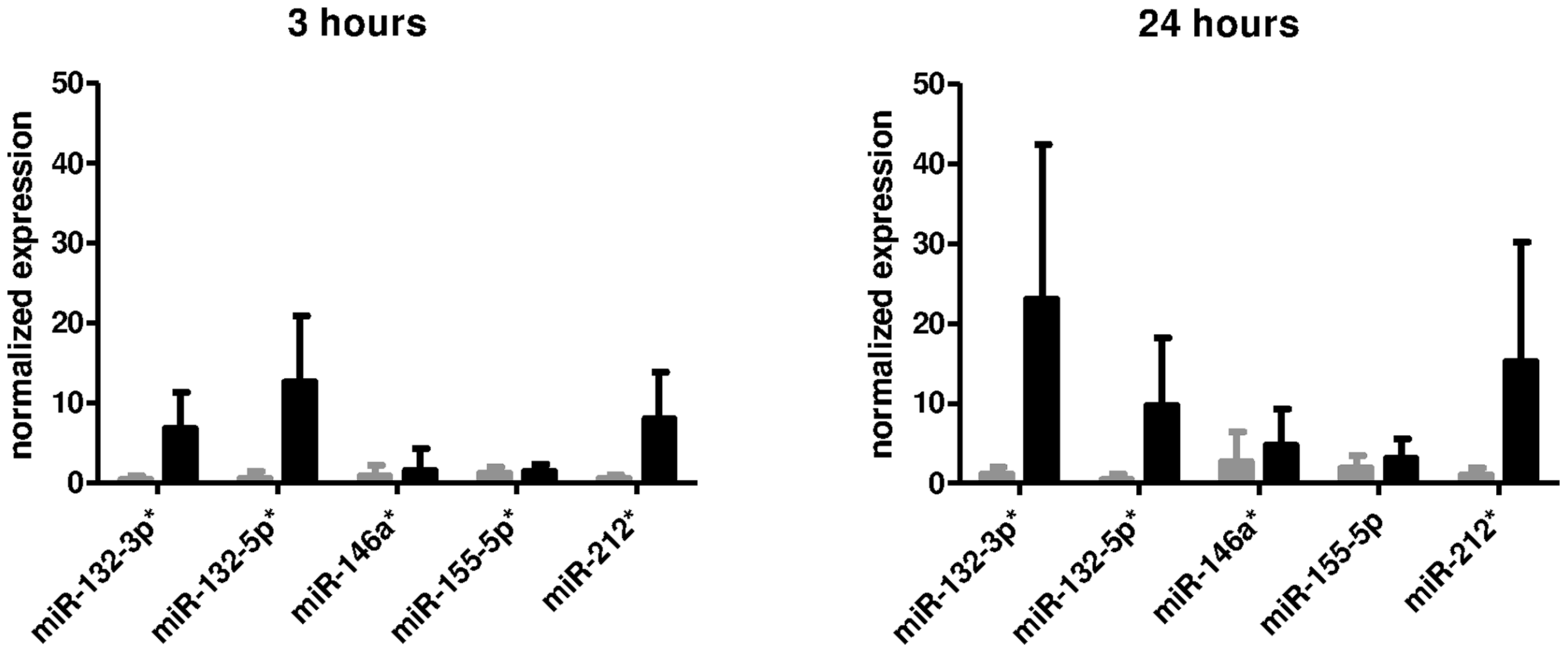
miRNAs induced by BCR stimulation of CLL cells. Normalized expression of selected miRNAs in CLL cells stimulated with anti-IgA (grey columns) or anti-IgM (black columns) beads for 3 or 24 hours (average ± SD N = 13). Induction by anti-IgM is significant for all miRNAs at both time points (* p<0.05), except for hsa-miR-155-5p after 24 hours.

**Figure 5:**
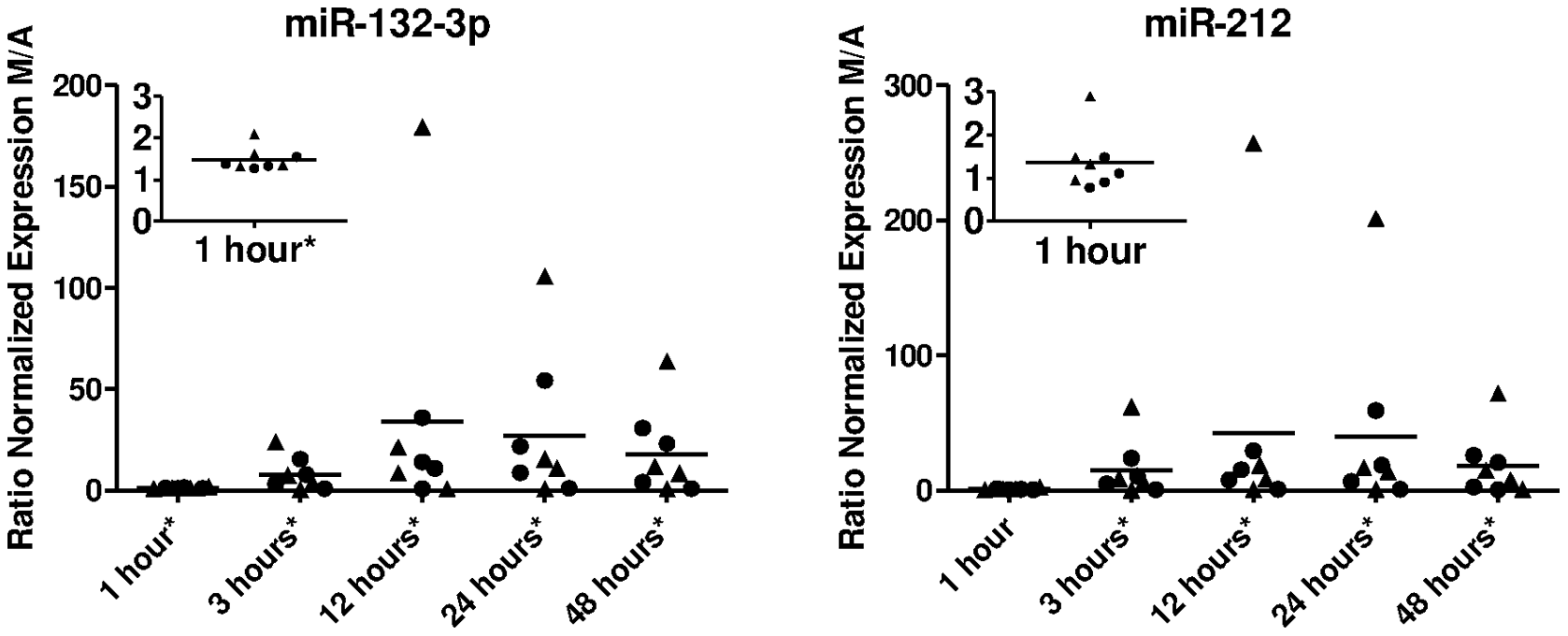
hsa-miR-132-3p and hsa-miR-212 expression induced by BCR stimulation of CLL cells peaks after 12 hours. Ratio of normalized expression (IgM stimulated/IgA stimulated) of hsa-miR-132-3p and hsa-miR-212 in CLL cells stimulated for the time as indicated (inset shows data for 1 hour in enlarged scale). *IGHV* mutated (M, •; N = 4) and *IGHV* unmutated cases (U, ▴; N = 4), horizontal lines represent average values. Significant induction is indicated (* p<0.05).

In our genome-wide transcriptome analysis after BCR stimulation, we observed clear kinetic modulation of many genes, suggesting a regulated expression. Given the importance of miRNAs on gene expression regulation and prior reports on prognostic relevance of miRNA expression in freshly isolated CLL cells [13]–[15], we went on to measure miRNA profiles in pre-amplified cDNA with a qPCR array assay covering 636 mature miRNAs, not including hsa-miR-155-5p. We detected 186 miRNAs in BCR stimulated CLL cells, listed in Table S5. Several of these were reported before by other groups to be relatively highly expressed in freshly isolated CLL cells [14], such as hsa-miR-150, also in our samples by far the most abundant microRNA. To detect modulation of miRNA expression after BCR stimulation, Rank Products analysis was performed. Table 2 shows up and down-regulated miRNAs (percentage false positive <0.05). The complete list of detected miRNAs, fold changes (the average of the expression ratios of anti-IgM stimulation/anti-IgA stimulation) and percentages false positive are shown in Table S6. Unsupervised clustering analysis revealed that neither mutational status nor stimulation was associated with the global miRNA signature (Fig. S5A and B). However, when clustering was restricted to the miRNAs hsa-miR-132-3p, hsa-miR-132-5p, hsa-miR-212, hsa-miR-146a and hsa-miR-155-3p, stimulated samples grouped almost perfectly together, albeit not according to time of stimulation nor donor identity (Fig. 3). In addition, this clustering shows a tight correlation between miR-132 and miR-212 expression. We selected five miRNAs for confirmation with qPCR without preceding amplification of the cDNA: (hsa-miR-132-3p, hsa-miR-132-5p, hsa-miR-212 (all significantly upregulated upon stimulation), hsa-miR-146a (borderline upregulated after 24 hours) and hsa-miR-155-5p (not present in the whole genome screen but reported before to be relevant in CLL prognostic signatures [13], [14]). As measured by single miRNA specific real-time PCR shown in Fig. 4, hsa-miR-132-3p, hsa-miR-132-5p and hsa-miR-212 were strongly upregulated 3 and 24 h after stimulation, confirming the array data, while the increase of hsa-miR146a and hsa-miR-155-5p expression was significant after 3 h but hardly after 24 h of stimulation (p<0.05 and not significant, respectively). We did not observe a significant difference in *IGHV* mutated compared to unmutated cases. Kinetics of the miR-132-3p and miR-212 upregulation in an independent series of samples (Fig. 5) revealed that the peak of induced expression was reached after 12 hours, and that even after 48 hours, expression was still clearly induced. Here again, the expression values measured in *IGHV* mutated and unmutated CLL were similar.

**Table 2:** Induction of miRNA expression after 3 or 24 hours of BCR stimulation.

3 hours of stimulation			24 hours of stimulation		
miR	FC	Pfp	miR	FC	Pfp
**hsa-mir-212**	17.75	0	**hsa-mir-212**	20.97	0
**hsa-mir-132-3p**	7.21	0	**hsa-mir-132-3p**	12.47	0
**hsa-mir-155-3p**	2.91	0.021	**hsa-mir-155-3p**	2.75	0.012
**hsa-mir-20a-3p**	2.46	0.026			
**hsa-mir-132-5p**	3.12	0.029			
**hsa-mir-19b-1-5p**	2.91	0.035			

FC: fold change (the average of the expression ratios of IgM stimulation/IgA stimulation), Pfp: percentage of false positives is indicated.

### Integrated miRNA/mRNA induction upon B-cell receptor stimulation supports cell cycle entry and progression

Pathway analysis demonstrated that many of the differentially expressed genes after 3 hours of stimulation were involved in BCR signaling and PI3K signaling (Table S7). As shown in Fig. S6A, after 3 hours of stimulation, downstream BCR effectors were upregulated (EGR1, NFAT, ELK1), while expression of upstream components of the BCR transducing components (CD19, CD79A, CD79B) and immediate downstream signaling components (LYN, SYK, VAV1, PI3K components and even ERK1) are already downmodulated while ERK1-degrading DUSP2 was upregulated. This wave of modulated expression results after 24 hours of stimulation in upregulation of NFκB pathway components (Fig. S6B). As listed in Table S7, 24 hours after stimulation, genes upregulated were involved in purine metabolism (more than 50 enzymes involved are upregulated, e.g. HPRT1), pyrimidine metabolism (30 enzymes involved), glycolysis (e.g. all enzymes in the catabolic pathway from glucose-6-phosphate down to pyruvate, including GAPDH) and protein turnover/antigen presentation (e.g. ubiquitination pathway with many proteasome subunits).

The miRNAs hsa-miR-132-3p and hsa-miR-212 belong to the same cluster [44], and show considerable overlap in predicted target genes, according to several algorithms currently in use (TargetScan or mirDB). Hsa-miR-132-5p is the complementary strand of the miR-132-3p/miR-132-5p duplex, not definitely shown to be incorporated in the RNA-induced silencing complex [45]. As almost no targets of hsa-miR-132-3p/hsa-miR-212 are experimentally validated, we calculated the correlation between quantile normalized expression data of each gene with normalized expression of hsa-miR-132-3p/hsa-miR-212 in the miRNA qPCR screening in the same sample. Genes showing significant inverse correlation with the miRNA expression are shown in Table S8. Some of these are predicted targets of hsa-miR-132-3p/hsa-miR-212, such as TMEM50B, EP400 and ZBTB5. Other genes predicted to be targeted by hsa-miR-132-3p/hsa-miR-212 and significantly inversely correlated in our expression data are CFL2, ZCCHC11, LRRFIP1, MFSD11, RAD21, EIF4A2, HSBP1, EID2B and TGFB1. It should be noted that in the set of correlating genes, hsa-miR targeted genes will be enriched but correlation in se does not prove targeting. When hsa-miR-132-3p/hsa-miR-212 target genes predicted by Targetscan are compared to Kyoto Encyclopedia of Genes and Genomes database (www.genome.jp/kegg), a significant association with the KEGG BCR signaling pathway was found, containing upstream components like CD19, CD79A en CD79B. Possibly, early downregulation of these genes we observed is in part mediated by hsa-miR-132-3p and hsa-miR-212.

To evaluate the function of the genes correlating with induced miRNA after BCR stimulation, we used our in-house developed web-based algorithm (www.mirnabodymap.org) [32]. The genes we found to be significantly inversely correlating with an upregulated miRNA are compared to a database containing 3445 published experimental gene sets. For the hsa-miR-132-3p/hsa-miR-212 cluster, 26 sets correlated significantly with both miRNAs (Table S9), of which 12 were related with B cell progenitor/lymphoma or modulated upon MYC activation, further suggesting the relevance in MYC amplified cellular activation, proliferation and oncogenesis.

## Discussion

In this study, we show that CLL cells transcriptionally respond to BCR stimulation with increased expression of MYC, irrespective of the IGHV mutation status. Genome wide expression analysis revealed a mRNA/miRNA signature associated with BCR activation, cell cycle entry and progression.

Considerable overlap exists between the expression profile we observed and that observed upon BCR triggering by Vallat et al., who also demonstrate a functional group of genes associated with *MYC* expression [46]. We found matches between the transcriptional program of stimulated CLL cells with that of activated B cells (upregulated GLA, CTPS, GFI1, NAMPT, CD63, PDIA4/5/6, ADSL, GART, HPRT1, CCND2, AK2, NME2, GSS, RPA1, YWHAB/G, downregulated BANK1) [34]. Pathway analysis showed that in our experiments pathways modulated by BCR triggering were those known to be downstream of the BCR target (SYK/PLCγ/NFAT; ITK/ERK1-2/FOS; PI3K/NFκB). Consequently, many of the genes modulated soon after stimulation are involved in cell cycle initiation and progression (MYC, CCND1, CCND2, RBL2, E2F complex) and survival (e.g. BCL2, FOXO3). Gene Set Enrichment Analysis learned that stimulated samples were enriched for genes involved in metabolism and cell cycle. In the collection of transcription factor gene sets, MYC gene sets was the most strongly enriched gene set, underscoring the importance of this transcriptional activator in BCR stimulated CLL cells. Burger et al. [47] found a similar spectrum of upregulated genes, including CCL3/CCL4; EGR2/EGR3 and MYCN, in CLL cells stimulated by co-culture with nurse-like cells. After 24 hours of stimulation, many of the genes found by Burger et al. [47] were also upregulated in our experiments, together with nucleotide metabolism pathways, glycolysis and protein turnover, as is expected in cells starting a program of cell cycle initiation and proliferation. Interestingly, the profile we detect is similar to that reported by Guarini et al. [12] for unmutated CLL cells: more than half of the genes reported by them to be upregulated were also found by us. In addition, the strongest upregulated genes (more than 3 fold) described by Guarini et al. [12] were also upregulated, without exception, in our study. In agreement with them and several other studies [6], [12], [47], [48], we observed downregulation of CXCR4 (Table 1 and Fig. S3 and S4) correlating with reduced CD62L expression [38], maybe related to altered migration of stimulated CLL cells reported before [38], [47], [48]. Some authors observed a correlation between markers of progressive disease (mutational status, ZAP-70 expression) and the magnitude of CXCR4 downregulation after BCR stimulation [38], [39]. Moreover, Stamatopoulos et al. [49] observed reduction in CXCR4 surface expression after contact with mesenchymal stromal cells only in ZAP70^+^, not in ZAP70^−^ CLL cells. We did not observe a difference between mutated and unmutated CLL cells, possibly because in contrast to these authors we stimulated the mononuclear cell fraction instead of purified CLL cells, and in addition the anti-IgM beads we use present immobilized anti-IgM antibodies, which elicit superior BCR stimulation compared to soluble anti-IgM antibodies which are rapidly internalized by endocytosis [7], [39], [50]. The fact that the transcriptional response is similar in both *IGHV* mutated and unmutated CLL does not preclude that protein expression and activation after BCR ligation is different, as observed by several authors [50], [51]. Taken together, the mRNA expression profiles match that of BCR activated cells with a prominent signature of MYC induction, known to promote proliferation and gene expression in leukemic and other cancer cells [43], [52]. It would be interesting to explore if the similarities in transcriptome between freshly isolated CLL cells and normal donor CD5+ B cells still hold after BCR stimulation, to further underscore the origin of CLL in CD5^+^ B cell subsets [53].

A unique miRNA signature in freshly isolated CLL cells is associated with prognostic factors and disease progression in CLL [13]–[15]. Notably, expression of hsa-miR-132-3p was found to correlate with *IGHV* unmutated status, and included in the signature of poor prognosis CLL [13]–[15]. In our experiments, we found hsa-miR-132-3p to be induced equally well in *IGHV* mutated and *IGHV* unmutated CLL cells, nor was the induction different in samples with or without 13q14 deletion. The same was true for hsa-miR-212. Given our transcriptome data, we suggest that both *IGHV* mutated and *IGHV* unmutated CLL cells respond similarly on BCR triggering, and that the difference observed in freshly isolated peripheral blood CLL cells reflects a difference in *in vivo* triggering of the BCR. As reported by Herishanu et al. [6], the difference in mRNA profile is mainly restricted to cells harvested from the blood, and barely present in cells isolated from lymph nodes. This suggests that in the micro-environment of the lymph node, BCR triggering occurs for both *IGHV* mutated and *IGHV* unmutated CLL cells, from what we would predict that miRNA signatures in the lymph node will be different from those published using CLL cells freshly isolated from peripheral blood.

Besides hsa-miR-132-3p and hsa-miR-212, we detected a moderate increase in hsa-miR-146a and hsa-miR-155-5p early after BCR triggering. Interestingly, these miRNAs are upregulated by NFκB, a pathway we show to be activated after BCR triggering. In the B cell line Ramos, hsa-miR-155-5p was shown to be induced following BCR induced activation of a ERK/ELK-1/FOS pathway [54] which we show to be clearly activated in CLL cells following BCR triggering.

In our experiments, increased expression of hsa-miR-132-3p and hsa-miR-212 did correlate with decreased TGFB1, EP400 (a partner of MYC for transformation [55]), and ZBTB5 expression. The latter two proteins are known to decrease CDKN1A expression [56], [57] and TGFB1 is an inhibitor of BCR responsiveness [58], suggesting a role for hsa-miR-132-3p and hsa-miR-212 in the complex transcriptional program determining cell cycle initiation and progression. However, as RB1 activity is blocked by phosphorylation by the CCND1/CCND2/CDK4 complex, RB1 function will likely decrease to the benefit of E2F complex activity (reviewed by [59]). In addition, the RB1 homologue RBL2 was decreased in expression upon BCR triggering. A recent publication describes an overrepresentation of hsa-miR-132-3p and hsa-miR-212 in pancreatic cancer and shows that RB1 is a target of these miRNAs by a luciferase UTR assay [60]. A hypothetical model of possible miRNA/mRNA interactions and signaling cascades leading to enhanced BCR response and cell cycle progression is shown in Fig. S7. The induced miRNAs might modulate the expression of several proteins and the consequent effects of MYC induction. We could not prove a causal relation between increased hsa-miR-132-3p and hsa-miR-212 expression and reduced TGFB1, EP400 and ZBTB5 expression experimentally in CLL cells, as in our hands transfected miRNA mimics of these miRNAs did not alter expression of these genes, nor did miRNA mimics with validated targets used as positive controls affect mRNA level of their target (e.g. hsa-miR-1 on PTK9 expression). In addition, electroporation of anti-miRs did not affect gene expression of validated targets (data not shown). Possibly, in contrast to CLL cell lines, primary CLL cells are not amenable to exogenous manipulation by hsa-miR mimics or inhibitors *in vitro*, and more research will be needed to show a direct causal link between miRNA expression modulation and target mRNA expression in these cells.

We conclude that our results point to a transcriptional response promoting cell cycle in *in vitro* BCR triggered CLL cells. The miRNAs induced might shape the response, with prominent induction of the hsa-miR-132-3p/hsa-miR-212 cluster that targets several anti-proliferative proteins. However, as reported by others and confirmed by our unpublished observations, BCR triggering *in vitro* is not sufficient to induce proliferation of isolated peripheral blood CLL cells. Most likely, additional signals that are present in a suitable micro-environment *in vivo*, such as the lymph node or bone marrow, are missing in *in vitro* culture systems [6]. The identification of these additional stimuli will be interesting to discover new therapeutic options in this at present incurable disease.

## Supporting Information

Figure S1 **Cell cycle initiation leads to DNA synthesis in a fraction of BCR stimulated CLL cells.** Flow cytometric analysis of peripheral blood mononuclear cells stimulated for 48 hours (IgA control stimulated or IgM stimulated) and stained with CD3-FITC, CD19-PE, and for stoichiometric staining of DNA, DRAQ5 was used. Plots show lymphocyte scatter gated (plots A), CD19^+/weak^ CD3^−^ gate cells (plots B), DRAQ5 wideness versus amplitude, allowing to gate on single cells (plots C), and DRAQ5 intensity histogram (histograms D). Figures indicate percentage of CLL cells in S/G2, present in the gated cells. This example shows results of patient CLL17 (IGHV mutated).
(TIFF)

Figure S2 **BCR stimulation of CLL cells induces CCL3 and CCL4 chemokine secretion.** Chemokine CCL3 and CCL4 secretion measured by ELISA in cultures of IgM stimulated CLL cells. Concentration measured (pg/mL) in function of time (hours of stimulation) is shown.
(TIFF)

Figure S3 **BCR stimulation of CLL cells induces reduced CXCR4 gene expression.** Kinetics of expression of CXCR4. Scatter plots show normalized mRNA expression for *IGHV* mutated (M, • ;N = 4) and *IGHV* unmutated cases (U, ▴; N = 4), horizontal lines represent average values. Significant differences are indicated (* p<0.05).
(TIFF)

Figure S4 **CXCR4 and CD19 cell surface expression is reduced after BCR stimulation of CLL cells.** (A) Bivariate dotplots of flow cytometric analysis of CXCR4 versus CD19 expression on PBMC incubated for 24 hours with anti-IgA (left panel) or anti-IgM beads. Events were gated on live cells, a representative sample is shown. (B) Expression of surface membrane CXCR4 in CLL cells stimulated with anti-IgA or anti-IgM beads for 24 hours. Scatter plots show normalized expression (ratio's of mean fluorescence intensity) for *IGHV* mutated (M, • ;N = 7) and *IGHV* unmutated cases (U, ▴; N = 7), horizontal line represent average value. Significant decrease of CXCR4 expression (p<0.05), however not significantly different between *IGHV* mutated and *IGHV* unmutated cases.
(TIFF)

Figure S5 **Unsupervised clustering of samples according to miR expression.** Heat-map shows unsupervised clustering of samples according to expression of all miRNAs detected, highlighted either for mutational status (A, (unmutated black tag, mutated grey tag) or stimulation (B, (anti-IgM stimulated black tag, control IgA stimulated grey tag). From blue over white to red indicates increased miR expression.
(TIF)

Figure S6 **Modulation of gene expression of selected genes upon BCR triggering in CLL cells.** Expression of indicated genes in CLL cells, after 3 hours (A) or 24 hours (B) of stimulation with anti-IgM beads. Fold change to the expression level in CLL cells incubated with anti-IgA beads is shown, grey scale indicate magnitude of fold change for representation purposes. Arrows represent “acts on”, hooks represents “inhibits”. Image constructed using Ingenuity IPA® software.
(TIF)

Figure S7 **Cell cycle control genes are modulated upon BCR stimulation in CLL cells.** Expression of indicated genes in CLL cells, after 24 hours of stimulation with anti-IgM beads. Fold change to the expression level in CLL cells incubated with anti-IgA beads is shown, grey scale indicate magnitude of fold change for representation purposes. Arrows represent “acts on”, hooks represents “inhibits”, P: phosphorylated protein. miR-132/212: hsa-miR-132-3p and hsa-miR-212 miRNA. Hypothetical model, constructed using Ingenuity IPA® software.
(TIFF)

Table S1 Overview of patient samples used.
(PDF)

Table S2 Patient characteristics.
(PDF)

Table S3 Rank-product analysis for significantly (percentage false positive <0.05) up- or downregulated genes (fold change FC at least 2) in samples stimulated for 3 and 24 hours, ranked according to increasing percentage false positive.
(PDF)

Table S4 Gene Set Enrichment Analysis of unstimulated versus stimulated sample categories. Table shows gene sets found significantly enriched in either sample category from gene set collections KEGG (shown in black characters), Gene Ontology Biological Process (shown in blue characters), and Transcription Factor Targets (shown in red characters), ranked according normalized enrichment score (NES). False discovery rate (FDR) q value, based on 1,000 permutations of category labels, was below 0.05. Category indicates sample type gene set is enriched in (negative scores of NES for stimulated samples).
(PDF)

Table S5 miRNAs detected in BCR stimulated CLL cells.
(PDF)

Table S6 Rank-product analysis of detected miRNA, showing fold change FC in samples stimulated for 3 and 24 hours, ranked according to increasing percentage false positive.
(PDF)

Table S7 Canonical pathways associated with modulated genes after 3 hours or 24 hours of BCR stimulation.
(PDF)

Table S8 Correlation coefficient between gene and hsa-miR-132-3p or hsa-miR-212 miRNA expression.
(PDF)

Table S9 Gene sets enriched for genes negatively correlating with hsa-miR-132-3p or hsa-miR-212 miRNA expression.
(PDF)
