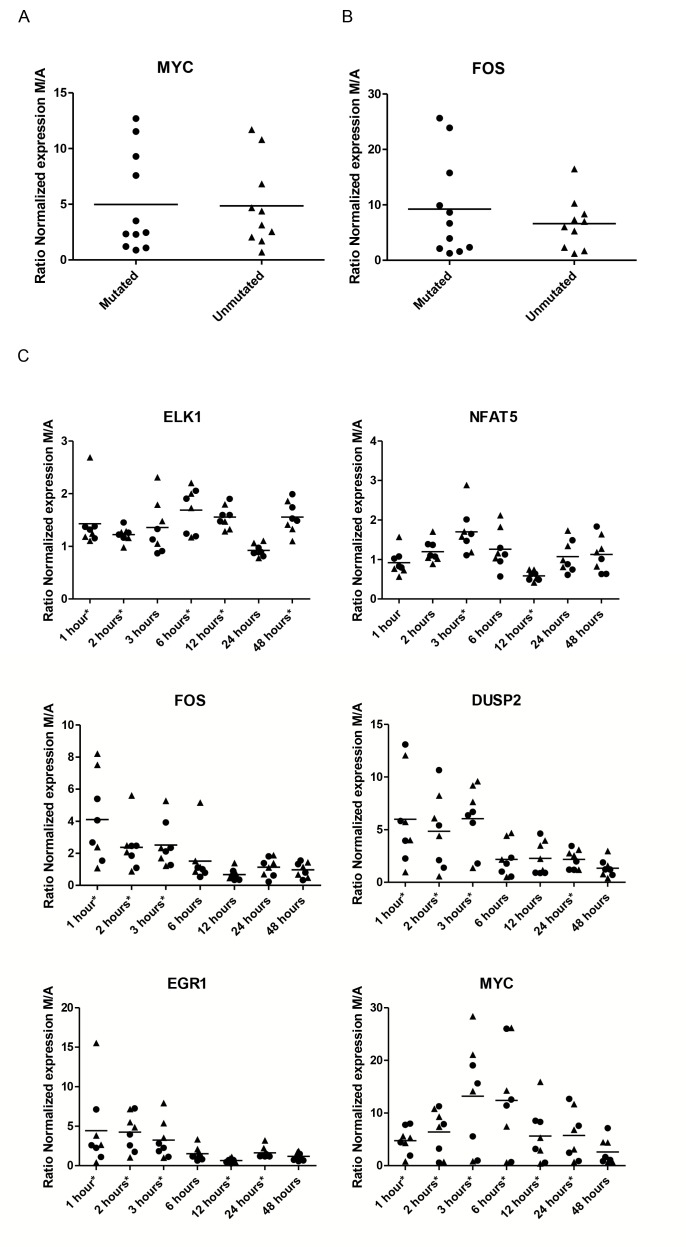# Correction: CLL Cells Respond to B-Cell Receptor Stimulation with a MicroRNA/mRNA Signature Associated with MYC Activation and Cell Cycle Progression

**DOI:** 10.1371/annotation/7ab67087-8bdd-458b-bb77-e03c166a87ca

**Published:** 2014-01-29

**Authors:** Valerie Pede, Ans Rombout, Jolien Vermeire, Evelien Naessens, Pieter Mestdagh, Nore Robberecht, Hanne Vanderstraeten, Nadine Van Roy, Jo Vandesompele, Frank Speleman, Jan Philippé, Bruno Verhasselt

Figure 1 is missing Panel C. The correct version of Figure 1 can be found here: 

**Figure:**