# Supplementary figures and images for: CLL Cells Respond to B-Cell Receptor Stimulation with a MicroRNA/mRNA Signature Associated with MYC Activation and Cell Cycle Progression
Source: PLoS One. 2013 Apr 1;8(4):e60275. doi: 10.1371/journal.pone.0060275 (PMC3613353; doi:10.1371/journal.pone.0060275)

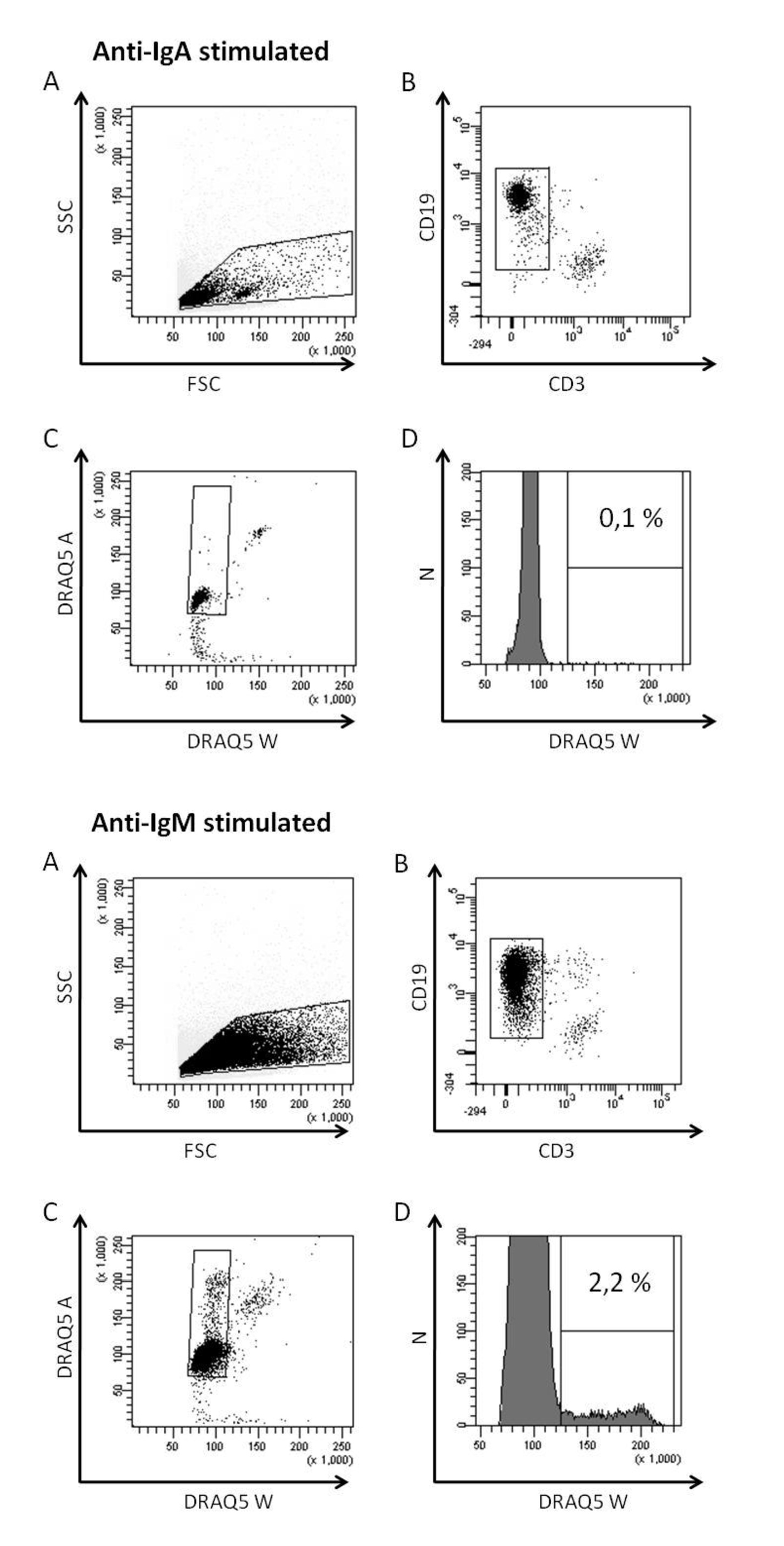

Supplement: Figure S1 — Cell cycle initiation leads to DNA synthesis in a fraction of BCR stimulated CLL cells. Flow cytometric analysis of peripheral blood mononuclear cells stimulated for 48 hours (IgA control stimulated or IgM stimulated) and stained with CD3-FITC, CD19-PE, and for stoichiometric staining of DNA, DRAQ5 was used. Plots show lymphocyte scatter gated (plots A), CD19+/weak CD3− gate cells (plots B), DRAQ5 wideness versus amplitude, allowing to gate on single cells (plots C), and DRAQ5 intensity histogram (histograms D). Figures indicate percentage of CLL cells in S/G2, present in the gated cells. This example shows results of patient CLL17 (IGHV mutated). (TIFF) [file pone.0060275.s001.tiff]

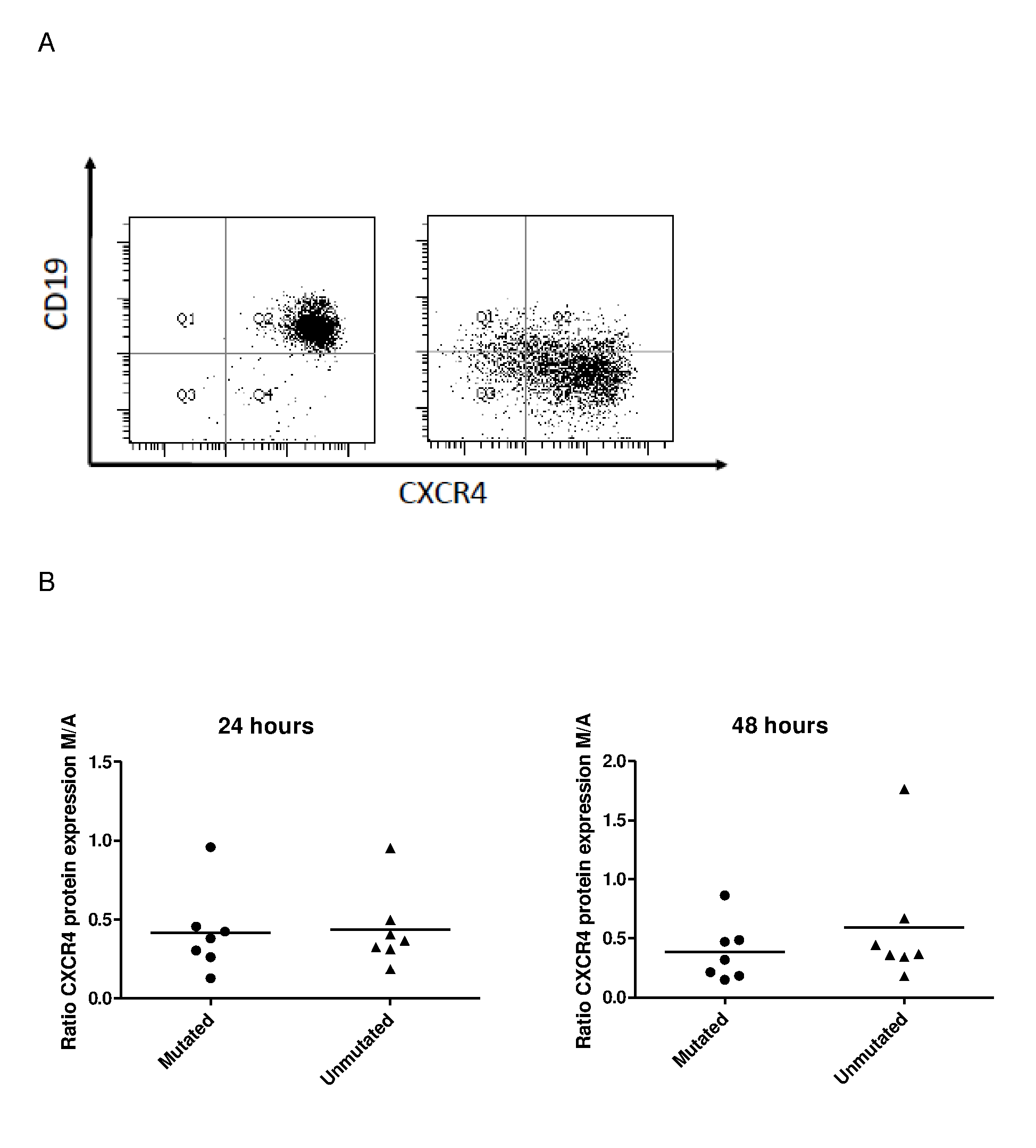

Supplement: Figure S4 — CXCR4 and CD19 cell surface expression is reduced after BCR stimulation of CLL cells. (A) Bivariate dotplots of flow cytometric analysis of CXCR4 versus CD19 expression on PBMC incubated for 24 hours with anti-IgA (left panel) or anti-IgM beads. Events were gated on live cells, a representative sample is shown. (B) Expression of surface membrane CXCR4 in CLL cells stimulated with anti-IgA or anti-IgM beads for 24 hours. Scatter plots show normalized expression (ratio's of mean fluorescence intensity) for IGHV mutated (M, • ;N = 7) and IGHV unmutated cases (U, ▴; N = 7), horizontal line represent average value. Significant decrease of CXCR4 expression (p<0.05), however not significantly different between IGHV mutated and IGHV unmutated cases. (TIFF) [file pone.0060275.s004.tiff]

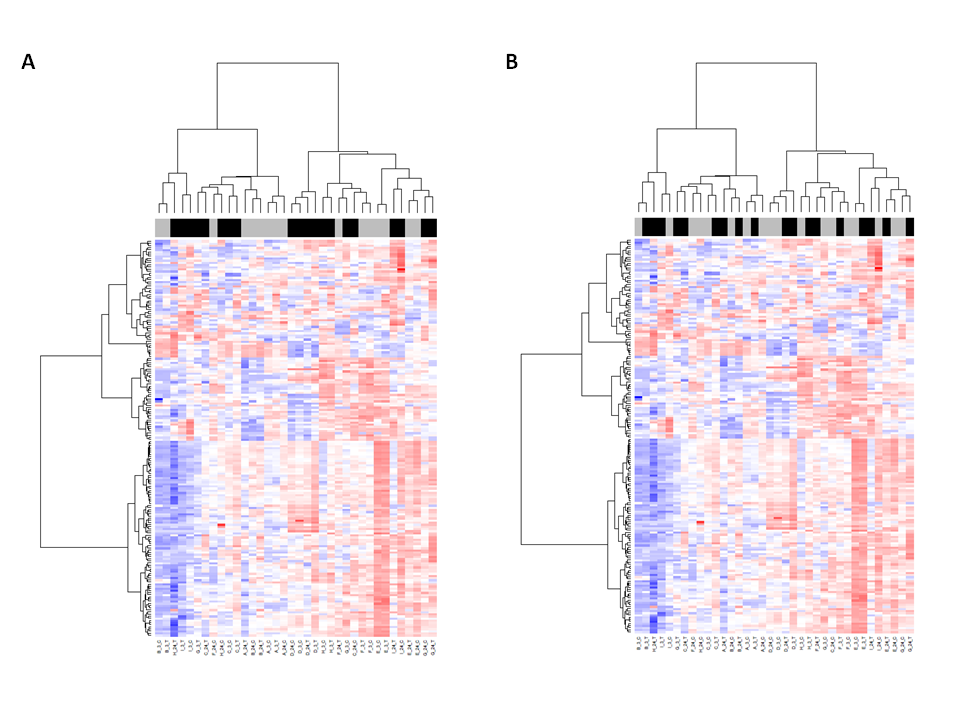

Supplement: Figure S5 — Unsupervised clustering of samples according to miR expression. Heat-map shows unsupervised clustering of samples according to expression of all miRNAs detected, highlighted either for mutational status (A, (unmutated black tag, mutated grey tag) or stimulation (B, (anti-IgM stimulated black tag, control IgA stimulated grey tag). From blue over white to red indicates increased miR expression. (TIF) [file pone.0060275.s005.tif]

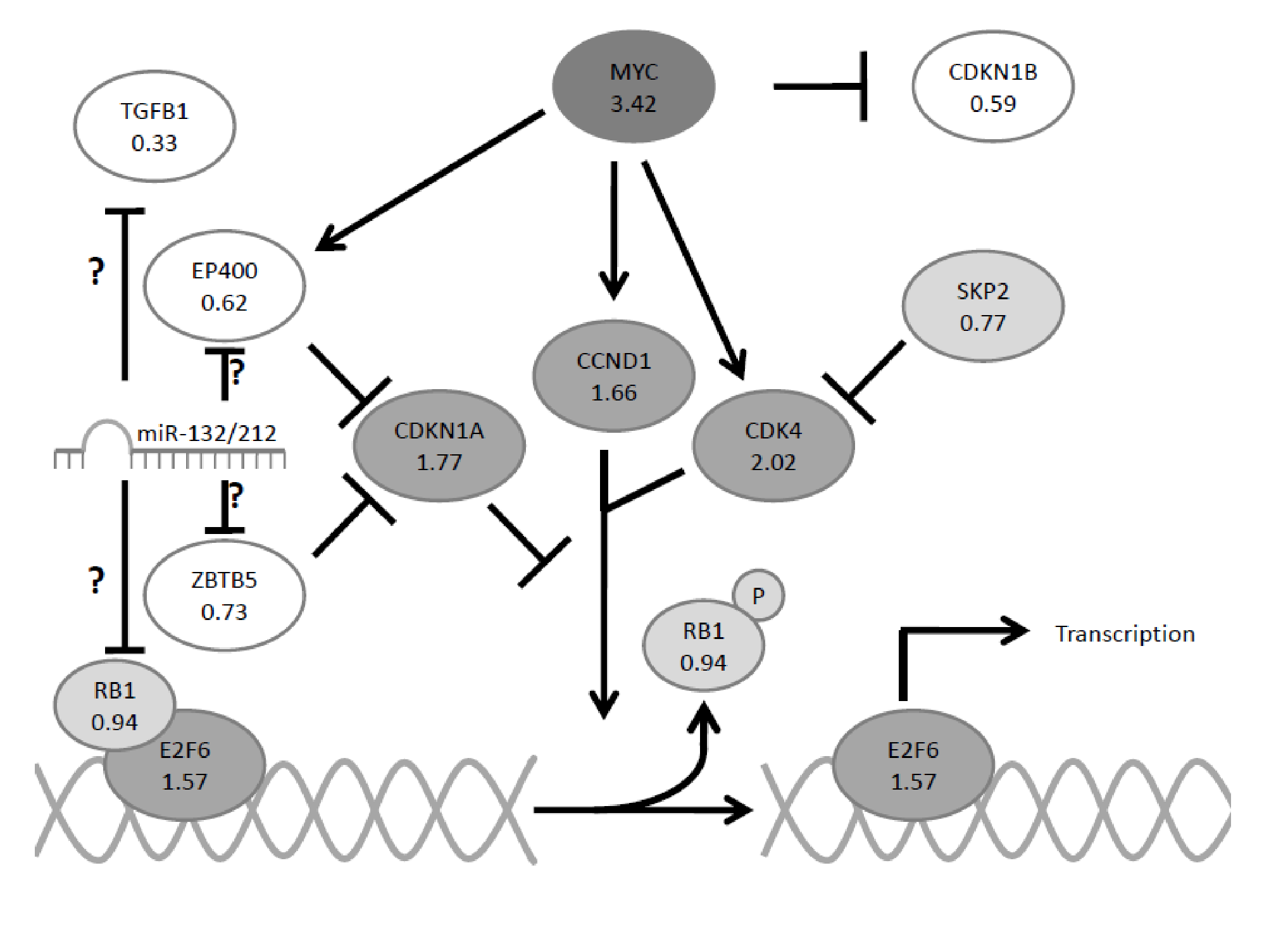

Supplement: Figure S7 — Cell cycle control genes are modulated upon BCR stimulation in CLL cells. Expression of indicated genes in CLL cells, after 24 hours of stimulation with anti-IgM beads. Fold change to the expression level in CLL cells incubated with anti-IgA beads is shown, grey scale indicate magnitude of fold change for representation purposes. Arrows represent “acts on”, hooks represents “inhibits”, P: phosphorylated protein. miR-132/212: hsa-miR-132-3p and hsa-miR-212 miRNA. Hypothetical model, constructed using Ingenuity IPA® software. (TIFF) [file pone.0060275.s007.tif]
