# Supplementary material for: CLL Cells Respond to B-Cell Receptor Stimulation with a MicroRNA/mRNA Signature Associated with MYC Activation and Cell Cycle Progression
Source: PLoS One. 2013 Apr 1;8(4):e60275. doi: 10.1371/journal.pone.0060275 (PMC3613353; doi:10.1371/journal.pone.0060275)
Supplement: Table S1 — Overview of patient samples used. (PDF) [file pone.0060275.s008.pdf]

Table S1

Patient ID	miRNA array qPCR	miRNA single qPCR	mRNA array	qPCR	Kinetic expression miRNA/mRNA qPCR	Cryo/Fresh	Percentage Lymphocytes	Percentage CD19/CD5 before selection (% from lymphocytes)	Percentage CD19 after selection (mean 3h and 24h for both A and M)
CLL-1		■		■		Cryo	85	81	99,3
CLL-2	■	■	■	■		Cryo	92	92	99,5
CLL-3	■	■	■	■		Cryo	89	96	99,0
CLL-4		■		■		Cryo	90	99	99,2
CLL-5		■		■		Fresh	63	82	99,1
CLL-6	■	■		■		Fresh	76	88	99,4
CLL-7	■	■		■		Fresh	73	62	99,2
CLL-8	■	■	■	■		Cryo	46	36	98,2
CLL-9	■	■	■	■		Fresh	76	94	98,7
CLL-10	■	■		■		Fresh	83	93	98,5
CLL-11	■	■		■		Fresh	71	98	98,3
CLL-12	■	■	■	■		Cryo	98	ND	98,6
CLL-13		■		■		Cryo	77	87	98,2
CLL-14				■	■	Cryo	86	90	ND
CLL-15				■	■	Cryo	69	96	ND
CLL-16				■	■	Cryo	75	93	ND
CLL-17				■	■	Cryo	88	97	ND
CLL-18				■	■	Cryo	96	94	ND
CLL-19				■	■	Cryo	97	99	ND
CLL-20				■	■	Cryo	76	94	ND
CLL-21				■	■	Cryo	91	97	ND

Use of samples, according to patient identity (ID). miRNA array qPCR: genome wide combined PCR array using pooled primers in reverse transcription; miRNA single PCR: qPCR using specific reverse transcription primer, mRNA array: genome wide Illumina mRNA array, qPCR: real-time PCR for genes as indicated in text and Fig. 1 and Fig. S1, Kinetic expression miRNA/mRNA qPCR:

real-time PCR for mRNA of genes shown in Fig. 1A and for miRNA shown in Fig. 6. Fresh/Cryo: indicates whether cells were used fresh upon blood sampling or after cryopreservation of mononuclear fraction, Percentage of lymphocytes: in mononuclear fraction in flow cytometry, Percentage of CD19/CD5 before selection: percentage of double positive CD19+CD5+ cells in of lymphocytes in mononuclear fraction, Percentage CD19+ after selection: percentage of CD19+ cells after enrichment from lymphocytes in mononuclear fraction.
