# Supplementary material for: CLL Cells Respond to B-Cell Receptor Stimulation with a MicroRNA/mRNA Signature Associated with MYC Activation and Cell Cycle Progression
Source: PLoS One. 2013 Apr 1;8(4):e60275. doi: 10.1371/journal.pone.0060275 (PMC3613353; doi:10.1371/journal.pone.0060275)
Supplement: Table S2 — Patient characteristics. (PDF) [file pone.0060275.s009.pdf]

Table S2: Patient Characteristics

Patient ID	Sex	Age at diagnosis	Cytogenetic analysis	CLL score	RAI	BINET	VH Usage	Percentage Mutations	Mutation status	ZAP70
CLL-1	M	52	11qdel[2]	5	0	A	3-30	95,7	Mut	N
CLL-2	M	59	13q14 del[1]	4	0	A	3-30	90,1	Mut	N
CLL-3	M	57	No del[1]	5	0	A	3-23	91,1	Mut	N
CLL-4	F	61	17p del[2]	5	0	A	3-21	93,9	Mut	N
CLL-5	M	58	13q14del[1], Other[3]	5	1	A	4-31	96,6	Mut	N
CLL-6	F	46	13q14 del[1]	5	2	B	1-08	93,7	Mut	N
CLL-7	F	34	13q14 del[1]	5	0	A	2-70	95,7	Mut	N
CLL-8	M	49	13q14 del[1]	5	0	A	2-70	99	Unmut	N
CLL-9	F	43	tris 12[1]	4	0	A	1-03	98,2	Unmut	P
CLL-10	F	57	13q14 del[1]	4	0	A	2-70	100	Unmut	P
CLL-11	M	46	13q14del[1] 11q14del[1] Other[4]	5	0	A	5-01	99,5	Unmut	P
CLL-12	F	73	No del[1]	3	0	A	3-30	100	Unmut	P
CLL-13	F	72	13q del[2]	5	0	A	3-33	100	Unmut	P
CLL-14	F	64	Other[2,5]	5	2	A	3-13	95	Mut	N
CLL-15	M	52	13q del[2]	5	1	A	3-07	96,2	Mut	N
CLL-16	M	50	Other[2,6]	5	0	A	4-34	91,4	Mut	N
CLL-17	F	45	Inv 11[2]	5	2	A	4-39	90,3	Mut	N
CLL-18	M	71	13q del[2]	4	0	A	1-69	100	Unmut	P
CLL-19	M	64	No del[2]	5	0	A	4-39	100	Unmut	P
CLL-20	F	74	17p del[2]	5	0	A	3-23	100	Unmut	P
CLL-21	M	52	No del[2]	5	0	A	1-18	98,6	Unmut	P

[1] CGH, [2] FISH, [3] loss 3p21, loss 8p23, loss 10q24,

[4] loss 2p25, [5] loss in chromosome 2,6 and 13, [6] loss in chromosome XY.

Flow cytometry scoring according to Matutes et al.; classical staging systems according to Rai et al. and Binet et al. (reviewed in [2]), M: male, F: female, Mut: mutated, Unmut: Unmutated, N: negative, P: positive
