# Supplementary material for: CLL Cells Respond to B-Cell Receptor Stimulation with a MicroRNA/mRNA Signature Associated with MYC Activation and Cell Cycle Progression
Source: PLoS One. 2013 Apr 1;8(4):e60275. doi: 10.1371/journal.pone.0060275 (PMC3613353; doi:10.1371/journal.pone.0060275)
Supplement: Table S3 — Rank-product analysis for significantly (percentage false positive <0.05) up- or downregulated genes (fold change FC at least 2) in samples stimulated for 3 and 24 hours, ranked according to increasing percentage false positive. (PDF) [file pone.0060275.s010.pdf]

RP analysis 3h		Upregulated			
	gene.index	RP/Rsum	FC:(class1/class2)	pfp	P.value
CCL3L1	4781	47,7908	6,4876	0	0
CCL4L2	4787	42,124	6,0398	0	0
DUSP2	7876	35,8667	6,0191	0	0
CCL3L1	4782	60,9642	5,8548	0	0
FOS	10102	66,2687	5,4153	0	0
CKS2	5638	51,0605	5,3398	0	0
CCL3	4780	55,9837	5,2664	0	0
UBTD1	46765	63,6582	5,2107	0	0
CCL4L1	4786	96,5929	5,0737	0	0
MYC	35990	62,9665	4,8165	0	0
NR4A3	36926	85,9131	4,7764	0	0
C13ORF15	2870	84,503	4,6399	0	0
FOSB	10103	95,8992	4,6133	0	0
PHLDB1	38811	75,0616	4,6028	0	0
EGR1	8140	103,1297	4,5835	0	0
CKS2	5637	78,1984	4,5473	0	0
MGC4677	35242	97,3535	4,4889	0	0
NR4A2	36921	77,1915	4,4584	0	0
CCL3L3	4784	110,2168	4,3239	0	0
RCAN1	40906	96,0508	4,202	0	0
SERPINE2	42408	151,8815	4,0174	0	0
CHRNA1	5545	143,8532	3,9466	0	0
HOMER1	12151	107,4119	3,9331	0	0
TRIB3	46044	100,7997	3,9274	0	0
EGR2	8141	129,175	3,9216	0	0
MYCN	35997	146,9304	3,9042	0	0
TRK1	46182	110,3637	3,8664	0	0
EGR3	8142	131,1695	3,8342	0	0
LOC143666	27442	125,2329	3,8105	0	0
HS.562534	21117	162,4065	3,7078	0	0
DDIT4	7028	137,3448	3,5875	0	0
RNF19A	41367	183,1988	3,5352	0	0
SERTAD1	42417	149,2325	3,5143	0	0
PTGER4	40198	151,274	3,5125	0	0
LOC653506	33238	230,2074	3,4946	0	0
PIM3	38925	161,2095	3,4892	0	0
MYC	35989	190,974	3,4492	0	0
TRQ1	46243	164,3288	3,447	0	0
CHRNA1	5544	201,3756	3,4158	0	0
HS.538259	17211	181,9894	3,4149	0	0
GRAMD4	11314	163,7056	3,3937	0	0
HS.543887	18873	217,4331	3,3781	0	0
RNF19A	41365	169,8218	3,3642	0	0
ATF3	1771	172,5779	3,3265	0	0
KLF10	26575	229,6837	3,2556	0	0

RHOB	41182	182,4627	3,2474	0	0
BTG3	2585	190,2639	3,2267	0	0
C17ORF91	3149	193,3062	3,2248	0	0
MAPK6	34704	205,9858	3,2185	0	0
C10ORF54	2695	233,5395	3,1841	0	0
PDCD1	38417	214,6011	3,1656	0	0
CD200	4921	264,527	3,1319	0	0
EIF2AK3	8175	216,6832	3,0709	0	0
NR4A2	36922	254,6787	2,9884	0	0
SRXN1	44089	216,467	2,983	0	0
SLAMF7	42774	260,4772	2,963	0	0
PRNP	39895	269,3788	2,9584	0	0
SPAG9	43804	223,8219	2,9582	0	0
BLVRB	2388	277,3582	2,9332	0	0
ZNF593	48501	254,3103	2,896	0	0
C5ORF30	3843	266,4126	2,8888	0	0
BTG3	2586	277,4659	2,8657	0	0
TMEM88	45644	279,3586	2,8301	0	0
RAB20	40468	309,7474	2,8166	0	0
HS.374278	15286	277,8714	2,8071	0	0
IDI1	25188	272,252	2,7953	0	0
TNFRSF12A	45736	267,781	2,7801	0	0
MAP2K3	34621	275,3458	2,7652	0	0
RCAN1	40904	283,0264	2,7595	0	0
UGCG	46814	285,1352	2,7493	0	0
LOC650832	32232	307,7838	2,6688	0	0
METRNL	35053	355,2928	2,9115	1,00E-04	0
IL2RB	25481	336,9612	2,7958	1,00E-04	0
WSB2	47590	329,1297	2,7839	1,00E-04	0
LOC653158	33046	345,3456	2,7302	1,00E-04	0
PHACTR1	38731	349,3032	2,7279	1,00E-04	0
AXUD1	2042	353,0192	2,7202	1,00E-04	0
HSPC111	25037	335,2318	2,7018	1,00E-04	0
RRAD	41702	353,7857	2,6777	1,00E-04	0
DDIT3	7027	314,6501	2,6614	1,00E-04	0
C19ORF48	3222	348,54	2,5822	1,00E-04	0
PRRX2	39986	353,0423	2,5727	1,00E-04	0
LPL	34121	393,802	2,8553	2,00E-04	0
GLA	10846	391,1439	2,6606	2,00E-04	0
TUBB2A	46514	367,8022	2,505	2,00E-04	0
LRRC32	34202	410,4958	2,7919	3,00E-04	0
GEM	10703	408,9129	2,6964	3,00E-04	0
CCL3L1	4783	442,8549	2,6823	3,00E-04	0
RGS1	41117	414,6836	2,6152	3,00E-04	0
METTL1	35060	419,9896	2,5915	3,00E-04	0
GEM	10702	449,5579	2,5377	3,00E-04	0
PGM2	38710	427,4431	2,5184	3,00E-04	0

GNA13	10954	428,5191	2,5162	3,00E-04	0
FAM57A	8992	426,7773	2,5123	3,00E-04	0
SLC7A5	43262	446,3838	2,4983	3,00E-04	0
PNO1	39289	406,9919	2,4739	3,00E-04	0
SPAG9	43805	437,6639	2,4711	3,00E-04	0
C19ORF48	3223	421,879	2,4667	3,00E-04	0
NEU1	36454	445,3121	2,4659	3,00E-04	0
RIOK1	41233	410,3403	2,4559	3,00E-04	0
CORO6	6110	449,5004	2,4465	3,00E-04	0
C6ORF114	3875	424,1795	2,4362	3,00E-04	0
PCGF5	38354	456,1099	2,4338	3,00E-04	0
RRAGD	41709	420,4736	2,401	3,00E-04	0
HS.545232	19350	433,391	2,388	3,00E-04	0
ALG13	865	453,1642	2,372	3,00E-04	0
SGK	42540	478,1018	2,6459	4,00E-04	0
SPRY2	43987	469,2504	2,6156	4,00E-04	0
PIF1	38857	484,849	2,5213	4,00E-04	0
DUSP5	7890	487,9102	2,5146	4,00E-04	0
HIF1A	11875	495,0234	2,4625	4,00E-04	0
HS.25892	14514	475,6012	2,4411	4,00E-04	0
PRNP	39894	477,5034	2,441	4,00E-04	0
TRAF1	45973	484,1493	2,4161	4,00E-04	0
IL21R	25451	458,6684	2,4035	4,00E-04	0
MTHFD2	35846	463,7514	2,4015	4,00E-04	0
BXDC1	2623	510,6286	2,3616	4,00E-04	0
NHEDC2	36557	472,7455	2,3279	4,00E-04	0
CYCSL1	6745	489,5476	2,3206	4,00E-04	0
LONRF3	34083	491,4441	2,2853	4,00E-04	0
LOC643930	29906	535,1978	2,619	5,00E-04	0
FAM152B	8873	556,6933	2,4663	5,00E-04	0
RPS24	41637	533,3591	2,409	5,00E-04	0
HMGCS1	12078	544,866	2,4083	5,00E-04	0
ELL2	8275	537,2268	2,3854	5,00E-04	0
RRP15	41727	473,0599	2,3765	5,00E-04	0
MRPS17	35695	534,6665	2,3427	5,00E-04	0
RCN1	40922	557,594	2,3406	5,00E-04	0
YRDC	47736	529,1861	2,2627	5,00E-04	0
NAMPT	36156	583,6362	2,3957	7,00E-04	0
TNFRSF18	45742	566,5102	2,3428	7,00E-04	0
GADD45A	10460	583,6088	2,2733	7,00E-04	0
LOC731049	33952	576,6703	2,2198	7,00E-04	0
MCOLN3	34914	564,0996	2,1761	7,00E-04	0
NFKBID	36522	594,8423	2,3283	8,00E-04	0
PPAT	39490	589,3133	2,266	8,00E-04	0
POLB	39329	596,4428	2,4139	9,00E-04	0
NR4A3	36924	597,45	2,3199	9,00E-04	0
PAK1IP1	38037	598,098	2,2929	9,00E-04	0

DUSP4	7889	595,3225	2,29	9,00E-04	0
TSC22D2	46262	595,7596	2,2867	9,00E-04	0
HES4	11820	612,1661	2,3844	0,001	0
PVT1	40375	615,0481	2,1902	0,001	0
FLJ43663	9913	614,9987	2,154	0,001	0
RHEB	41178	609,6659	2,1428	0,001	0
HEG1	11782	650,6894	2,2985	0,0011	0
VGF	47183	630,2373	2,2793	0,0011	0
KBTBD8	25926	627,511	2,2309	0,0011	0
NOLC1	36760	635,7436	2,2159	0,0011	0
GADD45A	10459	644,1916	2,1952	0,0011	0
KIAA0133	26171	621,1558	2,1768	0,0011	0
TNFSF9	45780	639,5007	2,167	0,0011	0
NIP7	36589	639,4609	2,1607	0,0011	0
HS.544512	19112	649,2663	2,1459	0,0011	0
OAS1	37196	681,3673	2,2069	0,0012	0
BYSL	2627	668,0089	2,1582	0,0012	0
LOC651816	32494	659,2492	2,1563	0,0012	0
SNX8	43654	664,9349	2,1463	0,0012	0
NETO2	36453	689,5192	2,1368	0,0012	0
HS.156550	13522	665,6128	2,1342	0,0012	0
ETF1	8563	687,5429	2,0862	0,0012	0
ZNF643	48551	667,3656	2,068	0,0012	0
C16ORF61	3064	674,236	2,0679	0,0012	0
FILIP1L	9495	665,9757	1,9743	0,0012	0
ADM	542	720,8753	2,3995	0,0013	0
HS.575551	23301	698,9888	2,1993	0,0013	0
MRTO4	35728	697,053	2,1249	0,0013	0
DPH3	7704	714,0497	2,0968	0,0013	0
TNFRSF9	45759	710,3174	2,2602	0,0014	0
GZMB	11600	708,5133	2,2301	0,0014	0
TNFRSF18	45741	710,4978	2,1945	0,0014	0
PRKCH	39843	702,4412	2,1874	0,0014	0
C5ORF32	3844	702,8054	2,1851	0,0014	0
LYAR	34378	712,2923	2,1559	0,0014	0
ZSWIM6	48778	700,4108	2,1315	0,0014	0
DCUN1D5	7003	742,397	2,1262	0,0014	0
GFOD1	10721	706,3565	2,1259	0,0014	0
DNAJB5	7574	742,6149	2,1157	0,0014	0
PDGFA	38496	737,2299	2,1093	0,0014	0
NFATC1	36482	742,8741	2,1042	0,0014	0
SNRPD1	43585	743,8311	2,0681	0,0014	0
EIF2C2	8184	709,9451	2,0488	0,0014	0
LY9	34373	708,1669	2,0404	0,0014	0
SLC3A2	43150	734,7122	2,0252	0,0014	0
FILIP1L	9493	704,0363	1,9285	0,0014	0
C13ORF25	2877	752,268	2,0921	0,0015	0

CENPN	5305	751,3543	2,0274	0,0015	0
FILIP1L	9494	749,3096	1,8783	0,0015	0
EVI2A	8594	764,5378	2,2335	0,0016	0
ICOS	25168	762,7778	2,1776	0,0016	0
RPS7	41690	773,1229	2,1566	0,0016	0
ZNF165	48078	776,7627	2,1424	0,0016	0
JUNB	25889	773,615	2,1104	0,0016	0
ITK	25804	762,5387	2,1068	0,0016	0
DCUN1D5	7002	765,9284	2,1058	0,0016	0
SLAMF1	42771	774,7741	2,0756	0,0016	0
SEH1L	42236	763,9509	2,0356	0,0016	0
HS.537591	17015	768,897	2,0118	0,0016	0
HS.562219	21058	782,6012	2,1326	0,0018	0
DPH3	7705	781,0048	2,0764	0,0018	0
IER2	25194	782,4036	2,0074	0,0018	0
SLCO4A1	43314	801,7001	2,1272	0,0019	0
AMD1	945	801,9425	2,1063	0,0019	0
KLF10	26577	815,2073	2,0714	0,0019	0
STX3	44373	804,4499	2,051	0,0019	0
BXDC2	2624	800,6134	2,0488	0,0019	0
ING1	25564	815,5917	2,0441	0,0019	0
RBBP8	40784	791,8505	2,0397	0,0019	0
PPP1R2	39597	794,5871	2,032	0,0019	0
MAT2A	34793	794,3492	2,0198	0,0019	0
ETF1	8564	814,0066	2,0194	0,0019	0
DUSP4	7888	809,8637	1,961	0,0019	0
LOC143543	27441	807,4122	1,947	0,0019	0
SIAH2	42677	809,7902	2,0789	0,002	0
HIC1	11873	819,0892	2,1404	0,0021	0
DIMT1L	7347	819,9778	2,0844	0,0021	0
DNAJB5	7576	821,1844	2,0358	0,0021	0
RPS7	41691	824,0924	2,0897	0,0022	0
ADO	547	825,1343	2,0481	0,0022	0
ALG13	864	829,7362	1,9925	0,0022	0
C6ORF66	3973	825,4165	1,9751	0,0022	0
CD84	5020	835,4308	2,0887	0,0023	0
MAFF	34469	837,6262	2,0424	0,0023	0
TMEM185B	45529	833,3655	2,027	0,0023	0
MYCN	35998	843,3043	2,0223	0,0023	0
CA2	4211	835,3859	2,0221	0,0023	0
NAMPT	36157	836,4351	2,0186	0,0023	0
HS.543241	18613	837,6126	2,0105	0,0023	0
BCL2	2250	839,2906	2,009	0,0023	0
PTS	40335	835,144	1,9518	0,0023	0
NAB2	36131	842,3436	1,9447	0,0023	0
METRNL	35051	861,3809	2,176	0,0024	0
ZFP36L1	47937	848,0768	2,103	0,0024	0

TXN	46568	862,5865	2,0929	0,0024	0
C14ORF172	2951	859,8615	1,978	0,0024	0
TFIP11	45100	855,2194	1,9631	0,0024	0
CBX3	4545	864,2001	1,991	0,0025	0
SLC25A4	42963	859,7795	1,9852	0,0025	0
STX4	44374	858,662	1,9685	0,0025	0
C3ORF14	3717	880,2125	1,7076	0,0025	0
NME1	36681	869,4592	2,1179	0,0026	0
SUSD1	44469	878,0569	1,9959	0,0026	0
HSPA9	25022	872,2625	1,9696	0,0026	0
EBI2	7985	885,5777	1,9377	0,0026	0
SPAG1	43781	866,1869	1,9362	0,0026	0
FOSL1	10104	870,6502	1,9327	0,0026	0
SEMA7A	42287	887,0474	1,929	0,0026	0
HECTD2	11778	876,2394	1,928	0,0026	0
SQSTM1	44026	899,6175	1,9277	0,0026	0
LOC654244	33519	874,3673	1,9233	0,0026	0
ZC3H8	47845	904,0498	1,9106	0,0026	0
ETNK1	8571	894,5972	1,9001	0,0026	0
SBDS	41975	902,8669	1,8901	0,0026	0
TAGAP	44676	911,3128	2,023	0,0027	0
FAM100B	8747	909,0845	1,9193	0,0027	0
TBC1D19	44769	914,9513	2,3266	0,0028	0
HOOK1	12158	926,4026	2,013	0,0028	0
PER3	38610	912,5665	1,9948	0,0028	0
SESN2	42424	926,5833	1,9799	0,0028	0
MPP6	35556	922,8892	1,9739	0,0028	0
IQGAP2	25666	914,2578	1,9493	0,0028	0
FUBP1	10306	919,3044	1,8893	0,0028	0
GNA13	10953	928,8846	1,95	0,0029	0
PLEKHA7	39133	938,5558	2,0563	0,003	0
GIT1	10806	933,8117	1,9798	0,003	0
MRPL47	35672	934,8812	1,9622	0,003	0
CENTG3	5328	936,3892	1,8724	0,003	0
TIMM8A	45263	937,8705	2,0064	0,0031	0
JMJD3	25864	939,5726	1,9067	0,0031	0
GNL3	11003	942,1322	2,0715	0,0032	0
ZNF263	48173	948,3675	1,9507	0,0032	0
OTUD6B	37893	953,1199	1,9629	0,0033	0
NEDD9	36412	957,3043	1,8818	0,0033	0
PTRH2	40332	971,2655	2,002	0,0034	0
KCNK12	26043	961,6248	1,9894	0,0034	0
CIRH1A	5611	966,9254	1,9097	0,0034	0
TFAM	45063	968,4229	1,9004	0,0034	0
EBI2	7986	974,5088	1,8062	0,0034	0
EIF1AY	8169	974,3061	1,3309	0,0034	0
ARL5B	1532	967,6813	1,9923	0,0035	0

GCLM	10643	976,8359	1,9858	0,0035	0
TNF	45717	988,5884	1,981	0,0035	0
HIF1A	11877	983,0448	1,9725	0,0035	0
NIN	36582	978,5755	1,9349	0,0035	0
LARP2	26991	972,8493	1,8924	0,0035	0
CDKN1A	5196	983,5582	1,8524	0,0035	0
HES6	11822	1009,9772	2,0336	0,0037	0
TXNRD1	46600	999,3431	1,9333	0,0037	0
RRAD	41703	1000,0667	1,9129	0,0037	0
GRPEL1	11424	1009,6999	1,9109	0,0037	0
EYA3	8665	1009,5488	1,8838	0,0037	0
NME1	36683	1028,4863	1,9974	0,0038	0
CTPS	6571	1013,8004	1,9932	0,0038	0
ABCE1	133	1011,8823	1,9665	0,0038	0
MKI67IP	35350	1011,2732	1,9496	0,0038	0
SACS	41903	1025,1172	1,9468	0,0038	0
SELK	42243	1019,8115	1,9258	0,0038	0
SPAG9	43803	1020,9094	1,9043	0,0038	0
ILVBL	25528	1022,3487	1,8941	0,0038	0
C9ORF21	4132	1027,9766	1,8825	0,0038	0
SLC25A4	42964	1013,2804	1,8649	0,0038	0
TCEB1	44893	1011,7983	1,8567	0,0038	0
NUP35	37135	1033,2291	1,9005	0,0039	0
ERF	8502	1032,081	1,8829	0,0039	0
BEX2	2329	1033,0297	1,8011	0,0039	0
SLC4A7	43199	1044,7798	1,9809	0,004	0
IER3	25195	1037,6735	1,9736	0,004	0
USP36	47030	1046,6804	1,9214	0,004	0
SPAG9	43802	1042,0904	1,9203	0,004	0
RBM13	40805	1042,8773	1,8935	0,004	0
WDR43	47418	1050,7846	1,8744	0,004	0
GOT1	11070	1045,4835	1,8513	0,004	0
METRNL	35050	1054,3566	2,026	0,0041	0
NMD3	36679	1067,8174	1,9759	0,0041	0
HS.521338	16616	1059,6963	1,9499	0,0041	0
LOC440093	28569	1057,4334	1,8334	0,0041	0
MESDC1	35039	1065,9589	1,8227	0,0041	0
FKBP1A	9509	1064,2183	1,8188	0,0041	0
CEBPG	5271	1061,7355	1,8168	0,0041	0
MRPL15	35618	1075,7555	1,9516	0,0043	0
CENPM	5302	1072,8205	1,9194	0,0043	0
RPP40	41611	1084,5935	1,9379	0,0044	0
PPAN	39462	1080,3052	1,9187	0,0044	0
SLC3A2	43151	1084,5712	1,8766	0,0044	0
CLLU1OS	5772	1079,0707	1,7125	0,0044	0
LOC652864	32942	1090,3838	1,9214	0,0045	0
TAF9	44670	1088,5287	1,8586	0,0045	0

TIMM10	45252	1097,5317	1,8077	0,0045	0
CD69	4999	1101,2184	1,982	0,0046	0
ARL4A	1526	1102,6831	1,9281	0,0046	0
LYAR	34377	1109,8413	1,9292	0,0048	0
AEBP2	586	1116,629	1,8878	0,0049	0
FYN	10367	1122,0669	2,0181	0,005	0
CCDC86	4734	1122,3386	1,9365	0,005	0
TMEM185B	45530	1125,327	1,8535	0,005	0
LOC374443	27946	1134,5214	1,9385	0,0051	0
STCH	44273	1125,1451	1,8478	0,0051	0
SFPQ	42477	1133,7054	1,8418	0,0051	0
EIF2S1	8188	1124,0284	1,8175	0,0051	0
THUMPD2	45217	1132,0017	1,8105	0,0051	0
AKAP12	746	1137,5532	2,0228	0,0052	0
SRFBP1	44045	1136,4697	1,8304	0,0052	0
C12ORF44	2844	1135,2054	1,7762	0,0052	0
TXN	46569	1148,7007	1,9796	0,0053	0
DDX21	7068	1148,2039	1,908	0,0053	0
C10ORF2	2664	1145,1567	1,9032	0,0053	0
PDE12	38445	1142,856	1,7964	0,0053	0
GADD45G	10462	1153,9415	1,8417	0,0054	0
HMOX1	12091	1156,6445	1,843	0,0055	0
GLYATL2	10920	1162,8488	1,9315	0,0057	0
C6ORF115	3876	1165,0714	1,886	0,0057	0
LOC641825	29006	1162,4464	1,8316	0,0057	0
FXR2	10344	1166,0654	1,8054	0,0057	0
NCOA6IP	36317	1164,1718	1,7846	0,0058	0
ADORA2A	551	1171,5627	1,7083	0,0058	0
HNRNPAB	12113	1180,0005	1,936	0,0059	0
UGT8	46857	1191,1661	1,7687	0,0061	0
UCK2	46791	1201,0142	1,8931	0,0062	0
HIF1A	11876	1196,5677	1,8646	0,0062	0
GABARAPL2	10412	1202,4276	1,8515	0,0062	0
MTHFD2	35848	1197,041	1,8468	0,0062	0
ZNF259	48170	1190,4313	1,8287	0,0062	0
NRAS	36938	1204,1088	1,7695	0,0062	0
E2F6	7971	1210,536	1,7673	0,0064	0
GALNT11	10512	1216,1734	1,8857	0,0065	0
RRP12	41726	1214,8703	1,8767	0,0065	0
TRIM29	46082	1218,842	1,7549	0,0066	0
TFB2M	45078	1229,7494	1,8872	0,0069	1,00E-04
LOC401321	28452	1239,7985	1,8802	0,0071	1,00E-04
CAMK2N2	4358	1238,058	1,858	0,0071	1,00E-04
ISG20L1	25722	1238,8339	1,8495	0,0071	1,00E-04
TUBB2A	46513	1240,2497	1,7655	0,0071	1,00E-04
GTPBP4	11546	1243,9341	1,8485	0,0072	1,00E-04
GADD45B	10461	1243,828	1,831	0,0072	1,00E-04

PTS	40334	1244,8499	1,8002	0,0072	1,00E-04
KPNA4	26715	1247,348	1,7514	0,0072	1,00E-04
NMD3	36680	1254,748	1,8773	0,0073	1,00E-04
SLC25A19	42926	1254,463	1,8097	0,0073	1,00E-04
TLE1	45299	1251,8825	1,8015	0,0073	1,00E-04
MAPKAPK2	34723	1249,4456	1,7892	0,0073	1,00E-04
C1ORF51	3384	1255,7632	1,7666	0,0073	1,00E-04
LY9	34374	1253,1357	1,7201	0,0073	1,00E-04
AKAP12	743	1262,677	1,9891	0,0074	1,00E-04
KIAA0020	26162	1261,4915	1,8594	0,0074	1,00E-04
ATF3	1772	1258,9976	1,8177	0,0074	1,00E-04
IRF2BP2	25687	1258,5902	1,8088	0,0074	1,00E-04
TSC22D2	46263	1261,3192	1,7701	0,0074	1,00E-04
BZW2	2636	1266,8988	1,8918	0,0075	1,00E-04
LARP4	26993	1273,28	1,8479	0,0075	1,00E-04
SELT	42254	1273,8663	1,8162	0,0075	1,00E-04
RNF19A	41366	1271,1049	1,8115	0,0075	1,00E-04
PHLDA1	38808	1263,9819	1,8052	0,0075	1,00E-04
EIF3J	8204	1274,8235	1,7758	0,0075	1,00E-04
RBKS	40791	1269,4989	1,7347	0,0075	1,00E-04
PIM3	38926	1287,4762	1,8029	0,0076	1,00E-04
EIF3A	8191	1282,3453	1,7712	0,0076	1,00E-04
MRPL32	35643	1288,0714	1,7494	0,0076	1,00E-04
CDKN3	5215	1287,9725	1,7474	0,0076	1,00E-04
SNAPC1	43480	1289,3961	1,7337	0,0076	1,00E-04
FABP5	8714	1291,0508	1,7327	0,0076	1,00E-04
ANKRD9	1150	1295,0354	1,8513	0,0077	1,00E-04
PI4K2A	38836	1294,8909	1,8125	0,0077	1,00E-04
WDR74	47464	1297,1747	1,7995	0,0077	1,00E-04
PDSS1	38556	1304,4613	1,8231	0,0078	1,00E-04
ZNF706	48627	1302,4047	1,7945	0,0078	1,00E-04
CAB39L	4224	1304,595	1,7837	0,0078	1,00E-04
NR4A1	36919	1304,9974	1,7666	0,0078	1,00E-04
LOC151579	27504	1312,5682	1,8252	0,0079	1,00E-04
AMD1	946	1310,6006	1,8019	0,0079	1,00E-04
C18ORF19	3156	1315,7485	1,8259	0,008	1,00E-04
FBXO11	9206	1315,5404	1,7611	0,008	1,00E-04
TBC1D7	44797	1323,216	1,8709	0,0081	1,00E-04
CISD1	5613	1324,2449	1,8427	0,0081	1,00E-04
MAP6D1	34676	1326,0515	1,837	0,0081	1,00E-04
BACE2	2104	1323,9326	1,8291	0,0081	1,00E-04
C4ORF32	3811	1322,8787	1,8264	0,0081	1,00E-04
PAK1IP1	38036	1326,6333	1,7797	0,0081	1,00E-04
SEMA6B	42278	1322,1363	1,7433	0,0081	1,00E-04
CYP20A1	6779	1322,6253	1,712	0,0081	1,00E-04
MIDN	35321	1335,0773	1,7971	0,0082	1,00E-04
XBP1	47636	1329,4864	1,752	0,0082	1,00E-04

PEA15	38582	1341,5068	1,7309	0,0084	1,00E-04
PPRC1	39673	1348,9413	1,8138	0,0086	1,00E-04
TMEM107	45412	1351,7375	1,4491	0,0086	1,00E-04
RPL29	41546	1361,4628	1,8136	0,0087	1,00E-04
NDFIP2	36332	1366,2246	1,8074	0,0087	1,00E-04
TIMM9	45265	1362,6851	1,7968	0,0087	1,00E-04
CLDND1	5696	1359,4623	1,7082	0,0087	1,00E-04
GNL3	11004	1366,1713	1,8388	0,0088	1,00E-04
CCNH	4828	1359,4596	1,7674	0,0088	1,00E-04
KIAA1553	26376	1376,2489	1,7702	0,009	1,00E-04
NFATC1	36483	1378,6046	1,7651	0,0091	1,00E-04
CSTF3	6473	1380,4726	1,7529	0,0091	1,00E-04
SAR1B	41955	1391,9534	1,8127	0,0094	1,00E-04
ETNK1	8573	1393,1343	1,8117	0,0094	1,00E-04
GPR137B	11166	1387,4718	1,7613	0,0094	1,00E-04
PELO	38599	1386,7044	1,7515	0,0094	1,00E-04
RCL1	40921	1393,0928	1,7491	0,0094	1,00E-04
HMGA1	12062	1392,6289	1,7378	0,0094	1,00E-04
RG9MTD1	41081	1400,0561	1,8317	0,0095	1,00E-04
CYP51A1	6853	1402,5254	1,8198	0,0095	1,00E-04
BACE2	2105	1401,2321	1,7741	0,0095	1,00E-04
NR3C1	36913	1401,6321	1,7656	0,0095	1,00E-04
BCAR3	2217	1396,8211	1,7561	0,0095	1,00E-04
SLC39A14	43132	1407,2508	1,7994	0,0096	1,00E-04
RBM12	40801	1413,305	1,7993	0,0097	1,00E-04
ABCE1	134	1409,449	1,7966	0,0097	1,00E-04
UBE3A	46731	1411,4587	1,7367	0,0097	1,00E-04
MRPL13	35616	1417,9212	1,8275	0,0098	1,00E-04
FYN	10364	1417,7426	1,8132	0,0098	1,00E-04
POLR1C	39354	1418,1509	1,8018	0,0098	1,00E-04
HECTD2	11776	1412,1901	1,7992	0,0098	1,00E-04
BZW1	2631	1416,1763	1,7053	0,0098	1,00E-04
CD22	4927	1423,5093	1,6705	0,01	1,00E-04
PMM2	39244	1434,5003	1,745	0,0102	1,00E-04
CPEB4	6165	1433,6429	1,7327	0,0102	1,00E-04
ZNF643	48552	1430,4296	1,7223	0,0102	1,00E-04
NARG1	36189	1435,8648	1,7484	0,0103	1,00E-04
MAP1LC3B	34609	1440,5443	1,7326	0,0103	1,00E-04
HNRNPAB	12114	1443,6155	1,8239	0,0104	1,00E-04
GNAI3	10960	1448,5038	1,7911	0,0104	1,00E-04
EXOSC3	8640	1448,0082	1,7683	0,0104	1,00E-04
NOLA1	36755	1442,929	1,7208	0,0104	1,00E-04
TMEM70	45623	1442,8427	1,7048	0,0104	1,00E-04
RAMP1	40643	1448,3409	1,396	0,0104	1,00E-04
RASAL1	40714	1452,5146	1,796	0,0105	1,00E-04
ZBTB32	47802	1451,7136	1,678	0,0105	1,00E-04
C6ORF66	3974	1456,4345	1,7724	0,0106	1,00E-04

EEF1E1	8069	1458,7067	1,8118	0,0107	1,00E-04
LOC728153	33622	1460,3067	1,7122	0,0107	1,00E-04
TXNRD1	46601	1461,9734	1,8449	0,0108	1,00E-04
ISG15	25720	1465,016	1,7918	0,0108	1,00E-04
MAPKAP1	34719	1466,1351	1,7465	0,0108	1,00E-04
ACSL1	306	1466,0737	1,7451	0,0108	1,00E-04
THOP1	45193	1468,9172	1,6791	0,0108	1,00E-04
ECD	7994	1469,5286	1,6618	0,0108	1,00E-04
KIAA0090	26165	1468,1264	1,6614	0,0108	1,00E-04
SRC	44033	1471,4235	1,7799	0,0109	1,00E-04
NXT1	37187	1474,3184	1,6572	0,011	1,00E-04
OPN1MW	37316	1484,9486	1,8574	0,0112	1,00E-04
EIF1AX	8167	1486,2447	1,7683	0,0112	1,00E-04
SLC12A6	42800	1482,7067	1,6926	0,0112	1,00E-04
POLR2D	39364	1485,846	1,6812	0,0112	1,00E-04
IFI30	25203	1491,795	1,7844	0,0113	1,00E-04
KLF4	26592	1494,1404	1,7753	0,0113	1,00E-04
LY6E	34358	1492,0296	1,7619	0,0113	1,00E-04
SLC1A5	42858	1496,5578	1,7377	0,0113	1,00E-04
TP53INP2	45892	1493,9614	1,7273	0,0113	1,00E-04
HAMP	11649	1498,6258	1,7267	0,0113	1,00E-04
C15ORF15	2997	1497,2408	1,7041	0,0113	1,00E-04
LOC731682	33986	1496,4735	1,3803	0,0114	1,00E-04
KIF21A	26480	1505,1816	1,8189	0,0115	1,00E-04
ECE2	7996	1502,1695	1,7751	0,0115	1,00E-04
FAM131A	8838	1511,8663	1,6832	0,0117	1,00E-04
UPP1	46949	1514,2064	1,7539	0,0118	1,00E-04
MAFG	34473	1512,5153	1,6788	0,0118	1,00E-04
HS.193767	13905	1520,0633	1,7961	0,0119	1,00E-04
FKBP5	9520	1521,5823	1,7484	0,0119	1,00E-04
ZNF410	48314	1524,005	1,6816	0,0119	1,00E-04
MRPS12	35691	1523,4043	1,6602	0,0119	1,00E-04
RBM28	40824	1528,5348	1,7215	0,012	1,00E-04
SLC3A2	43149	1537,2002	1,6696	0,0122	1,00E-04
ARF4	1383	1533,7803	1,6413	0,0122	1,00E-04
DPH3	7706	1536,9311	1,6865	0,0123	1,00E-04
ADNP2	546	1544,8932	1,7169	0,0124	1,00E-04
TOP1P2	45860	1547,9913	1,7044	0,0124	1,00E-04
BACH2	2110	1542,6769	1,7038	0,0124	1,00E-04
DCTN6	6993	1543,8676	1,649	0,0124	1,00E-04
SC4MOL	41988	1553,751	1,6458	0,0125	1,00E-04
ZC3H15	47836	1558,0702	1,7369	0,0126	1,00E-04
JOSD1	25870	1553,4135	1,7325	0,0126	1,00E-04
NAB1	36130	1551,7763	1,7108	0,0126	1,00E-04
SAR1A	41953	1555,7633	1,706	0,0126	1,00E-04
LOC143543	27440	1555,1573	1,6613	0,0126	1,00E-04
PER2	38609	1555,0305	1,6415	0,0126	1,00E-04

PTGS2	40211	1558,2135	1,6286	0,0126	1,00E-04
MPDU1	35533	1561,8304	1,6864	0,0127	1,00E-04
METRNL	35054	1570,0508	1,8241	0,0129	1,00E-04
STX11	44361	1573,1842	1,7736	0,0129	1,00E-04
DDX3X	7083	1574,6693	1,6713	0,0129	1,00E-04
ZNF706	48629	1576,2006	1,7424	0,013	1,00E-04
NFATC1	36485	1579,5676	1,7034	0,013	1,00E-04
CHCHD8	5451	1577,4301	1,6933	0,013	1,00E-04
VAPA	47117	1581,2649	1,686	0,013	1,00E-04
CTNNAL1	6550	1581,5151	1,6235	0,013	1,00E-04
GCLM	10644	1584,0624	1,6946	0,0131	1,00E-04
FAM35A	8935	1584,9246	1,633	0,0131	1,00E-04
ZNF331	48251	1586,1366	1,6281	0,0131	1,00E-04
ARHGEF12	1469	1588,1872	1,6722	0,0132	1,00E-04
C11ORF31	2755	1595,1581	1,7116	0,0134	1,00E-04
GPRIN1	11285	1596,761	1,6533	0,0134	1,00E-04
TFB2M	45079	1605,1345	1,7711	0,0137	2,00E-04
TIMM23	45260	1604,892	1,7154	0,0137	2,00E-04
ATP6V1F	1950	1606,2492	1,7139	0,0137	2,00E-04
HNRNPAB	12115	1615,2872	1,7719	0,0139	2,00E-04
AZIN1	2050	1618,817	1,7328	0,0141	2,00E-04
PPAN-P2RY11	39463	1623,0413	1,6968	0,0141	2,00E-04
USP9X	47065	1618,2789	1,6566	0,0141	2,00E-04
KLK1	26654	1633,7479	1,7195	0,0143	2,00E-04
RPS4Y2	41667	1632,8177	1,6479	0,0143	2,00E-04
MAFF	34472	1633,6515	1,6445	0,0143	2,00E-04
LONRF3	34084	1638,2458	1,6408	0,0143	2,00E-04
TOLLIP	45836	1638,0958	1,6387	0,0143	2,00E-04
CLDND1	5697	1633,5289	1,6227	0,0143	2,00E-04
PYCR1	40401	1646,6447	1,7575	0,0145	2,00E-04
ACSL4	313	1644,1462	1,7088	0,0145	2,00E-04
CCT4	4884	1649,91	1,6181	0,0146	2,00E-04
CALU	4338	1655,81	1,6335	0,0147	2,00E-04
TIMM23	45259	1659,5512	1,6811	0,0148	2,00E-04
PRR6	39972	1662,0113	1,7351	0,0149	2,00E-04
ADARB1	487	1662,3677	1,7202	0,0149	2,00E-04
UBIAD1	46739	1662,2441	1,6712	0,0149	2,00E-04
RBM39	40834	1663,8824	1,6584	0,0149	2,00E-04
HS.489254	16273	1667,4285	1,7346	0,015	2,00E-04
NUCB2	37068	1678,8294	1,7066	0,0153	2,00E-04
LOC653980	33437	1677,012	1,7002	0,0153	2,00E-04
KLF6	26595	1673,9864	1,679	0,0153	2,00E-04
PSPC1	40148	1679,6284	1,6517	0,0153	2,00E-04
TLE3	45301	1675,5359	1,5877	0,0153	2,00E-04
MCTS1	34922	1687,6345	1,721	0,0154	2,00E-04
CCT5	4885	1684,7723	1,6911	0,0154	2,00E-04
NANS	36166	1682,0108	1,6673	0,0154	2,00E-04

PHF19	38772	1684,6722	1,6424	0,0154	2,00E-04
EIF3M	8206	1692,7129	1,6995	0,0156	2,00E-04
ENO2	8345	1693,1636	1,6938	0,0156	2,00E-04
TNFSF14	45771	1692,5342	1,689	0,0156	2,00E-04
EAF2	7976	1703,8767	1,7523	0,0159	2,00E-04
CD40LG	4975	1704,7476	1,7248	0,0159	2,00E-04
SLC25A25	42937	1700,6251	1,6763	0,0159	2,00E-04
TUBB2C	46516	1704,7584	1,6674	0,0159	2,00E-04
ATP1B3	1852	1701,5568	1,649	0,0159	2,00E-04
EIF5A2	8239	1702,4552	1,6349	0,0159	2,00E-04
NOLA3	36759	1703,223	1,5847	0,0159	2,00E-04
MAPRE2	34729	1715,5065	1,6886	0,0161	2,00E-04
TNFRSF4	45752	1717,3901	1,6397	0,0161	2,00E-04
MAP1LC3A	34608	1714,4997	1,6306	0,0161	2,00E-04
LOC644128	30020	1714,5165	1,6111	0,0161	2,00E-04
CTLA4	6542	1712,0821	1,4333	0,0161	2,00E-04
GSG1L	11445	1720,5143	1,6705	0,0162	2,00E-04
HSGT1	24988	1722,3133	1,5888	0,0163	2,00E-04
HBB	11683	1722,7433	1,1921	0,0163	2,00E-04
NCL	36307	1728,4327	1,6851	0,0165	2,00E-04
ARL8B	1549	1728,2347	1,6287	0,0165	2,00E-04
CREM	6270	1732,1171	1,6719	0,0166	2,00E-04
TAGAP	44675	1731,2361	1,6569	0,0166	2,00E-04
BXDC2	2625	1734,2138	1,6552	0,0166	2,00E-04
HBA2	11681	1732,2518	1,4245	0,0166	2,00E-04
PPP2CA	39614	1740,7307	1,6732	0,0168	2,00E-04
HECTD2	11777	1738,1507	1,6732	0,0168	2,00E-04
GALNT1	10508	1740,5068	1,6695	0,0168	2,00E-04
NOLA1	36756	1739,6684	1,6636	0,0169	2,00E-04
TMEM2	45550	1745,0165	1,6426	0,0169	2,00E-04
ICHTHYIN	25161	1750,1882	1,7093	0,0171	2,00E-04
SRGN	44056	1749,3045	1,6893	0,0171	2,00E-04
HBA2	11682	1751,7544	1,2724	0,0171	2,00E-04
SDCBP	42131	1754,666	1,6764	0,0172	2,00E-04
HS.534439	16886	1755,3393	1,6378	0,0172	2,00E-04
AK2	723	1762,9777	1,7305	0,0174	2,00E-04
UTP11L	47070	1762,139	1,6914	0,0175	2,00E-04
CAPRIN1	4406	1766,3021	1,6253	0,0175	2,00E-04
VPS37B	47265	1762,5144	1,5911	0,0175	2,00E-04
ZEB2	47909	1766,7	1,6901	0,0176	2,00E-04
TCEB1	44892	1768,3182	1,6633	0,0176	2,00E-04
ECE2	7997	1770,9599	1,6716	0,0177	2,00E-04
WDR12	47365	1775,624	1,6915	0,0179	2,00E-04
KLF10	26576	1778,583	1,669	0,0179	2,00E-04
DNAJB11	7557	1778,5081	1,6458	0,0179	2,00E-04
UAP1	46629	1778,1799	1,6759	0,018	2,00E-04
HIST2H2AC	11979	1781,6467	1,6139	0,018	2,00E-04

RPL34	41559	1784,5652	1,7198	0,0181	2,00E-04
CD69	5000	1794,0075	1,7824	0,0182	2,00E-04
SCD	42024	1792,4856	1,4723	0,0182	2,00E-04
GABPB2	10426	1793,186	1,6677	0,0183	2,00E-04
FKBP2	9514	1791,3436	1,5969	0,0183	2,00E-04
HIST2H2AA3	11976	1803,9182	1,6337	0,0185	2,00E-04
RQCD1	41701	1811,2024	1,6149	0,0185	2,00E-04
NAF1	36141	1804,8843	1,587	0,0185	2,00E-04
PSMD12	40119	1805,4197	1,5634	0,0185	2,00E-04
MRPL50	35675	1810,5894	1,6972	0,0186	2,00E-04
WDR12	47366	1815,6824	1,6752	0,0186	2,00E-04
ARMCX6	1576	1806,1918	1,6437	0,0186	2,00E-04
SNRPG	43593	1810,5013	1,6174	0,0186	2,00E-04
MAGOH	34552	1811,0691	1,5774	0,0186	2,00E-04
ZNF26	48171	1813,8532	1,7175	0,0187	2,00E-04
GFI1	10716	1810,1998	1,6302	0,0187	2,00E-04
FNDC3A	10083	1810,2622	1,6091	0,0187	2,00E-04
SP4	43766	1814,9262	1,6056	0,0187	2,00E-04
OTUD6B	37892	1824,6193	1,6141	0,019	2,00E-04
KLF9	26600	1826,2886	1,5385	0,0191	2,00E-04
PHF5A	38787	1830,6551	1,6585	0,0192	2,00E-04
C1ORF97	3434	1831,3498	1,6411	0,0192	2,00E-04
LOC649555	31874	1834,3077	1,6823	0,0193	3,00E-04
CA2	4212	1834,479	1,6093	0,0193	3,00E-04
PEF1	38591	1834,9394	1,5559	0,0193	3,00E-04
CCNYL1	4846	1842,5814	1,6657	0,0195	3,00E-04
HLA-DRB6	12027	1840,3193	1,3165	0,0195	3,00E-04
NUP35	37134	1846,1554	1,6485	0,0196	3,00E-04
TIPARP	45277	1846,1811	1,5863	0,0196	3,00E-04
C1ORF52	3385	1860,0963	1,6203	0,0198	3,00E-04
SHF	42640	1852,2144	1,6041	0,0198	3,00E-04
ELK1	8268	1853,2237	1,5774	0,0198	3,00E-04
SUB1	44397	1861,8199	1,6826	0,0199	3,00E-04
NCBP2	36287	1857,1099	1,65	0,0199	3,00E-04
C18ORF1	3151	1865,3373	1,6245	0,0199	3,00E-04
BOAT	2445	1859,4191	1,6192	0,0199	3,00E-04
BA16L21.2.1	2097	1863,5633	1,5847	0,0199	3,00E-04
DLEU2	7453	1870,5653	1,7094	0,02	3,00E-04
PTPN22	40277	1868,0225	1,6316	0,02	3,00E-04
C1ORF97	3433	1868,3255	1,6129	0,02	3,00E-04
RBM12	40802	1867,1678	1,5962	0,02	3,00E-04
C19ORF10	3179	1868,4614	1,5712	0,02	3,00E-04
TAF9	44671	1878,5568	1,6486	0,0202	3,00E-04
TIMM23	45257	1874,9541	1,6275	0,0202	3,00E-04
EXOSC6	8643	1883,0598	1,5949	0,0203	3,00E-04
SNORD34	43550	1886,6767	1,6861	0,0204	3,00E-04
NEDD9	36410	1882,6001	1,5526	0,0204	3,00E-04

LOC644943	30387	1890,0186	1,7144	0,0205	3,00E-04
TIMM23	45258	1893,2981	1,6549	0,0205	3,00E-04
POLR1C	39355	1891,5484	1,6496	0,0205	3,00E-04
HS.555181	20418	1889,9226	1,6461	0,0205	3,00E-04
C15ORF5	3029	1895,0605	1,6676	0,0206	3,00E-04
LOC201164	27569	1897,42	1,6719	0,0207	3,00E-04
MCOLN3	34913	1897,7379	1,5846	0,0207	3,00E-04
ETNK1	8572	1898,9586	1,565	0,0207	3,00E-04
LOC144383	27446	1896,6822	1,6531	0,0208	3,00E-04
BATF3	2173	1906,0821	1,6996	0,0211	3,00E-04
CGI-96	5427	1911,076	1,6695	0,0211	3,00E-04
GFM1	10718	1910,1005	1,6596	0,0211	3,00E-04
PRKCQ	39845	1910,6965	1,6346	0,0211	3,00E-04
MRPS7	35721	1907,4943	1,588	0,0211	3,00E-04
RPL29	41545	1916,5516	1,6854	0,0212	3,00E-04
ADCY3	500	1918,6311	1,5959	0,0212	3,00E-04
MAFG	34474	1915,6159	1,5579	0,0212	3,00E-04
G3BP1	10385	1923,8148	1,686	0,0214	3,00E-04
RAN	40648	1924,4741	1,676	0,0214	3,00E-04
ATF4	1776	1922,6819	1,661	0,0214	3,00E-04
LMBR1	27308	1926,3447	1,6465	0,0214	3,00E-04
PINX1	38930	1921,6899	1,621	0,0214	3,00E-04
SBDSP	41976	1925,7476	1,5792	0,0214	3,00E-04
C9ORF105	4091	1932,1852	1,6821	0,0215	3,00E-04
SAMSN1	41937	1930,9551	1,6649	0,0215	3,00E-04
COPS2	6078	1932,7505	1,6546	0,0215	3,00E-04
EIF4A1	8208	1928,9415	1,6485	0,0215	3,00E-04
LOC649009	31716	1931,1131	1,6151	0,0215	3,00E-04
PTTG1	40337	1930,2268	1,5863	0,0215	3,00E-04
RBMX	40866	1939,2688	1,6063	0,0218	3,00E-04
CTLA4	6544	1938,5709	1,393	0,0218	3,00E-04
CEBPZ	5272	1941,0875	1,5763	0,0219	3,00E-04
C7ORF11	3988	1943,7799	1,5404	0,0219	3,00E-04
CNIH4	5878	1947,785	1,6886	0,022	3,00E-04
SNX16	43621	1954,344	1,6467	0,0221	3,00E-04
GMFB	10935	1956,6784	1,6004	0,0222	3,00E-04
MRPL27	35635	1953,3593	1,5809	0,0222	3,00E-04
WDSOF1	47489	1971,2372	1,6455	0,0229	3,00E-04
CLECL1	5740	1975,9499	1,6862	0,0232	3,00E-04
LOC440926	28689	1979,1046	1,6567	0,0232	3,00E-04
MRPL47	35671	1977,5169	1,6391	0,0232	3,00E-04
NFIL3	36512	1979,2898	1,6231	0,0232	3,00E-04
LOC440145	28575	1984,4791	1,571	0,0235	3,00E-04
HCST	11729	1995,7861	1,6348	0,0238	3,00E-04
CCNC	4809	1990,1879	1,6315	0,0238	3,00E-04
TOMM40	45846	1993,5211	1,6175	0,0238	3,00E-04
LOC221710	27605	1994,8374	1,5731	0,0238	3,00E-04

PHF6	38789	1991,5922	1,5663	0,0238	3,00E-04
CCNH	4829	2002,8916	1,577	0,0241	4,00E-04
MSI2	35772	2001,1485	1,5738	0,0241	3,00E-04
FAM96B	9098	2001,3789	1,5378	0,0241	3,00E-04
ILVBL	25529	2006,4524	1,5703	0,0242	4,00E-04
RASGRP1	40729	2013,7785	1,675	0,0244	4,00E-04
HS.444152	15946	2013,6894	1,6053	0,0245	4,00E-04
MGC4677	35241	2018,9245	1,7687	0,0246	4,00E-04
SLC20A1	42861	2016,3499	1,5787	0,0246	4,00E-04
CD86	5021	2022,3377	1,6329	0,0248	4,00E-04
HS.569162	22477	2028,8757	1,6207	0,025	4,00E-04
HMGCR	12077	2033,2504	1,6342	0,0252	4,00E-04
YWHAG	47753	2038,3776	1,6326	0,0253	4,00E-04
TMEM170	45507	2040,5505	1,6036	0,0253	4,00E-04
CCNYL1	4845	2034,9155	1,6	0,0253	4,00E-04
CIITA	5603	2038,1038	1,6521	0,0254	4,00E-04
NEK6	36436	2042,2588	1,6261	0,0254	4,00E-04
TAGAP	44674	2042,4522	1,5847	0,0254	4,00E-04
LONRF1	34080	2037,1824	1,5845	0,0254	4,00E-04
CYP20A1	6780	2038,1027	1,5612	0,0254	4,00E-04
COX17	6120	2043,6701	1,5406	0,0254	4,00E-04
BXDC5	2626	2048,7793	1,5827	0,0257	4,00E-04
E2F3	7967	2057,565	1,583	0,0258	4,00E-04
RPL41	41579	2055,7472	1,6331	0,0259	4,00E-04
ERN1	8525	2056,1607	1,6247	0,0259	4,00E-04
PHF5A	38788	2057,5642	1,616	0,0259	4,00E-04
CST7	6461	2057,4002	1,599	0,0259	4,00E-04
GUF1	11572	2057,0369	1,529	0,0259	4,00E-04
ATP6V0A2	1924	2063,6121	1,6532	0,0261	4,00E-04
TFRC	45107	2064,3468	1,634	0,0261	4,00E-04
HSPE1	25048	2071,7986	1,6336	0,0264	4,00E-04
RRN3	41725	2070,5459	1,6106	0,0264	4,00E-04
TESK1	45038	2075,4631	1,4466	0,0265	4,00E-04
CAPZA1	4416	2086,0894	1,6452	0,027	4,00E-04
SNORD68	43568	2086,4798	1,5888	0,027	4,00E-04
ADPRHL2	560	2092,3004	1,5948	0,0273	4,00E-04
IRF2BP2	25688	2098,3025	1,552	0,0276	4,00E-04
OAS1	37197	2099,303	1,7174	0,0277	4,00E-04
CDKN1A	5197	2100,2541	1,6074	0,0277	4,00E-04
EIF1	8164	2103,3132	1,5742	0,0279	4,00E-04
KIAA0672	26244	2105,597	1,504	0,028	4,00E-04
HS.548831	19829	2111,4909	1,5946	0,0282	4,00E-04
CD164	4907	2114,34	1,63	0,0283	4,00E-04
NIN	36583	2116,2425	1,6026	0,0284	4,00E-04
MRPL36	35650	2116,915	1,5914	0,0284	4,00E-04
FYN	10365	2123,4932	1,6825	0,0286	4,00E-04
ERGIC2	8509	2122,3597	1,6213	0,0286	4,00E-04

TRAF5	45985	2122,2371	1,5597	0,0286	4,00E-04
NCOA7	36318	2126,2795	1,5576	0,0286	4,00E-04
FNDC3A	10081	2124,1048	1,5423	0,0286	4,00E-04
ATP1B3	1854	2128,8833	1,6171	0,0287	4,00E-04
EIF5B	8242	2138,0941	1,5323	0,0292	5,00E-04
ARL5A	1530	2141,6269	1,6318	0,0293	5,00E-04
MAP2K3	34625	2141,4388	1,5092	0,0294	5,00E-04
GOSR2	11068	2147,166	1,6008	0,0295	5,00E-04
RPL6	41583	2146,5589	1,5881	0,0295	5,00E-04
XBP1	47637	2145,748	1,5678	0,0295	5,00E-04
CD55	4988	2151,6303	1,6115	0,0296	5,00E-04
U2AF1	46619	2149,7424	1,5421	0,0296	5,00E-04
TMEM93	45650	2151,4312	1,5306	0,0296	5,00E-04
KCTD9	26135	2148,7809	1,5283	0,0296	5,00E-04
KLF6	26597	2152,7087	1,5862	0,0297	5,00E-04
CCND1	4810	2153,678	1,5235	0,0297	5,00E-04
IRF8	25698	2155,8684	1,6302	0,0298	5,00E-04
CD63	4995	2157,549	1,6142	0,0299	5,00E-04
TIMM44	45261	2162,1349	1,5392	0,0299	5,00E-04
PSMD8	40132	2160,9613	1,5002	0,0299	5,00E-04
LTV1	34345	2167,6406	1,6083	0,0301	5,00E-04
B4GALT5	2093	2167,126	1,5952	0,0301	5,00E-04
IRF4	25690	2169,2627	1,6421	0,0302	5,00E-04
MTRR	35898	2172,4948	1,5224	0,0303	5,00E-04
ASNS	1723	2177,4982	1,532	0,0306	5,00E-04
PLK3	39180	2180,0859	1,5686	0,0307	5,00E-04
RALGDS	40635	2180,9637	1,5627	0,0307	5,00E-04
SDCBP	42132	2184,9522	1,5796	0,0309	5,00E-04
CXORF26	6690	2184,333	1,5732	0,0309	5,00E-04
LCP1	27080	2187,7788	1,5663	0,031	5,00E-04
NFAT5	36480	2189,6129	1,5449	0,031	5,00E-04
PHC2	38744	2193,0112	1,5243	0,0311	5,00E-04
C12ORF49	2849	2193,5278	1,5178	0,0311	5,00E-04
LOC440145	28576	2196,0479	1,5514	0,0313	5,00E-04
LOC651524	32413	2198,8535	1,5457	0,0314	5,00E-04
RBBP6	40778	2198,2951	1,5066	0,0314	5,00E-04
GNPDA1	11012	2206,3557	1,6127	0,0317	5,00E-04
CTNNAL1	6551	2206,3514	1,5312	0,0318	5,00E-04
PHF19	38771	2210,543	1,5301	0,0319	5,00E-04
BTBD7	2572	2208,6097	1,5033	0,0319	5,00E-04
RAP1B	40670	2212,8099	1,618	0,032	5,00E-04
RAP1A	40667	2215,4492	1,5495	0,0322	5,00E-04
MRPL17	35620	2221,1346	1,6149	0,0323	5,00E-04
TNS3	45828	2229,1107	1,6432	0,0327	5,00E-04
PIGR	38880	2227,2715	1,521	0,0327	5,00E-04
CNKSR3	5881	2235,2253	1,5381	0,0331	5,00E-04
FAM36A	8937	2239,0564	1,5633	0,0333	5,00E-04

FGFRL1	9454	2238,8143	1,5409	0,0333	5,00E-04
SLC30A5	43053	2239,9557	1,5303	0,0333	5,00E-04
PTGS1	40209	2243,4547	1,3962	0,0335	6,00E-04
EVI2A	8595	2248,2303	1,5849	0,0336	6,00E-04
INSIG1	25606	2247,2825	1,5804	0,0336	6,00E-04
TUBB4Q	46519	2246,0724	1,5796	0,0336	6,00E-04
SPATA2L	43840	2248,1476	1,5625	0,0336	6,00E-04
GPR65	11250	2247,7212	1,4988	0,0336	6,00E-04
WDR74	47462	2250,2983	1,6119	0,0337	6,00E-04
EIF6	8243	2250,601	1,557	0,0337	6,00E-04
IFRD1	25257	2259,2876	1,5757	0,0339	6,00E-04
NIN	36585	2258,8756	1,5718	0,034	6,00E-04
DNTTIP2	7657	2256,7577	1,5657	0,034	6,00E-04
MFN1	35096	2260,1274	1,5538	0,034	6,00E-04
LOC650128	32029	2258,5684	1,5379	0,034	6,00E-04
STAG3L4	44221	2256,2847	1,5282	0,034	6,00E-04
RN7SK	41264	2257,8813	1,4323	0,034	6,00E-04
PLEK	39121	2263,2996	1,6462	0,0342	6,00E-04
HS.551538	20056	2265,2345	1,4696	0,0343	6,00E-04
EHMT1	8152	2266,7614	1,5089	0,0344	6,00E-04
SPRYD4	43994	2271,078	1,6098	0,0346	6,00E-04
HIST2H2AA3	11977	2273,6413	1,5736	0,0347	6,00E-04
BANP	2152	2273,5217	1,5535	0,0347	6,00E-04
CCDC59	4696	2277,1115	1,5696	0,0348	6,00E-04
DAZAP1	6915	2274,8177	1,5477	0,0348	6,00E-04
DUS3L	7855	2276,3141	1,5456	0,0348	6,00E-04
SLC7A1	43251	2277,29	1,5368	0,0348	6,00E-04
AK2	725	2282,6063	1,6035	0,035	6,00E-04
C9ORF123	4104	2283,5835	1,5313	0,035	6,00E-04
LOC652481	32720	2289,9959	1,569	0,0353	6,00E-04
SHFM1	42643	2295,4198	1,5316	0,0357	6,00E-04
UBXD5	46779	2295,1065	1,5043	0,0357	6,00E-04
ASNS	1724	2297,8199	1,5373	0,0358	6,00E-04
ATHL1	1807	2296,6705	1,4668	0,0358	6,00E-04
RGS16	41132	2301,5565	1,6063	0,036	6,00E-04
C10ORF119	2650	2301,1599	1,4882	0,036	6,00E-04
ZYX	48800	2304,1188	1,5086	0,0361	6,00E-04
CSNK1D	6419	2303,6467	1,4935	0,0361	6,00E-04
BANP	2150	2310,1315	1,5074	0,0364	6,00E-04
HS.545589	19490	2309,5362	1,5878	0,0365	6,00E-04
CHMP2B	5504	2309,7928	1,531	0,0365	6,00E-04
ALCAM	814	2308,5632	1,5093	0,0365	6,00E-04
SGPP2	42557	2317,3726	1,5622	0,0366	6,00E-04
NGLY1	36552	2319,4598	1,5593	0,0366	6,00E-04
LOC731314	33968	2317,0454	1,5269	0,0366	6,00E-04
BCCIP	2228	2318,3372	1,5163	0,0366	6,00E-04
LOC389293	28118	2317,0237	1,5956	0,0367	6,00E-04

IVNS1ABP	25830	2315,247	1,5871	0,0367	6,00E-04
SELS	42252	2316,3685	1,5483	0,0367	6,00E-04
KLK2	26667	2320,6623	1,5285	0,0367	6,00E-04
CD86	5022	2316,9331	1,4526	0,0367	6,00E-04
STRAP	44345	2322,5791	1,5667	0,0368	6,00E-04
EEF1B2	8063	2327,3788	1,6007	0,037	6,00E-04
EEF1B2	8061	2328,1	1,5724	0,037	6,00E-04
S100A8	41882	2332,2072	1,2791	0,0372	7,00E-04
GPSM1	11293	2333,8387	1,5789	0,0373	7,00E-04
UTP15	47074	2336,2076	1,5228	0,0373	7,00E-04
LAT1-3TM	27014	2343,1073	1,5952	0,0375	7,00E-04
SOCS3	43665	2345,6243	1,5941	0,0375	7,00E-04
TSPYL2	46347	2341,5514	1,5257	0,0375	7,00E-04
LY9	34375	2339,8161	1,516	0,0375	7,00E-04
ATP6V1C1	1942	2345,9205	1,5015	0,0375	7,00E-04
TMEM93	45649	2342,5248	1,4911	0,0375	7,00E-04
PHEX	38748	2342,1342	1,4911	0,0375	7,00E-04
NRIP1	36957	2344,2165	1,4465	0,0375	7,00E-04
RIF1	41217	2341,4814	1,5785	0,0376	7,00E-04
LOC648024	31452	2351,6627	1,5199	0,0379	7,00E-04
MRPL12	35615	2357,4682	1,6015	0,0382	7,00E-04
SAR1B	41956	2363,2366	1,5669	0,0386	7,00E-04
TXNL2	46597	2365,847	1,5849	0,0387	7,00E-04
NME2	36687	2368,4289	1,5817	0,0387	7,00E-04
ZNF295	48212	2367,8522	1,5786	0,0387	7,00E-04
UBQLN1	46754	2367,3257	1,5031	0,0387	7,00E-04
C9ORF80	4185	2372,1433	1,5467	0,0388	7,00E-04
VPS37A	47263	2376,2587	1,5513	0,0389	7,00E-04
DDX39	7081	2374,9152	1,5038	0,0389	7,00E-04
HRK	12277	2377,1576	1,537	0,039	7,00E-04
HS.565734	21899	2378,7494	1,5307	0,039	7,00E-04
SNAPC1	43479	2375,9481	1,5224	0,039	7,00E-04
SETDB2	42442	2376,2568	1,5123	0,039	7,00E-04
HS.143018	13141	2381,6188	1,5687	0,0391	7,00E-04
HIVEP3	11991	2381,9402	1,5432	0,0391	7,00E-04
BRUNOL6	2536	2384,4734	1,5361	0,0391	7,00E-04
FAHD1	8731	2384,1019	1,5319	0,0391	7,00E-04
C3ORF37	3750	2383,6421	1,5248	0,0391	7,00E-04
CD58	4990	2389,163	1,5736	0,0394	7,00E-04
NDRG1	36337	2389,4327	1,5199	0,0394	7,00E-04
INTS6	25630	2390,7755	1,5068	0,0394	7,00E-04
ATF5	1777	2391,8319	1,3075	0,0394	7,00E-04
PRDM1	39735	2396,0394	1,4704	0,0396	7,00E-04
CCT3	4882	2399,2075	1,5666	0,0398	7,00E-04
NOL1	36736	2400,3751	1,563	0,0398	7,00E-04
RRS1	41730	2407,6722	1,5738	0,04	7,00E-04
LOC100008589	27345	2405,4632	1,565	0,04	7,00E-04

GRWD1	11433	2406,3876	1,5142	0,04	7,00E-04
C20ORF100	3469	2405,2658	1,3796	0,04	7,00E-04
BOLA2	2452	2410,7591	1,6175	0,0401	7,00E-04
TWISTNB	46562	2410,021	1,5371	0,0401	7,00E-04
SFRS7	42507	2413,081	1,6215	0,0402	7,00E-04
JTV1	25884	2422,5773	1,5711	0,0406	7,00E-04
CHD1	5453	2422,0742	1,5161	0,0406	7,00E-04
SNORA10	43514	2435,6545	1,5905	0,0411	8,00E-04
RPS15	41623	2429,7142	1,5721	0,0411	8,00E-04
SPRED1	43964	2433,3178	1,5352	0,0411	8,00E-04
ATPBD1B	1977	2436,2063	1,4654	0,0411	8,00E-04
CRY1	6331	2430,6058	1,3426	0,0411	8,00E-04
HAT1	11675	2435,3748	1,5637	0,0412	8,00E-04
OAS1	37195	2435,603	1,5384	0,0412	8,00E-04
SEH1L	42234	2433,0289	1,5285	0,0412	8,00E-04
LOC205251	27586	2441,139	1,5514	0,0414	8,00E-04
C12ORF5	2850	2448,0315	1,5822	0,0418	8,00E-04
RGS10	41119	2447,9439	1,4762	0,0418	8,00E-04
PISD	38952	2453,8311	1,5142	0,0419	8,00E-04
TMEM93	45651	2451,8043	1,4703	0,0419	8,00E-04
PSMD14	40122	2453,7258	1,5533	0,042	8,00E-04
GABARAPL2	10413	2454,7038	1,5231	0,042	8,00E-04
SLC12A2	42794	2457,045	1,5432	0,0421	8,00E-04
UBE3A	46730	2463,3757	1,4678	0,0424	8,00E-04
SLC2A14	43033	2471,5171	1,5205	0,0428	8,00E-04
CYCS	6744	2476,3855	1,5474	0,0431	8,00E-04
B3GNT2	2073	2480,2931	1,4795	0,0432	8,00E-04
TDG	44976	2481,3842	1,5263	0,0433	8,00E-04
GPATCH4	11089	2483,2357	1,5372	0,0434	8,00E-04
ISOC2	25731	2486,5343	1,5664	0,0435	8,00E-04
FUS	10316	2486,3771	1,5302	0,0435	8,00E-04
HAX1	11678	2484,6457	1,5027	0,0435	8,00E-04
OPN1LW	37315	2486,3535	1,5732	0,0436	8,00E-04
PRDM13	39740	2489,2	1,475	0,0436	8,00E-04
EHD4	8149	2493,224	1,5241	0,0439	8,00E-04
AP3M2	1245	2494,6238	1,4734	0,0439	8,00E-04
KIAA0194	26178	2498,2977	1,4686	0,0441	8,00E-04
SBDSP	41977	2501,3131	1,4666	0,0442	8,00E-04
FOXP1	10167	2504,2292	1,4911	0,0443	8,00E-04
EZH2	8674	2507,6207	1,4456	0,0445	9,00E-04
TGFBR3	45122	2509,6845	1,3474	0,0445	9,00E-04
HLA-DRB1	12022	2508,1278	1,0655	0,0445	9,00E-04
CCT2	4881	2521,4351	1,561	0,0449	9,00E-04
LRRC8B	34251	2520,2119	1,5234	0,045	9,00E-04
SLC43A3	43163	2520,8846	1,5197	0,045	9,00E-04
PTMA	40230	2520,8286	1,5169	0,045	9,00E-04
NDFIP1	36331	2521,3766	1,503	0,045	9,00E-04

SFRS12	42487	2518,8207	1,5	0,045	9,00E-04
PRCP	39731	2526,0296	1,496	0,045	9,00E-04
TXNL1	46595	2525,5916	1,5839	0,0451	9,00E-04
PRDX4	39766	2527,0694	1,5592	0,0451	9,00E-04
ZDHHC9	47905	2519,9161	1,5552	0,0451	9,00E-04
ATP1B3	1856	2528,0894	1,5768	0,0452	9,00E-04
NME1	36682	2529,7959	1,5204	0,0452	9,00E-04
C20ORF45	3535	2535,8576	1,484	0,0456	9,00E-04
LOC650803	32222	2534,8416	1,4787	0,0456	9,00E-04
INSIG1	25607	2544,3219	1,5311	0,046	9,00E-04
PPM2C	39565	2543,055	1,5273	0,046	9,00E-04
HS.570700	22682	2542,0378	1,4963	0,046	9,00E-04
SRGN	44055	2549,788	1,5409	0,0463	9,00E-04
ARF4	1384	2554,5403	1,4723	0,0467	9,00E-04
SDSL	42179	2556,9083	1,5754	0,0468	9,00E-04
RAB8B	40542	2557,6252	1,5059	0,0468	9,00E-04
HSPC171	25040	2556,4923	1,4573	0,0468	9,00E-04
LOC649679	31902	2563,7646	1,5078	0,0472	9,00E-04
NUP98	37151	2567,5279	1,4545	0,0473	9,00E-04
FAM89A	9071	2570,3139	1,5238	0,0475	9,00E-04
PLEKHO2	39166	2572,8385	1,494	0,0476	9,00E-04
HIST1H4K	11974	2576,473	1,5612	0,0479	9,00E-04
KLHDC4	26605	2580,2536	1,4811	0,0482	0,001
CYBRD1	6742	2581,0997	1,4752	0,0482	0,001
OXCT2	37916	2583,2113	1,5245	0,0484	0,001
SNRPB2	43582	2586,8176	1,5135	0,0485	0,001
FAM133B	8844	2593,8359	1,5623	0,0486	0,001
SMARCA5	43378	2590,0172	1,5006	0,0486	0,001
UBE2L3	46701	2594,9242	1,4667	0,0486	0,001
NELF	36440	2590,7396	1,4618	0,0486	0,001
PARVB	38147	2592,8546	1,4491	0,0486	0,001
WARS2	47322	2591,446	1,3916	0,0486	0,001
UFM1	46811	2596,5423	1,5186	0,0487	0,001
WDR33	47396	2592,66	1,5018	0,0487	0,001
EIF4G1	8226	2598,8705	1,51	0,0488	0,001
SARS	41960	2599,4964	1,5062	0,0488	0,001
RBM15	40807	2601,2131	1,4892	0,0488	0,001
POMC	39401	2603,2043	1,2977	0,049	0,001
CD48	4983	2604,9365	1,4998	0,0491	0,001
CD84	5019	2606,6204	1,5347	0,0492	0,001
MGC72080	35274	2616,2895	1,5198	0,0496	0,001
GNL1	11001	2620,1767	1,4883	0,0498	0,001
NIN	36581	2621,1065	1,5502	0,0499	0,001

RP analysis 3h **Downregulated**

TargetID	gene.index	RP/Rsum	FC:(class1/class2)	pfp	P.value
TXNIP	46594	40,9925	0,2003	0,0000	0
CD79B	5010	33,5714	0,2035	0,0000	0
CD79B	5012	34,1783	0,2084	0,0000	0
CD79B	5011	35,981	0,2091	0,0000	0
CXCR4	6673	40,4772	0,2237	0,0000	0
CYBASC3	6739	70,6472	0,2855	0,0000	0
SEMA4B	42266	86,022	0,2866	0,0000	0
PRICKLE1	39796	140,4758	0,3054	0,0000	0
CXCR4	6671	140,8089	0,3094	0,0000	0
BCL11A	2245	201,4012	0,3216	0,0000	0
NUAK2	37061	122,4769	0,3267	0,0000	0
FLOT2	10035	134,1675	0,3276	0,0000	0
CTDSP2	6524	142,9422	0,3315	0,0000	0
C6ORF32	3955	199,4907	0,3485	0,0000	0
PIM2	38923	205,3361	0,3513	0,0000	0
LYL1	34386	227,1297	0,3568	0,0000	0
C13ORF18	2872	260,7566	0,3656	0,0000	0
KIAA0430	26218	236,6312	0,3684	0,0000	0
PDE7A	38481	220,898	0,3685	0,0000	0
UCP2	46798	295,7427	0,3738	0,0000	0
NUB1	37063	204,5154	0,374	0,0000	0
NUAK2	37060	197,2479	0,3744	0,0000	0
P2RY8	37964	225,8174	0,3747	0,0000	0
PRPSAP1	39950	250,7005	0,3763	0,0000	0
CDKN1B	5198	250,6623	0,3782	0,0000	0
HSH2D	24989	254,9401	0,3809	0,0000	0
BMF	2394	303,8056	0,3872	0,0000	0
ARL6IP5	1545	316,6044	0,3896	0,0000	0
FGD3	9399	321,1481	0,3936	0,0000	0
MKNK2	35359	336,2406	0,3958	0,0000	0
BMF	2391	346,4796	0,3969	0,0000	0
C13ORF18	2873	368,7064	0,3972	0,0000	0
ETS1	8576	310,2842	0,3979	0,0000	0
TRAF3IP3	45981	278,8346	0,4013	0,0000	0
MGC3207	35192	306,0392	0,4034	0,0000	0
SASH3	41966	422,6777	0,4039	0,0000	0
PTPRCAP	40300	357,6091	0,4074	0,0000	0
FAM65A	9007	388,7241	0,4077	0,0000	0
PDE4B	38457	361,4249	0,4099	0,0000	0
IFFO	25200	312,4724	0,4099	0,0000	0
SYVN1	44597	306,8149	0,4106	0,0000	0

TEAD2	44999	383,5441	0,4107	0,0000	0
GGA2	10740	430,585	0,4125	0,0000	0
C5ORF29	3841	420,8685	0,4168	0,0000	0
TLR10	45315	393,7453	0,4185	0,0000	0
SAMD9L	41934	357,4698	0,419	0,0000	0
ST6GALNAC4	44181	382,2626	0,4207	0,0000	0
PEX11B	38619	329,1028	0,4215	0,0000	0
PIK3IP1	38902	443,8191	0,4239	0,0000	0
MGC3207	35189	359,0172	0,4254	0,0000	0
SH2D3C	42574	385,0374	0,4281	0,0000	0
NAPSB	36185	384,1975	0,4285	0,0000	0
C20ORF177	3507	353,5925	0,4291	0,0000	0
PSCD1	40022	443,2872	0,4321	0,0000	0
NCF1	36291	397,784	0,4369	0,0000	0
NKTR	36621	401,5351	0,4391	0,0000	0
ATG4C	1798	430,0478	0,4414	0,0000	0
ILK	25524	447,0267	0,4421	0,0000	0
KLHL14	26622	425,4808	0,4427	0,0000	0
CDC40	5085	445,8812	0,4444	0,0000	0
TUSC4	46552	439,2738	0,4541	0,0000	0
FLJ39827	9841	454,6553	0,4574	0,0000	0
VAMP1	47104	450,9351	0,4611	0,0000	0
THYN1	45220	464,4875	0,4626	0,0000	0
MOBKL2A	35484	483,8783	0,4353	0,0001	0
ADAM28	420	482,1463	0,4365	0,0001	0
TNK2	45786	474,0261	0,4614	0,0001	0
FGD2	9398	472,7379	0,4515	0,0002	0
GRAP	11318	503,1153	0,4256	0,0003	0
APOL3	1327	510,132	0,4358	0,0003	0
PSCD4	40027	509,3241	0,4432	0,0003	0
SYK	44515	516,0588	0,4488	0,0003	0
NAPSB	36186	513,837	0,4561	0,0003	0
TUBB	46511	506,9499	0,4571	0,0003	0
THYN1	45222	507,9469	0,4723	0,0003	0
C1ORF162	3320	518,9224	0,4602	0,0004	0
ST6GALNAC4	44183	538,8458	0,4643	0,0004	0
C10ORF73	2720	536,2681	0,4651	0,0004	0
SELL	42245	539,3715	0,4427	0,0005	0
HS.356079	15128	541,394	0,4499	0,0005	0
PARP4	38134	539,7643	0,4507	0,0005	0
ST3GAL5	44166	543,2083	0,4543	0,0005	0
BCL11A	2246	551,3788	0,4594	0,0005	0
FOXJ2	10139	549,8991	0,4647	0,0005	0
E2F5	7969	557,1686	0,4693	0,0005	0
GNAI2	10959	547,4727	0,473	0,0005	0
PHACTR4	38736	542,7028	0,474	0,0005	0
TNFSF10	45760	564,8955	0,4641	0,0007	0

TRIM8	46164	573,2504	0,4716	0,0007	0
RASSF7	40764	566,4387	0,4719	0,0007	0
FLJ40142	9848	571,1055	0,477	0,0007	0
VPREB3	47227	598,2797	0,4298	0,0009	0
ZNF439	48340	590,0073	0,4573	0,0009	0
DCK	6963	592,4852	0,4673	0,0009	0
SKP2	42761	601,2554	0,487	0,0009	0
TRABD	45968	605,9047	0,4606	0,001	0
CLSTN1	5793	622,8753	0,4874	0,001	0
SPOCK2	43947	616,0713	0,449	0,0011	0
CD79A	5007	620,1001	0,4679	0,0011	0
RAB40C	40520	609,7792	0,4719	0,0011	0
CD37	4963	613,6008	0,4729	0,0011	0
EDG1	8036	610,1096	0,4764	0,0011	0
SLC46A3	43184	616,1654	0,4814	0,0011	0
SLC37A4	43112	617,7345	0,4848	0,0011	0
TLR7	45323	648,5847	0,4506	0,0012	0
USF1	46972	648,0004	0,4769	0,0012	0
UNC93B1	46909	648,8435	0,4773	0,0012	0
LOC339344	27846	647,3267	0,4834	0,0012	0
CCDC115	4592	654,0241	0,4935	0,0012	0
LTB	34325	688,6305	0,4489	0,0013	0
HS.185764	13839	635,1208	0,4673	0,0013	0
BCL3	2271	687,6278	0,4796	0,0013	0
CSK	6402	638,2692	0,4832	0,0013	0
YPEL2	47732	643,6461	0,4849	0,0013	0
CD37	4964	635,0248	0,4858	0,0013	0
C4ORF34	3813	657,0381	0,4868	0,0013	0
SLC25A28	42946	645,61	0,487	0,0013	0
CCM2	4795	686,3496	0,4914	0,0013	0
TTC13	46374	637,654	0,4924	0,0013	0
GORASP1	11063	690,3847	0,4989	0,0013	0
LOC652493	32727	701,3805	0,6466	0,0013	0
EVI2B	8598	696,4242	0,4709	0,0014	0
LOC90925	34054	694,4212	0,4746	0,0014	0
ALDH5A1	843	671,2452	0,4815	0,0014	0
ALOX5AP	907	681,2858	0,4817	0,0014	0
PDLIM1	38527	673,7568	0,4835	0,0014	0
GIT2	10807	676,3092	0,4838	0,0014	0
ZYG11B	48798	698,4122	0,4894	0,0014	0
IL10RA	25361	679,1399	0,4906	0,0014	0
LOC649923	31961	667,3545	0,5063	0,0014	0
IRF5	25691	696,1561	0,5066	0,0014	0
ZMYND8	48031	669,4294	0,5079	0,0014	0
IGLL1	25311	672,5402	0,6468	0,0014	0
STAT2	44255	722,0587	0,4943	0,0017	0
GCHFR	10636	720,4361	0,4944	0,0017	0

UBAC1	46637	714,6729	0,4959	0,0017	0
MCM7	34905	714,7832	0,5004	0,0017	0
THAP11	45154	720,9945	0,5114	0,0017	0
ARHGEF3	1476	748,27	0,484	0,0019	0
ARHGDIB	1456	767,9257	0,4863	0,0019	0
TLR10	45314	730,4858	0,4916	0,0019	0
PYCARD	40398	748,58	0,5003	0,0019	0
TIAF1	45226	757,0688	0,5016	0,0019	0
LOC728499	33672	743,2838	0,5057	0,0019	0
RALGPS1	40636	736,904	0,5073	0,0019	0
ANKZF1	1158	745,1932	0,5077	0,0019	0
HS.282467	14660	758,0396	0,5085	0,0019	0
FAM96A	9096	735,0778	0,5089	0,0019	0
EPN1	8439	747,3078	0,5095	0,0019	0
C17ORF44	3097	731,8013	0,5135	0,0019	0
DNASE2	7621	767,2552	0,519	0,0019	0
ULK1	46869	790,1056	0,4949	0,002	0
RHBDF2	41165	788,5231	0,5147	0,002	0
PIK3CD	38900	777,8582	0,5152	0,002	0
LRCH4	34137	791,4303	0,5266	0,002	0
SIPA1	42716	784,1896	0,4789	0,0021	0
C6ORF105	3868	819,6706	0,4811	0,0021	0
CD5	4984	809,2002	0,4875	0,0021	0
PDE4B	38456	824,584	0,502	0,0021	0
ZNF821	48700	783,7627	0,5027	0,0021	0
MXD4	35963	825,5956	0,505	0,0021	0
REPIN1	40993	798,9017	0,5055	0,0021	0
BLK	2378	820,0127	0,5061	0,0021	0
TNFRSF17	45740	801,9222	0,5065	0,0021	0
PHF15	38764	825,9164	0,5091	0,0021	0
WAS	47323	804,8853	0,5105	0,0021	0
MANBA	34585	820,2893	0,5111	0,0021	0
NCF1C	36293	797,5154	0,5117	0,0021	0
DIS3L	7371	784,9249	0,5128	0,0021	0
ZDHHC8	47904	805,8297	0,5167	0,0021	0
MAP3K4	34647	806,3828	0,5172	0,0021	0
CARM1	4431	826,6574	0,5254	0,0021	0
TBC1D13	44764	806,7588	0,5278	0,0021	0
C9ORF45	4156	829,1548	0,5124	0,0022	0
MAPBPIP	34682	833,113	0,5242	0,0022	0
PLEKHA2	39127	850,5336	0,4977	0,0023	0
SLC7A7	43268	850,4589	0,5005	0,0023	0
MKNK2	35360	844,844	0,5043	0,0023	0
TSPAN33	46326	858,2551	0,5053	0,0023	0
REEP5	40966	856,7584	0,5057	0,0023	0
CTPS2	6573	861,1779	0,5127	0,0023	0
PSMB9	40095	848,1869	0,5142	0,0023	0

ORAI2	37800	879,9225	0,5159	0,0023	0
RHBDF2	41167	864,9344	0,518	0,0023	0
ZNF337	48259	836,1666	0,5193	0,0023	0
ECH1	8003	862,4816	0,5205	0,0023	0
FAM39E	8945	867,4901	0,5226	0,0023	0
LOC647000	31120	835,049	0,5246	0,0023	0
C1ORF2	3352	850,5355	0,5257	0,0023	0
ECOP	8013	883,8214	0,5263	0,0023	0
MICB	35312	839,6682	0,5289	0,0023	0
AP2S1	1236	869,1753	0,5324	0,0023	0
FCRLB	9347	849,2285	0,5327	0,0023	0
BTN2A1	2593	836,8201	0,5349	0,0023	0
LOC647450	31278	865,9157	0,6877	0,0023	0
RERE	41002	893,3079	0,5109	0,0024	0
RAB24	40477	877,3966	0,5138	0,0024	0
ERCC5	8493	846,4568	0,5208	0,0024	0
DYRK2	7948	894,9029	0,5236	0,0024	0
NOD2	36730	883,8033	0,5237	0,0024	0
ALDH3A2	831	874,2674	0,5237	0,0024	0
SNX27	43637	883,4933	0,5263	0,0024	0
C1ORF63	3397	891,5236	0,5288	0,0024	0
ACAD8	214	874,5263	0,5299	0,0024	0
PRDM2	39749	884,6407	0,5301	0,0024	0
CRIPAK	6282	871,774	0,5322	0,0024	0
HPS3	12241	880,758	0,5346	0,0024	0
GBP4	10613	882,6905	0,5508	0,0024	0
YPEL3	47733	907,814	0,5061	0,0025	0
SSH2	44108	907,3672	0,5074	0,0025	0
BCL11A	2244	905,2398	0,5144	0,0025	0
CD19	4911	905,1079	0,5172	0,0025	0
NBPF11	36241	904,9074	0,5246	0,0025	0
DFFB	7219	903,9099	0,5354	0,0025	0
MTMR4	35877	905,5851	0,5367	0,0025	0
RTP4	41798	892,0024	0,5408	0,0025	0
TNIP1	45782	914,3877	0,542	0,0025	0
LRIG1	34148	914,9851	0,5131	0,0026	0
FCRLA	9346	908,4717	0,5306	0,0026	0
POLR3GL	39393	917,0905	0,5327	0,0026	0
IRF9	25699	902,3406	0,5415	0,0026	0
MPPE1	35558	948,5522	0,5164	0,0027	0
STX7	44381	945,407	0,5231	0,0027	0
SHISA5	42649	929,0579	0,5248	0,0027	0
TRIM22	46070	930,3252	0,5267	0,0027	0
ZFP106	47928	930,3152	0,5275	0,0027	0
LOC651309	32358	931,4736	0,5278	0,0027	0
SIN3A	42711	930,4172	0,5295	0,0027	0
CYFIP2	6752	949,9597	0,5299	0,0027	0

C14ORF139	2926	949,499	0,5322	0,0027	0
ARL2	1519	934,9915	0,5349	0,0027	0
LBA1	27023	935,7255	0,5363	0,0027	0
CASP2	4457	935,1394	0,5375	0,0027	0
SAMD9	41933	950,1984	0,5427	0,0027	0
FOXK1	10142	953,5872	0,5424	0,0028	0
ZMAT3	48001	957,8607	0,5435	0,0029	0
HS.556082	20461	970,4435	0,5399	0,003	0
LOC374395	27945	972,0608	0,5437	0,003	0
RHOT1	41198	980,6813	0,5413	0,0031	0
FCRL2	9337	975,4914	0,5456	0,0031	0
STARD7	44247	984,1898	0,5528	0,0033	0
KIAA1370	26344	999,089	0,5016	0,0035	0
DGKA	7241	1012,6092	0,5209	0,0035	0
ARHGEF18	1473	990,3034	0,5258	0,0035	0
GRINA	11380	1010,0032	0,5278	0,0035	0
HDAC1	11730	1003,0677	0,5302	0,0035	0
TMEM109	45414	999,6534	0,5353	0,0035	0
ADD1	511	1011,3805	0,539	0,0035	0
PSD4	40036	1012,637	0,5403	0,0035	0
ACSS1	335	996,892	0,5464	0,0035	0
HS.354359	15116	1015,3184	0,5473	0,0035	0
SMARCAL1	43381	989,5276	0,5478	0,0035	0
IL10RB	25362	1010,1712	0,5489	0,0035	0
ZNF791	48686	999,0326	0,5492	0,0035	0
SKP2	42762	991,9458	0,5516	0,0035	0
DGKA	7239	1019,5249	0,5194	0,0036	0
GRINA	11379	1020,957	0,5316	0,0036	0
UROS	46969	1021,6152	0,5425	0,0036	0
MRPL34	35646	1025,1221	0,5463	0,0036	0
NKTR	36620	1034,5564	0,5468	0,0036	0
SLC15A4	42819	1025,2615	0,5518	0,0036	0
C6ORF192	3932	1033,9456	0,5195	0,0037	0
LPAR5	34107	1035,7125	0,5253	0,0037	0
ULK1	46870	1047,8488	0,5297	0,0037	0
IL10RB	25363	1031,6647	0,5327	0,0037	0
EHD1	8146	1032,7977	0,5334	0,0037	0
BIN1	2358	1036,6794	0,5352	0,0037	0
CXXC1	6717	1040,9914	0,5407	0,0037	0
NUCB1	37067	1031,3366	0,5407	0,0037	0
HLA-DOB	12011	1036,9194	0,5438	0,0037	0
SP110	43756	1027,8906	0,5467	0,0037	0
NIF3L1	36580	1038,2551	0,5496	0,0037	0
TMEM101	45401	1034,0005	0,5573	0,0037	0
GRN	11420	1056,6357	0,5379	0,0038	0
RBBP9	40786	1052,9116	0,5523	0,0038	0
TPRG1L	45943	1051,8704	0,5614	0,0038	0

GTF2IP1	11520	1059,6027	0,558	0,0039	0
TCF3	44924	1064,178	0,5481	0,004	0
BCKDHA	2235	1072,1538	0,5453	0,0041	0
ZFP36L2	47938	1071,783	0,5465	0,0041	0
ALOX5	904	1069,4327	0,5473	0,0041	0
HS.91389	24831	1082,0606	0,5538	0,0042	0
TMEM79	45629	1081,417	0,558	0,0042	0
IL7	25507	1089,2178	0,5632	0,0043	0
MCM5	34902	1097,759	0,5441	0,0044	0
C5ORF39	3853	1096,9688	0,5466	0,0044	0
SLC12A9	42804	1093,443	0,5551	0,0044	0
SLC16A5	42834	1095,2528	0,5675	0,0044	0
FCRL3	9340	1090,3293	0,5974	0,0044	0
COASY	5946	1102,5926	0,5561	0,0045	0
PRIM1	39800	1094,5203	0,5662	0,0045	0
ST6GALNAC6	44185	1107,0659	0,5434	0,0046	0
P2RY10	37945	1107,8263	0,5597	0,0046	0
UGP2	46824	1117,0852	0,5601	0,0049	0
NDE1	36328	1119,189	0,5535	0,005	0
TAPBP	44703	1118,0489	0,5604	0,005	0
HS.18081	13815	1116,9666	0,5612	0,005	0
ALS2CR13	922	1123,272	0,5114	0,0051	0
GALNAC4S-6ST	10505	1122,799	0,534	0,0051	0
NPHP3	36842	1134,8145	0,5434	0,0051	0
ABLIM1	190	1122,2652	0,5472	0,0051	0
MAPK8IP3	34716	1131,9844	0,5531	0,0051	0
C17ORF59	3117	1125,5636	0,5746	0,0051	0
DGCR2	7231	1140,5813	0,5497	0,0052	0
NEDD8	36408	1137,9101	0,5645	0,0052	0
FAM39DP	8943	1151,0774	0,5465	0,0054	0
TNFRSF13B	45737	1150,4542	0,6737	0,0054	0
ETV6	8587	1158,4224	0,5601	0,0055	0
CHRNB1	5557	1157,5071	0,5674	0,0055	0
PIK3R2	38908	1161,0575	0,579	0,0055	0
SCRN1	42100	1167,3999	0,5505	0,0056	0
CDKN1B	5199	1174,2705	0,5212	0,0057	0
SHPK	42662	1174,0526	0,5608	0,0057	0
POLM	39348	1174,3237	0,5657	0,0057	0
ZFP161	47931	1186,9767	0,5548	0,0058	0
KIAA0922	26279	1183,204	0,5577	0,0058	0
DGCR6	7233	1186,3563	0,5584	0,0058	0
EIF2AK1	8173	1180,9981	0,5774	0,0058	0
VASH1	47124	1192,9116	0,6689	0,0059	0
HNRPUL1	12145	1192,4172	0,5472	0,006	0
ANKRA2	1055	1191,5665	0,5541	0,006	0
XAF1	47619	1194,5063	0,5657	0,006	0
BNIP3L	2440	1202,3107	0,5435	0,0061	0

UNC84B	46906	1204,1056	0,5527	0,0061	0
PYHIN1	40412	1199,5139	0,555	0,0061	0
C7ORF27	4002	1213,1462	0,5578	0,0061	0
GNG7	10997	1213,8507	0,5679	0,0061	0
SMARCC2	43386	1211,676	0,5778	0,0061	0
INPP5D	25597	1206,4998	0,5416	0,0062	0
BBS2	2189	1211,2574	0,5492	0,0062	0
C20ORF72	3552	1216,3227	0,5615	0,0062	0
RNF144B	41335	1209,7693	0,5616	0,0062	0
CTSH	6596	1210,7147	0,5634	0,0062	0
SNX3	43639	1219,9941	0,5732	0,0062	0
IL16	25385	1221,095	0,5357	0,0063	0
LAPTM5	26982	1228,8989	0,5405	0,0065	0
PVRIG	40364	1229,6153	0,548	0,0065	0
ARL17P1	1518	1228,8857	0,5556	0,0065	0
GOLPH3L	11054	1231,2109	0,5636	0,0065	0
BTN3A2	2600	1228,6563	0,5798	0,0065	0
WDR54	47439	1238,6469	0,5621	0,0066	0
IDH1	25180	1248,6903	0,5666	0,0067	0
TNFSF12	45764	1248,3538	0,5673	0,0067	0
LOC388969	28091	1251,0602	0,5735	0,0067	0
TGFBI	45117	1256,0533	0,5484	0,0068	0
C20ORF117	3481	1252,9377	0,5656	0,0068	0
VPS45	47273	1251,8915	0,5799	0,0068	0
BCL11A	2243	1258,7846	0,5472	0,0069	0
PPM1F	39556	1255,3611	0,5645	0,0069	0
ITGAE	25758	1255,6806	0,5691	0,0069	0
B3GALT4	2062	1260,719	0,5743	0,0069	1,00E-04
CCDC28A	4649	1271,0707	0,572	0,007	1,00E-04
FLJ12949	9588	1269,7611	0,575	0,007	1,00E-04
STAT1	44253	1275,4089	0,5571	0,0071	1,00E-04
PITPNM1	38957	1274,4435	0,5598	0,0071	1,00E-04
PLCG2	39093	1278,9605	0,5362	0,0072	1,00E-04
ALDH9A1	851	1281,4423	0,5742	0,0072	1,00E-04
IL16	25384	1277,2418	0,5799	0,0072	1,00E-04
RNF135	41324	1285,1961	0,5599	0,0074	1,00E-04
ALKBH5	885	1285,3073	0,5648	0,0074	1,00E-04
MEN1	35028	1289,4858	0,5733	0,0075	1,00E-04
HS.436134	15728	1293,0661	0,5697	0,0077	1,00E-04
SDHA	42150	1300,0628	0,573	0,0078	1,00E-04
SLFN11	43319	1298,5122	0,581	0,0078	1,00E-04
USP24	47014	1301,574	0,5597	0,0079	1,00E-04
PLSCR3	39202	1308,6625	0,5609	0,0079	1,00E-04
ZNF828	48704	1310,6205	0,5632	0,0079	1,00E-04
LYN	34387	1308,63	0,5569	0,008	1,00E-04
EDG6	8040	1306,1898	0,5584	0,008	1,00E-04
LTA4H	34324	1307,9911	0,5621	0,008	1,00E-04

ZNF133	48048	1306,0074	0,5723	0,008	1,00E-04
RCSD1	40933	1306,5224	0,5749	0,008	1,00E-04
MAP4K2	34666	1314,9901	0,5635	0,0081	1,00E-04
ZC3H7A	47843	1321,8818	0,5719	0,0081	1,00E-04
BRD3	2490	1316,9388	0,577	0,0081	1,00E-04
RHOT1	41199	1323,4022	0,5769	0,0082	1,00E-04
FRAT2	10199	1329,8121	0,5798	0,0082	1,00E-04
TRAK1	45997	1323,1776	0,5864	0,0082	1,00E-04
PLEKHG1	39144	1334,88	0,5636	0,0084	1,00E-04
TDRD7	44993	1336,075	0,5943	0,0084	1,00E-04
ARRDC2	1613	1340,6923	0,5648	0,0085	1,00E-04
NAPSA	36184	1343,6166	0,5665	0,0085	1,00E-04
CORO1B	6103	1339,1548	0,5695	0,0085	1,00E-04
RAB11FIP1	40451	1341,3597	0,5752	0,0085	1,00E-04
HHEX	11855	1339,8969	0,5785	0,0085	1,00E-04
RCOR3	40929	1367,4327	0,5626	0,0089	1,00E-04
C5ORF25	3837	1355,767	0,5665	0,0089	1,00E-04
BRWD2	2542	1354,9481	0,568	0,0089	1,00E-04
CYSLTR1	6860	1363,7173	0,5701	0,0089	1,00E-04
HSD17B4	24962	1361,7408	0,5785	0,0089	1,00E-04
MKRN1	35361	1357,8325	0,5827	0,0089	1,00E-04
PHF21A	38782	1352,4267	0,5834	0,0089	1,00E-04
USP21	47008	1360,4622	0,5838	0,0089	1,00E-04
AHNAK	687	1354,4622	0,5853	0,0089	1,00E-04
ISCU	25718	1360,614	0,587	0,0089	1,00E-04
WDR8	47473	1371,3172	0,5861	0,0091	1,00E-04
KLHDC2	26603	1378,5875	0,5732	0,0094	1,00E-04
RBL2	40794	1383,4684	0,5836	0,0094	1,00E-04
ABR	198	1395,0623	0,559	0,0095	1,00E-04
RAB5B	40530	1393,8747	0,5792	0,0095	1,00E-04
RASSF2	40752	1387,1379	0,5863	0,0095	1,00E-04
VIM	47196	1398,9858	0,5915	0,0095	1,00E-04
ZMYND8	48029	1391,4887	0,5995	0,0095	1,00E-04
AIFM1	700	1403,3416	0,6029	0,0095	1,00E-04
PPM1K	39562	1402,6834	0,5675	0,0096	1,00E-04
PASK	38153	1397,999	0,568	0,0096	1,00E-04
KIAA0355	26204	1399,8897	0,57	0,0096	1,00E-04
SPHK2	43901	1401,7346	0,5776	0,0096	1,00E-04
DPYSL2	7755	1399,9302	0,5806	0,0096	1,00E-04
MAP4K1	34664	1405,0496	0,5849	0,0096	1,00E-04
BRD8	2498	1404,4269	0,5956	0,0096	1,00E-04
TBC1D10C	44762	1407,508	0,5634	0,0097	1,00E-04
DUSP18	7874	1410,9692	0,576	0,0098	1,00E-04
APAF1	1252	1414,9467	0,5676	0,0099	1,00E-04
C11ORF17	2751	1415,032	0,5745	0,0099	1,00E-04
AKNA	770	1412,9357	0,588	0,0099	1,00E-04
DYRK2	7947	1417,0173	0,5673	0,01	1,00E-04

HS.561747	20971	1423,3658	0,5682	0,01	1,00E-04
SIGLEC10	42684	1424,6943	0,5713	0,01	1,00E-04
ARL2BP	1520	1422,6487	0,5802	0,01	1,00E-04
C5ORF41	3857	1426,1863	0,5911	0,0101	1,00E-04
COMMD3	6052	1427,9904	0,5916	0,0101	1,00E-04
LOC730256	33881	1434,956	0,5762	0,0102	1,00E-04
HS.569104	22470	1434,2059	0,5785	0,0102	1,00E-04
TMEM50B	45598	1430,5577	0,5795	0,0102	1,00E-04
ZNF75	48651	1431,8474	0,5855	0,0102	1,00E-04
TMEM156	45470	1433,5434	0,5855	0,0102	1,00E-04
SP110	43757	1440,7143	0,5722	0,0103	1,00E-04
MGC12966	35141	1447,9511	0,594	0,0104	1,00E-04
VHL	47190	1447,2923	0,5847	0,0105	1,00E-04
JAK1	25839	1448,3766	0,5857	0,0105	1,00E-04
DEF8	7125	1454,456	0,5744	0,0106	1,00E-04
MAT2B	34794	1456,4924	0,6075	0,0106	1,00E-04
C14ORF106	2897	1457,5676	0,5778	0,0107	1,00E-04
LOC729559	33811	1458,2677	0,5828	0,0107	1,00E-04
RAB7L1	40540	1460,7444	0,5835	0,0107	1,00E-04
ANKRD13D	1070	1460,5203	0,5968	0,0107	1,00E-04
FLJ25778	9698	1460,8067	0,602	0,0107	1,00E-04
XKR8	47660	1464,6811	0,5923	0,0108	1,00E-04
LOC727762	33553	1469,6255	0,5827	0,0109	1,00E-04
ARHGAP1	1407	1478,4212	0,5818	0,011	1,00E-04
BTN3A1	2598	1475,8741	0,5824	0,011	1,00E-04
METT11D1	35056	1475,7898	0,59	0,011	1,00E-04
TUBB	46510	1475,3551	0,5975	0,011	1,00E-04
FCRL2	9338	1483,8633	0,5894	0,0111	1,00E-04
ZNF34	48263	1485,491	0,5957	0,0111	1,00E-04
SIDT1	42678	1487,141	0,5704	0,0112	1,00E-04
SLC15A3	42818	1488,0708	0,5867	0,0112	1,00E-04
LOC652495	32728	1487,3431	0,6084	0,0112	1,00E-04
TCTN1	44974	1490,1831	0,6269	0,0112	1,00E-04
LTB	34326	1500,835	0,5645	0,0113	1,00E-04
CXCL16	6661	1500,6866	0,5704	0,0113	1,00E-04
MCCC1	34878	1498,2714	0,578	0,0113	1,00E-04
C17ORF56	3110	1499,7443	0,6027	0,0113	1,00E-04
FLJ20444	9633	1494,3509	0,6034	0,0113	1,00E-04
HS.532698	16864	1507,0371	0,5814	0,0115	1,00E-04
DUS2L	7854	1504,3808	0,5895	0,0115	1,00E-04
SLC22A15	42875	1507,625	0,5995	0,0115	1,00E-04
CASP2	4458	1505,0164	0,6015	0,0115	1,00E-04
PCMTD2	38368	1518,2577	0,5796	0,0117	1,00E-04
PTPRO	40317	1512,4407	0,5823	0,0117	1,00E-04
MED16	34967	1514,5773	0,5833	0,0117	1,00E-04
CASP1	4449	1512,4929	0,6006	0,0117	1,00E-04
RPUSD1	41697	1510,8413	0,6021	0,0117	1,00E-04

KIAA0040	26163	1509,4	0,6029	0,0117	1,00E-04
LOC652694	32844	1510,1405	0,8011	0,0117	1,00E-04
FAM53B	8982	1519,2834	0,5726	0,0118	1,00E-04
LIPA	27275	1519,7639	0,592	0,0118	1,00E-04
GPT2	11298	1518,0399	0,6082	0,0118	1,00E-04
C21ORF33	3581	1525,3416	0,5811	0,0119	1,00E-04
FAM116A	8794	1522,4858	0,5827	0,0119	1,00E-04
LOC338799	27830	1523,8595	0,595	0,0119	1,00E-04
CDCA7L	5118	1535,3056	0,5938	0,0122	1,00E-04
SLC16A4	42832	1533,8128	0,5986	0,0122	1,00E-04
CBFA2T2	4513	1531,3905	0,5994	0,0122	1,00E-04
CEP192	5342	1533,0219	0,603	0,0122	1,00E-04
SMARCB1	43382	1541,4745	0,5788	0,0123	1,00E-04
FAM62A	9000	1542,2547	0,5849	0,0123	1,00E-04
LACTB	26926	1545,0954	0,5923	0,0123	1,00E-04
IRF5	25692	1539,4679	0,5933	0,0123	1,00E-04
CRYZL1	6354	1537,0847	0,6003	0,0123	1,00E-04
DDX23	7069	1541,397	0,6108	0,0123	1,00E-04
SYNE2	44531	1540,7272	0,6352	0,0123	1,00E-04
ERP29	8533	1548,2649	0,6045	0,0124	1,00E-04
ANKRD13A	1063	1551,8051	0,5832	0,0125	1,00E-04
STK36	44317	1552,8169	0,6009	0,0125	1,00E-04
ST3GAL5	44165	1564,4957	0,578	0,0128	1,00E-04
LOC650826	32229	1565,5547	0,617	0,0128	1,00E-04
TIFA	45239	1571,5583	0,5863	0,0129	1,00E-04
IFIT2	25213	1568,8276	0,5873	0,0129	1,00E-04
AP1M1	1219	1564,3529	0,6015	0,0129	1,00E-04
RNASEL	41289	1572,2437	0,6025	0,0129	1,00E-04
FOXO3	10162	1567,1326	0,6042	0,0129	1,00E-04
USP21	47006	1572,9887	0,6095	0,0129	1,00E-04
ARID4A	1497	1571,121	0,6102	0,0129	1,00E-04
HERC5	11809	1589,3921	0,5857	0,0133	1,00E-04
LSP1	34309	1588,3188	0,5868	0,0133	1,00E-04
KIAA1370	26343	1601,0803	0,5745	0,0134	1,00E-04
ZFP90	47946	1598,5067	0,5894	0,0134	1,00E-04
GNS	11024	1601,544	0,5995	0,0134	1,00E-04
CDAN1	5045	1595,4838	0,6001	0,0134	1,00E-04
LYK5	34384	1601,543	0,601	0,0134	1,00E-04
HS.406106	15516	1591,7556	0,6116	0,0134	1,00E-04
TAP2	44702	1591,8853	0,6287	0,0134	1,00E-04
CD79A	5008	1608,8821	0,5757	0,0136	1,00E-04
SS18	44091	1608,6186	0,5918	0,0136	1,00E-04
FAM39DP	8941	1613,3401	0,5925	0,0137	1,00E-04
RPS4Y1	41666	1611,7676	0,8896	0,0137	1,00E-04
TMEM154	45468	1616,999	0,5949	0,0138	1,00E-04
C14ORF159	2944	1616,8318	0,6029	0,0138	1,00E-04
SPECC1L	43875	1624,6405	0,5892	0,014	1,00E-04

DCLRE1C	6970	1625,1951	0,618	0,014	1,00E-04
SP110	43754	1629,5191	0,6057	0,0141	1,00E-04
SYT17	44575	1627,6275	0,6199	0,0141	1,00E-04
RPA1	41483	1631,3695	0,5935	0,0143	2,00E-04
MGEA5	35279	1634,2415	0,5956	0,0143	2,00E-04
SDHA	42149	1633,5657	0,6003	0,0143	2,00E-04
PTPRA	40291	1637,3324	0,6057	0,0143	2,00E-04
USF1	46971	1650,7971	0,5927	0,0144	2,00E-04
TSC1	46253	1647,6148	0,594	0,0144	2,00E-04
LAMA5	26952	1653,08	0,5961	0,0144	2,00E-04
ARID1A	1487	1640,157	0,5967	0,0144	2,00E-04
SPAST	43827	1644,124	0,6082	0,0144	2,00E-04
ABLIM1	193	1649,6045	0,6094	0,0144	2,00E-04
MED16	34966	1644,5758	0,6112	0,0144	2,00E-04
DHRS1	7277	1641,3352	0,6167	0,0144	2,00E-04
DEF6	7122	1655,8123	0,5901	0,0145	2,00E-04
LOC730744	33924	1646,2778	0,5968	0,0145	2,00E-04
PAPSS1	38087	1654,1307	0,604	0,0145	2,00E-04
RBM33	40828	1643,1678	0,6091	0,0145	2,00E-04
GALK2	10502	1649,38	0,6101	0,0145	2,00E-04
C6ORF136	3893	1646,931	0,6135	0,0145	2,00E-04
ZNF792	48687	1646,8503	0,6135	0,0145	2,00E-04
ZNF74	48646	1644,0672	0,621	0,0145	2,00E-04
SLC15A4	42820	1653,7695	0,6259	0,0145	2,00E-04
LOC339123	27842	1659,6193	0,5967	0,0147	2,00E-04
MAP3K7IP1	34653	1661,0988	0,6214	0,0147	2,00E-04
ECOP	8014	1662,5028	0,5978	0,0148	2,00E-04
PLA2G4B	39044	1669,9363	0,6061	0,0148	2,00E-04
CBR4	4533	1668,3511	0,6169	0,0148	2,00E-04
DHRS7	7288	1664,4388	0,6202	0,0148	2,00E-04
MIA3	35298	1668,9482	0,6056	0,0149	2,00E-04
GTF2IP1	11521	1667,5876	0,6155	0,0149	2,00E-04
KLF13	26584	1673,8869	0,604	0,015	2,00E-04
RASSF2	40750	1674,1507	0,6095	0,015	2,00E-04
CXXC5	6719	1677,5985	0,5836	0,0151	2,00E-04
SGSM2	42562	1681,2988	0,589	0,0151	2,00E-04
RWDD2A	41838	1678,7193	0,6046	0,0151	2,00E-04
TCL1A	44941	1678,876	0,6048	0,0151	2,00E-04
RPA1	41482	1677,7639	0,6067	0,0151	2,00E-04
SNX1	43610	1678,0641	0,6073	0,0151	2,00E-04
JARID1D	25850	1681,4084	0,7531	0,0151	2,00E-04
UPF3A	46935	1685,6564	0,6199	0,0152	2,00E-04
SLC25A12	42916	1684,9826	0,6207	0,0152	2,00E-04
NBN	36228	1687,7992	0,6254	0,0152	2,00E-04
ATRN	1993	1690,7346	0,6037	0,0153	2,00E-04
TSPAN3	46321	1688,9107	0,6258	0,0153	2,00E-04
TMEM14C	45460	1692,7079	0,6265	0,0153	2,00E-04

TSPO	46340	1687,398	0,7718	0,0153	2,00E-04
HIVEP1	11989	1695,2291	0,5725	0,0154	2,00E-04
SP1	43750	1694,6858	0,6085	0,0154	2,00E-04
ZNF671	48590	1700,241	0,6269	0,0154	2,00E-04
GPX1	11299	1704,4479	0,5919	0,0155	2,00E-04
FLJ20489	9637	1704,8933	0,6097	0,0155	2,00E-04
ATG2A	1789	1703,4682	0,6014	0,0156	2,00E-04
ZFP106	47929	1702,8228	0,6108	0,0156	2,00E-04
OSGEPL1	37847	1706,0767	0,6334	0,0156	2,00E-04
LOC651575	32427	1711,993	0,6117	0,0158	2,00E-04
HS.550293	19920	1724,3736	0,5937	0,0161	2,00E-04
PLAC8	39065	1720,5127	0,6028	0,0161	2,00E-04
TRAPPC2	46006	1724,6336	0,6208	0,0161	2,00E-04
SNRP70	43575	1722,5674	0,6255	0,0161	2,00E-04
C21ORF59	3613	1722,5257	0,6275	0,0161	2,00E-04
CAPN1	4381	1729,2773	0,6189	0,0162	2,00E-04
DPAGT1	7692	1731,4837	0,6213	0,0162	2,00E-04
C11ORF2	2752	1742,1122	0,5982	0,0163	2,00E-04
PYHIN1	40411	1740,2252	0,5993	0,0163	2,00E-04
CYFIP2	6753	1740,0192	0,6089	0,0163	2,00E-04
ITM2B	25810	1736,8986	0,6128	0,0163	2,00E-04
NUP62	37145	1743,3017	0,6132	0,0163	2,00E-04
STARD7	44248	1742,4493	0,625	0,0163	2,00E-04
RNASET2	41292	1745,5375	0,6011	0,0164	2,00E-04
IFNAR2	25244	1742,0326	0,6299	0,0164	2,00E-04
APPL2	1343	1749,5225	0,6034	0,0165	2,00E-04
TIMELESS	45251	1748,9891	0,6109	0,0165	2,00E-04
LOC728554	33680	1760,4043	0,5986	0,0166	2,00E-04
TYK2	46605	1758,8935	0,6008	0,0166	2,00E-04
RHBDD2	41162	1755,9464	0,6043	0,0166	2,00E-04
OSBPL9	37842	1761,6352	0,6159	0,0166	2,00E-04
SYS1	44562	1755,5132	0,6162	0,0166	2,00E-04
KIAA0125	26170	1756,6111	0,6188	0,0166	2,00E-04
CECR5	5278	1760,06	0,6197	0,0166	2,00E-04
TAF1C	44654	1755,8518	0,6209	0,0166	2,00E-04
FBXL15	9185	1759,6244	0,6262	0,0166	2,00E-04
RIPK5	41245	1763,0863	0,6117	0,0167	2,00E-04
OPN3	37319	1764,6168	0,6124	0,0168	2,00E-04
SAMM50	41936	1767,8787	0,621	0,0168	2,00E-04
TRIM25	46077	1765,4796	0,6333	0,0168	2,00E-04
BLR1	2386	1771,2168	0,6183	0,017	2,00E-04
LOC153364	27518	1773,1329	0,6266	0,017	2,00E-04
ETS1	8577	1777,8541	0,5927	0,0172	2,00E-04
LRDD	34138	1777,9344	0,615	0,0172	2,00E-04
ZNF564	48460	1777,1354	0,6217	0,0172	2,00E-04
POLD4	39333	1780,7154	0,6171	0,0173	2,00E-04
SPG7	43897	1789,3942	0,5991	0,0175	2,00E-04

HS.481659	16233	1790,026	0,6212	0,0175	2,00E-04
ZBTB24	47797	1790,3133	0,6238	0,0175	2,00E-04
TMEM154	45467	1792,4178	0,5862	0,0176	2,00E-04
FXYD5	10356	1798,9522	0,5975	0,0177	2,00E-04
LFNG	27149	1799,9412	0,6279	0,0177	2,00E-04
S100PBP	41887	1799,3755	0,632	0,0177	2,00E-04
IKZF1	25351	1805,4976	0,5938	0,0178	2,00E-04
LANCL1	26973	1805,2059	0,617	0,0178	2,00E-04
C22ORF29	3648	1807,3067	0,6336	0,0178	2,00E-04
HS.20255	14067	1804,7124	0,6404	0,0178	2,00E-04
TMCO3	45377	1810,222	0,6042	0,018	2,00E-04
FLJ20718	9646	1817,7525	0,6303	0,0183	2,00E-04
MGC71993	35273	1823,2198	0,6142	0,0184	2,00E-04
ZNF446	48349	1820,0998	0,6193	0,0184	2,00E-04
RFX5	41072	1824,3476	0,6197	0,0184	2,00E-04
GUSBL1	11577	1823,4554	0,6285	0,0184	2,00E-04
SPG11	43886	1820,9352	0,6337	0,0184	2,00E-04
ATP5A1	1889	1824,7906	0,6338	0,0184	2,00E-04
TMEM77	45628	1829,4274	0,6044	0,0185	2,00E-04
DNAJB2	7570	1841,3234	0,6109	0,0187	2,00E-04
ZFP3	47934	1836,4992	0,6165	0,0187	2,00E-04
ZNF573	48475	1840,4549	0,6171	0,0187	2,00E-04
FNBP1	10075	1835,0626	0,6287	0,0187	2,00E-04
GRN	11421	1843,818	0,6047	0,0188	2,00E-04
ADAM19	412	1842,9013	0,6135	0,0188	2,00E-04
C12ORF52	2853	1840,2846	0,6159	0,0188	2,00E-04
CHI3L2	5478	1841,1702	0,6954	0,0188	2,00E-04
AMT	978	1851,0472	0,6117	0,019	2,00E-04
GMPR2	10948	1854,871	0,6166	0,019	2,00E-04
HNRPUL1	12144	1851,5474	0,6204	0,019	2,00E-04
HARS2	11667	1850,5106	0,6381	0,019	2,00E-04
ZMAT5	48005	1848,4556	0,6413	0,019	2,00E-04
HNRPUL2	12147	1863,0294	0,6228	0,0193	3,00E-04
C12ORF10	2816	1870,6268	0,6341	0,0195	3,00E-04
RERE	41004	1873,8697	0,6226	0,0197	3,00E-04
TCEAL8	44889	1878,1485	0,6157	0,0199	3,00E-04
NBPF3	36251	1880,7038	0,6244	0,0199	3,00E-04
CNDP2	5859	1885,4396	0,6201	0,02	3,00E-04
STAT1	44252	1883,6	0,6341	0,02	3,00E-04
LOC153364	27517	1884,4827	0,6455	0,02	3,00E-04
ECHDC2	8006	1888,3601	0,6085	0,0201	3,00E-04
KLHDC3	26604	1889,7509	0,6332	0,0201	3,00E-04
TCFL5	44934	1892,2131	0,6189	0,0202	3,00E-04
ZNF443	48346	1893,4398	0,6198	0,0202	3,00E-04
GIT2	10808	1902,0062	0,6333	0,0205	3,00E-04
ATP2B4	1877	1901,858	0,6398	0,0206	3,00E-04
VIM	47197	1909,3813	0,6245	0,0207	3,00E-04

SLC25A42	42968	1907,9625	0,6281	0,0207	3,00E-04
IFNGR2	25252	1911,6732	0,6287	0,0207	3,00E-04
ATP5A1	1888	1909,5115	0,6372	0,0207	3,00E-04
CIAO1	5584	1904,4846	0,6429	0,0207	3,00E-04
MCEE	34881	1913,5826	0,6421	0,0208	3,00E-04
NDUFS3	36383	1914,9108	0,6436	0,0208	3,00E-04
ZBTB4	47810	1920,6908	0,6108	0,0209	3,00E-04
HS.400256	15470	1917,3327	0,621	0,0209	3,00E-04
HS.194225	13909	1918,1267	0,621	0,0209	3,00E-04
C8ORF55	4066	1918,3959	0,6223	0,0209	3,00E-04
CTPS2	6575	1918,8251	0,6229	0,0209	3,00E-04
ACAD11	213	1924,7266	0,6078	0,021	3,00E-04
PLEKHO1	39165	1922,8093	0,6128	0,021	3,00E-04
RALB	40632	1925,2769	0,6386	0,021	3,00E-04
FAM111A	8787	1927,0384	0,6085	0,0211	3,00E-04
CAPRIN2	4409	1930,6606	0,6261	0,0212	3,00E-04
NLRX1	36675	1929,7074	0,6278	0,0212	3,00E-04
RNF34	41388	1929,4052	0,6308	0,0212	3,00E-04
ARHGEF6	1480	1934,0364	0,6067	0,0214	3,00E-04
NDUFB10	36367	1934,9455	0,639	0,0214	3,00E-04
NSUN5C	37011	1937,4696	0,6559	0,0215	3,00E-04
CDC2L6	5081	1943,0034	0,6129	0,0218	3,00E-04
C4ORF14	3791	1943,4689	0,6425	0,0218	3,00E-04
SYPL1	44559	1948,7407	0,6246	0,0219	3,00E-04
PASK	38152	1951,3609	0,6262	0,0219	3,00E-04
PGM1	38709	1949,4772	0,6318	0,0219	3,00E-04
MKKS	35352	1948,3531	0,6343	0,0219	3,00E-04
PRKAB2	39811	1955,3998	0,6341	0,022	3,00E-04
ANKRA2	1056	1959,0505	0,6254	0,0221	3,00E-04
ADD3	519	1957,7155	0,6258	0,0221	3,00E-04
RHBDD2	41161	1962,0447	0,6177	0,0222	3,00E-04
CTNNBIP1	6559	1962,8258	0,6193	0,0222	3,00E-04
WNT3	47567	1960,1072	0,7238	0,0222	3,00E-04
DNASE1L1	7615	1966,8133	0,6165	0,0224	3,00E-04
HLTF	12039	1970,3512	0,6514	0,0226	3,00E-04
ATP2A3	1866	1976,1543	0,6047	0,0228	3,00E-04
THYN1	45221	1976,7107	0,6549	0,0228	3,00E-04
TRPT1	46228	1978,0597	0,6179	0,0229	3,00E-04
MED20	34971	1981,7128	0,6198	0,023	3,00E-04
DGCR6	7235	1980,4732	0,6306	0,023	3,00E-04
ZNF177	48088	1981,4531	0,6323	0,023	3,00E-04
SCARB2	42015	1983,6441	0,6187	0,0231	3,00E-04
TMEM134	45443	1984,4762	0,6273	0,0231	3,00E-04
MFSD3	35104	1982,9829	0,6377	0,0231	3,00E-04
FRAT1	10197	1983,1447	0,6392	0,0231	3,00E-04
HS.572649	22943	1993,0703	0,6297	0,0233	3,00E-04
RNF146	41337	1990,5404	0,6322	0,0233	3,00E-04

CEACAM1	5256	1999,4538	0,6348	0,0234	3,00E-04
LOC647000	31119	1999,1043	0,638	0,0234	3,00E-04
PDHB	38512	1994,6938	0,6438	0,0234	3,00E-04
LOC153561	27520	1997,7723	0,6199	0,0235	3,00E-04
NIPSNAP1	36596	1998,6533	0,6304	0,0235	3,00E-04
PDE7A	38480	2006,9232	0,6374	0,0236	3,00E-04
INPPL1	25602	2009,2632	0,6157	0,0237	3,00E-04
MBD4	34832	2009,2868	0,6385	0,0237	3,00E-04
PARP3	38132	2015,6565	0,6216	0,0238	3,00E-04
FAM156A	8884	2010,9515	0,6338	0,0238	3,00E-04
RAI1	40627	2013,9442	0,6396	0,0238	3,00E-04
HDDC3	11749	2012,4234	0,6545	0,0238	3,00E-04
PARP10	38121	2023,8346	0,6306	0,024	4,00E-04
ITGB7	25791	2025,1888	0,6307	0,0241	4,00E-04
PSME1	40134	2021,8162	0,6364	0,0241	4,00E-04
IDH1	25179	2023,4172	0,6469	0,0241	4,00E-04
GNB5	10984	2033,5744	0,6318	0,0243	4,00E-04
BAD	2113	2029,8811	0,6414	0,0243	4,00E-04
CEP68	5354	2033,0153	0,6381	0,0244	4,00E-04
AP2S1	1235	2037,2143	0,6418	0,0245	4,00E-04
RPAIN	41488	2035,7752	0,6436	0,0245	4,00E-04
MLKL	35379	2044,1807	0,6188	0,0249	4,00E-04
ADA	393	2048,0897	0,619	0,025	4,00E-04
ZC3H3	47838	2046,6148	0,6202	0,025	4,00E-04
RNF38	41393	2047,1026	0,6206	0,025	4,00E-04
RUNDC1	41810	2050,7116	0,6227	0,025	4,00E-04
OSTF1	37859	2051,85	0,6296	0,025	4,00E-04
SAP130	41939	2047,6391	0,6352	0,025	4,00E-04
ZC3HAV1	47848	2049,8944	0,6367	0,025	4,00E-04
VAMP8	47112	2045,4886	0,6462	0,025	4,00E-04
SH2B1	42567	2053,3222	0,6447	0,0251	4,00E-04
CXCR5	6674	2057,7845	0,6365	0,0254	4,00E-04
GUSBL1	11578	2060,5907	0,622	0,0255	4,00E-04
BIN1	2357	2065,098	0,6278	0,0256	4,00E-04
C10ORF6	2705	2069,035	0,6292	0,0256	4,00E-04
PAOX	38073	2067,487	0,6419	0,0256	4,00E-04
SMARCC2	43387	2062,3142	0,6444	0,0256	4,00E-04
HS.371609	15250	2065,5168	0,6465	0,0256	4,00E-04
PRMT2	39885	2069,8902	0,6523	0,0256	4,00E-04
GVIN1	11581	2068,3554	0,6126	0,0257	4,00E-04
MAPK3	34701	2071,3009	0,633	0,0257	4,00E-04
C4ORF34	3814	2070,6479	0,6415	0,0257	4,00E-04
HS1BP3	24928	2068,9825	0,6508	0,0257	4,00E-04
LOC653853	33394	2074,2402	0,6576	0,0258	4,00E-04
GLB1	10849	2078,057	0,6131	0,0259	4,00E-04
COG4	5960	2079,4566	0,6464	0,0259	4,00E-04
RNASEL	41288	2078,7148	0,6474	0,0259	4,00E-04

DPY30	7752	2079,4568	0,6498	0,0259	4,00E-04
FAM120A	8803	2077,2102	0,6537	0,0259	4,00E-04
PRKCB1	39836	2081,7999	0,6452	0,026	4,00E-04
LOC652616	32793	2082,0222	0,6536	0,026	4,00E-04
C14ORF93	2995	2088,5786	0,6324	0,0261	4,00E-04
DAPP1	6902	2088,3658	0,645	0,0261	4,00E-04
PHF12	38757	2084,5857	0,6502	0,0261	4,00E-04
HS.254477	14472	2093,5629	0,6515	0,0263	4,00E-04
UNC93B1	46908	2095,2035	0,6452	0,0264	4,00E-04
PPP1R3D	39605	2099,1575	0,6482	0,0265	4,00E-04
CCDC34	4658	2100,1667	0,6551	0,0265	4,00E-04
ADAR	484	2108,4764	0,6377	0,0266	4,00E-04
HS.163426	13665	2104,9903	0,6426	0,0266	4,00E-04
CRYZL1	6355	2104,7465	0,6483	0,0266	4,00E-04
TRIM38	46105	2111,7311	0,6514	0,0266	4,00E-04
HS.561844	20991	2111,5528	0,6523	0,0266	4,00E-04
DBT	6938	2109,8397	0,6527	0,0266	4,00E-04
PIM1	38922	2111,3505	0,6189	0,0267	4,00E-04
FBXO32	9244	2107,1179	0,6284	0,0267	4,00E-04
ZNF362	48278	2108,1965	0,6318	0,0267	4,00E-04
DYRK2	7949	2115,1644	0,6333	0,0267	4,00E-04
VAV1	47130	2108,3139	0,638	0,0267	4,00E-04
RANBP10	40651	2108,0384	0,6535	0,0267	4,00E-04
GOLGA3	11029	2116,5914	0,6466	0,0268	4,00E-04
ZNF248	48159	2118,6728	0,6446	0,0269	4,00E-04
NDUFS8	36388	2127,5679	0,6337	0,0273	4,00E-04
RBM4B	40850	2126,3731	0,6347	0,0273	4,00E-04
ZMIZ1	48006	2135,5802	0,6508	0,0277	4,00E-04
MUM1	35940	2134,9615	0,6305	0,0278	4,00E-04
CRYGS	6347	2141,7798	0,6462	0,028	4,00E-04
MYH9	36027	2141,6884	0,6279	0,0281	4,00E-04
C4ORF41	3823	2145,6992	0,6524	0,0281	5,00E-04
RNASE6	41280	2140,9813	0,6591	0,0281	4,00E-04
FCRL3	9341	2145,7659	0,7268	0,0281	5,00E-04
BTN3A3	2601	2145,4756	0,6283	0,0282	5,00E-04
MAGED1	34528	2156,5048	0,6341	0,0287	5,00E-04
TPD52	45902	2156,6926	0,6352	0,0287	5,00E-04
CTCF	6517	2157,7913	0,6412	0,0287	5,00E-04
GMPR2	10946	2163,5448	0,6589	0,029	5,00E-04
IFITM1	25218	2163,392	0,7894	0,0291	5,00E-04
TYSND1	46612	2170,506	0,633	0,0295	5,00E-04
TOP3B	45865	2173,1426	0,6288	0,0296	5,00E-04
WDR35	47400	2172,6378	0,6486	0,0296	5,00E-04
HPS4	12244	2173,5915	0,6911	0,0296	5,00E-04
AKR1B1	774	2180,1247	0,7077	0,0298	5,00E-04
ZNF831	48710	2188,2126	0,6212	0,0303	5,00E-04
CD6	4994	2187,727	0,715	0,0303	5,00E-04

DCPS	6979	2192,9496	0,639	0,0306	5,00E-04
TMEM131	45436	2193,5284	0,6525	0,0306	5,00E-04
DMAP1	7493	2203,1194	0,6345	0,031	5,00E-04
BBS1	2185	2199,6235	0,6376	0,031	5,00E-04
ZCCHC11	47854	2204,0521	0,6378	0,031	5,00E-04
SLC35E3	43092	2201,9381	0,6391	0,031	5,00E-04
C1ORF38	3378	2198,3778	0,6638	0,031	5,00E-04
ZNF33B	48262	2206,4498	0,6403	0,0311	5,00E-04
FAM111A	8786	2208,6718	0,6292	0,0312	5,00E-04
HLA-DMB	12009	2211,3409	0,6332	0,0313	5,00E-04
FAM39DP	8940	2213,0737	0,6367	0,0313	5,00E-04
NPAL3	36813	2212,5311	0,6496	0,0313	5,00E-04
LYSMD2	34414	2216,5117	0,6441	0,0314	5,00E-04
LOC730994	33941	2222,4332	0,6232	0,0316	5,00E-04
ST3GAL1	44159	2221,5981	0,6368	0,0316	5,00E-04
C16ORF48	3051	2222,3605	0,6442	0,0316	5,00E-04
ZNF827	48703	2219,1626	0,6466	0,0316	5,00E-04
BRCC3	2487	2218,8674	0,6625	0,0316	5,00E-04
SPI1	43904	2225,3608	0,6374	0,0317	5,00E-04
PARP12	38125	2224,3716	0,6469	0,0317	5,00E-04
LOC124446	27372	2225,4601	0,6501	0,0317	5,00E-04
TMEM14C	45461	2227,4335	0,6642	0,0317	5,00E-04
HSD17B11	24954	2224,2263	0,6438	0,0318	5,00E-04
ATXN2	2003	2230,9296	0,6462	0,0318	5,00E-04
ARSD	1629	2229,2111	0,6867	0,0318	5,00E-04
SETDB1	42441	2234,924	0,6598	0,032	5,00E-04
XRCC1	47680	2237,666	0,6519	0,0321	5,00E-04
COL4A3	6008	2234,8603	0,6835	0,0321	5,00E-04
ICK	25165	2241,5166	0,6464	0,0323	5,00E-04
TRRAP	46245	2243,8539	0,6489	0,0323	5,00E-04
HS.572538	22933	2243,0281	0,6505	0,0323	5,00E-04
HS.573047	22985	2242,3362	0,6604	0,0323	5,00E-04
C1ORF83	3410	2246,3021	0,6678	0,0324	6,00E-04
IFNAR1	25243	2251,8673	0,6427	0,0328	6,00E-04
HDGF2	11752	2256,2111	0,6448	0,033	6,00E-04
WRB	47582	2260,1704	0,6493	0,0331	6,00E-04
HS.483906	16242	2262,5654	0,664	0,0332	6,00E-04
RPRC1	41613	2264,4768	0,6465	0,0333	6,00E-04
SETBP1	42428	2269,7532	0,6657	0,0335	6,00E-04
GPS1	11288	2271,4789	0,6502	0,0336	6,00E-04
ST6GALNAC4	44182	2274,5398	0,6638	0,0338	6,00E-04
SEMA4A	42265	2277,5395	0,6427	0,0339	6,00E-04
SDCCAG3	42141	2277,4829	0,6516	0,0339	6,00E-04
SGCE	42535	2276,8916	0,6742	0,0339	6,00E-04
S100A4	41874	2282,8254	0,7295	0,0341	6,00E-04
EEF2K	8073	2286,0034	0,6501	0,0342	6,00E-04
CD27	4935	2292,4004	0,6381	0,0345	6,00E-04

ENTPD6	8377	2293,2371	0,6382	0,0345	6,00E-04
SMARCA2	43376	2292,1908	0,6421	0,0345	6,00E-04
HSDL1	24970	2290,9713	0,6585	0,0345	6,00E-04
TMEM156	45471	2297,5801	0,6304	0,0347	6,00E-04
FAM125A	8815	2296,9086	0,6385	0,0347	6,00E-04
CSNK1G2	6427	2299,2062	0,6427	0,0347	6,00E-04
TMEM140	45450	2300,7929	0,6463	0,0347	6,00E-04
TBC1D2B	44786	2303,9133	0,6589	0,035	6,00E-04
LCOR	27076	2306,036	0,661	0,035	6,00E-04
TACC3	44621	2310,6117	0,6314	0,0351	6,00E-04
LOC646817	31056	2310,3608	0,6405	0,0351	6,00E-04
ZNF589	48499	2308,773	0,6476	0,0351	6,00E-04
AFG3L1	605	2308,5137	0,6538	0,0351	6,00E-04
EVL	8601	2312,9414	0,643	0,0352	6,00E-04
PIK3CG	38901	2314,3434	0,6567	0,0353	6,00E-04
SGSH	42559	2315,8219	0,6492	0,0354	6,00E-04
NME3	36691	2316,4803	0,6569	0,0354	6,00E-04
NOD1	36729	2319,3258	0,6564	0,0355	6,00E-04
TMEM62	45610	2322,7933	0,6469	0,0356	6,00E-04
ZMIZ2	48008	2323,5426	0,6561	0,0356	6,00E-04
KIAA1267	26328	2323,2002	0,6747	0,0356	6,00E-04
C16ORF33	3042	2329,1827	0,6279	0,0358	6,00E-04
PNPLA6	39297	2328,8792	0,6435	0,0358	6,00E-04
GSTM2	11472	2329,9484	0,7952	0,0358	6,00E-04
C14ORF131	2918	2333,2887	0,6595	0,036	6,00E-04
APOBEC3G	1308	2334,9249	0,6615	0,036	6,00E-04
ARID3B	1493	2334,8533	0,6438	0,0361	6,00E-04
TMEM59	45607	2336,7612	0,6586	0,0361	6,00E-04
GLB1	10847	2340,3665	0,63	0,0363	6,00E-04
LOC727820	33566	2344,1837	0,6581	0,0363	7,00E-04
HVCN1	25101	2342,6153	0,6651	0,0363	7,00E-04
MZF1	36114	2346,3123	0,6611	0,0364	7,00E-04
GTPBP3	11545	2345,4564	0,6713	0,0364	7,00E-04
SYT11	44565	2349,3799	0,6505	0,0365	7,00E-04
QARS	40417	2348,7029	0,6543	0,0365	7,00E-04
NAGPA	36148	2352,3649	0,6444	0,0367	7,00E-04
HS.444683	15971	2355,0929	0,6677	0,0368	7,00E-04
ZIK1	47988	2360,783	0,675	0,0372	7,00E-04
LOC116236	27353	2365,5182	0,6475	0,0375	7,00E-04
PI4KB	38841	2368,0503	0,6615	0,0375	7,00E-04
PGBD2	38686	2367,5706	0,6647	0,0375	7,00E-04
PHF14	38762	2366,8269	0,6689	0,0375	7,00E-04
DAPP1	6903	2370,4604	0,6612	0,0376	7,00E-04
MLL	35381	2376,0255	0,648	0,038	7,00E-04
EXDL2	8610	2375,8997	0,6705	0,038	7,00E-04
NDUFA3	36356	2377,8297	0,6694	0,0381	7,00E-04
SIPA1	42715	2382,2893	0,651	0,0383	7,00E-04

FIG4	9486	2383,6021	0,6555	0,0384	7,00E-04
PPFIBP2	39510	2384,9079	0,705	0,0384	7,00E-04
KIAA1545	26372	2387,7258	0,6532	0,0385	7,00E-04
SUSD3	44471	2392,7337	0,6343	0,0387	7,00E-04
ACAA1	206	2393,0077	0,6694	0,0387	7,00E-04
PYGO2	40409	2392,4684	0,6715	0,0387	7,00E-04
CRLF3	6300	2391,6596	0,6764	0,0387	7,00E-04
SYPL1	44557	2403,6746	0,6358	0,039	7,00E-04
ADRB2	573	2399,587	0,6522	0,039	7,00E-04
CECR1	5274	2401,1177	0,6551	0,039	7,00E-04
SBK1	41983	2399,6725	0,6559	0,039	7,00E-04
PHKB	38804	2402,468	0,6624	0,039	7,00E-04
KIAA1683	26407	2403,8563	0,6692	0,039	7,00E-04
SPCS1	43863	2406,7763	0,6702	0,0391	7,00E-04
TMPO	45674	2408,3401	0,6567	0,0392	7,00E-04
HS.46506	16139	2413,9103	0,6493	0,0394	7,00E-04
HERPUD1	11812	2412,6269	0,6639	0,0394	7,00E-04
GPR132	11161	2417,2777	0,6322	0,0395	7,00E-04
KIAA0256	26189	2417,8783	0,6483	0,0395	7,00E-04
C5ORF41	3858	2414,5082	0,6489	0,0395	7,00E-04
TRIM4	46109	2416,3356	0,6564	0,0396	7,00E-04
LASP1	27002	2417,175	0,6754	0,0396	7,00E-04
ATP6V0E2	1938	2423,0047	0,6471	0,0397	7,00E-04
ST8SIA4	44203	2422,3374	0,6509	0,0397	7,00E-04
ZNF395	48299	2425,1514	0,6528	0,0397	7,00E-04
PPP2R4	39649	2420,899	0,6566	0,0397	7,00E-04
PSAP	40017	2422,9448	0,6604	0,0397	7,00E-04
MAPK3	34700	2425,0669	0,6595	0,0398	7,00E-04
IKBKE	25346	2425,922	0,6737	0,0398	8,00E-04
CXCL10	6652	2426,952	0,7843	0,0398	8,00E-04
CAPN12	4388	2432,3819	0,6612	0,04	8,00E-04
MMP11	35431	2433,9186	0,6723	0,0401	8,00E-04
LIME1	27245	2436,5843	0,6553	0,0402	8,00E-04
USP13	46993	2437,8436	0,667	0,0402	8,00E-04
PSMB10	40084	2443,7017	0,6718	0,0402	8,00E-04
KIAA2026	26457	2439,989	0,6514	0,0403	8,00E-04
ALDH3A2	832	2441,8118	0,6558	0,0403	8,00E-04
ACVR1	375	2447,8716	0,6583	0,0403	8,00E-04
HS.173957	13773	2442,004	0,6585	0,0403	8,00E-04
FXYD5	10358	2447,5947	0,6604	0,0403	8,00E-04
C21ORF59	3611	2444,9772	0,6667	0,0403	8,00E-04
ILK	25525	2440,8143	0,6667	0,0403	8,00E-04
HS.122456	12532	2443,0036	0,6781	0,0403	8,00E-04
C2ORF28	3674	2441,3624	0,6829	0,0403	8,00E-04
GBP5	10614	2442,9701	0,8368	0,0403	8,00E-04
PYHIN1	40410	2450,9271	0,6401	0,0404	8,00E-04
ABHD10	152	2447,5813	0,655	0,0404	8,00E-04

KIAA1407	26348	2447,5255	0,6667	0,0404	8,00E-04
PHKB	38801	2455,0192	0,6679	0,0406	8,00E-04
N4BP2L1	36120	2457,3025	0,6559	0,0407	8,00E-04
AES	587	2460,4056	0,6443	0,0408	8,00E-04
CDC25B	5068	2460,3938	0,6532	0,0409	8,00E-04
LRRC37A4	34209	2461,5603	0,6543	0,0409	8,00E-04
SIDT2	42679	2463,9885	0,6576	0,0409	8,00E-04
KIAA0195	26179	2465,8406	0,6586	0,041	8,00E-04
ZNF342	48265	2463,2655	0,6695	0,041	8,00E-04
SNX17	43623	2463,451	0,671	0,041	8,00E-04
PARP2	38129	2470,5822	0,6736	0,0413	8,00E-04
ZFYVE26	47964	2473,2231	0,6498	0,0415	8,00E-04
SUOX	44454	2479,6716	0,6475	0,0417	8,00E-04
HINT2	11895	2476,9641	0,6568	0,0417	8,00E-04
LMO4	27326	2478,4892	0,6519	0,0418	8,00E-04
SESN1	42423	2481,8947	0,6546	0,0418	8,00E-04
RDH11	40939	2479,2031	0,6656	0,0418	8,00E-04
DPF2	7698	2485,3725	0,6586	0,042	8,00E-04
CASD1	4445	2487,8676	0,6444	0,0421	8,00E-04
PRC1	39727	2488,4117	0,6601	0,0421	8,00E-04
MSL3L1	35777	2491,7183	0,6741	0,0422	8,00E-04
PIH1D1	38890	2491,7142	0,6624	0,0423	8,00E-04
STAM	44222	2504,6711	0,6487	0,0424	8,00E-04
UBE2T	46716	2496,3988	0,6646	0,0424	8,00E-04
ITFG2	25739	2504,5067	0,6782	0,0424	8,00E-04
GSTM1	11470	2504,9431	0,7886	0,0424	8,00E-04
COASY	5947	2498,8031	0,6612	0,0425	8,00E-04
PDCD4	38426	2503,8294	0,6669	0,0425	8,00E-04
STXBP3	44387	2498,266	0,6753	0,0425	8,00E-04
CALM3	4325	2503,7646	0,6769	0,0425	8,00E-04
WNT10A	47559	2503,334	0,6566	0,0426	8,00E-04
KIAA0999	26284	2509,0032	0,6642	0,0426	9,00E-04
COL9A3	6037	2509,5761	0,6655	0,0426	9,00E-04
DICER1	7342	2503,7196	0,6675	0,0426	8,00E-04
TMEM149	45457	2503,7208	0,6926	0,0426	8,00E-04
RFTN1	41053	2511,5554	0,825	0,0426	9,00E-04
VPS28	47256	2502,9029	0,6711	0,0427	8,00E-04
CLUAP1	5813	2502,7908	0,6737	0,0427	8,00E-04
PRKD2	39854	2516,1851	0,6713	0,0429	9,00E-04
SNX2	43625	2518,5267	0,6519	0,043	9,00E-04
ZNF341	48264	2517,9087	0,6715	0,043	9,00E-04
PHF3	38786	2529,3642	0,6585	0,0432	9,00E-04
SH3GLB2	42602	2530,7433	0,6585	0,0432	9,00E-04
ALAD	804	2529,1008	0,663	0,0432	9,00E-04
ZSCAN18	48758	2524,9769	0,6466	0,0433	9,00E-04
ACSS2	339	2535,5717	0,6467	0,0433	9,00E-04
HCP5	11724	2528,6464	0,6507	0,0433	9,00E-04

KIAA0907	26277	2535,1643	0,6631	0,0433	9,00E-04
DDX42	7088	2533,8065	0,6631	0,0433	9,00E-04
CMAS	5824	2532,3948	0,6633	0,0433	9,00E-04
CDK5RAP2	5177	2527,5052	0,6654	0,0433	9,00E-04
PPP1CA	39568	2535,0574	0,6666	0,0433	9,00E-04
XYLT2	47698	2532,9695	0,6686	0,0433	9,00E-04
PTPLAD1	40251	2528,7295	0,6708	0,0433	9,00E-04
ZNF2	48110	2538,4685	0,6805	0,0433	9,00E-04
TMEM80	45631	2526,056	0,7158	0,0433	9,00E-04
TRAM2	46002	2538,0585	0,6617	0,0434	9,00E-04
CRTAP	6325	2537,2059	0,6754	0,0434	9,00E-04
LEF1	27113	2539,934	0,7704	0,0434	9,00E-04
SP140	43759	2540,7658	0,6601	0,0435	9,00E-04
CAPN12	4389	2541,0651	0,6627	0,0435	9,00E-04
TUBGCP4	46534	2542,9088	0,6763	0,0435	9,00E-04
TAGLN	44677	2542,7767	0,6733	0,0436	9,00E-04
LOC728014	33600	2545,1695	0,76	0,0436	9,00E-04
LRSAM1	34281	2545,536	0,6547	0,0437	9,00E-04
CXCR3	6670	2550,7279	0,6638	0,0439	9,00E-04
XRCC6BP1	47688	2550,7027	0,6692	0,0439	9,00E-04
RUSC1	41829	2555,4518	0,6624	0,0441	9,00E-04
C3ORF17	3721	2558,1929	0,6651	0,0441	9,00E-04
CTSZ	6607	2557,9245	0,6701	0,0441	9,00E-04
DBNL	6932	2558,9651	0,6897	0,0441	9,00E-04
GSDML	11441	2557,4857	0,664	0,0442	9,00E-04
SOCS1	43662	2560,35	0,6741	0,0442	9,00E-04
EPSTI1	8462	2561,0908	0,6903	0,0442	9,00E-04
FN3KRP	10074	2568,9778	0,6612	0,0443	9,00E-04
RAPGEF1	40680	2569,4905	0,6642	0,0443	9,00E-04
NLRP1	36655	2563,7845	0,6721	0,0443	9,00E-04
PHKA2	38800	2566,6854	0,6559	0,0444	9,00E-04
DKFZP586I1420	7412	2566,316	0,6647	0,0444	9,00E-04
LCMT1	27065	2568,1681	0,6796	0,0444	9,00E-04
RMI1	41258	2564,9356	0,6859	0,0444	9,00E-04
LIN9	27267	2567,7266	0,6865	0,0444	9,00E-04
UBL4A	46741	2575,2226	0,6756	0,0446	9,00E-04
ZSCAN16	48757	2577,1162	0,6633	0,0447	9,00E-04
HS.137078	13105	2580,2662	0,6847	0,0448	9,00E-04
CPEB2	6162	2581,3213	0,6571	0,0449	9,00E-04
CCDC84	4730	2581,6546	0,6761	0,0449	9,00E-04
HS.126768	12656	2583,6773	0,6706	0,045	9,00E-04
IL28RA	25476	2584,0893	0,6706	0,045	9,00E-04
LOC653383	33175	2583,0585	0,673	0,045	9,00E-04
WDSUB1	47490	2585,9215	0,6836	0,0451	0,001
TP53AP1	45881	2587,4341	0,6882	0,0451	0,001
CENTB1	5312	2591,8815	0,6487	0,0454	0,001
LOC197135	27556	2592,5768	0,6529	0,0454	0,001

MED25	34980	2592,5406	0,6607	0,0454	0,001
MPHOSPH8	35544	2593,5813	0,6631	0,0454	0,001
HS.371060	15243	2594,1086	0,6666	0,0454	0,001
ZNF500	48398	2591,4474	0,6709	0,0454	0,001
CUGBP2	6622	2597,3308	0,6535	0,0456	0,001
CCR6	4866	2599,0556	0,6967	0,0456	0,001
PTP4A2	40237	2600,672	0,6734	0,0457	0,001
LOC729008	33754	2598,9842	0,6886	0,0457	0,001
TRADD	45970	2601,0556	0,692	0,0457	0,001
FKBP15	9508	2603,8103	0,6756	0,0458	0,001
TUBGCP2	46532	2607,3454	0,6635	0,0459	0,001
PSAP	40016	2607,3438	0,6715	0,046	0,001
IL13RA1	25375	2606,6866	0,6885	0,046	0,001
ZNF589	48498	2611,5017	0,6688	0,0461	0,001
GPSM3	11295	2610,7698	0,6718	0,0461	0,001
ZNF30	48218	2614,6306	0,6678	0,0464	0,001
ISCU	25719	2619,3697	0,6748	0,0467	0,001
NAT6	36212	2619,3136	0,6782	0,0467	0,001
SLFN13	43323	2624,3879	0,6812	0,0469	0,001
BSCL2	2545	2625,3034	0,6666	0,047	0,001
GOLGA8B	11042	2633,8387	0,6772	0,0473	0,001
CCDC130	4610	2633,3681	0,6792	0,0473	0,001
AADACL1	34	2632,9794	0,6875	0,0473	0,001
NMI	36698	2634,1977	0,6882	0,0473	0,001
TOP1MT	45856	2632,8355	0,6714	0,0474	0,001
PCMT1	38366	2636,7304	0,6855	0,0474	0,001
PAFAH1B3	38010	2636,9958	0,6981	0,0474	0,001
CKAP2	5621	2638,3433	0,6615	0,0475	0,001
GAK	10487	2640,0407	0,6665	0,0477	0,001
QRICH1	40430	2641,0859	0,6684	0,0477	0,001
LRRC8D	34253	2643,4284	0,6679	0,0478	0,001
ZKSCAN4	47994	2645,6872	0,6762	0,0478	0,001
MTIF3	35855	2645,6123	0,691	0,0478	0,001
GPR18	11211	2648,6441	0,6467	0,048	0,0011
AP1G2	1216	2647,9111	0,6658	0,048	0,001
KIAA0892	26275	2652,0205	0,6623	0,0481	0,0011
PTPLAD1	40252	2652,3689	0,6843	0,0481	0,0011
ABCC5	114	2651,7523	0,6793	0,0482	0,0011
SLC10A7	42787	2655,0175	0,6867	0,0482	0,0011
CDK5RAP3	5181	2654,0693	0,6945	0,0482	0,0011
PLOD3	39190	2657,3753	0,6717	0,0483	0,0011
LETMD1	27146	2659,3829	0,6797	0,0483	0,0011
CRLF3	6301	2661,1667	0,6863	0,0483	0,0011
UBE2L6	46703	2661,6181	0,6863	0,0483	0,0011
LHFPL2	27187	2660,9889	0,6706	0,0484	0,0011
ABHD8	174	2665,8177	0,6653	0,0485	0,0011
KTELC1	26901	2665,0403	0,6671	0,0485	0,0011

PIGS	38881	2664,4753	0,69	0,0485	0,0011
NISCH	36600	2667,2966	0,6589	0,0486	0,0011
HS.576106	23379	2669,8752	0,6629	0,0486	0,0011
CDC16	5060	2667,3401	0,6702	0,0486	0,0011
TSHZ1	46288	2669,7091	0,6715	0,0487	0,0011
SHMT1	42651	2668,794	0,6741	0,0487	0,0011
TMSB10	45697	2673,145	0,6783	0,0487	0,0011
ZNF517	48413	2668,8494	0,6856	0,0487	0,0011
ZNF510	48404	2671,7624	0,6949	0,0487	0,0011
FAM46A	8962	2671,0272	0,837	0,0487	0,0011
EIF3F	8199	2678,393	0,6709	0,0491	0,0011
PTPRO	40316	2680,8668	0,6589	0,0492	0,0011
C11ORF17	2748	2683,6385	0,6608	0,0493	0,0011
ZBTB4	47809	2684,8234	0,6545	0,0494	0,0011
HS.475334	16197	2686,7913	0,6699	0,0495	0,0011

**RP analysis 24h**		**Upregulated**			
	gene.index	RP/Rsum	FC:(class1/class2)	pfp	P.value
CCL4L2	4787	11,5808	8,7435	0	0
CCL4L1	4786	25,6663	7,6943	0	0
DDIT4	7028	25,5553	5,5857	0	0
TRIB3	46044	26,738	5,1818	0	0
RGS1	41117	40,5178	5,0846	0	0
SLC7A5	43262	43,8047	4,5674	0	0
CCL3	4780	102,9678	4,1414	0	0
GZMB	11600	102,0556	4,0722	0	0
MTHFD2	35846	61,9732	4,0213	0	0
CCL3L1	4781	133,9742	3,8379	0	0
CCL3L1	4782	140,4172	3,8229	0	0
CCL3L3	4784	131,2426	3,7966	0	0
MGC4677	35242	73,2208	3,6872	0	0
MYC	35989	91,7037	3,4237	0	0
C20ORF100	3469	155,0222	3,3732	0	0
IGSF3	25325	154,6975	3,3299	0	0
PSAT1	40018	138,6356	3,2664	0	0
FAM152B	8873	100,3335	3,2625	0	0
CD1C	4914	114,4133	3,1973	0	0
DBN1	6926	102,1153	3,195	0	0
RCAN1	40906	121,2957	3,1887	0	0
MTHFD1L	35845	115,1331	3,1865	0	0
OAS3	37203	101,7614	3,1506	0	0
ADM	542	156,1252	3,1057	0	0
LRRC32	34202	151,8198	3,0821	0	0
BATF3	2173	143,0287	3,0711	0	0
SLC1A5	42858	132,8707	3,0381	0	0

IL4I1	25493	209,4351	2,9918	0	0
SUSD1	44469	189,8096	2,9282	0	0
MTHFD2	35848	152,0143	2,9181	0	0
TNFRSF4	45752	260,954	2,9083	0	0
GALR2	10536	132,7895	2,8834	0	0
LILRB4	27235	185,364	2,84	0	0
MYC	35990	152,1715	2,8199	0	0
PHACTR1	38731	169,6765	2,7854	0	0
SERPINE2	42408	234,8295	2,7641	0	0
SLC43A3	43161	135,2445	2,7602	0	0
NME1	36681	169,1077	2,7098	0	0
UCK2	46791	144,7624	2,7057	0	0
NPM3	36854	156,5415	2,704	0	0
OAS1	37196	174,751	2,6969	0	0
DUSP4	7889	156,3804	2,681	0	0
RAMP1	40643	188,5366	2,6778	0	0
ATF5	1777	252,2661	2,6303	0	0
NAMPT	36156	173,1052	2,6254	0	0
GRAMD4	11314	237,614	2,6128	0	0
GFI1	10716	266,2172	2,5836	0	0
TNFRSF9	45759	306,4641	2,568	0	0
TNFRSF18	45741	361,3634	2,5633	0	0
TNFRSF18	45742	288,1148	2,5564	0	0
EGR1	8140	181,1132	2,5545	0	0
PPP1R14B	39582	224,0941	2,5383	0	0
FABP5	8714	203,4045	2,5366	0	0
NOLC1	36760	184,0117	2,5364	0	0
RCN1	40922	265,1276	2,5356	0	0
GALE	10497	197,7251	2,5333	0	0
RPS7	41691	223,9893	2,5112	0	0
GOT1	11070	213,984	2,4593	0	0
DUSP2	7876	217,8625	2,4304	0	0
RPS7	41690	269,3104	2,4282	0	0
IGSF3	25326	368,5483	2,4244	0	0
ASNS	1723	237,0598	2,4216	0	0
LOC399942	28337	371,402	2,4115	0	0
UCHL1	46785	343,7115	2,4112	0	0
CORO6	6110	288,6331	2,4007	0	0
CARD9	4429	295,3633	2,3986	0	0
NAMPT	36157	221,2983	2,3921	0	0
ACP5	291	303,9432	2,3779	0	0
PRR6	39972	272,4763	2,3744	0	0
UBTD1	46765	287,0154	2,3743	0	0
GAPDH	10549	310,5165	2,3718	0	0
CCND2	4811	442,6444	2,3706	0	0
FASN	9140	305,4847	2,3658	0	0
FERMT3	9373	234,7694	2,3654	0	0

XBP1	47636	282,3122	2,3602	0	0
RPL29	41546	242,9078	2,3471	0	0
C19ORF48	3223	302,074	2,3436	0	0
SRM	44062	241,4225	2,3431	0	0
NME1	36683	287,0517	2,337	0	0
CHST7	5575	327,0092	2,3362	0	0
WARS	47320	395,69	2,3356	0	0
NR4A2	36922	301,8126	2,3313	0	0
SLC43A3	43163	230,9937	2,3263	0	0
MTP18	35889	276,3139	2,3081	0	0
GARS	10560	300,7429	2,2951	0	0
RRAGD	41709	315,7747	2,2925	0	0
RPL29	41545	287,9684	2,2918	0	0
SESN2	42424	267,0106	2,2916	0	0
NEK6	36436	290,9181	2,2854	0	0
RYR1	41856	306,7034	2,2813	0	0
ADCY3	500	406,4817	2,2804	0	0
GM2A	10927	389,5312	2,2537	0	0
ENO1	8344	300,8191	2,2533	0	0
CCDC86	4734	279,9345	2,2483	0	0
GAPDH	10551	362,0361	2,2331	0	0
RPS15	41623	346,5413	2,2189	0	0
PIM3	38925	283,3183	2,2104	0	0
GLA	10846	292,0858	2,1999	0	0
PYCR1	40401	339,9643	2,1816	0	0
MRPL12	35615	323,5279	2,1752	0	0
LONP1	34077	344,2596	2,1725	0	0
TXN	46569	369,3176	2,1712	0	0
PRDX4	39766	320,0722	2,17	0	0
PAICS	38025	341,925	2,1602	0	0
RPS24	41637	389,2143	2,1539	0	0
RCAN1	40904	345,2319	2,1531	0	0
PAICS	38027	345,6453	2,1518	0	0
CTPS	6571	352,0815	2,1464	0	0
JTV1	25884	359,9149	2,1399	0	0
SLC3A2	43151	447,6522	2,1364	0	0
LDHA	27091	379,5745	2,1333	0	0
BACE2	2105	301,2011	2,1322	0	0
LOC648024	31452	399,6707	2,1317	0	0
FERMT3	9374	367,0058	2,125	0	0
OAS1	37195	403,5933	2,1154	0	0
ACOT7	275	354,7234	2,1122	0	0
IARS	25139	407,9126	2,112	0	0
BYSL	2627	401,5183	2,1116	0	0
GAPDH	10550	443,1591	2,1103	0	0
PCK2	38362	450,8306	2,1097	0	0
ENTPD1	8369	345,8679	2,0976	0	0

PHGDH	38797	320,2878	2,0968	0	0
TRAF1	45973	420,0937	2,095	0	0
SLC3A2	43149	450,6377	2,0866	0	0
ARPC5L	1595	432,885	2,0758	0	0
TXN	46568	457,3999	2,0665	0	0
XBP1	47637	449,7102	2,0664	0	0
HNRNPAB	12113	412,0785	2,0598	0	0
ZNF593	48501	360,9357	2,0538	0	0
PSMB2	40085	430,8782	2,0499	0	0
RRP12	41726	421,5345	2,0459	0	0
PCK2	38361	460,4322	2,0438	0	0
C17ORF91	3149	451,4828	2,0314	0	0
C17ORF58	3114	401,0442	2,0186	0	0
SLC25A4	42964	424,72	2,0067	0	0
MTP18	35887	434,7314	2,0061	0	0
ACOT9	281	439,0452	2,0052	0	0
NOLA1	36755	428,9486	2,0047	0	0
ASCC1	1695	405,8955	2,0003	0	0
HSPC111	25037	465,2676	1,9894	0	0
SDF2L1	42145	458,5638	1,986	0	0
ARMET	1577	460,0875	1,9521	0	0
HS.25892	14514	455,9527	1,9499	0	0
CST7	6461	478,3647	2,2668	1,00E-04	0
CTH	6537	480,6172	2,2401	1,00E-04	0
SLC6A9	43249	472,6342	2,211	1,00E-04	0
CBS	4534	471,8061	2,2081	1,00E-04	0
CCL3L1	4783	491,5258	2,203	1,00E-04	0
GYPC	11591	484,7207	2,1723	1,00E-04	0
GYPC	11590	474,1834	2,1692	1,00E-04	0
MYCN	35997	523,4124	2,1132	1,00E-04	0
METTL1	35060	484,846	2,0896	1,00E-04	0
FKBP4	9519	479,0614	2,0865	1,00E-04	0
EZH2	8674	475,3545	2,0692	1,00E-04	0
IL21R	25451	519,2534	2,0643	1,00E-04	0
RPL8	41589	492,9831	2,0594	1,00E-04	0
LY9	34373	516,0197	2,0264	1,00E-04	0
EGR2	8141	481,7431	2,026	1,00E-04	0
C19ORF48	3222	499,5806	2,0242	1,00E-04	0
RAB20	40468	471,8313	2,0232	1,00E-04	0
RPL6	41583	488,9115	1,9966	1,00E-04	0
OAS1	37197	512,2759	1,9919	1,00E-04	0
CD58	4989	502,4597	1,9881	1,00E-04	0
ADAM15	404	468,9438	1,9853	1,00E-04	0
LOC732007	34005	518,1739	1,9765	1,00E-04	0
CKS2	5638	523,062	1,9677	1,00E-04	0
C17ORF58	3112	476,5574	1,9315	1,00E-04	0
EBNA1BP2	7988	480,215	1,9303	1,00E-04	0

C17ORF58	3113	470,0258	1,9269	1,00E-04	0
CYCSL1	6745	522,1889	1,901	1,00E-04	0
LYAR	34378	520,6663	1,8985	1,00E-04	0
BACE2	2104	511,0598	1,8604	1,00E-04	0
HOMER1	12151	498,8201	1,8565	1,00E-04	0
CCND2	4812	555,5529	2,2152	2,00E-04	0
WARS	47321	539,7112	2,1103	2,00E-04	0
CDK4	5169	549,8005	2,0231	2,00E-04	0
LOC286016	27801	562,7283	2,0131	2,00E-04	0
LOC644774	30320	535,3023	1,9933	2,00E-04	0
PGAM4	38679	541,297	1,9665	2,00E-04	0
LRP8	34169	529,4704	1,9583	2,00E-04	0
DUSP10	7860	531,9805	1,93	2,00E-04	0
GNPDA1	11012	528,0763	1,9278	2,00E-04	0
PRKCQ	39845	550,8815	1,9267	2,00E-04	0
PGAM1	38675	542,5264	1,9197	2,00E-04	0
SNORA33	43517	535,7035	1,9139	2,00E-04	0
WDR12	47365	553,0044	1,894	2,00E-04	0
WDR12	47366	546,1833	1,8882	2,00E-04	0
SLC25A4	42963	551,9669	1,8709	2,00E-04	0
MTP18	35888	556,4524	1,8564	2,00E-04	0
LYAR	34377	524,2964	1,8562	2,00E-04	0
PSMD12	40119	550,485	1,8392	2,00E-04	0
SIRPA	42720	565,0162	2,0963	3,00E-04	0
FOSB	10103	599,9839	2,0928	3,00E-04	0
PKM2	39010	565,6353	2,0277	3,00E-04	0
LOC653506	33238	616,4894	1,9768	3,00E-04	0
ARID3A	1492	616,1631	1,9727	3,00E-04	0
IL2RB	25481	603,9501	1,9645	3,00E-04	0
LOC653888	33414	610,7809	1,9308	3,00E-04	0
SARS	41960	576,2385	1,9272	3,00E-04	0
SGK	42540	588,4991	1,9169	3,00E-04	0
CD58	4990	590,5428	1,9009	3,00E-04	0
C3ORF26	3735	568,8651	1,8933	3,00E-04	0
PRMT5	39887	570,8546	1,8928	3,00E-04	0
NR4A2	36921	592,1921	1,8913	3,00E-04	0
CKS2	5637	619,7688	1,8825	3,00E-04	0
EZH2	8673	582,6023	1,8817	3,00E-04	0
IMPDH2	25546	620,3669	1,8633	3,00E-04	0
TIMM44	45261	606,5982	1,8551	3,00E-04	0
ISOC2	25731	605,3661	1,8487	3,00E-04	0
LRRC33	34203	627,9549	1,846	3,00E-04	0
PELO	38599	627,6458	1,8414	3,00E-04	0
UBQLN1	46754	590,0308	1,8191	3,00E-04	0
AK2	725	611,1634	1,7888	3,00E-04	0
BCAT1	2226	630,1164	2,0799	4,00E-04	0
KCNK12	26043	650,9881	1,9579	4,00E-04	0

HMGA1	12062	651,2896	1,9501	4,00E-04	0
TPI1	45915	643,5919	1,9403	4,00E-04	0
YARS	47703	649,15	1,9364	4,00E-04	0
MIF	35326	636,4979	1,9014	4,00E-04	0
ALDOA	855	636,4464	1,8743	4,00E-04	0
MRPS7	35721	657,1763	1,8473	4,00E-04	0
TOMM40	45846	629,7603	1,8446	4,00E-04	0
BOP1	2463	639,5288	1,8428	4,00E-04	0
MRTO4	35728	645,9318	1,8247	4,00E-04	0
IFRD2	25259	653,8534	1,7899	4,00E-04	0
CCL22	4769	692,1963	1,9915	5,00E-04	0
NFE2L1	36502	695,2711	1,9874	5,00E-04	0
ASNS	1724	680,0155	1,8963	5,00E-04	0
GPATCH4	11088	674,3128	1,8919	5,00E-04	0
PPA1	39458	662,9724	1,8533	5,00E-04	0
C1QBP	3439	676,1756	1,8503	5,00E-04	0
IPO4	25644	675,775	1,8431	5,00E-04	0
HSP90AB1	24994	682,7466	1,8283	5,00E-04	0
NFKBIE	36523	693,43	1,8278	5,00E-04	0
ACY1	386	683,7554	1,8115	5,00E-04	0
MCM6	34903	663,3964	1,8084	5,00E-04	0
SFXN4	42525	696,2168	1,732	5,00E-04	0
LOC650215	32058	706,09	1,9376	6,00E-04	0
SAMSN1	41937	692,8885	1,8899	6,00E-04	0
PRPF19	39926	707,4826	1,8069	6,00E-04	0
PTGER4	40198	703,7791	1,7956	6,00E-04	0
VEGFA	47168	692,6408	1,792	6,00E-04	0
TUBA1C	46501	735,0732	1,9913	7,00E-04	0
FAM57A	8992	712,2816	1,8981	7,00E-04	0
PLEK	39121	721,4873	1,8933	7,00E-04	0
TUBB2C	46516	712,4362	1,8329	7,00E-04	0
WDR4	47408	711,7652	1,7894	7,00E-04	0
LOC650832	32232	733,9784	1,7677	7,00E-04	0
PSMB5	40088	711,2677	1,7667	7,00E-04	0
HSPA9	25022	722,6112	1,7372	7,00E-04	0
APOD	1316	720,871	1,5132	7,00E-04	0
TUBB	46510	783,8324	1,9748	8,00E-04	0
FOS	10102	759,7968	1,9218	8,00E-04	0
TPI1	45914	753,3025	1,8749	8,00E-04	0
UBE2F	46684	781,5174	1,86	8,00E-04	0
HYOU1	25133	768,7565	1,8293	8,00E-04	0
HNRNPAB	12114	773,445	1,8209	8,00E-04	0
ACOX3	286	725,1657	1,8027	8,00E-04	0
DDX21	7068	727,4917	1,8018	8,00E-04	0
PRMT5	39888	775,0149	1,7999	8,00E-04	0
PGAM1	38674	723,8513	1,7921	8,00E-04	0
ABCB6	91	762,3996	1,791	8,00E-04	0

TALDO1	44689	760,9221	1,787	8,00E-04	0
PEA15	38582	782,7147	1,7857	8,00E-04	0
HAGHL	11646	748,0403	1,7839	8,00E-04	0
NOLA2	36757	769,9792	1,78	8,00E-04	0
TRAP1	46003	760,8324	1,7789	8,00E-04	0
PRDX1	39759	745,0308	1,749	8,00E-04	0
DCUN1D5	7002	758,4923	1,7288	8,00E-04	0
RRP15	41727	782,2348	1,7268	8,00E-04	0
SLAMF7	42774	762,7884	1,7215	8,00E-04	0
PSMD8	40132	787,3113	1,7214	8,00E-04	0
NIP7	36589	749,5985	1,7181	8,00E-04	0
NME1	36682	733,57	1,7156	8,00E-04	0
LOC731049	33952	747,413	1,6999	8,00E-04	0
RPL34	41559	797,9589	1,8438	9,00E-04	0
PGAM4	38680	799,2918	1,8414	9,00E-04	0
AK2	723	791,615	1,7738	9,00E-04	0
SRXN1	44089	801,9715	1,7647	9,00E-04	0
PGAM4	38678	795,3471	1,7618	9,00E-04	0
CD151	4901	772,0649	1,7451	9,00E-04	0
PEMT	38601	796,7497	1,7385	9,00E-04	0
NOLA1	36756	803,3059	1,7362	9,00E-04	0
TNFSF13B	45769	797,2677	1,7307	9,00E-04	0
PDSS1	38556	790,0508	1,7186	9,00E-04	0
OPRS1	37339	772,8199	1,6932	9,00E-04	0
CCDC90A	4740	799,8625	1,691	9,00E-04	0
UCHL5	46787	791,0756	1,688	9,00E-04	0
RPS21	41635	822,2133	1,7956	0,001	0
EIF4A1	8208	833,7316	1,7877	0,001	0
ANKRD37	1121	829,5809	1,7816	0,001	0
HSPD1	25044	817,312	1,7718	0,001	0
PPP1R15A	39585	827,719	1,7708	0,001	0
PRNP	39895	820,9433	1,7639	0,001	0
SLC25A19	42926	820,2646	1,7608	0,001	0
HNRNPAB	12115	813,4221	1,7561	0,001	0
DCXR	7006	825,2465	1,7297	0,001	0
ATP1B3	1854	823,1197	1,7174	0,001	0
NEU1	36454	833,6822	1,7128	0,001	0
ACOT7	277	832,372	1,705	0,001	0
NDUFAF2	36365	815,6106	1,6935	0,001	0
SLC7A1	43251	848,9279	1,8153	0,0011	0
SLC25A22	42932	839,1313	1,78	0,0011	0
FKBP2	9514	837,1227	1,7593	0,0011	0
RNH1	41418	845,8375	1,7515	0,0011	0
PRMT3	39886	849,9474	1,6745	0,0011	0
HMBS	12050	852,5185	1,7275	0,0012	0
TUBA1A	46498	877,2215	1,9311	0,0013	0
DUSP5	7890	882,2129	1,7846	0,0013	0

LOC643300	29632	877,6612	1,7591	0,0013	0
PRMT1	39881	862,5162	1,7566	0,0013	0
PSPH	40151	864,1028	1,7459	0,0013	0
SAP30	41941	867,5344	1,7115	0,0013	0
RUVBL1	41834	863,9752	1,7086	0,0013	0
SNHG3-RCC1	43505	866,2988	1,6673	0,0013	0
NETO2	36453	861,7245	1,6597	0,0013	0
SIRPA	42721	896,9534	1,7989	0,0014	0
PGK1	38699	900,1027	1,7449	0,0014	0
VDAC1	47163	892,981	1,737	0,0014	0
BCAR3	2217	887,5895	1,7363	0,0014	0
PUS7	40359	899,8115	1,7309	0,0014	0
KCNG1	25972	887,0405	1,7279	0,0014	0
STIP1	44291	880,5509	1,7034	0,0014	0
C3ORF26	3734	889,7745	1,6975	0,0014	0
NANS	36166	881,5745	1,6971	0,0014	0
NUP188	37128	896,8624	1,6864	0,0014	0
RANGAP1	40665	882,0445	1,6799	0,0014	0
EGR3	8142	889,8775	1,6746	0,0014	0
BOLA3	2456	884,9519	1,671	0,0014	0
ATL3	1809	891,0036	1,6631	0,0014	0
BXDC1	2623	901,1748	1,637	0,0014	0
LOC221710	27605	885,0292	1,632	0,0014	0
HES6	11822	910,499	1,7492	0,0015	0
C16ORF14	3038	912,7669	1,6938	0,0015	0
NSMAF	36998	917,6556	1,6812	0,0015	0
CIRH1A	5611	909,948	1,67	0,0015	0
WDR43	47418	915,0316	1,6426	0,0015	0
AARS	47	923,2066	1,8339	0,0016	0
HOMER2	12153	927,7559	1,73	0,0016	0
DDIT3	7027	925,8145	1,6886	0,0016	0
LHFP	27185	934,4076	1,943	0,0017	0
PSMD3	40124	934,7753	1,6716	0,0017	0
KDELR2	26139	936,9095	1,6686	0,0017	0
RUVBL1	41833	938,1846	1,6535	0,0017	0
SHMT2	42654	949,352	1,776	0,0018	0
DPP3	7720	946,6303	1,753	0,0018	0
GLRX2	10888	944,0718	1,6478	0,0018	0
HYOU1	25132	951,4563	1,7305	0,0019	0
METRNL	35053	963,7437	1,7476	0,002	0
TMEM97	45653	965,2231	1,7157	0,002	0
CBX6	4550	962,4639	1,675	0,002	0
SRPRB	44080	960,0024	1,6424	0,002	0
TGFBR3	45122	965,1636	1,6225	0,002	0
BZW2	2636	968,6231	1,7071	0,0021	0
HSPC171	25040	968,7452	1,6627	0,0021	0
MPP6	35556	977,6495	1,6137	0,0021	0

HLA-DRB6	12027	977,9206	1,4916	0,0021	0
HS.132448	12977	989,8519	1,7622	0,0022	0
PLEKHA7	39133	980,4752	1,6985	0,0022	0
C17ORF79	3138	981,4557	1,6681	0,0022	0
LOC651816	32494	986,1762	1,642	0,0022	0
SLC35B1	43078	991,1379	1,6418	0,0022	0
STX11	44361	997,7774	1,7541	0,0023	0
TKT	45295	1001,4143	1,7343	0,0023	0
C6ORF129	3888	996,8202	1,7266	0,0023	0
MAPKAPK3	34724	1000,2894	1,7115	0,0023	0
PFKP	38658	998,3799	1,6949	0,0023	0
FKBP11	9505	1005,2343	1,6588	0,0023	0
STT3A	44356	997,8975	1,6539	0,0023	0
GALNT1	10508	1000,4487	1,6438	0,0023	0
BAG3	2116	1005,1401	1,6328	0,0023	0
PPIL1	39529	1008,2824	1,6265	0,0023	0
NFKB1	36514	1027,8563	1,7485	0,0024	0
LOC647000	31120	1017,1223	1,7073	0,0024	0
GPI	11114	1026,4117	1,6781	0,0024	0
C5ORF30	3843	1012,3144	1,6656	0,0024	0
NAT10	36203	1020,8331	1,6587	0,0024	0
SFXN1	42522	1026,0872	1,644	0,0024	0
GPR137B	11166	1012,8268	1,6265	0,0024	0
CRELD2	6269	1026,4208	1,6261	0,0024	0
MAFG	34473	1019,9361	1,6227	0,0024	0
LOC647000	31119	1032,9214	1,8111	0,0025	0
KIAA0020	26162	1036,3374	1,6832	0,0025	0
PSMD14	40122	1030,5516	1,6583	0,0025	0
SDSL	42179	1046,6892	1,7993	0,0026	0
SNX5	43646	1049,6895	1,7009	0,0026	0
SLC39A14	43132	1040,1615	1,6658	0,0026	0
PLOD1	39186	1050,5106	1,6567	0,0026	0
ODC1	37233	1042,0138	1,6484	0,0026	0
BRF2	2511	1044,6727	1,6178	0,0026	0
GNL3	11004	1049,2288	1,6157	0,0026	0
PDCD5	38428	1060,4331	1,6426	0,0027	0
PTPRE	40304	1064,9869	1,6742	0,0028	0
ITGAX	25763	1062,886	1,6309	0,0028	0
POLR2D	39364	1062,648	1,6011	0,0028	0
UTP11L	47070	1078,0688	1,6699	0,0029	0
PCK2	38363	1079,1884	1,6375	0,0029	0
PAK1IP1	38037	1084,4762	1,6293	0,003	0
ELL2	8275	1093,1222	1,6712	0,0031	0
TBC1D7	44797	1094,2622	1,6603	0,0031	0
STAT3	44256	1089,256	1,641	0,0031	0
FABP5	8715	1094,1261	1,6388	0,0031	0
RPL13	41515	1090,8455	1,6045	0,0031	0

NFIL3	36512	1103,0384	1,5838	0,0032	0
TM6SF1	45347	1106,0268	1,7226	0,0033	0
GART	10562	1105,4977	1,6688	0,0033	0
NME2	36687	1110,0349	1,6658	0,0033	0
ATP1B3	1852	1110,3948	1,6292	0,0033	0
EEF1B2	8061	1104,7353	1,5921	0,0033	0
C1ORF19	3347	1111,4085	1,7288	0,0034	0
NHP2L1	36569	1112,7985	1,5846	0,0034	0
PKM2	39012	1120,7792	1,6731	0,0035	0
PDLIM7	38538	1121,8309	1,5729	0,0035	0
HSPD1	25047	1128,644	1,6968	0,0036	0
ATOX1	1823	1124,6867	1,6874	0,0036	0
CGI-96	5427	1139,4241	1,6234	0,0036	0
EXOSC4	8641	1138,095	1,615	0,0036	0
SEH1L	42236	1131,5394	1,6036	0,0036	0
G6PD	10398	1128,4428	1,5823	0,0036	0
QPCT	40424	1135,0306	1,714	0,0037	0
LPIN1	34117	1154,4279	1,6894	0,0037	0
C19ORF10	3179	1147,0022	1,6532	0,0037	0
ENDOG	8341	1143,609	1,643	0,0037	0
MAP2K3	34621	1133,0153	1,6171	0,0037	0
PI4K2A	38836	1144,8213	1,6148	0,0037	0
KPNA2	26712	1153,2258	1,6645	0,0038	0
PSMC3	40100	1159,9127	1,6297	0,0038	0
RENBP	40989	1153,464	1,6184	0,0038	0
LOC732165	34018	1164,721	1,6999	0,0039	0
NUP93	37149	1170,3025	1,6686	0,004	0
VCP	47146	1168,3753	1,6052	0,004	0
NP	36808	1172,0829	1,7075	0,0041	0
PRDX1	39760	1173,2069	1,6543	0,0041	0
BOLA2	2455	1171,1397	1,6463	0,0041	0
MRPL24	35633	1186,286	1,6211	0,0042	0
BIRC7	2372	1185,2368	1,6166	0,0042	0
GUF1	11572	1193,0142	1,5928	0,0042	0
BEX2	2329	1184,3321	1,451	0,0042	0
CHCHD6	5448	1207,2397	1,6754	0,0043	0
EBI3	7987	1199,8601	1,6656	0,0043	0
NCBP2	36287	1203,3669	1,6431	0,0043	0
TCEB3	44897	1199,0828	1,633	0,0043	0
P4HB	37971	1196,4537	1,6106	0,0043	0
AHCY	682	1188,3953	1,6023	0,0043	0
EIF4G1	8224	1216,0525	1,5922	0,0043	0
RYR1	41855	1200,5518	1,5891	0,0043	0
PFN1	38660	1197,5095	1,5797	0,0043	0
FAH	8730	1213,5812	1,5793	0,0043	0
TXNL2	46597	1208,1015	1,5768	0,0043	0
EIF4EBP1	8219	1213,6301	1,5646	0,0043	0

NOL14	36740	1205,2965	1,557	0,0043	0
NME1-NME2	36684	1196,1755	1,5563	0,0043	0
LONRF3	34083	1201,1049	1,551	0,0043	0
ITGB2	25776	1197,7562	1,5382	0,0043	0
HLA-DRB1	12022	1183,8571	1,2992	0,0043	0
ABCE1	134	1221,0013	1,6219	0,0044	0
ALKBH2	882	1225,0402	1,6405	0,0045	0
PHEX	38748	1238,7805	1,7199	0,0046	0
PER2	38609	1226,8165	1,6011	0,0046	0
GART	10561	1234,3887	1,5968	0,0046	0
AMY1A	981	1241,2738	1,5845	0,0046	0
C21ORF70	3624	1237,9087	1,5845	0,0046	0
MAP1LC3B	34609	1237,7244	1,582	0,0046	0
ECE2	7996	1232,3816	1,5701	0,0046	0
PDSS1	38555	1231,9883	1,5689	0,0046	0
YRDC	47736	1230,1944	1,5595	0,0046	0
ZNF259	48170	1229,3786	1,548	0,0046	0
PKM2	39011	1246,0441	1,6773	0,0047	0
PGK1	38700	1253,5317	1,6474	0,0047	0
SCD	42024	1248,5184	1,6273	0,0047	0
XTP3TPA	47694	1245,4256	1,6222	0,0047	0
UBQLN1	46753	1244,8101	1,5284	0,0047	0
GLRX2	10886	1255,5914	1,5774	0,0048	0
DHRS9	7291	1258,5919	1,784	0,0049	0
TRMT1	46183	1260,7421	1,624	0,0049	0
ANXA11	1179	1261,4697	1,5999	0,0049	0
MAP3K6	34649	1274,674	1,6147	0,005	0
SLC1A4	42857	1272,2145	1,6052	0,005	0
C20ORF59	3541	1270,1808	1,593	0,005	0
CPEB1	6159	1269,6468	1,5766	0,005	0
HSPBP1	25032	1265,0434	1,5434	0,005	0
APOC1	1312	1264,6717	1,5409	0,005	0
STRA13	44342	1272,0895	1,534	0,005	0
PARVB	38147	1279,2362	1,7987	0,0051	1,00E-04
PRDM1	39735	1291,2458	1,7649	0,0051	1,00E-04
RFTN1	41053	1291,9491	1,6584	0,0051	1,00E-04
CCND1	4810	1281,5235	1,6576	0,0051	1,00E-04
ARPC1B	1586	1295,1137	1,6314	0,0051	1,00E-04
ATP1B3	1856	1291,9499	1,5744	0,0051	1,00E-04
BOLA3	2457	1280,216	1,5688	0,0051	1,00E-04
PTRH1	40331	1290,3255	1,5602	0,0051	1,00E-04
C19ORF24	3195	1271,5983	1,5593	0,0051	0
GSPT1	11451	1290,5525	1,5426	0,0051	1,00E-04
ARMC6	1558	1277,3479	1,5325	0,0051	1,00E-04
GFPT1	10723	1291,9135	1,5324	0,0051	1,00E-04
VARS	47121	1289,7775	1,6857	0,0052	1,00E-04
XPOT	47677	1300,1521	1,6394	0,0052	1,00E-04

ALDOA	853	1286,5353	1,6257	0,0052	1,00E-04
TOR3A	45876	1283,9091	1,5925	0,0052	1,00E-04
QSOX1	40435	1287,6866	1,592	0,0052	1,00E-04
SLC3A2	43150	1300,6853	1,5828	0,0052	1,00E-04
EHD4	8149	1310,6122	1,5753	0,0052	1,00E-04
MRPL24	35634	1294,0179	1,5652	0,0052	1,00E-04
C8ORF33	4045	1294,3169	1,5529	0,0052	1,00E-04
ETF1	8563	1304,0466	1,5474	0,0052	1,00E-04
PSPH	40150	1293,215	1,5464	0,0052	1,00E-04
MAPK6	34704	1301,0218	1,5277	0,0052	1,00E-04
PPME1	39566	1312,0596	1,5242	0,0052	1,00E-04
IPO11	25642	1306,8304	1,521	0,0052	1,00E-04
RNASEH2A	41285	1309,6031	1,5179	0,0052	1,00E-04
CTLA4	6542	1283,9199	1,5116	0,0052	1,00E-04
RPS19BP1	41632	1303,0341	1,5083	0,0052	1,00E-04
CARS	4433	1323,5992	1,6497	0,0053	1,00E-04
TXNRD1	46600	1315,4615	1,6418	0,0053	1,00E-04
LOC646197	30835	1319,0875	1,6371	0,0053	1,00E-04
ANXA2	1185	1322,0569	1,5759	0,0053	1,00E-04
PPAN	39462	1308,7205	1,5628	0,0053	1,00E-04
HSPBP1	25033	1317,5761	1,5597	0,0053	1,00E-04
PDIA6	38520	1315,9867	1,5582	0,0053	1,00E-04
RUVBL2	41835	1327,2234	1,5579	0,0053	1,00E-04
POLDIP2	39334	1325,2511	1,5261	0,0053	1,00E-04
SLC16A6	42835	1331,7172	1,6284	0,0054	1,00E-04
NCOA7	36318	1331,4822	1,5771	0,0054	1,00E-04
ATAD3A	1759	1332,5355	1,5441	0,0054	1,00E-04
HSPA8	25020	1331,9985	1,5224	0,0054	1,00E-04
CDKN1A	5196	1339,3334	1,77	0,0055	1,00E-04
STAT3	44258	1338,069	1,6052	0,0055	1,00E-04
DCUN1D5	7003	1340,461	1,5777	0,0055	1,00E-04
MRRF	35723	1342,7995	1,5356	0,0055	1,00E-04
MRPS2	35701	1339,5171	1,5331	0,0055	1,00E-04
ATP6V0B	1929	1345,159	1,5089	0,0056	1,00E-04
E2F6	7971	1352,8471	1,566	0,0058	1,00E-04
DKC1	7385	1351,3942	1,5034	0,0058	1,00E-04
NR4A3	36926	1357,3501	1,6073	0,0059	1,00E-04
LOC652545	32754	1355,4595	1,5834	0,0059	1,00E-04
SERTAD1	42417	1357,7608	1,5695	0,0059	1,00E-04
C8ORF33	4044	1358,1353	1,5473	0,0059	1,00E-04
SCPEP1	42095	1367,6553	1,5863	0,006	1,00E-04
OXCT2	37916	1372,0845	1,5779	0,006	1,00E-04
ECE2	7997	1360,6969	1,5645	0,006	1,00E-04
RHOC	41189	1367,9739	1,5592	0,006	1,00E-04
SAR1B	41955	1371,054	1,5564	0,006	1,00E-04
ARMC10	1553	1362,8897	1,5429	0,006	1,00E-04
KIAA1553	26376	1363,7804	1,5353	0,006	1,00E-04

GLI1	10859	1365,1071	1,5302	0,006	1,00E-04
RBM28	40824	1360,6134	1,529	0,006	1,00E-04
KEAP1	26146	1383,2441	1,5739	0,0061	1,00E-04
PMVK	39271	1377,8425	1,5103	0,0061	1,00E-04
TTYH3	46494	1385,2293	1,673	0,0062	1,00E-04
ATP5G1	1899	1387,7369	1,5718	0,0062	1,00E-04
LOC653381	33171	1383,8242	1,5508	0,0062	1,00E-04
TWF2	46557	1386,9485	1,5184	0,0062	1,00E-04
TUBG1	46529	1392,529	1,6293	0,0063	1,00E-04
TMED9	45392	1395,876	1,6023	0,0063	1,00E-04
RPL36	41563	1397,525	1,5761	0,0063	1,00E-04
FKBP1A	9509	1396,737	1,5605	0,0063	1,00E-04
CSTF2	6471	1395,8712	1,5412	0,0063	1,00E-04
LMNB2	27320	1402,785	1,5635	0,0064	1,00E-04
SRP68	44070	1404,0838	1,5484	0,0064	1,00E-04
SQSTM1	44026	1398,861	1,5259	0,0064	1,00E-04
CALR	4336	1406,4716	1,5874	0,0065	1,00E-04
LOC643668	29790	1411,6407	1,5502	0,0066	1,00E-04
RQCD1	41701	1408,3595	1,5475	0,0066	1,00E-04
C3ORF68	3783	1413,0304	1,4764	0,0066	1,00E-04
AK3L1	727	1417,1681	1,7477	0,0067	1,00E-04
ATP2A2	1864	1421,8646	1,6712	0,0067	1,00E-04
RGS16	41132	1417,3895	1,6131	0,0067	1,00E-04
TUBB	46511	1414,4824	1,6096	0,0067	1,00E-04
EIF6	8243	1419,3822	1,5911	0,0067	1,00E-04
HK2	11998	1417,8423	1,5851	0,0067	1,00E-04
ABCE1	133	1417,7057	1,5539	0,0067	1,00E-04
SPAG9	43804	1415,6809	1,5098	0,0067	1,00E-04
CDK5R1	5171	1429,169	1,6007	0,0068	1,00E-04
MRPL15	35618	1427,2082	1,578	0,0068	1,00E-04
HCFC1R1	11704	1430,6406	1,5175	0,0068	1,00E-04
MAFG	34474	1429,6522	1,4953	0,0068	1,00E-04
C3ORF14	3717	1427,2955	1,4307	0,0068	1,00E-04
CD83	5018	1432,9651	1,734	0,0069	1,00E-04
SFXN4	42526	1432,445	1,4868	0,0069	1,00E-04
ATP2A2	1862	1439,8708	1,632	0,007	1,00E-04
WBSCR22	47351	1440,805	1,5729	0,007	1,00E-04
MYO1G	36074	1442,8261	1,568	0,0071	1,00E-04
RANBP1	40650	1446,8098	1,5466	0,0071	1,00E-04
HSD17B12	24956	1446,569	1,5295	0,0071	1,00E-04
CCT2	4881	1456,1115	1,6258	0,0072	1,00E-04
PPRC1	39673	1455,9237	1,5706	0,0073	1,00E-04
ATP5G1	1898	1458,594	1,5657	0,0073	1,00E-04
MRPS17	35695	1453,9577	1,5337	0,0073	1,00E-04
PGM2	38710	1455,4124	1,5155	0,0073	1,00E-04
SNRPD1	43585	1453,5257	1,5037	0,0073	1,00E-04
EXOSC5	8642	1460,9409	1,4747	0,0073	1,00E-04

B4GALT5	2093	1468,535	1,7287	0,0074	1,00E-04
APRT	1345	1471,3596	1,5424	0,0074	1,00E-04
EDG4	8038	1471,3662	1,5342	0,0074	1,00E-04
TMEM147	45455	1466,3975	1,5291	0,0074	1,00E-04
TUFM	46539	1468,8738	1,5113	0,0074	1,00E-04
GSTA4	11462	1471,5091	1,4937	0,0074	1,00E-04
COMTD1	6067	1466,2952	1,4931	0,0074	1,00E-04
WDR74	47464	1471,1393	1,4579	0,0074	1,00E-04
KLF4	26592	1482,134	1,624	0,0075	1,00E-04
PRNP	39894	1476,9537	1,5695	0,0075	1,00E-04
EZH2	8672	1477,2124	1,5354	0,0075	1,00E-04
GNL3	11003	1481,7085	1,5321	0,0075	1,00E-04
LOC728689	33700	1481,8669	1,5127	0,0075	1,00E-04
BCCIP	2230	1480,2955	1,4861	0,0075	1,00E-04
PGAM5	38681	1484,5186	1,6233	0,0076	1,00E-04
PA2G4	37977	1488,6975	1,6207	0,0076	1,00E-04
EIF4G1	8226	1489,3912	1,5426	0,0076	1,00E-04
POLR1C	39354	1491,1006	1,5368	0,0076	1,00E-04
YIF1A	47712	1484,7835	1,5087	0,0076	1,00E-04
MRRF	35724	1491,9746	1,4987	0,0076	1,00E-04
GHITM	10774	1483,3385	1,4845	0,0076	1,00E-04
NOL5A	36746	1495,0988	1,6159	0,0077	1,00E-04
KIAA0664	26243	1495,4279	1,5309	0,0077	1,00E-04
SIL1	42703	1498,649	1,5279	0,0078	1,00E-04
RRS1	41730	1509,5934	1,5833	0,008	1,00E-04
PNPO	39301	1509,6758	1,566	0,008	1,00E-04
MTMR14	35869	1508,6727	1,5313	0,008	1,00E-04
MRPL37	35651	1509,1939	1,522	0,008	1,00E-04
ARD1A	1377	1510,2584	1,5122	0,008	1,00E-04
ELOVL6	8295	1514,2892	1,5606	0,0081	1,00E-04
PSMD2	40123	1523,5891	1,518	0,0083	1,00E-04
UBE2M	46705	1521,0954	1,5151	0,0083	1,00E-04
COMT	6066	1523,5383	1,5003	0,0083	1,00E-04
ATG7	1802	1525,2167	1,5717	0,0084	1,00E-04
PRKAR1B	39830	1530,455	1,5136	0,0085	1,00E-04
GK	10839	1529,4438	1,5132	0,0085	1,00E-04
TTLL12	46427	1537,4026	1,546	0,0086	1,00E-04
PINX1	38930	1537,7344	1,4902	0,0086	1,00E-04
AP1S1	1222	1538,2671	1,4738	0,0086	1,00E-04
TUBG1	46530	1547,816	1,5643	0,0087	1,00E-04
FAM86A	9062	1557,4457	1,5391	0,0089	1,00E-04
ILVBL	25529	1552,0116	1,5012	0,0089	1,00E-04
TMED3	45385	1561,1202	1,5114	0,009	1,00E-04
CANX	4376	1560,6474	1,5037	0,009	1,00E-04
SLC16A3	42830	1568,185	1,5516	0,0092	1,00E-04
IDE	25178	1568,9643	1,4901	0,0092	1,00E-04
PFAS	38641	1579,2036	1,5406	0,0093	1,00E-04

FDX1L	9353	1576,143	1,512	0,0093	1,00E-04
BCS1L	2301	1579,3551	1,4868	0,0093	1,00E-04
PSMD7	40131	1575,5898	1,481	0,0093	1,00E-04
GPATCH4	11089	1586,2201	1,5871	0,0095	1,00E-04
CASP3	4459	1584,5901	1,5705	0,0095	1,00E-04
IMP4	25536	1596,2844	1,5147	0,0097	1,00E-04
MRPL23	35632	1593,7358	1,4911	0,0097	1,00E-04
NDFIP2	36332	1606,0321	1,5579	0,0099	1,00E-04
DPM2	7712	1600,8259	1,5188	0,0099	1,00E-04
ATP6V0B	1930	1601,383	1,514	0,0099	1,00E-04
LOC646531	30955	1602,9052	1,503	0,0099	1,00E-04
LOC652595	32781	1604,6474	1,4905	0,0099	1,00E-04
VKORC1L1	47207	1603,1149	1,4855	0,0099	1,00E-04
ETF1	8564	1602,9389	1,4691	0,0099	1,00E-04
PSMA1	40070	1609,6245	1,5474	0,01	1,00E-04
EIF3B	8193	1608,8276	1,5123	0,01	1,00E-04
EPGN	8407	1608,3909	1,5027	0,01	1,00E-04
ADM2	543	1611,7816	1,5499	0,0101	1,00E-04
KIF21B	26483	1617,6752	1,5107	0,0101	1,00E-04
ZSCAN2	48761	1614,828	1,503	0,0101	1,00E-04
TPM3	45925	1614,8912	1,4982	0,0101	1,00E-04
POMP	39404	1616,1734	1,4755	0,0101	1,00E-04
TNFSF13B	45770	1615,1801	1,468	0,0101	1,00E-04
FTSJ1	10300	1611,6209	1,4488	0,0101	1,00E-04
YIF1B	47714	1623,3796	1,4669	0,0103	1,00E-04
MARS	34767	1630,4359	1,6064	0,0104	1,00E-04
RAB40B	40519	1627,669	1,5576	0,0104	1,00E-04
TIMM23	45257	1627,4706	1,5237	0,0104	1,00E-04
FKBP1A	9511	1630,3568	1,5206	0,0104	1,00E-04
PSMB3	40086	1625,9638	1,5161	0,0104	1,00E-04
JOSD1	25870	1632,8883	1,4671	0,0104	1,00E-04
APRT	1346	1634,7082	1,5141	0,0105	1,00E-04
LOC391811	28285	1639,5779	1,5077	0,0107	1,00E-04
IGF2R	25290	1640,2593	1,5007	0,0107	1,00E-04
STX3	44373	1642,3684	1,5109	0,0108	1,00E-04
ANXA11	1180	1643,7422	1,4886	0,0108	2,00E-04
E2F3	7967	1641,274	1,4598	0,0108	1,00E-04
LOC100008589	27345	1650,4694	1,501	0,0109	2,00E-04
PTTG3	40341	1652,689	1,484	0,0109	2,00E-04
ZDHHC9	47905	1652,0695	1,4776	0,0109	2,00E-04
WDR74	47462	1651,0002	1,4693	0,0109	2,00E-04
PMM2	39244	1650,2738	1,4681	0,0109	2,00E-04
PDXP	38561	1652,0179	1,4533	0,0109	2,00E-04
RASAL1	40714	1654,1184	1,5246	0,011	2,00E-04
DPH3	7705	1651,9328	1,4976	0,011	2,00E-04
EXOSC7	8644	1653,914	1,4885	0,011	2,00E-04
DPH2	7703	1656,9627	1,4823	0,011	2,00E-04

NOLA2	36758	1659,6247	1,5756	0,0111	2,00E-04
PRELID1	39776	1661,7458	1,5049	0,0111	2,00E-04
CANX	4377	1662,2541	1,4754	0,0111	2,00E-04
DUSP10	7861	1673,8268	1,5182	0,0114	2,00E-04
TUBA1B	46500	1676,0526	1,6033	0,0115	2,00E-04
ITGB4BP	25784	1678,6257	1,5169	0,0116	2,00E-04
KEAP1	26144	1682,4889	1,5122	0,0116	2,00E-04
SURF4	44467	1683,7727	1,5048	0,0116	2,00E-04
STAT3	44257	1681,9675	1,4965	0,0116	2,00E-04
MRPL17	35620	1682,1355	1,4875	0,0116	2,00E-04
YWHAG	47753	1683,2756	1,486	0,0116	2,00E-04
HAX1	11679	1680,7657	1,4571	0,0116	2,00E-04
CISD1	5612	1680,2129	1,4985	0,0117	2,00E-04
BAX	2174	1689,8488	1,5147	0,0118	2,00E-04
MRPL13	35616	1691,4166	1,4853	0,0118	2,00E-04
LOC728564	33683	1692,6277	1,4611	0,0119	2,00E-04
WDR46	47425	1704,5741	1,5369	0,0122	2,00E-04
KLK1	26654	1708,3795	1,5061	0,0122	2,00E-04
DDX39	7080	1707,9321	1,4631	0,0122	2,00E-04
AP1S1	1221	1703,981	1,4453	0,0122	2,00E-04
LOC731682	33986	1707,7322	1,258	0,0122	2,00E-04
NOL1	36734	1713,4318	1,6021	0,0123	2,00E-04
TIMM8A	45263	1710,8268	1,4852	0,0123	2,00E-04
LOC285216	27770	1712,9175	1,4783	0,0123	2,00E-04
C6ORF125	3884	1712,033	1,4745	0,0123	2,00E-04
GTPBP4	11546	1717,7355	1,49	0,0124	2,00E-04
TST	46367	1716,1474	1,4895	0,0124	2,00E-04
BOLA2	2454	1717,7819	1,4838	0,0124	2,00E-04
ICOSLG	25169	1719,8161	1,4781	0,0124	2,00E-04
CD320	4953	1720,0847	1,4732	0,0124	2,00E-04
MAD2L2	34454	1719,1244	1,468	0,0124	2,00E-04
PSMC2	40099	1724,2292	1,4718	0,0125	2,00E-04
HLA-DPB2	12014	1726,8511	1,6079	0,0126	2,00E-04
BRP44	2522	1733,7452	1,4891	0,0127	2,00E-04
DUSP4	7888	1730,9393	1,4846	0,0127	2,00E-04
C10ORF2	2664	1732,2502	1,4793	0,0127	2,00E-04
MRPS6	35720	1735,1474	1,4276	0,0127	2,00E-04
PIPSL	38949	1734,9497	1,51	0,0128	2,00E-04
MOCOS	35493	1744,9804	1,5896	0,0129	2,00E-04
SNORA10	43514	1743,6191	1,5139	0,0129	2,00E-04
C1ORF24	3369	1746,9294	1,5081	0,0129	2,00E-04
BOLA2	2450	1743,0385	1,4917	0,0129	2,00E-04
GNG8	10998	1740,557	1,3479	0,0129	2,00E-04
RHOC	41190	1748,7973	1,4771	0,013	2,00E-04
YBX1	47705	1744,7165	1,4737	0,013	2,00E-04
SELS	42252	1749,1475	1,4379	0,013	2,00E-04
LOC400455	28359	1753,702	1,4665	0,0131	2,00E-04

ATP1A1	1841	1752,0127	1,4638	0,0131	2,00E-04
MPDU1	35533	1756,4873	1,5124	0,0132	2,00E-04
ACSL1	306	1756,1247	1,5047	0,0132	2,00E-04
TIPIN	45278	1755,651	1,4323	0,0132	2,00E-04
KCNK4	26056	1760,6748	1,4734	0,0133	2,00E-04
PGM1	38709	1762,6691	1,5559	0,0134	2,00E-04
MGC13057	35145	1767,0285	1,4843	0,0135	2,00E-04
ELK1	8268	1769,9218	1,4351	0,0135	2,00E-04
BOLA2	2452	1767,5452	1,5221	0,0136	2,00E-04
TIMM10	45252	1771,0715	1,4939	0,0136	2,00E-04
HCST	11729	1774,1033	1,4938	0,0136	2,00E-04
YIF1B	47715	1773,2217	1,4695	0,0136	2,00E-04
ABCB1	85	1773,3391	1,454	0,0136	2,00E-04
LOC643668	29789	1786,6439	1,5309	0,0138	2,00E-04
MGC40489	35221	1783,4211	1,4911	0,0138	2,00E-04
SCO2	42091	1784,6343	1,5245	0,0139	2,00E-04
SH2D5	42581	1787,789	1,4845	0,0139	2,00E-04
ECGF1	8001	1793,3441	1,5309	0,014	2,00E-04
PFN1	38659	1794,0527	1,5177	0,014	2,00E-04
TCEB1	44892	1793,8996	1,4678	0,014	2,00E-04
LY9	34374	1795,1263	1,4544	0,0141	2,00E-04
GCLM	10643	1797,961	1,4595	0,0142	2,00E-04
EMG1	8306	1800,2175	1,5591	0,0143	2,00E-04
SCN3A	42070	1807,1073	1,5046	0,0144	2,00E-04
MT2A	35818	1806,3903	1,4419	0,0144	2,00E-04
ITGB2	25777	1808,2474	1,4081	0,0144	2,00E-04
TCP11L1	44963	1813,5266	1,4429	0,0146	2,00E-04
PSMD1	40110	1816,5087	1,5208	0,0147	2,00E-04
ACTN4	361	1823,0535	1,4746	0,0148	2,00E-04
C9ORF114	4097	1821,1923	1,4461	0,0148	2,00E-04
HSD17B12	24955	1826,2538	1,4702	0,0149	2,00E-04
FBXO6	9268	1829,6715	1,5205	0,015	2,00E-04
EIF2B2	8178	1827,8083	1,4311	0,015	2,00E-04
CMTM7	5849	1829,4358	1,4198	0,015	2,00E-04
CD83	5017	1837,6139	1,5814	0,0152	2,00E-04
HSD17B10	24952	1835,9918	1,4704	0,0152	2,00E-04
ATP6V1F	1950	1838,8294	1,4462	0,0153	2,00E-04
INHBE	25584	1845,1151	1,5165	0,0154	2,00E-04
LGALS8	27163	1842,3098	1,4792	0,0154	2,00E-04
STOML2	44334	1844,9633	1,4587	0,0154	2,00E-04
FKBP1A	9510	1851,0456	1,4826	0,0155	2,00E-04
AVEN	2029	1847,8218	1,4826	0,0155	2,00E-04
ACAT2	228	1853,0843	1,5278	0,0156	2,00E-04
ATP5B	1890	1853,5131	1,5071	0,0156	2,00E-04
SLC25A25	42937	1853,5567	1,4936	0,0156	2,00E-04
POLR2H	39367	1855,016	1,4377	0,0156	2,00E-04
PRMT1	39882	1859,9404	1,5068	0,0158	3,00E-04

PARP8	38141	1858,5365	1,4573	0,0158	3,00E-04
C9ORF46	4157	1859,6146	1,4398	0,0158	3,00E-04
SELS	42251	1863,7902	1,4083	0,0159	3,00E-04
C11ORF48	2773	1869,4699	1,4647	0,016	3,00E-04
ILF2	25519	1874,0499	1,4664	0,0161	3,00E-04
ECGF1	8002	1879,5131	1,524	0,0163	3,00E-04
DHCR7	7264	1879,5571	1,5072	0,0163	3,00E-04
MAT2A	34793	1881,7572	1,4471	0,0163	3,00E-04
MRPS15	35693	1889,0137	1,4503	0,0165	3,00E-04
THOC4	45186	1889,789	1,4883	0,0166	3,00E-04
AP1S1	1223	1890,0957	1,4196	0,0166	3,00E-04
ALG3	870	1899,723	1,484	0,0169	3,00E-04
CTSZ	6607	1899,4449	1,4835	0,0169	3,00E-04
RPL37A	41571	1902,4459	1,4523	0,0169	3,00E-04
LRRC42	34223	1899,8753	1,4297	0,0169	3,00E-04
C1ORF163	3321	1905,978	1,4808	0,017	3,00E-04
SLC30A5	43053	1904,2226	1,4245	0,017	3,00E-04
MT1X	35817	1909,7221	1,4743	0,0171	3,00E-04
ALDOC	857	1909,4147	1,4696	0,0172	3,00E-04
C20ORF27	3526	1918,516	1,4451	0,0175	3,00E-04
CHMP2A	5501	1930,6728	1,457	0,0177	3,00E-04
TNFRSF10A	45728	1924,0701	1,4378	0,0177	3,00E-04
NXT1	37187	1934,1359	1,4287	0,0179	3,00E-04
FKBP1A	9512	1936,5951	1,5122	0,018	3,00E-04
FARSA	9134	1940,0036	1,4578	0,018	3,00E-04
PDF	38494	1938,7301	1,4427	0,018	3,00E-04
GNG5	10996	1936,6027	1,4182	0,018	3,00E-04
PCCB	38214	1941,0728	1,5154	0,0181	3,00E-04
LOC652826	32921	1940,9504	1,4908	0,0181	3,00E-04
LOC402221	28510	1942,1256	1,4735	0,0181	3,00E-04
PTTG1	40337	1939,875	1,446	0,0181	3,00E-04
ATF4	1774	1947,7051	1,4721	0,0184	3,00E-04
PTPRE	40303	1947,4355	1,4282	0,0184	3,00E-04
PLEKHB2	39138	1951,6383	1,4576	0,0187	3,00E-04
PDIA3P	38517	1954,512	1,4843	0,0188	3,00E-04
CLPP	5784	1953,8116	1,4269	0,0188	3,00E-04
ATP6V0C	1933	1953,9251	1,3985	0,0188	3,00E-04
IRF4	25690	1959,1986	1,4785	0,0189	3,00E-04
FXYD5	10357	1956,3255	1,4284	0,0189	3,00E-04
C5ORF32	3844	1969,5077	1,5017	0,0193	3,00E-04
BNIP3	2438	1972,65	1,4909	0,0194	3,00E-04
BCKDK	2240	1970,1287	1,4468	0,0194	3,00E-04
ZNF207	48121	1973,5462	1,4435	0,0195	3,00E-04
RNH1	41419	1978,4504	1,4364	0,0197	3,00E-04
PXMP2	40388	1979,6995	1,4064	0,0197	3,00E-04
LOC644330	30116	1979,6651	1,4033	0,0197	3,00E-04
NDUFAB1	36363	1982,2079	1,447	0,0198	3,00E-04

ASS1	1741	1982,3535	1,4432	0,0198	3,00E-04
C6ORF115	3876	1981,8678	1,4229	0,0198	3,00E-04
ARPC4	1592	1988,1873	1,4933	0,0199	3,00E-04
GRWD1	11433	1987,584	1,4419	0,0199	3,00E-04
ADORA3	554	1990,8046	1,4404	0,0201	3,00E-04
ESRRA	8554	1991,6912	1,4354	0,0201	3,00E-04
TMEM185B	45530	1992,3236	1,3952	0,0201	3,00E-04
EXOSC3	8640	1997,8987	1,4919	0,0204	3,00E-04
PSMG1	40143	2006,3948	1,4567	0,0207	4,00E-04
MRPS18B	35698	2006,8014	1,454	0,0207	4,00E-04
TUBG1	46528	2007,0665	1,4539	0,0207	4,00E-04
SLC29A1	43018	2004,2345	1,4518	0,0207	4,00E-04
THOP1	45193	2007,1135	1,4381	0,0207	4,00E-04
AARSD1	49	2004,4675	1,4273	0,0207	4,00E-04
TMEM109	45414	2013,2061	1,4371	0,0209	4,00E-04
RBM18	40812	2013,8938	1,4185	0,0209	4,00E-04
LOC653344	33155	2019,6033	1,5487	0,021	4,00E-04
SNRPB	43579	2016,4792	1,421	0,021	4,00E-04
CHCHD8	5451	2018,8439	1,4176	0,021	4,00E-04
RPLP0	41595	2022,1193	1,4938	0,0211	4,00E-04
SNTB1	43599	2023,9915	1,449	0,0211	4,00E-04
EIF5B	8242	2020,5272	1,4203	0,0211	4,00E-04
CCT7	4894	2029,788	1,4873	0,0212	4,00E-04
PDCD1	38417	2027,9691	1,4383	0,0212	4,00E-04
CCRN4L	4877	2035,4398	1,4704	0,0214	4,00E-04
RPA1	41483	2035,3331	1,4353	0,0214	4,00E-04
SPAG9	43805	2033,7151	1,4298	0,0214	4,00E-04
GSR	11454	2036,4076	1,4151	0,0214	4,00E-04
TRUB2	46249	2034,5003	1,3985	0,0214	4,00E-04
KHSRP	26161	2043,6151	1,4205	0,0216	4,00E-04
ITPKA	25816	2054,5529	1,4842	0,022	4,00E-04
TIMM23	45258	2056,1507	1,4413	0,0221	4,00E-04
UST	47068	2057,0021	1,412	0,0221	4,00E-04
NIT2	36602	2060,0088	1,4608	0,0223	4,00E-04
TFRC	45107	2063,4951	1,4503	0,0223	4,00E-04
C3ORF37	3750	2060,8917	1,4219	0,0223	4,00E-04
USMG5	46985	2062,1749	1,3517	0,0223	4,00E-04
HLA-DRB4	12024	2061,8438	1,3321	0,0223	4,00E-04
TPM2	45922	2062,7732	1,3309	0,0223	4,00E-04
ATF4	1776	2065,2683	1,4653	0,0224	4,00E-04
SNORD48	43561	2068,6678	1,4377	0,0224	4,00E-04
LOC644422	30158	2069,2806	1,4181	0,0224	4,00E-04
GABARAPL1	10411	2079,8455	1,4964	0,0227	4,00E-04
MAPKAP1	34719	2081,2803	1,4737	0,0227	4,00E-04
SMG5	43418	2076,8431	1,4678	0,0227	4,00E-04
SND1	43495	2076,9361	1,4576	0,0227	4,00E-04
ABCF2	139	2077,0212	1,4473	0,0227	4,00E-04

PHPT1	38823	2075,952	1,4421	0,0227	4,00E-04
NUTF2	37162	2077,8324	1,4374	0,0227	4,00E-04
HTATIP2	25056	2080,778	1,4224	0,0227	4,00E-04
WDR18	47374	2086,3846	1,4316	0,0228	4,00E-04
GOLPH4	11056	2082,744	1,412	0,0228	4,00E-04
KIAA0368	26208	2088,8236	1,4454	0,0229	4,00E-04
MCTS1	34922	2088,4731	1,4354	0,0229	4,00E-04
RPP40	41611	2086,103	1,4266	0,0229	4,00E-04
PHB	38739	2095,0405	1,4309	0,0231	4,00E-04
MYO1E	36072	2097,4316	1,4399	0,0232	4,00E-04
GCDH	10627	2098,4459	1,4507	0,0233	4,00E-04
LPL	34121	2102,5762	1,3683	0,0234	4,00E-04
TUBA3D	46504	2106,0928	1,4898	0,0235	4,00E-04
C7ORF24	3999	2109,8605	1,4843	0,0236	4,00E-04
SLC35F2	43095	2108,3448	1,4554	0,0236	4,00E-04
HK2	11997	2118,757	1,4904	0,0239	4,00E-04
GFOD1	10721	2117,283	1,3544	0,0239	4,00E-04
TRIM29	46082	2120,0762	1,4562	0,024	4,00E-04
HERPUD1	11812	2123,9618	1,483	0,0241	4,00E-04
SLC15A3	42818	2123,4549	1,4423	0,0241	4,00E-04
C7ORF20	3992	2123,19	1,4261	0,0241	4,00E-04
DNAJB6	7578	2125,4179	1,422	0,0241	4,00E-04
FLJ38482	9821	2126,3048	1,4141	0,0241	4,00E-04
MAGMAS	34551	2126,1761	1,3883	0,0241	4,00E-04
CTLA4	6544	2130,5379	1,181	0,0243	4,00E-04
CENPN	5305	2135,0722	1,4453	0,0244	5,00E-04
PDIA4	38518	2132,667	1,4287	0,0244	5,00E-04
TTC27	46393	2131,4884	1,3828	0,0244	5,00E-04
PINX1	38931	2131,2613	1,3756	0,0244	5,00E-04
MGAT4B	35126	2136,8313	1,4219	0,0245	5,00E-04
MTHFD1	35844	2137,2663	1,3993	0,0245	5,00E-04
HS.14706	13275	2142,7151	1,4005	0,0247	5,00E-04
TPM2	45924	2141,5544	1,3347	0,0247	5,00E-04
SNX8	43654	2146,0792	1,5086	0,0249	5,00E-04
PTRH2	40332	2154,7632	1,4224	0,0252	5,00E-04
LY96	34376	2154,9066	1,4215	0,0252	5,00E-04
NDUFA4L2	36358	2157,7814	1,5116	0,0254	5,00E-04
HCST	11728	2159,8325	1,3494	0,0254	5,00E-04
POLR3B	39387	2161,6521	1,4927	0,0255	5,00E-04
CEBPG	5271	2164,0089	1,4557	0,0255	5,00E-04
FAM98A	9099	2165,243	1,4359	0,0255	5,00E-04
LOC162073	27541	2163,38	1,4356	0,0255	5,00E-04
G6PD	10397	2162,2623	1,4204	0,0255	5,00E-04
FADD	8723	2165,7551	1,4207	0,0256	5,00E-04
CCT7	4893	2170,5259	1,4393	0,0258	5,00E-04
TSR1	46358	2169,2129	1,4211	0,0258	5,00E-04
PHYH	38828	2175,3011	1,4418	0,026	5,00E-04

SUPV3L1	44464	2176,9965	1,3994	0,026	5,00E-04
CCT3	4882	2176,5019	1,385	0,026	5,00E-04
LOC143666	27442	2179,7704	1,4864	0,0261	5,00E-04
C16ORF35	3044	2182,0573	1,4398	0,0262	5,00E-04
MAPKAP1	34721	2187,4397	1,4251	0,0264	5,00E-04
HERPUD1	11811	2191,9777	1,4493	0,0265	5,00E-04
LOC643287	29627	2191,7957	1,4431	0,0266	5,00E-04
HEATR1	11763	2193,2206	1,4154	0,0266	5,00E-04
SRD5A1	44036	2194,1916	1,4048	0,0266	5,00E-04
LOC731314	33968	2201,2289	1,3811	0,027	5,00E-04
CHMP4A	5505	2210,3511	1,44	0,0274	5,00E-04
HMBS	12049	2211,1494	1,4153	0,0274	5,00E-04
LOC643668	29791	2219,4787	1,4814	0,0278	5,00E-04
TNFSF4	45778	2220,3973	1,4498	0,0278	5,00E-04
TIMM23	45260	2218,354	1,4468	0,0278	5,00E-04
TRIP13	46178	2219,5753	1,4163	0,0278	5,00E-04
P2RX7	37942	2227,0104	1,4516	0,0282	5,00E-04
SSSCA1	44120	2226,0923	1,4279	0,0282	5,00E-04
MCAT	34875	2229,3479	1,4109	0,0283	5,00E-04
CDK2AP2	5167	2233,6265	1,4273	0,0285	6,00E-04
DUSP10	7858	2234,632	1,4137	0,0286	6,00E-04
ARHGDIA	1455	2237,4504	1,4006	0,0286	6,00E-04
PGD	38695	2238,7766	1,4958	0,0287	6,00E-04
PSMC4	40105	2236,7647	1,4864	0,0287	6,00E-04
HLA-DRB3	12023	2235,7208	1,4711	0,0287	6,00E-04
LARP1	26987	2238,2247	1,4296	0,0287	6,00E-04
RAD23A	40586	2237,2563	1,4275	0,0287	6,00E-04
LSS	34317	2241,8143	1,4396	0,0288	6,00E-04
C2ORF47	3689	2243,7115	1,4274	0,0288	6,00E-04
CCT6A	4887	2242,8849	1,4041	0,0288	6,00E-04
TIMM23	45259	2246,4902	1,4252	0,0289	6,00E-04
HADH2	11639	2244,5906	1,409	0,0289	6,00E-04
ANAPC11	1003	2249,0674	1,4162	0,029	6,00E-04
KLK4	26671	2253,4236	1,571	0,0292	6,00E-04
MGAT1	35117	2259,4174	1,4103	0,0295	6,00E-04
LOC644330	30115	2261,3826	1,4048	0,0295	6,00E-04
SEC61G	42227	2265,1059	1,3936	0,0297	6,00E-04
C12ORF49	2849	2268,2073	1,4362	0,0298	6,00E-04
HNRNPA1	12109	2271,5373	1,4428	0,03	6,00E-04
C6ORF89	3982	2272,8425	1,3955	0,03	6,00E-04
HS.538259	17211	2273,8782	1,4192	0,0301	6,00E-04
RER1	41001	2279,2545	1,4036	0,0302	6,00E-04
PSMD4	40127	2276,3591	1,4027	0,0302	6,00E-04
RNMTL1	41421	2277,445	1,3922	0,0302	6,00E-04
DTD1	7823	2280,1685	1,3833	0,0303	6,00E-04
ATP6V1H	1957	2281,8812	1,3819	0,0303	6,00E-04
SEPHS2	42303	2284,7085	1,3645	0,0304	6,00E-04

RCL1	40921	2288,9634	1,4026	0,0305	6,00E-04
CALU	4338	2284,6323	1,3841	0,0305	6,00E-04
SLC35F2	43096	2291,6734	1,4392	0,0306	6,00E-04
LOC646849	31064	2291,1639	1,4338	0,0306	6,00E-04
PRDM13	39740	2288,7902	1,3749	0,0306	6,00E-04
MYL2	36033	2293,8302	1,4805	0,0307	6,00E-04
G3BP1	10385	2295,489	1,4577	0,0307	6,00E-04
ACOT9	280	2294,0545	1,4549	0,0307	6,00E-04
RIOK1	41233	2294,638	1,4128	0,0307	6,00E-04
TBL3	44823	2297,9701	1,4272	0,0308	6,00E-04
NFKBIB	36521	2300,767	1,3869	0,0308	6,00E-04
HEG1	11782	2300,6377	1,3788	0,0308	6,00E-04
ABI2	179	2299,1694	1,3748	0,0308	6,00E-04
MID1IP1	35316	2302,2517	1,5014	0,0309	6,00E-04
ATIC	1808	2302,7983	1,4113	0,0309	6,00E-04
RPS6KB2	41686	2302,4812	1,3662	0,0309	6,00E-04
C7ORF50	4024	2306,3	1,4124	0,031	6,00E-04
COX7B	6138	2313,562	1,4365	0,0312	6,00E-04
GSS	11455	2312,2278	1,4301	0,0312	6,00E-04
CD97	5035	2312,7802	1,4011	0,0312	6,00E-04
HS.563053	21202	2314,9268	1,393	0,0313	6,00E-04
CLTA	5800	2316,649	1,3896	0,0313	6,00E-04
HS.10862	12336	2321,2167	1,5858	0,0315	6,00E-04
DPH3	7704	2320,5562	1,414	0,0315	6,00E-04
PSMB6	40089	2321,7888	1,4078	0,0315	6,00E-04
BATF	2171	2324,7212	1,493	0,0316	6,00E-04
LOC652493	32727	2326,6959	0,7551	0,0317	6,00E-04
DHX33	7306	2331,0201	1,3825	0,0319	7,00E-04
CAD	4297	2334,0805	1,3902	0,032	7,00E-04
DNAJB11	7557	2338,8319	1,4178	0,0322	7,00E-04
SNHG4	43507	2346,0471	1,3971	0,0327	7,00E-04
RBM18	40811	2355,0341	1,3544	0,0332	7,00E-04
SEC11C	42184	2358,1142	1,4194	0,0333	7,00E-04
MRPL47	35672	2358,6875	1,4121	0,0333	7,00E-04
XPO5	47674	2361,5078	1,409	0,0333	7,00E-04
NUBP1	37064	2362,0697	1,3975	0,0333	7,00E-04
CTSC	6588	2357,1314	1,3918	0,0333	7,00E-04
AXUD1	2042	2362,6861	1,383	0,0333	7,00E-04
C20ORF45	3535	2359,9645	1,3523	0,0333	7,00E-04
DOK3	7677	2366,3479	1,4029	0,0334	7,00E-04
BHLHB2	2339	2370,7926	1,5061	0,0335	7,00E-04
LOC401115	28427	2372,1646	1,4628	0,0335	7,00E-04
COX5A	6127	2371,2978	1,4388	0,0335	7,00E-04
FAM96B	9098	2370,3759	1,3844	0,0336	7,00E-04
SNUPN	43604	2370,5307	1,3831	0,0336	7,00E-04
IFITM2	25219	2378,2321	1,4133	0,0337	7,00E-04
SEMA4A	42265	2378,9771	1,375	0,0337	7,00E-04

TUBA1A	46499	2377,2351	1,485	0,0338	7,00E-04
PLAGL2	39074	2377,8903	1,4159	0,0338	7,00E-04
DNASE2	7621	2381,6466	1,4027	0,0338	7,00E-04
MT1A	35805	2381,0026	1,3478	0,0338	7,00E-04
GOT2	11072	2386,5635	1,4632	0,034	7,00E-04
TCEB1	44893	2385,022	1,3809	0,034	7,00E-04
GATAD2A	10592	2395,0514	1,3939	0,0345	7,00E-04
SLC39A1	43127	2397,3741	1,3784	0,0345	7,00E-04
ILVBL	25528	2396,6526	1,3698	0,0345	7,00E-04
LRMP	34154	2396,5835	1,4011	0,0346	7,00E-04
PNO1	39289	2403,8647	1,3999	0,0348	7,00E-04
LOC723972	33535	2402,7096	1,3989	0,0348	7,00E-04
IMPAD1	25542	2404,6082	1,3801	0,0348	7,00E-04
NAB2	36131	2405,7678	1,3605	0,0349	7,00E-04
TXNDC17	46580	2409,7081	1,3957	0,0351	7,00E-04
POLR2F	39365	2413,0191	1,3748	0,0353	7,00E-04
MRPS12	35690	2414,5126	1,3914	0,0354	7,00E-04
GBE1	10605	2417,6185	1,397	0,0355	8,00E-04
SLCO4A1	43314	2417,4895	1,3961	0,0355	8,00E-04
ATP6V0A2	1924	2421,1797	1,4413	0,0356	8,00E-04
CD151	4900	2420,7716	1,3657	0,0356	8,00E-04
MEMO1	35024	2422,1201	1,3658	0,0357	8,00E-04
GEMIN4	10705	2424,5285	1,3964	0,0358	8,00E-04
GBA	10598	2425,1163	1,3876	0,0358	8,00E-04
NCBP1	36284	2427,9425	1,3533	0,0358	8,00E-04
ADSL	579	2427,7865	1,4468	0,0359	8,00E-04
RPL7L1	41587	2433,9002	1,431	0,0361	8,00E-04
AHSA1	693	2436,8633	1,4527	0,0362	8,00E-04
ARF4	1383	2435,4617	1,3932	0,0362	8,00E-04
ATF3	1771	2440,4787	1,4146	0,0364	8,00E-04
EIF3I	8202	2440,0891	1,4086	0,0364	8,00E-04
ZSCAN2	48760	2441,2347	1,3805	0,0364	8,00E-04
BANF1	2147	2444,4433	1,4343	0,0366	8,00E-04
BUD31	2619	2445,7974	1,4035	0,0366	8,00E-04
SEC24D	42217	2446,154	1,3638	0,0366	8,00E-04
PVT1	40375	2450,0197	1,4027	0,0367	8,00E-04
VEGFA	47169	2453,885	1,3824	0,0368	8,00E-04
SPCS3	43866	2454,9352	1,3592	0,0368	8,00E-04
DNAJC3	7600	2453,5511	1,3563	0,0368	8,00E-04
YWHAB	47749	2453,2043	1,3709	0,0369	8,00E-04
HRAS	12258	2457,5008	1,3571	0,0369	8,00E-04
BNIP1	2436	2464,0764	1,4289	0,037	8,00E-04
CD97	5037	2461,7468	1,4209	0,037	8,00E-04
TNPO2	45811	2461,9489	1,404	0,037	8,00E-04
ALG8	876	2460,4718	1,3541	0,037	8,00E-04
DARS2	6906	2461,239	1,3466	0,037	8,00E-04
PRKAG1	39821	2464,0029	1,4475	0,0371	8,00E-04

SNORA64	43519	2465,7657	1,4074	0,0371	8,00E-04
PIM3	38926	2471,0201	1,3988	0,0374	8,00E-04
C1ORF43	3381	2471,0379	1,3506	0,0374	8,00E-04
GMPPB	10942	2474,4843	1,4441	0,0375	8,00E-04
HPRT1	12236	2475,1347	1,3915	0,0375	8,00E-04
TMED10P	45383	2476,6921	1,391	0,0376	8,00E-04
C12ORF24	2821	2478,6671	1,4231	0,0377	8,00E-04
HSPA5	25017	2481,9496	1,3803	0,0377	8,00E-04
CTTN	6608	2481,7317	1,4721	0,0378	8,00E-04
DNLZ	7633	2480,7653	1,4137	0,0378	8,00E-04
CSNK2A1P	6434	2481,0527	1,3821	0,0378	8,00E-04
SOX8	43748	2484,783	1,3667	0,0379	8,00E-04
LARP2	26991	2488,739	1,3627	0,0381	8,00E-04
HAVCR2	11677	2488,2381	1,3522	0,0381	8,00E-04
RRAGC	41708	2493,2212	1,3839	0,0383	8,00E-04
IRAK1	25676	2495,4251	1,3923	0,0384	8,00E-04
TCP1	44954	2500,528	1,4027	0,0386	9,00E-04
PHF5A	38787	2504,8558	1,3708	0,0388	9,00E-04
ANAPC11	1000	2506,9745	1,4111	0,0389	9,00E-04
FIBP	9484	2508,1767	1,3701	0,039	9,00E-04
PABPC4	37985	2508,8563	1,3544	0,039	9,00E-04
STK39	44320	2510,8951	1,3285	0,039	9,00E-04
C21ORF70	3623	2515,481	1,4059	0,0391	9,00E-04
HNRPLL	12140	2514,7923	1,3548	0,0391	9,00E-04
CTH	6539	2516,7487	1,3997	0,0392	9,00E-04
TSPAN17	46313	2514,5266	1,3547	0,0392	9,00E-04
RAP1GAP	40671	2522,5663	1,4426	0,0394	9,00E-04
LAP3	26978	2521,1846	1,4078	0,0394	9,00E-04
UBFD1	46738	2524,284	1,3938	0,0394	9,00E-04
NRAS	36938	2522,4178	1,3914	0,0394	9,00E-04
NAT5	36211	2526,1048	1,3874	0,0394	9,00E-04
LOC727761	33550	2523,7857	1,3861	0,0394	9,00E-04
KIAA0241	26184	2525,5578	1,3807	0,0394	9,00E-04
PDHA1	38509	2523,9921	1,358	0,0394	9,00E-04
C6ORF108	3873	2527,2474	1,4704	0,0395	9,00E-04
BTG3	2585	2528,2552	1,4343	0,0395	9,00E-04
DCI	6961	2537,5758	1,3663	0,0401	9,00E-04
TWIST1	46558	2540,0521	1,3908	0,0402	9,00E-04
MRPL3	35640	2542,1628	1,3859	0,0403	9,00E-04
ITPKB	25817	2546,9694	1,3127	0,0405	9,00E-04
HAX1	11680	2546,6983	1,3595	0,0406	9,00E-04
RAB38	40506	2551,1018	1,4923	0,0407	9,00E-04
DPP3	7719	2553,2656	1,4051	0,0408	9,00E-04
RHBDF1	41164	2553,121	1,3947	0,0408	9,00E-04
ACTG1	349	2553,5352	1,3793	0,0408	9,00E-04
WDR55	47440	2556,3079	1,3551	0,0409	9,00E-04
NOL6	36748	2564,9695	1,4264	0,0412	9,00E-04

PDIA5	38519	2563,4435	1,3894	0,0412	9,00E-04
CARS	4432	2561,814	1,384	0,0412	9,00E-04
EIF1AY	8169	2562,6627	1,032	0,0412	9,00E-04
PTCD1	40165	2568,4711	1,3761	0,0413	9,00E-04
ME2	34948	2565,8223	1,3759	0,0413	9,00E-04
CAMTA2	4371	2566,5692	1,3752	0,0413	9,00E-04
CCDC58	4695	2568,4062	1,3688	0,0413	9,00E-04
HK1	11993	2561,7755	1,3599	0,0413	9,00E-04
PPAT	39490	2568,9908	1,3498	0,0413	9,00E-04
PFKFB4	38654	2570,1126	1,4356	0,0414	0,001
PSMC1	40098	2570,9188	1,3856	0,0414	0,001
C6ORF66	3973	2575,6584	1,341	0,0416	0,001
C3ORF31	3740	2578,4446	1,3542	0,0417	0,001
CCT3	4883	2581,0182	1,4153	0,0418	0,001
SLC29A1	43019	2580,2478	1,3672	0,0418	0,001
PSMC4	40106	2584,7006	1,4377	0,0419	0,001
NUDC	37070	2583,5682	1,3949	0,0419	0,001
TXNRD1	46601	2589,152	1,4117	0,0422	0,001
DPP3	7718	2591,7983	1,4036	0,0422	0,001
TFB2M	45078	2590,4817	1,403	0,0422	0,001
SEH1L	42234	2588,7651	1,3762	0,0422	0,001
ARF1	1379	2593,3542	1,3478	0,0423	0,001
C18ORF1	3151	2591,561	1,3364	0,0423	0,001
SURF6	44468	2598,1377	1,3817	0,0424	0,001
PSMD11	40114	2597,9024	1,3588	0,0424	0,001
PTTG1	40336	2600,84	1,3582	0,0426	0,001
MID1IP1	35317	2611,4991	1,437	0,0431	0,001
NINJ1	36586	2609,1131	1,3484	0,0431	0,001
AMDHD2	948	2612,4462	1,3899	0,0432	0,001
LOC642489	29278	2614,814	1,4209	0,0433	0,001
POLA2	39328	2615,1095	1,3902	0,0433	0,001
MED19	34970	2625,1786	1,3924	0,0438	0,001
TSEN2	46270	2623,3844	1,3672	0,0438	0,001
GTF2IRD1	11522	2628,4896	1,394	0,0439	0,001
ATP1A1	1843	2625,0775	1,3834	0,0439	0,001
TBC1D13	44764	2627,0109	1,3677	0,0439	0,001
WDR40A	47411	2627,405	1,3549	0,0439	0,001
CLIC4	5746	2628,2542	1,4126	0,044	0,001
HARS	11666	2634,5392	1,3705	0,0443	0,001
RPA1	41482	2634,9389	1,3446	0,0443	0,001
SGPP2	42556	2640,8286	1,4146	0,0445	0,0011
EIF3B	8194	2638,8844	1,3824	0,0445	0,0011
TUBB4Q	46519	2640,1132	1,3803	0,0445	0,0011
METRNL	35054	2645,7197	1,3574	0,0448	0,0011
GUK1	11573	2648,2558	1,3536	0,0449	0,0011
PRNPIP	39896	2649,9665	1,3703	0,045	0,0011
DLK2	7476	2652,1448	1,3764	0,0451	0,0011

C12ORF11	2818	2656,4639	1,4186	0,0453	0,0011
ATPBD1B	1977	2661,0834	1,4035	0,0454	0,0011
LOC653147	33039	2659,6187	1,3869	0,0454	0,0011
BCAT2	2227	2659,2854	1,3671	0,0454	0,0011
HNRNPA3	12111	2657,8755	1,3666	0,0454	0,0011
PIGR	38880	2662,0225	1,3557	0,0455	0,0011
POLR1C	39355	2672,5101	1,3756	0,0463	0,0011
FECH	9357	2672,3477	1,3622	0,0463	0,0011
BSG	2547	2678,2111	1,3627	0,0465	0,0011
CISD1	5613	2680,3695	1,369	0,0467	0,0011
LOC646567	30967	2683,9744	1,3335	0,0469	0,0011
GOSR2	11068	2687,1747	1,4046	0,0471	0,0011
BXDC2	2624	2689,299	1,3403	0,0472	0,0011
ITPA	25813	2691,3309	1,359	0,0474	0,0011
CCT6A	4886	2693,8807	1,3912	0,0475	0,0011
FLJ20699	9643	2700,4885	1,3771	0,0481	0,0012
ITPA	25814	2701,5972	1,356	0,0482	0,0012
AURKAIP1	2022	2704,0825	1,3358	0,0483	0,0012
CD276	4938	2705,6345	1,3899	0,0484	0,0012
LOC647450	31278	2705,5774	0,7713	0,0484	0,0012
RPL32	41555	2707,3562	1,33	0,0485	0,0012
CTSD	6590	2713,3766	1,4034	0,0487	0,0012
SGTA	42564	2711,7929	1,3433	0,0487	0,0012
MTMR14	35867	2717,0447	1,4081	0,049	0,0012
LOC654244	33519	2721,9658	1,3846	0,0491	0,0012
LOC649143	31753	2720,9701	1,2981	0,0491	0,0012
RANBP1	40649	2723,9312	1,4139	0,0492	0,0012
PSMD13	40120	2723,6003	1,3702	0,0492	0,0012
CHCHD1	5443	2721,7409	1,352	0,0492	0,0012
RPS4Y1	41666	2724,3929	0,852	0,0492	0,0012
MRPS16	35694	2729,309	1,3398	0,0495	0,0012
CCT7	4892	2731,1374	1,3764	0,0496	0,0012
EIF3A	8191	2732,6293	1,3499	0,0497	0,0012

RP analysis 24h **Downregulated**

	gene.index	RP/Rsum	FC:(class1/class2)	pfp	P.value
LTB	34325	47,4189	0,1789	0	0
VPREB3	47227	38,7515	0,2209	0	0
CD24	4929	51,6218	0,2269	0	0
LTB	34326	115,8455	0,254	0	0
TXNIP	46594	80,9778	0,2759	0	0
CECR1	5274	60,9348	0,2906	0	0
TMEM71	45624	91,6962	0,3038	0	0
ALOX5	904	79,1002	0,3117	0	0
SNORD13	43532	115,5095	0,3126	0	0
GNG7	10997	92,4924	0,3141	0	0

CXCR4	6673	109,5496	0,3188	0	0
C1ORF162	3320	147,9929	0,3252	0	0
TGFBI	45117	137,6737	0,329	0	0
SPOCK2	43947	113,8165	0,3314	0	0
GPR18	11211	146,7112	0,3381	0	0
C6ORF105	3868	188,867	0,3384	0	0
PYHIN1	40412	156,7867	0,3551	0	0
EVI2B	8598	145,1206	0,3574	0	0
DPEP2	7695	137,7466	0,361	0	0
RNASE6	41280	218,3885	0,3613	0	0
DEF8	7125	133,1259	0,3622	0	0
HS.185764	13839	137,2281	0,3682	0	0
LOC90925	34054	231,6771	0,3718	0	0
LAMA5	26952	140,112	0,3749	0	0
TTC21A	46384	144,1439	0,376	0	0
CXCR4	6671	219,1836	0,3808	0	0
RARRES3	40705	245,1906	0,3862	0	0
ADRB2	573	168,7753	0,3868	0	0
PRICKLE1	39796	251,9518	0,3891	0	0
SELL	42245	241,4765	0,3895	0	0
FCGBP	9307	361,6826	0,3905	0	0
VIPR1	47200	229,1561	0,3906	0	0
NCR3	36325	308,378	0,397	0	0
IFIT2	25213	241,6134	0,3974	0	0
PYHIN1	40411	247,519	0,3997	0	0
TCTN1	44974	241,8374	0,402	0	0
GGA2	10740	188,9379	0,4023	0	0
PDE7B	38483	222,786	0,4054	0	0
LFNG	27149	269,859	0,4068	0	0
NCF1C	36293	218,7784	0,4091	0	0
PRDM8	39756	244,5443	0,4097	0	0
ABCA7	79	198,9142	0,4121	0	0
SLA	42765	219,1355	0,4134	0	0
LOC197135	27556	206,1749	0,4137	0	0
CD37	4963	249,5186	0,4254	0	0
RAB37	40501	242,9386	0,4293	0	0
GALNAC4S-6ST	10505	304,7543	0,4314	0	0
KLF2	26589	242,4214	0,4326	0	0
FAM129C	8833	263,1546	0,4338	0	0
COBLL1	5949	333,6379	0,4361	0	0
PYHIN1	40410	307,8518	0,4364	0	0
ADAM19	412	259,0713	0,4386	0	0
LBH	27026	284,4538	0,4393	0	0
LHPP	27191	284,6578	0,4419	0	0
NCF1	36291	287,3111	0,4436	0	0
CD37	4964	282,5251	0,4451	0	0
SAMD9	41933	301,0022	0,4457	0	0

C10ORF73	2720	285,8668	0,4462	0	0
ZNF831	48710	285,8614	0,4469	0	0
EDG1	8036	309,5595	0,4512	0	0
TEAD2	44999	363,4303	0,4513	0	0
SAMD9L	41934	318,1469	0,4532	0	0
SEMA4B	42266	309,0321	0,4587	0	0
SIGLEC10	42684	362,6766	0,4603	0	0
XAF1	47619	314,7238	0,461	0	0
CTPS2	6573	320,8235	0,4636	0	0
YPEL3	47733	359,6243	0,4822	0	0
RALGPS1	40636	358,5354	0,4867	0	0
C5ORF29	3841	417,0137	0,4438	1,00E-04	0
ITGB7	25791	376,3585	0,4443	1,00E-04	0
ALOX5AP	907	376,7729	0,4603	1,00E-04	0
C16ORF74	3075	390,6551	0,4646	1,00E-04	0
SUSD3	44471	418,8975	0,4654	1,00E-04	0
CDC25B	5068	366,2881	0,4689	1,00E-04	0
SLC44A2	43167	383,7375	0,4724	1,00E-04	0
CROP	6309	370,9537	0,4747	1,00E-04	0
HS.18081	13815	378,1613	0,4799	1,00E-04	0
C6ORF32	3955	417,2722	0,4808	1,00E-04	0
FGR	9468	418,7944	0,4809	1,00E-04	0
EVL	8601	408,8757	0,4823	1,00E-04	0
C9ORF45	4156	400,8686	0,4827	1,00E-04	0
HS.576106	23379	417,5639	0,484	1,00E-04	0
C1ORF63	3397	381,6981	0,4878	1,00E-04	0
PDE7A	38481	424,0082	0,482	2,00E-04	0
SLC9A9	43294	431,1907	0,5006	2,00E-04	0
C5	3828	439,2158	0,47	3,00E-04	0
CHI3L2	5478	445,1957	0,4295	4,00E-04	0
RASGRP2	40731	527,7051	0,4863	4,00E-04	0
BTG2	2584	535,8105	0,5019	4,00E-04	0
C7ORF41	4016	526,6858	0,5073	4,00E-04	0
LOC730256	33881	517,9673	0,5126	4,00E-04	0
OSBPL10	37823	517,6385	0,5131	4,00E-04	0
AMN1	964	536,4682	0,5236	4,00E-04	0
NBPF11	36241	534,0247	0,5305	4,00E-04	0
CEACAM1	5256	506,4032	0,4628	5,00E-04	0
CD24	4930	498,3651	0,4631	5,00E-04	0
RXRA	41845	548,9583	0,4676	5,00E-04	0
SYPL1	44557	486,8173	0,4711	5,00E-04	0
LY86	34372	444,3756	0,4729	5,00E-04	0
LRIG1	34148	469,8208	0,4759	5,00E-04	0
HS.532698	16864	478,4421	0,4846	5,00E-04	0
HLA-DOB	12011	444,8329	0,4939	5,00E-04	0
ZNF266	48175	460,58	0,4943	5,00E-04	0
KLHL14	26622	471,9934	0,4991	5,00E-04	0

KIAA1407	26348	485,1976	0,5002	5,00E-04	0
FAM111A	8786	461,8753	0,5007	5,00E-04	0
TCL1A	44941	475,7847	0,5013	5,00E-04	0
C3ORF34	3744	482,0201	0,5014	5,00E-04	0
ADAM28	420	460,9875	0,5025	5,00E-04	0
FGD3	9399	507,3166	0,5046	5,00E-04	0
MAPK8IP3	34716	453,1232	0,5124	5,00E-04	0
BRWD2	2542	492,329	0,5144	5,00E-04	0
NUAK2	37061	501,8789	0,5157	5,00E-04	0
HERC5	11809	495,8119	0,5161	5,00E-04	0
KIAA0182	26177	503,2465	0,5179	5,00E-04	0
CBX7	4551	503,7572	0,5216	5,00E-04	0
RERE	41002	499,9654	0,5242	5,00E-04	0
TBC1D9	44803	448,609	0,4443	6,00E-04	0
ADD3	518	551,123	0,5081	6,00E-04	0
KIAA1370	26344	554,8002	0,5095	6,00E-04	0
CLK1	5761	561,5687	0,5161	6,00E-04	0
ADAM28	422	551,1618	0,5171	6,00E-04	0
DDX51	7097	566,5732	0,5273	6,00E-04	0
P2RY8	37964	552,5766	0,5321	6,00E-04	0
MS4A1	35733	557,2885	0,5403	6,00E-04	0
HS.478682	16216	552,7766	0,5469	6,00E-04	0
CYSLTR1	6860	569,0732	0,4855	7,00E-04	0
CNTNAP2	5936	582,5906	0,5151	8,00E-04	0
MAP3K1	34635	578,2582	0,5193	8,00E-04	0
MCART1	34870	580,4243	0,5212	8,00E-04	0
PDE4B	38457	576,1283	0,5259	8,00E-04	0
PCMTD1	38367	587,6558	0,5304	8,00E-04	0
ZNF549	48443	581,2444	0,5316	8,00E-04	0
KLF11	26579	588,5212	0,5325	8,00E-04	0
TCF3	44924	583,8282	0,5387	8,00E-04	0
C4ORF34	3813	610,1537	0,5067	9,00E-04	0
LRCH4	34137	593,6651	0,5335	9,00E-04	0
IL16	25385	594,4682	0,5342	9,00E-04	0
NKTR	36621	594,5924	0,5353	9,00E-04	0
TAGLN	44677	608,5286	0,5421	9,00E-04	0
LPP	34123	634,2842	0,5196	0,0011	0
BRDG1	2501	634,4564	0,5247	0,0011	0
NUB1	37063	621,6045	0,5294	0,0011	0
TOP1MT	45854	624,0445	0,5414	0,0011	0
GSDML	11441	636,6741	0,5426	0,0011	0
RASGRP2	40730	643,6889	0,5144	0,0012	0
APOL3	1327	638,9438	0,515	0,0012	0
IL10RA	25361	680,1472	0,5225	0,0012	0
CXXC5	6719	678,4274	0,5287	0,0012	0
GVIN1	11581	639,4854	0,5293	0,0012	0
IFT57	25269	632,5134	0,5326	0,0012	0

PRIC285	39795	677,72	0,5334	0,0012	0
GOLGA8B	11042	667,8816	0,5348	0,0012	0
VEZF1	47180	627,8103	0,5349	0,0012	0
ZNF33B	48262	627,9526	0,5349	0,0012	0
C6ORF192	3932	679,1473	0,5368	0,0012	0
C21ORF24	3578	628,198	0,5435	0,0012	0
MAGT1	34554	680,5923	0,5451	0,0012	0
POFUT1	39314	665,3183	0,5481	0,0012	0
HS.91389	24831	666,374	0,56	0,0012	0
ANXA1	1177	682,0466	0,5077	0,0013	0
SLC7A7	43268	648,3855	0,5086	0,0013	0
PLAC8	39065	659,3905	0,5136	0,0013	0
LOC90586	34050	670,7846	0,5408	0,0013	0
LOC730994	33941	657,9607	0,5425	0,0013	0
FAM111A	8787	674,3316	0,5432	0,0013	0
FCRLA	9346	654,3038	0,5441	0,0013	0
TP53INP1	45890	657,1952	0,5453	0,0013	0
SULT1A1	44413	670,4455	0,5461	0,0013	0
C13ORF18	2873	670,9608	0,5523	0,0013	0
HERC6	11810	671,5896	0,5569	0,0013	0
WNT3	47567	693,3032	0,4923	0,0014	0
TNK2	45786	688,2994	0,5612	0,0014	0
GLIPR1	10863	695,2282	0,5001	0,0015	0
ADAM8	440	695,6812	0,5239	0,0015	0
ARL16	1516	696,1979	0,5309	0,0015	0
PLEKHG1	39145	714,4379	0,5415	0,0015	0
PLCL2	39098	729,0373	0,5434	0,0015	0
TRIM22	46070	723,8492	0,5435	0,0015	0
HYPK	25136	729,1509	0,5535	0,0015	0
C13ORF18	2872	706,3387	0,5539	0,0015	0
DUSP19	7875	715,3471	0,5552	0,0015	0
IL18	25406	707,3751	0,5584	0,0015	0
EBI2	7986	712,5844	0,4783	0,0016	0
PTPRO	40317	705,3441	0,5039	0,0016	0
IGJ	25309	736,4792	0,517	0,0016	0
MYOM1	36093	712,316	0,5293	0,0016	0
LBA1	27023	721,9652	0,533	0,0016	0
TSPAN32	46323	721,8477	0,5344	0,0016	0
PDE4B	38456	729,7662	0,5392	0,0016	0
NPHP3	36842	739,7927	0,5443	0,0016	0
HCG2P7	11710	736,0473	0,5456	0,0016	0
PLEKHA2	39127	709,0649	0,5462	0,0016	0
PRKCE	39841	739,9932	0,5465	0,0016	0
ZNF83	48708	734,9023	0,5537	0,0016	0
LOC441087	28720	697,797	0,5541	0,0016	0
H3F3B	11629	735,4405	0,5556	0,0016	0
CTPS2	6575	711,4931	0,5643	0,0016	0

ZBTB4	47810	710,7343	0,5658	0,0016	0
SH3GLB2	42602	720,3416	0,5669	0,0016	0
ETS1	8576	720,3892	0,5716	0,0016	0
ISG20	25721	785,2054	0,5032	0,0017	0
PIK3IP1	38902	762,5093	0,5204	0,0017	0
HS.539623	17588	776,7645	0,5218	0,0017	0
ATM	1811	760,6822	0,5402	0,0017	0
ABR	198	760,6228	0,5448	0,0017	0
PTPRCAP	40300	790,6574	0,5479	0,0017	0
TP53INP1	45891	745,4116	0,5492	0,0017	0
ZBTB20	47796	808,1667	0,5522	0,0017	0
QSOX2	40437	769,1821	0,5538	0,0017	0
SLC25A28	42946	770,7344	0,5568	0,0017	0
PLEKHG1	39144	763,5715	0,5583	0,0017	0
LOC727820	33566	804,9341	0,5613	0,0017	0
ABCA1	62	759,2879	0,5632	0,0017	0
DMC1	7499	796,6131	0,5651	0,0017	0
LOC728499	33672	754,3613	0,5653	0,0017	0
RBM33	40828	787,4372	0,5689	0,0017	0
ATM	1812	796,4125	0,5712	0,0017	0
TMEM17	45505	800,7531	0,5721	0,0017	0
KIAA0430	26218	794,7701	0,574	0,0017	0
CDKN2D	5214	801,2115	0,5757	0,0017	0
LOC652493	32727	752,741	0,7551	0,0017	0
CXCR3	6670	793,4784	0,5289	0,0018	0
ARHGEF3	1476	824,8146	0,549	0,0018	0
HSPA6	25018	822,9394	0,5519	0,0018	0
E2F5	7969	783,8758	0,5576	0,0018	0
B3GALT4	2062	813,6009	0,5663	0,0018	0
EFHC1	8100	779,8691	0,5663	0,0018	0
ZNF439	48340	781,7603	0,5663	0,0018	0
HS.105618	12321	794,6713	0,5728	0,0018	0
GABBR1	10416	811,009	0,5796	0,0018	0
CDC2L6	5081	820,6605	0,5871	0,0018	0
PTPRO	40316	834,9118	0,5371	0,0019	0
HS.202577	14068	850,0995	0,5468	0,0019	0
DYRK2	7947	846,5314	0,5473	0,0019	0
ZMAT3	48002	827,8721	0,5572	0,0019	0
CD79B	5012	832,9967	0,5612	0,0019	0
CATSPER2	4499	850,3246	0,5632	0,0019	0
PSCD4	40027	849,1563	0,5635	0,0019	0
IFI44L	25206	848,8051	0,564	0,0019	0
GUCY2C	11568	833,6456	0,5649	0,0019	0
NME3	36691	835,5449	0,5663	0,0019	0
EID2B	8161	847,2622	0,5667	0,0019	0
MCM8	34909	849,7441	0,5778	0,0019	0
PCMTD2	38368	848,592	0,578	0,0019	0

TRAF5	45988	819,183	0,5801	0,0019	0
KLF13	26584	834,6927	0,5804	0,0019	0
MGC24039	35166	827,7184	0,5843	0,0019	0
USPL1	47067	851,8199	0,5872	0,0019	0
DCK	6963	856,6514	0,552	0,002	0
SGSM2	42562	864,5909	0,5589	0,002	0
ZBED5	47785	866,262	0,5735	0,002	0
IFFO	25200	857,6609	0,5753	0,002	0
C14ORF24	2964	857,0198	0,5797	0,002	0
ZBTB4	47809	863,6756	0,5879	0,002	0
SSH2	44108	856,6651	0,5951	0,002	0
IL11RA	25366	877,8292	0,5678	0,0021	0
PSMD12	40115	873,7402	0,5679	0,0021	0
ZBED5	47784	871,2064	0,5694	0,0021	0
TIMP2	45267	879,0525	0,5839	0,0021	0
KIAA0999	26284	874,3629	0,5932	0,0021	0
FLJ45244	9968	880,2999	0,5935	0,0021	0
PAN2	38059	878,8658	0,5973	0,0021	0
NCF4	36297	888,4701	0,5657	0,0022	0
ZNF14	48059	886,87	0,5691	0,0022	0
TRAK1	45997	889,5107	0,578	0,0022	0
C6ORF111	3874	879,2948	0,5794	0,0022	0
CBLN3	4529	888,1048	0,5891	0,0022	0
SP110	43757	890,4806	0,5909	0,0022	0
SGCE	42535	888,8427	0,5954	0,0022	0
TMEM107	45412	904,1285	0,5576	0,0023	0
PLAC8	39064	896,3388	0,5652	0,0023	0
CAT	4496	896,1984	0,5672	0,0023	0
KCNH6	25993	908,1981	0,5763	0,0023	0
MBD4	34832	902,5611	0,5787	0,0023	0
ALPP	914	897,7728	0,585	0,0023	0
C5ORF39	3853	901,9171	0,5905	0,0023	0
PNPLA7	39298	912,3181	0,5596	0,0024	0
BCL11A	2245	906,5434	0,5726	0,0024	0
CHRNA5	5552	909,5687	0,5731	0,0024	0
CCDC106	4579	906,6024	0,5909	0,0024	0
PLA2G7	39054	927,6571	0,559	0,0025	0
LOC643031	29517	920,9601	0,569	0,0025	0
CDKN2AIPNL	5206	931,1105	0,5819	0,0025	0
CLK4	5770	919,8496	0,5889	0,0025	0
ADAM28	421	918,8663	0,5945	0,0025	0
WDR33	47394	921,7182	0,5961	0,0025	0
MMP9	35467	934,2122	0,6443	0,0025	0
JUN	25888	948,0736	0,5564	0,0026	0
ADHFE1	530	964,3191	0,5648	0,0026	0
SNORD16	43537	956,8975	0,5664	0,0026	0
TMEM77	45628	940,7301	0,5665	0,0026	0

FCGRT	9324	947,9867	0,5696	0,0026	0
LOC338758	27828	940,6674	0,5708	0,0026	0
GPM6A	11122	956,9545	0,5721	0,0026	0
ARGLU1	1406	928,2397	0,5726	0,0026	0
PKIA	38997	929,0931	0,5731	0,0026	0
CCDC88A	4736	932,105	0,5742	0,0026	0
BTN3A1	2598	946,6473	0,5783	0,0026	0
BBS2	2189	944,6975	0,5792	0,0026	0
AFF3	602	968,1148	0,5809	0,0026	0
NLRP8	36672	933,1459	0,581	0,0026	0
CCDC14	4621	958,2131	0,5826	0,0026	0
SPTLC1	44013	964,9858	0,5827	0,0026	0
FGR	9466	940,1051	0,5834	0,0026	0
TRAF3IP3	45981	950,3658	0,5853	0,0026	0
FKBP14	9506	942,5138	0,5875	0,0026	0
HSD17B7	24964	950,7752	0,5894	0,0026	0
SDHALP1	42158	932,197	0,59	0,0026	0
BACH2	2111	960,8712	0,5901	0,0026	0
SLAMF6	42773	945,896	0,5914	0,0026	0
STX16	44364	947,0828	0,5963	0,0026	0
FCHSD2	9329	951,1266	0,5967	0,0026	0
ZNF615	48525	951,4432	0,5984	0,0026	0
RBL2	40794	957,5449	0,6034	0,0026	0
UBA7	46636	950,481	0,6041	0,0026	0
LOC729446	33801	944,4	0,6072	0,0026	0
SYPL1	44559	965,7952	0,5656	0,0027	0
FAM119A	8799	971,0134	0,5892	0,0027	0
DAPP1	6903	975,8928	0,5832	0,0028	0
GLTSCR2	10908	980,4843	0,6059	0,0028	0
MARCKSL1	34756	984,4341	0,5914	0,0029	0
SLA	42766	984,0961	0,5958	0,0029	0
TNFSF10	45760	990,5175	0,5818	0,003	0
RCP9	40930	989,4909	0,5886	0,003	0
RCSD1	40933	988,12	0,6049	0,003	0
KIAA1370	26343	992,2828	0,5868	0,0031	0
CMIP	5827	997,6028	0,5888	0,0031	0
ADD3	517	1002,1612	0,5893	0,0031	0
NUAK2	37060	1001,6395	0,5965	0,0031	0
NCF1B	36292	1001,5385	0,612	0,0031	0
RN7SK	41264	1000,2322	0,5571	0,0032	0
YPEL2	47732	1009,4353	0,5727	0,0032	0
HSH2D	24989	1001,2538	0,5835	0,0032	0
CREB1	6247	1011,198	0,5886	0,0032	0
VAMP1	47104	1006,6433	0,5898	0,0032	0
LOC645895	30721	1006,6179	0,6019	0,0032	0
UCP2	46798	1017,0674	0,5513	0,0033	0
PAQR8	38098	1024,5094	0,5656	0,0033	0

ZFP36L2	47938	1023,8808	0,5695	0,0033	0
LYSMD2	34414	1029,3014	0,5859	0,0033	0
SEMA3E	42261	1025,7517	0,5927	0,0033	0
APBB3	1265	1024,5578	0,5951	0,0033	0
HS.572649	22943	1024,8862	0,5958	0,0033	0
ZNF69	48609	1029,249	0,5988	0,0033	0
LOC399900	28332	1013,4938	0,5998	0,0033	0
ADA	393	1020,6544	0,6119	0,0033	0
LOC647450	31278	1021,4005	0,7713	0,0033	0
PIM1	38922	1037,781	0,5834	0,0034	0
CDKN1B	5198	1030,3221	0,588	0,0034	0
AMT	978	1039,2432	0,5939	0,0034	0
ADD3	519	1043,6925	0,5951	0,0034	0
CHD9	5466	1041,1566	0,5976	0,0034	0
FKTN	9539	1030,8683	0,6023	0,0034	0
SERTAD2	42418	1037,2944	0,6122	0,0034	0
LTA4H	34324	1052,7357	0,5768	0,0036	0
PFAAP5	38640	1054,2608	0,5836	0,0036	0
HS.535028	16910	1056,46	0,6023	0,0037	0
LAX1	27021	1059,8089	0,5866	0,0038	0
EP400	8385	1063,7682	0,6205	0,0039	0
EBI2	7985	1075,8538	0,5345	0,004	0
PLA2G4B	39044	1072,7071	0,5905	0,004	0
ZNF394	48298	1070,9835	0,5907	0,004	0
SCARNA9	42021	1070,1552	0,5953	0,004	0
ZMYM1	48010	1070,5073	0,599	0,004	0
TTC14	46377	1075,9855	0,5999	0,004	0
CYP27A1	6790	1077,3339	0,6071	0,004	0
PDCD6IP	38430	1079,7228	0,6143	0,004	0
ZBTB34	47804	1075,4436	0,6211	0,004	0
NOD2	36730	1091,5325	0,587	0,0041	0
TBC1D10C	44762	1082,318	0,5898	0,0041	0
XPNPEP3	47670	1085,1973	0,5928	0,0041	0
C8ORF37	4048	1086,1001	0,6033	0,0041	0
SEPW1	42339	1080,7291	0,6166	0,0041	0
KIAA1545	26372	1081,145	0,6193	0,0041	0
SSBP4	44105	1091,6138	0,6199	0,0041	0
ANKRA2	1055	1090,4431	0,5941	0,0042	0
TOMM7	45850	1090,123	0,6078	0,0042	0
CCBE1	4562	1090,0615	0,6103	0,0042	0
ZNF486	48386	1101,8662	0,6086	0,0044	0
LOC401152	28431	1100,2848	0,6149	0,0044	0
C14ORF85	2994	1116,8405	0,6031	0,0045	0
FAM73A	9025	1115,9826	0,6073	0,0045	0
C9ORF80	4186	1109,0546	0,6145	0,0045	0
ZNF154	48067	1118,3233	0,621	0,0045	0
HS.356079	15128	1113,8065	0,6222	0,0045	0

ABCA6	76	1108,7661	0,6493	0,0045	0
KIAA0125	26170	1109,0933	0,6677	0,0045	0
CRBN	6242	1133,7151	0,6077	0,0046	0
LRAP	34128	1130,1694	0,6103	0,0046	0
YPEL5	47735	1130,9147	0,6142	0,0046	0
CD47	4981	1133,1836	0,6171	0,0046	0
HBB	11683	1131,8095	0,7103	0,0046	0
C8ORF45	4055	1137,6638	0,5972	0,0047	0
TTC32	46403	1141,3498	0,6003	0,0047	0
ANKRA2	1056	1141,7207	0,6013	0,0047	0
BCL11A	2246	1145,0862	0,6013	0,0048	0
TMEM50B	45598	1144,8012	0,6186	0,0048	0
CEP110	5329	1143,5533	0,6205	0,0048	0
SLC46A3	43184	1145,3887	0,6313	0,0048	0
TRIB1	46042	1148,9601	0,5983	0,0049	0
ZNF682	48602	1150,4418	0,6039	0,0049	0
COL4A3	6008	1151,3386	0,6045	0,0049	0
FAM39E	8945	1151,7374	0,6142	0,0049	0
RN7SK	41265	1157,9788	0,5944	0,005	0
HSD17B11	24954	1155,5417	0,597	0,005	0
MGC16075	35154	1156,9634	0,5974	0,005	0
KIAA1407	26349	1158,6457	0,6093	0,005	0
DOPEY2	7689	1167,2737	0,6176	0,005	0
PRC1	39727	1167,1844	0,5732	0,0051	0
TYROBP	46610	1165,3952	0,5957	0,0051	0
KIAA1751	26419	1168,9294	0,6051	0,0051	0
TANK	44692	1172,9856	0,6125	0,0051	0
RBM17	40810	1173,5077	0,6194	0,0051	0
HSPC268	25041	1172,0895	0,6259	0,0051	0
PSCD1	40022	1176,1233	0,6325	0,0051	0
HS.544637	19153	1181,5421	0,6123	0,0052	0
PDCD7	38432	1185,9445	0,6253	0,0052	0
GSDML	11439	1180,5791	0,6295	0,0052	0
SPSB3	43998	1185,493	0,621	0,0053	0
FBXO15	9211	1194,357	0,565	0,0054	0
CYP1B1	6778	1193,2992	0,5933	0,0054	0
STAT1	44253	1204,1366	0,5916	0,0055	0
IL10	25359	1207,3971	0,6182	0,0055	0
CASP4	4462	1201,3123	0,6216	0,0055	0
HS.444683	15971	1202,9663	0,632	0,0055	0
RERE	41004	1209,2768	0,6275	0,0056	0
TRIM33	46090	1213,6466	0,6344	0,0056	0
PRKAA1	39807	1212,2372	0,6367	0,0056	0
ANKRD49	1134	1219,5764	0,6166	0,0057	1,00E-04
GRIPAP1	11395	1227,1326	0,62	0,0057	1,00E-04
LOC651309	32358	1227,0717	0,6224	0,0057	1,00E-04
FAM65A	9007	1225,6993	0,6287	0,0057	1,00E-04

LRIG1	34147	1224,7102	0,6407	0,0057	1,00E-04
TMEM80	45631	1235,7892	0,599	0,0058	1,00E-04
ZNF211	48124	1230,0668	0,6124	0,0058	1,00E-04
C14ORF153	2942	1237,998	0,6178	0,0058	1,00E-04
CYP2U1	6816	1232,4998	0,6244	0,0058	1,00E-04
SLAMF6	42772	1234,9168	0,6398	0,0058	1,00E-04
GRAP	11318	1242,1488	0,5935	0,0059	1,00E-04
RAB37	40502	1232,1665	0,6259	0,0059	1,00E-04
PTK2	40220	1243,61	0,6365	0,0059	1,00E-04
AUTS2	2028	1250,7542	0,5993	0,006	1,00E-04
OR13A1	37394	1254,1032	0,6142	0,006	1,00E-04
CEACAM1	5254	1263,4892	0,5912	0,0061	1,00E-04
UNC84B	46906	1261,3622	0,613	0,0061	1,00E-04
PNPT1	39303	1261,5252	0,6201	0,0061	1,00E-04
CD79B	5011	1261,8022	0,6275	0,0061	1,00E-04
JSRP1	25882	1258,4556	0,6276	0,0061	1,00E-04
UBE2J1	46695	1258,8505	0,6303	0,0061	1,00E-04
GORASP1	11063	1266,7551	0,6336	0,0061	1,00E-04
TOP2B	45862	1256,4765	0,6386	0,0061	1,00E-04
SLFN11	43319	1269,4592	0,6096	0,0062	1,00E-04
ATP2A3	1866	1266,6339	0,6279	0,0062	1,00E-04
ADAMTS6	467	1265,97	0,6324	0,0062	1,00E-04
VNN2	47219	1276,7833	0,5966	0,0064	1,00E-04
ANTXR2	1174	1279,7729	0,6194	0,0065	1,00E-04
OCIAD1	37220	1288,5574	0,6245	0,0065	1,00E-04
KLHDC9	26612	1278,7387	0,6371	0,0065	1,00E-04
VAV3	47134	1287,6822	0,6724	0,0065	1,00E-04
IGLL1	25311	1278,7309	0,7586	0,0065	1,00E-04
OMA1	37291	1285,8652	0,6143	0,0066	1,00E-04
HS.556082	20461	1293,2939	0,6177	0,0066	1,00E-04
MEF2C	34996	1295,2343	0,624	0,0066	1,00E-04
EPS15	8450	1293,1497	0,6278	0,0066	1,00E-04
ATP2B4	1878	1292,1333	0,6282	0,0066	1,00E-04
FAM115A	8793	1296,603	0,6311	0,0066	1,00E-04
CDAN1	5046	1285,4828	0,6337	0,0066	1,00E-04
RPS4Y1	41666	1295,7108	0,852	0,0066	1,00E-04
TTC14	46378	1300,8995	0,6231	0,0067	1,00E-04
ARHGAP20	1421	1315,697	0,62	0,0068	1,00E-04
SHCBP1	42636	1315,4599	0,6272	0,0068	1,00E-04
AIRE	717	1316,3613	0,6272	0,0068	1,00E-04
CD79B	5010	1313,7441	0,6305	0,0068	1,00E-04
CREB1	6245	1316,0246	0,6306	0,0068	1,00E-04
C2ORF64	3706	1306,9	0,6315	0,0068	1,00E-04
MGC29891	35181	1314,6765	0,6396	0,0068	1,00E-04
TRABD	45968	1323,8629	0,625	0,0069	1,00E-04
LOC729776	33832	1328,9088	0,6418	0,0069	1,00E-04
BCOR	2291	1325,6118	0,6482	0,0069	1,00E-04

LYST	34419	1327,9978	0,6083	0,007	1,00E-04
ETS1	8577	1328,8945	0,6315	0,007	1,00E-04
FAM39DP	8940	1329,3846	0,6319	0,007	1,00E-04
FAM46C	8964	1333,436	0,621	0,0071	1,00E-04
PARP3	38132	1333,8838	0,6285	0,0071	1,00E-04
TUG1	46541	1332,6792	0,6309	0,0071	1,00E-04
NBPF3	36251	1336,6578	0,6452	0,0071	1,00E-04
RAPGEF3	40682	1342,3004	0,5998	0,0072	1,00E-04
VASH1	47124	1342,1099	0,7368	0,0072	1,00E-04
LOC653086	32991	1347,4684	0,6294	0,0073	1,00E-04
USP24	47014	1346,9467	0,6305	0,0073	1,00E-04
RUNDC2C	41813	1345,9912	0,637	0,0073	1,00E-04
TESC	45037	1358,4088	0,5929	0,0074	1,00E-04
C12ORF35	2832	1357,3927	0,6222	0,0074	1,00E-04
BLZF1	2390	1359,4114	0,6295	0,0074	1,00E-04
PRPF4B	39940	1360,6155	0,6465	0,0074	1,00E-04
MPHOSPH1	35540	1359,9216	0,6119	0,0075	1,00E-04
ST3GAL5	44166	1368,9697	0,6256	0,0075	1,00E-04
ARHGAP24	1429	1368,5432	0,6326	0,0075	1,00E-04
ZNF430	48330	1374,3773	0,6327	0,0076	1,00E-04
KIAA0256	26189	1370,2749	0,636	0,0076	1,00E-04
NSUN5C	37011	1375,6557	0,645	0,0076	1,00E-04
C11ORF80	2813	1377,6517	0,6043	0,0077	1,00E-04
SYNE2	44531	1381,7918	0,6202	0,0077	1,00E-04
TLR6	45321	1380,1263	0,6373	0,0077	1,00E-04
PHF1	38751	1384,1203	0,6486	0,0078	1,00E-04
GSTM2	11472	1389,5682	0,72	0,0078	1,00E-04
TDP1	44984	1394,2238	0,6297	0,0079	1,00E-04
PDE4C	38461	1399,5915	0,6369	0,008	1,00E-04
LMBRD1	27310	1403,7792	0,6084	0,0081	1,00E-04
C14ORF106	2897	1413,2342	0,6109	0,0081	1,00E-04
C4ORF34	3814	1413,3308	0,6506	0,0081	1,00E-04
BLK	2378	1412,3386	0,6157	0,0082	1,00E-04
C1QTNF6	3455	1410,5423	0,6212	0,0082	1,00E-04
LOC221442	27604	1412,3992	0,631	0,0082	1,00E-04
MXD4	35963	1407,061	0,6337	0,0082	1,00E-04
C5ORF25	3837	1415,8755	0,6443	0,0082	1,00E-04
POU2AF1	39435	1416,5488	0,6456	0,0082	1,00E-04
RSBN1	41734	1423,4929	0,6342	0,0083	1,00E-04
LOC132241	27408	1423,2095	0,6457	0,0083	1,00E-04
MTIF3	35855	1428,1294	0,6309	0,0084	1,00E-04
CD46	4980	1426,0512	0,6411	0,0084	1,00E-04
KIAA1683	26407	1431,9357	0,6552	0,0084	1,00E-04
P2RY10	37945	1440,3092	0,617	0,0085	1,00E-04
HS.163084	13661	1438,6965	0,653	0,0085	1,00E-04
HHEX	11855	1444,0778	0,6192	0,0086	1,00E-04
CXCR7	6677	1449,3502	0,646	0,0086	1,00E-04

SLC16A12	42824	1448,7895	0,6469	0,0086	1,00E-04
DGKA	7239	1449,1361	0,6532	0,0086	1,00E-04
DUXAP3	7910	1457,9755	0,6191	0,0087	1,00E-04
BCL3	2271	1461,8299	0,6452	0,0087	1,00E-04
CAPRIN2	4409	1462,4421	0,6642	0,0087	1,00E-04
RTP4	41798	1465,6549	0,6323	0,0088	1,00E-04
PMAIP1	39224	1470,4943	0,6143	0,0089	1,00E-04
CNN3	5885	1475,1387	0,6171	0,009	1,00E-04
MAP1LC3A	34608	1477,3559	0,6491	0,0091	1,00E-04
HS.22689	14306	1481,3541	0,648	0,0093	1,00E-04
ADAM29	423	1491,0254	0,5709	0,0095	1,00E-04
PHIP	38798	1492,0065	0,6423	0,0095	1,00E-04
TMEM106B	45408	1495,8427	0,6425	0,0095	1,00E-04
HS.406106	15516	1496,8113	0,6534	0,0095	1,00E-04
ARL17P1	1518	1488,2665	0,6599	0,0095	1,00E-04
LOC731985	34001	1491,5947	0,6607	0,0095	1,00E-04
GPM6A	11121	1498,6607	0,6245	0,0096	1,00E-04
RASGRP3	40733	1498,9046	0,6414	0,0096	1,00E-04
SPINT2	43933	1513,2044	0,6284	0,0098	1,00E-04
ATHL1	1807	1513,4264	0,631	0,0098	1,00E-04
FHDC1	9470	1510,3033	0,6415	0,0098	1,00E-04
OGT	37264	1519,1319	0,6616	0,0099	1,00E-04
HS.508889	16406	1530,4121	0,6211	0,01	1,00E-04
HS.5724	22923	1524,8921	0,6261	0,01	1,00E-04
CD5	4984	1527,9545	0,628	0,01	1,00E-04
FLJ46309	10010	1521,1869	0,6442	0,01	1,00E-04
FCGR2B	9318	1530,8832	0,651	0,01	1,00E-04
OGT	37265	1524,906	0,652	0,01	1,00E-04
FAM39DP	8943	1529,4391	0,6562	0,01	1,00E-04
LAPTM5	26982	1527,9884	0,6659	0,01	1,00E-04
BIRC3	2365	1531,0847	0,6751	0,01	1,00E-04
PPA2	39460	1533,0047	0,6467	0,0101	1,00E-04
LOC649841	31941	1532,4239	0,653	0,0101	1,00E-04
SMCHD1	43407	1539,1591	0,6435	0,0102	1,00E-04
C5ORF28	3839	1540,7186	0,6499	0,0103	1,00E-04
TOMM7	45849	1539,7438	0,6588	0,0103	1,00E-04
CSAD	6359	1542,3294	0,6645	0,0103	1,00E-04
GGA1	10737	1543,7353	0,6626	0,0104	1,00E-04
CCR6	4866	1551,5724	0,6284	0,0106	1,00E-04
PRKAA1	39808	1555,6054	0,6512	0,0106	1,00E-04
ICAM3	25154	1550,5499	0,6553	0,0106	1,00E-04
HS.193784	13906	1562,2854	0,6216	0,0107	1,00E-04
ZNF451	48353	1557,9215	0,6429	0,0107	1,00E-04
CDC42SE2	5103	1564,5272	0,6483	0,0107	1,00E-04
LOC729603	33815	1563,404	0,6569	0,0107	1,00E-04
FCAR	9298	1560,5575	0,6596	0,0107	1,00E-04
CBX4	4548	1564,1739	0,6597	0,0107	1,00E-04

LOC51149	28913	1557,7542	0,6671	0,0107	1,00E-04
JARID1D	25850	1564,0111	0,782	0,0107	1,00E-04
PAN3	38060	1576,5558	0,6436	0,0108	1,00E-04
CORO1B	6103	1580,6109	0,647	0,0108	1,00E-04
LMOD3	27331	1568,3139	0,6478	0,0108	1,00E-04
SLC44A4	43170	1581,3586	0,6513	0,0108	1,00E-04
GUSBL1	11577	1582,5317	0,6597	0,0108	1,00E-04
ABHD3	169	1574,3846	0,6602	0,0108	1,00E-04
MX2	35960	1574,4739	0,6664	0,0108	1,00E-04
PLEKHA1	39124	1577,6997	0,6672	0,0108	1,00E-04
PTPN22	40274	1574,0079	0,6674	0,0108	1,00E-04
ABCA6	77	1573,0942	0,6742	0,0108	1,00E-04
BCYRN1	2302	1572,6932	0,6534	0,0109	1,00E-04
CWF19L2	6646	1570,9754	0,6537	0,0109	1,00E-04
PBXIP1	38193	1583,0622	0,6577	0,0109	1,00E-04
CBLB	4523	1571,0493	0,6692	0,0109	1,00E-04
FAM53B	8982	1593,0826	0,6365	0,011	1,00E-04
C1ORF176	3332	1598,6065	0,6479	0,011	1,00E-04
PARP3	38133	1590,0446	0,6483	0,011	1,00E-04
C21ORF81	3629	1594,175	0,6494	0,011	1,00E-04
HS.440088	15858	1587,9748	0,6495	0,011	1,00E-04
LILRB1	27229	1590,1787	0,6548	0,011	1,00E-04
INADL	25552	1597,272	0,6607	0,011	1,00E-04
TMEM156	45470	1596,6542	0,662	0,011	1,00E-04
MPPE1	35558	1594,8855	0,666	0,011	1,00E-04
FLJ40142	9848	1597,0666	0,6683	0,011	1,00E-04
TNFRSF13B	45737	1595,6608	0,8016	0,011	1,00E-04
P2RY10	37946	1601,8937	0,634	0,0111	1,00E-04
CCL2	4766	1602,8298	0,6354	0,0111	1,00E-04
CDKN1B	5199	1610,5719	0,6498	0,0111	1,00E-04
HS.436134	15728	1599,7786	0,6582	0,0111	1,00E-04
CUGBP2	6622	1608,0798	0,6605	0,0111	1,00E-04
DYRK1A	7940	1604,2086	0,6638	0,0111	1,00E-04
SNX10	43611	1605,3832	0,6671	0,0111	1,00E-04
SYNJ2BP	44547	1608,3128	0,6742	0,0111	1,00E-04
IFI44	25205	1613,4226	0,6904	0,0112	1,00E-04
TMEM50B	45597	1616,5954	0,6598	0,0113	1,00E-04
APOBEC3F	1305	1625,2937	0,6265	0,0114	1,00E-04
NT5C3	37021	1628,3421	0,6415	0,0115	1,00E-04
RCOR3	40929	1624,3583	0,6503	0,0115	1,00E-04
TNFRSF13C	45738	1633,6661	0,6469	0,0116	1,00E-04
ANKRD30B	1105	1632,0846	0,6492	0,0116	1,00E-04
BCL11A	2244	1630,075	0,6533	0,0116	1,00E-04
RAB3IP	40515	1633,5528	0,6593	0,0116	1,00E-04
CAPRIN2	4408	1630,7881	0,6754	0,0116	1,00E-04
METTL7A	35072	1646,6776	0,6553	0,0117	2,00E-04
KIAA0494	26222	1643,6063	0,6608	0,0117	1,00E-04

LOC649923	31961	1645,4799	0,6637	0,0117	1,00E-04
GRK5	11402	1649,8415	0,6589	0,0119	2,00E-04
DYRK2	7948	1653,4534	0,6538	0,012	2,00E-04
RPS29	41657	1656,9696	0,6755	0,012	2,00E-04
SLC6A16	43234	1667,397	0,6637	0,0124	2,00E-04
LOC652694	32844	1666,5303	0,8685	0,0124	2,00E-04
DIDO1	7345	1668,6977	0,6537	0,0125	2,00E-04
CYFIP2	6752	1668,6081	0,657	0,0125	2,00E-04
GPX1	11299	1674,8938	0,6597	0,0125	2,00E-04
PGCP	38693	1669,6282	0,6626	0,0125	2,00E-04
ATP2B4	1877	1672,1429	0,6682	0,0125	2,00E-04
SPG11	43886	1672,3284	0,6694	0,0125	2,00E-04
CEP192	5342	1671,5673	0,6705	0,0125	2,00E-04
LOC23117	27618	1670,0384	0,6843	0,0125	2,00E-04
C14ORF28	2967	1681,3243	0,6333	0,0126	2,00E-04
ERAP2	8469	1677,3841	0,6487	0,0126	2,00E-04
IFIT3	25215	1685,1627	0,652	0,0126	2,00E-04
RAXL1	40770	1679,3052	0,6549	0,0126	2,00E-04
C5ORF5	3861	1680,0053	0,655	0,0126	2,00E-04
FAM39DP	8941	1681,6819	0,6566	0,0126	2,00E-04
KIAA0240	26183	1685,6814	0,6598	0,0126	2,00E-04
HS.137971	13118	1680,8466	0,6619	0,0126	2,00E-04
C20ORF72	3552	1674,7363	0,6648	0,0126	2,00E-04
HBP1	11691	1674,8152	0,6722	0,0126	2,00E-04
COX19	6123	1680,012	0,6745	0,0126	2,00E-04
P2RX1	37929	1683,5344	0,6821	0,0126	2,00E-04
ALS2CR13	922	1684,7453	0,6404	0,0127	2,00E-04
COL9A3	6037	1692,6329	0,6556	0,0127	2,00E-04
LOC728014	33600	1686,633	0,6782	0,0127	2,00E-04
GSTM1	11470	1691,9358	0,7466	0,0127	2,00E-04
TSC22D3	46265	1694,7152	0,635	0,0128	2,00E-04
FLJ44124	9938	1695,7571	0,6522	0,0128	2,00E-04
BAZ2B	2182	1695,5056	0,6655	0,0128	2,00E-04
HS.413494	15556	1697,7056	0,6646	0,0129	2,00E-04
HS.279842	14636	1699,6778	0,6537	0,013	2,00E-04
ZNF480	48378	1703,9222	0,655	0,013	2,00E-04
ZNF10	48032	1703,9474	0,6637	0,013	2,00E-04
HIVEP1	11989	1700,0355	0,6704	0,013	2,00E-04
FAM89B	9073	1705,6681	0,653	0,0131	2,00E-04
AHR	690	1706,5803	0,6588	0,0131	2,00E-04
MKNK2	35359	1705,8029	0,6708	0,0131	2,00E-04
KNTC1	26710	1705,9969	0,68	0,0131	2,00E-04
PHKB	38804	1712,3111	0,6387	0,0132	2,00E-04
FAM76B	9033	1711,6326	0,6641	0,0132	2,00E-04
SPEN	43882	1709,8835	0,6718	0,0132	2,00E-04
CDC40	5085	1710,2096	0,6743	0,0132	2,00E-04
DUT	7899	1710,2296	0,678	0,0132	2,00E-04

C11ORF35	2758	1721,7711	0,6672	0,0134	2,00E-04
CD27	4935	1732,9702	0,6308	0,0136	2,00E-04
HS.554324	20336	1728,6266	0,633	0,0136	2,00E-04
SIAH1	42673	1728,973	0,6514	0,0136	2,00E-04
PARP12	38125	1728,008	0,6578	0,0136	2,00E-04
IFIT1	25210	1728,6488	0,6598	0,0136	2,00E-04
ETV6	8587	1729,4623	0,6625	0,0136	2,00E-04
STIM2	44290	1727,1891	0,6658	0,0136	2,00E-04
PTPN22	40276	1731,727	0,6673	0,0136	2,00E-04
POLR3GL	39393	1731,1729	0,6674	0,0136	2,00E-04
DTWD2	7834	1733,3876	0,6686	0,0136	2,00E-04
FGD2	9398	1734,9192	0,6738	0,0136	2,00E-04
CRYZL1	6354	1731,167	0,6759	0,0136	2,00E-04
FCRL2	9337	1730,6118	0,7157	0,0136	2,00E-04
TCFL5	44934	1738,4044	0,6542	0,0137	2,00E-04
KMO	26705	1738,0542	0,663	0,0137	2,00E-04
FBXO21	9222	1748,6733	0,6591	0,0141	2,00E-04
AFG3L1	605	1752,0946	0,6741	0,0142	2,00E-04
C5ORF41	3857	1758,976	0,6464	0,0144	2,00E-04
DDX17	7059	1759,5285	0,6627	0,0144	2,00E-04
CDK5RAP3	5181	1761,4301	0,6672	0,0144	2,00E-04
TNRC6B	45821	1760,4999	0,6724	0,0144	2,00E-04
CXCL16	6661	1765,2042	0,6748	0,0144	2,00E-04
SELM	42246	1767,8589	0,6407	0,0145	2,00E-04
HS.143408	13148	1769,3494	0,6494	0,0145	2,00E-04
GNB5	10984	1772,7244	0,6741	0,0145	2,00E-04
DUT	7900	1768,429	0,6769	0,0145	2,00E-04
LOC728888	33730	1771,4295	0,6865	0,0145	2,00E-04
IL1B	25417	1770,3843	0,8648	0,0145	2,00E-04
PTPN22	40277	1774,1036	0,6445	0,0146	2,00E-04
PQLC3	39690	1776,2078	0,6632	0,0146	2,00E-04
DYRK1A	7943	1773,3734	0,6832	0,0146	2,00E-04
HS.542993	18510	1780,173	0,6687	0,0147	2,00E-04
GLYATL2	10920	1781,3244	0,6776	0,0147	2,00E-04
ZDHHC17	47889	1782,1367	0,6802	0,0147	2,00E-04
CTSL1	6601	1784,1798	0,6295	0,0148	2,00E-04
LIPT1	27291	1784,7224	0,6556	0,0148	2,00E-04
DYRK2	7949	1782,3945	0,6767	0,0148	2,00E-04
MON2	35507	1781,0523	0,6858	0,0148	2,00E-04
ASIP	1714	1785,975	0,6556	0,0149	2,00E-04
CLK1	5759	1788,7241	0,6707	0,0149	2,00E-04
AES	587	1790,3515	0,6588	0,015	2,00E-04
IL16	25384	1796,9204	0,6532	0,0152	2,00E-04
CCDC24	4644	1801,5391	0,6621	0,0152	2,00E-04
DNAJB2	7570	1799,7331	0,6686	0,0152	2,00E-04
FAIM3	8744	1804,7424	0,6617	0,0153	2,00E-04
LOC389517	28135	1807,286	0,6773	0,0154	2,00E-04

IL4R	25496	1810,3493	0,6638	0,0155	2,00E-04
ACACB	211	1815,932	0,6552	0,0156	2,00E-04
ARHGAP24	1430	1816,1593	0,6743	0,0156	2,00E-04
MZF1	36114	1817,337	0,6821	0,0157	2,00E-04
USP34	47028	1826,026	0,6812	0,0159	2,00E-04
AK3	726	1829,9974	0,6595	0,016	2,00E-04
ZNF443	48346	1830,5331	0,6663	0,016	2,00E-04
RPS15A	41626	1827,8586	0,6669	0,016	2,00E-04
RPS24	41638	1836,5083	0,6627	0,0162	2,00E-04
BIRC3	2364	1837,1201	0,6648	0,0162	2,00E-04
FCGR2B	9317	1838,3775	0,6683	0,0162	2,00E-04
SEC62	42228	1837,7209	0,6786	0,0162	2,00E-04
VHL	47190	1840,2885	0,6888	0,0162	2,00E-04
MAP4K2	34666	1838,8984	0,6955	0,0162	2,00E-04
HEATR5B	11770	1847,1472	0,6746	0,0165	2,00E-04
ITGB1	25765	1850,9143	0,6577	0,0167	3,00E-04
IGFBP4	25297	1852,6761	0,6675	0,0167	3,00E-04
LOC728014	33601	1851,045	0,6782	0,0167	3,00E-04
MAP4K4	34670	1858,6168	0,6901	0,0169	3,00E-04
FGL2	9465	1863,7425	0,6947	0,0169	3,00E-04
LEF1	27113	1859,6462	0,7263	0,0169	3,00E-04
CD82	5015	1866,8091	0,6464	0,017	3,00E-04
MST1	35793	1863,1261	0,6667	0,017	3,00E-04
RBM5	40851	1863,4459	0,679	0,017	3,00E-04
PNRC2	39305	1866,3083	0,6841	0,017	3,00E-04
C21ORF7	3622	1880,784	0,6379	0,0172	3,00E-04
HS.13291	13001	1876,7228	0,6464	0,0172	3,00E-04
PYCARD	40398	1878,0055	0,6523	0,0172	3,00E-04
HS.163426	13665	1879,9854	0,658	0,0172	3,00E-04
HS.580797	24088	1872,7971	0,6699	0,0172	3,00E-04
STAG3L1	44217	1872,6887	0,6812	0,0172	3,00E-04
BANK1	2149	1877,2521	0,6849	0,0172	3,00E-04
B4GALT1	2086	1875,7616	0,6928	0,0172	3,00E-04
GPBP1	11094	1884,8406	0,682	0,0173	3,00E-04
ZNF550	48444	1884,6718	0,6873	0,0173	3,00E-04
ZNF342	48265	1882,8888	0,6877	0,0173	3,00E-04
HS.4988	16347	1889,2699	0,6632	0,0175	3,00E-04
ALDH3A2	831	1896,0824	0,6628	0,0176	3,00E-04
SFRS5	42504	1895,9794	0,6672	0,0176	3,00E-04
UPF3A	46935	1893,9535	0,6741	0,0176	3,00E-04
HS.561844	20991	1903,1346	0,6826	0,0178	3,00E-04
PNN	39288	1905,1115	0,6757	0,0179	3,00E-04
STAG3L2	44220	1918,7676	0,6805	0,0183	3,00E-04
ZNF230	48146	1914,2487	0,6807	0,0183	3,00E-04
SPG7	43897	1917,7323	0,6853	0,0183	3,00E-04
RDH5	40945	1914,8454	0,6974	0,0183	3,00E-04
LOC648470	31565	1922,9462	0,6677	0,0184	3,00E-04

HS.550293	19920	1919,977	0,6746	0,0184	3,00E-04
KIAA0040	26163	1926,2697	0,6747	0,0184	3,00E-04
CNN2	5884	1924,6089	0,6783	0,0184	3,00E-04
HS.542027	18238	1925,5663	0,6809	0,0184	3,00E-04
PHACS	38730	1924,6751	0,6817	0,0184	3,00E-04
NCF4	36296	1921,8528	0,6911	0,0184	3,00E-04
LOC648405	31552	1929,0268	0,703	0,0185	3,00E-04
C8ORF70	4070	1932,6155	0,6629	0,0186	3,00E-04
HS.572538	22933	1934,1213	0,6947	0,0186	3,00E-04
NKTR	36620	1936,6732	0,6831	0,0187	3,00E-04
TPD52	45902	1939,2379	0,6542	0,0188	3,00E-04
ARHGEF6	1480	1943,626	0,6656	0,0188	3,00E-04
ELF2	8261	1941,9669	0,6672	0,0188	3,00E-04
LAG3	26934	1941,4271	0,7386	0,0188	3,00E-04
TRRAP	46245	1944,9707	0,6856	0,0189	3,00E-04
DHRS1	7277	1948,5821	0,684	0,019	3,00E-04
FIG4	9486	1946,3353	0,6864	0,019	3,00E-04
CHKB	5491	1952,5664	0,6791	0,0191	3,00E-04
AGXT2L2	677	1950,1134	0,6901	0,0191	3,00E-04
XAF1	47620	1954,6682	0,6597	0,0192	3,00E-04
SIDT2	42679	1961,2234	0,6548	0,0193	3,00E-04
MYO5C	36081	1964,2286	0,662	0,0193	3,00E-04
EPSTI1	8462	1964,9599	0,6653	0,0193	3,00E-04
JMJD1C	25857	1958,9878	0,6714	0,0193	3,00E-04
SMAD5	43364	1956,6167	0,674	0,0193	3,00E-04
DDR1	7035	1964,9609	0,6795	0,0193	3,00E-04
CDK5RAP2	5177	1957,5864	0,6875	0,0193	3,00E-04
MGC39372	35208	1962,2784	0,6913	0,0193	3,00E-04
PFDN5	38646	1971,204	0,6626	0,0195	3,00E-04
CD55	4988	1970,9369	0,6644	0,0195	3,00E-04
RPL17	41522	1973,5538	0,6701	0,0195	3,00E-04
SERPINF1	42409	1971,5999	0,6768	0,0195	3,00E-04
ARHGAP24	1427	1979,2628	0,6862	0,0196	3,00E-04
ZNF217	48130	1977,5848	0,6865	0,0196	3,00E-04
LOC643977	29927	1980,38	0,6947	0,0196	3,00E-04
NASP	36199	1975,8592	0,7004	0,0196	3,00E-04
RAB6IP1	40537	1978,6789	0,7023	0,0196	3,00E-04
GBP4	10613	1978,0659	0,7377	0,0196	3,00E-04
IKZF1	25351	1985,1213	0,6772	0,0197	3,00E-04
FNBP4	10078	1985,4514	0,6812	0,0197	3,00E-04
LUC7L	34348	1982,0671	0,6892	0,0197	3,00E-04
CSF2RA	6383	1991,4124	0,672	0,0199	3,00E-04
SP3	43765	1991,9792	0,6836	0,0199	3,00E-04
FAM98C	9103	1992,0896	0,6887	0,0199	3,00E-04
DDX60	7109	1991,37	0,6895	0,0199	3,00E-04
DDX58	7104	1995,278	0,6955	0,0199	3,00E-04
CTDSP2	6524	1996,3027	0,704	0,0199	3,00E-04

HNRPDL	12132	2002,3391	0,673	0,02	3,00E-04
SLC2A5	43038	2002,9901	0,6853	0,02	3,00E-04
C15ORF48	3026	2002,704	0,6864	0,02	3,00E-04
NSUN5	37005	2005,1405	0,6834	0,0201	3,00E-04
ZNF540	48432	2001,7153	0,6968	0,0201	3,00E-04
XPC	47666	2008,7061	0,6896	0,0202	3,00E-04
FAM46A	8962	2007,7768	0,7761	0,0202	3,00E-04
ZNF518B	48415	2011,7191	0,6793	0,0203	3,00E-04
UNC84A	46905	2010,9949	0,686	0,0203	3,00E-04
LOC400027	28341	2022,6216	0,6843	0,0207	3,00E-04
IFNGR2	25252	2022,8754	0,6942	0,0207	3,00E-04
CHCHD7	5450	2020,6707	0,6976	0,0207	3,00E-04
ZNF223	48136	2023,8413	0,6893	0,0208	4,00E-04
AHSA2	694	2026,3757	0,6955	0,0208	4,00E-04
SMARCB1	43382	2027,9682	0,6833	0,0209	4,00E-04
PDLIM1	38527	2029,4905	0,6958	0,0209	4,00E-04
BCL11A	2243	2034,5427	0,6768	0,021	4,00E-04
ZNF266	48176	2038,1723	0,6782	0,0211	4,00E-04
USP49	47052	2040,7886	0,6774	0,0212	4,00E-04
LEF1	27114	2044,2786	0,7281	0,0213	4,00E-04
C9ORF103	4090	2048,7325	0,6828	0,0214	4,00E-04
TMEM175	45511	2048,1998	0,6922	0,0214	4,00E-04
KLF11	26578	2052,9074	0,6907	0,0216	4,00E-04
TAF1C	44654	2063,9842	0,6906	0,0221	4,00E-04
PUM2	40346	2066,7194	0,6853	0,0223	4,00E-04
TMEM80	45632	2071,6041	0,6635	0,0224	4,00E-04
FCGR2B	9316	2074,4852	0,6774	0,0225	4,00E-04
HS.158923	13580	2071,4597	0,6833	0,0225	4,00E-04
LOC54103	28917	2071,071	0,7013	0,0225	4,00E-04
HS.572642	22940	2078,1815	0,6989	0,0226	4,00E-04
MTSS1	35900	2080,067	0,6711	0,0227	4,00E-04
ZNF189	48099	2080,4351	0,7096	0,0227	4,00E-04
STAT1	44254	2083,6794	0,6825	0,0228	4,00E-04
DUSP22	7881	2088,2317	0,6732	0,023	4,00E-04
BTN3A3	2601	2092,4628	0,6793	0,023	4,00E-04
DIP2C	7362	2088,8161	0,6828	0,023	4,00E-04
ITPR3	25821	2091,2938	0,6869	0,023	4,00E-04
ZNF91	48713	2094,4429	0,687	0,023	4,00E-04
SPG3A	43893	2093,2939	0,6947	0,023	4,00E-04
ARRDC5	1618	2090,8343	0,7253	0,023	4,00E-04
FMOD	10067	2092,102	0,7562	0,023	4,00E-04
CCDC109B	4585	2096,3903	0,6714	0,0231	4,00E-04
CHPT1	5525	2098,3392	0,6823	0,0231	4,00E-04
SETBP1	42428	2102,6346	0,6768	0,0233	4,00E-04
SYK	44515	2107,65	0,6894	0,0235	4,00E-04
HS.126768	12656	2115,1778	0,6944	0,0238	4,00E-04
IL8	25511	2113,3653	0,7337	0,0238	4,00E-04

ITGB1	25767	2117,8491	0,681	0,0239	4,00E-04
ARL6IP5	1545	2118,3634	0,6909	0,0239	4,00E-04
HS.545615	19499	2115,0813	0,7034	0,0239	4,00E-04
ARRDC2	1613	2118,7528	0,6959	0,024	4,00E-04
C5ORF41	3858	2124,0127	0,6662	0,0241	4,00E-04
PILRB	38919	2124,169	0,6947	0,0241	4,00E-04
C1ORF186	3342	2127,1692	0,6956	0,0242	4,00E-04
SERPINI1	42415	2129,504	0,6712	0,0243	4,00E-04
SYTL2	44593	2134,0379	0,6718	0,0244	4,00E-04
SH3BGRL	42586	2133,477	0,688	0,0244	4,00E-04
RUNX1	41818	2131,1432	0,7122	0,0244	4,00E-04
CLSTN1	5793	2137,4289	0,6793	0,0245	4,00E-04
ZNF302	48221	2136,4674	0,6944	0,0245	4,00E-04
HS.231861	14315	2136,4799	0,6954	0,0245	4,00E-04
LOC613037	28950	2136,0651	0,7336	0,0245	4,00E-04
ITGB1	25766	2140,4143	0,6648	0,0246	4,00E-04
LOC652616	32793	2142,3079	0,7046	0,0247	4,00E-04
SNRP70	43574	2145,8422	0,7048	0,0249	4,00E-04
LHFPL2	27187	2148,1937	0,6871	0,025	4,00E-04
SLC16A5	42834	2150,0425	0,7119	0,0251	5,00E-04
APPL2	1343	2150,75	0,7207	0,0251	5,00E-04
LOC648852	31677	2156,586	0,6887	0,0254	5,00E-04
TMEM134	45443	2155,6244	0,6896	0,0254	5,00E-04
ITPR1	25819	2155,9061	0,6984	0,0254	5,00E-04
PIP5K2B	38943	2159,235	0,6904	0,0255	5,00E-04
C20ORF195	3517	2159,7598	0,7026	0,0255	5,00E-04
SPIB	43905	2161,7725	0,6846	0,0256	5,00E-04
MMD	35421	2165,8363	0,6898	0,0258	5,00E-04
RAB31	40493	2169,445	0,6581	0,026	5,00E-04
IL12A	25368	2171,477	0,6806	0,026	5,00E-04
GJC1	10833	2173,4262	0,6939	0,026	5,00E-04
STAG3	44216	2178,8901	0,7572	0,0262	5,00E-04
CCM2	4794	2182,5515	0,7115	0,0264	5,00E-04
MORC3	35510	2187,9972	0,6889	0,0266	5,00E-04
SLC16A4	42832	2195,8969	0,6869	0,0269	5,00E-04
PGBD2	38686	2194,7511	0,7016	0,0269	5,00E-04
ALS2CR16	924	2198,845	0,6955	0,027	5,00E-04
TTC3	46397	2203,2641	0,6796	0,0271	5,00E-04
MGC3207	35192	2201,3625	0,6891	0,0271	5,00E-04
C21ORF55	3599	2199,2215	0,6913	0,0271	5,00E-04
AGPAT5	652	2205,0613	0,7042	0,0271	5,00E-04
ZNF193	48104	2203,3124	0,7142	0,0271	5,00E-04
C1ORF66	3400	2202,7775	0,6903	0,0272	5,00E-04
HS.520349	16590	2202,3079	0,6963	0,0272	5,00E-04
ECHDC2	8006	2201,1529	0,7043	0,0272	5,00E-04
USF1	46972	2203,2099	0,705	0,0272	5,00E-04
ABTB1	203	2211,655	0,6768	0,0274	5,00E-04

COMMD6	6058	2212,6553	0,6884	0,0274	5,00E-04
FXYD7	10360	2213,5085	0,6698	0,0275	5,00E-04
RUNDC2C	41812	2214,4177	0,6951	0,0275	5,00E-04
KRCC1	26726	2220,0287	0,6949	0,0276	5,00E-04
ARHGAP24	1428	2218,9952	0,7046	0,0276	5,00E-04
LOC728734	33706	2218,127	0,6955	0,0277	5,00E-04
CD6	4994	2218,9717	0,7503	0,0277	5,00E-04
ANKRD10	1059	2222,8254	0,7041	0,0278	5,00E-04
RNGTT	41417	2233,5015	0,6836	0,0282	5,00E-04
SHISA5	42649	2234,0757	0,6965	0,0282	5,00E-04
SNX1	43609	2232,7491	0,7051	0,0282	5,00E-04
KIAA0101	26169	2231,4146	0,7108	0,0282	5,00E-04
ABI1	177	2239,7869	0,6827	0,0283	5,00E-04
AKR1D1	788	2236,5245	0,6978	0,0283	5,00E-04
HS.46506	16139	2238,6819	0,7166	0,0283	5,00E-04
HBA2	11682	2237,8574	0,8537	0,0283	5,00E-04
CDC16	5060	2241,8754	0,7012	0,0284	5,00E-04
CGGBP1	5425	2251,3023	0,6868	0,0287	5,00E-04
SRRM2	44087	2253,6091	0,6994	0,0287	5,00E-04
SF3B1	42459	2251,9131	0,7012	0,0287	5,00E-04
HBP1	11692	2251,8678	0,7043	0,0287	5,00E-04
LOC388969	28091	2250,918	0,7087	0,0287	5,00E-04
ANKRD13A	1063	2255,2308	0,7071	0,0288	5,00E-04
ZCCHC11	47854	2262,2122	0,6882	0,0291	6,00E-04
AP1G2	1216	2263,8753	0,7066	0,0291	6,00E-04
MSRB2	35789	2261,1011	0,7097	0,0291	6,00E-04
BMPR2	2422	2266,5516	0,6731	0,0292	6,00E-04
DKFZP586I1420	7412	2266,7624	0,7038	0,0292	6,00E-04
ZFP90	47946	2269,1218	0,7037	0,0293	6,00E-04
HS.565545	21828	2270,5902	0,7052	0,0293	6,00E-04
MTX3	35912	2270,8127	0,7172	0,0293	6,00E-04
SELP	42248	2270,014	0,7224	0,0293	6,00E-04
AIM2	712	2274,5491	0,6756	0,0294	6,00E-04
NMT2	36708	2279,1762	0,678	0,0295	6,00E-04
SYNE2	44530	2278,1061	0,6825	0,0295	6,00E-04
CLDN23	5686	2278,0711	0,6705	0,0296	6,00E-04
ST6GAL1	44174	2277,2588	0,7022	0,0296	6,00E-04
BIRC3	2366	2281,5911	0,7317	0,0297	6,00E-04
CTSL1	6600	2285,7794	0,6851	0,0298	6,00E-04
ZNF831	48709	2284,6458	0,7037	0,0298	6,00E-04
CDK5RAP3	5179	2283,2215	0,7182	0,0298	6,00E-04
ZNF671	48590	2292,0129	0,7034	0,03	6,00E-04
D2HGDH	6865	2292,5223	0,7035	0,03	6,00E-04
ENPP2	8357	2292,5948	0,715	0,03	6,00E-04
ANGEL2	1014	2295,0897	0,6898	0,0301	6,00E-04
SCRN1	42100	2296,5599	0,6934	0,0301	6,00E-04
LOC643509	29734	2294,3265	0,7051	0,0301	6,00E-04

SLC44A1	43165	2300,1658	0,6799	0,0302	6,00E-04
NPEPL1	36829	2299,5569	0,6924	0,0302	6,00E-04
ANKRD12	1062	2302,02	0,6806	0,0303	6,00E-04
C5ORF4	3855	2305,1656	0,7006	0,0305	6,00E-04
DCLRE1C	6972	2308,8035	0,7022	0,0307	6,00E-04
SYS1	44562	2312,2753	0,6935	0,0308	6,00E-04
TOR1AIP1	45870	2316,0321	0,7081	0,0309	6,00E-04
DUSP22	7880	2317,4491	0,6927	0,031	6,00E-04
AES	588	2319,6175	0,6987	0,031	6,00E-04
DACT1	6880	2315,7286	0,7192	0,031	6,00E-04
CAMK1D	4340	2322,763	0,7027	0,0311	6,00E-04
SENP7	42298	2319,5867	0,7028	0,0311	6,00E-04
CXCR5	6675	2322,3044	0,7115	0,0311	6,00E-04
SLC15A2	42817	2322,1328	0,7141	0,0311	6,00E-04
C8ORF70	4069	2328,1854	0,6969	0,0314	6,00E-04
PELI2	38597	2330,2763	0,7252	0,0315	6,00E-04
LSM14A	34295	2334,447	0,7065	0,0316	6,00E-04
SIRT1	42728	2333,1418	0,7076	0,0316	6,00E-04
PIGC	38862	2334,6296	0,7193	0,0316	6,00E-04
ZNF160	48077	2338,1118	0,7169	0,0318	6,00E-04
RPS6KA5	41681	2338,0637	0,7007	0,0319	6,00E-04
GPR1	11129	2343,1186	0,6907	0,032	6,00E-04
SESN1	42423	2345,1835	0,6968	0,0321	6,00E-04
LOC338799	27830	2348,5409	0,715	0,0322	6,00E-04
AKAP11	741	2346,9552	0,7218	0,0322	6,00E-04
ZNF559	48455	2352,46	0,6867	0,0323	6,00E-04
REEP5	40966	2351,7847	0,6937	0,0323	6,00E-04
XRCC2	47681	2358,0187	0,7034	0,0325	7,00E-04
LOC88523	34043	2358,8599	0,709	0,0325	7,00E-04
EAF2	7976	2360,4494	0,6945	0,0326	7,00E-04
NIPBL	36595	2362,0314	0,6973	0,0326	7,00E-04
LOC644935	30386	2366,5096	0,6994	0,0326	7,00E-04
SIDT1	42678	2368,4148	0,7015	0,0326	7,00E-04
NOSIP	36780	2369,0199	0,7024	0,0326	7,00E-04
CSTF3	6473	2365,5802	0,7047	0,0326	7,00E-04
SSTR2	44124	2361,1817	0,7081	0,0326	7,00E-04
TRIM13	46050	2361,488	0,7106	0,0326	7,00E-04
ZNF239	48157	2365,9009	0,7116	0,0326	7,00E-04
ST3GAL1	44159	2364,5074	0,7129	0,0326	7,00E-04
C11ORF46	2770	2365,5529	0,7177	0,0326	7,00E-04
PIAS1	38842	2367,5311	0,7204	0,0326	7,00E-04
MYO9B	36086	2374,9517	0,6996	0,033	7,00E-04
LOC730302	33887	2376,4326	0,7258	0,033	7,00E-04
GBP1	10609	2375,1347	0,7818	0,033	7,00E-04
ZNF652	48562	2378,4763	0,7169	0,0331	7,00E-04
HS.334831	15003	2376,2861	0,7229	0,0331	7,00E-04
PEG10	38594	2383,6392	0,7097	0,0333	7,00E-04

LOC492311	28901	2386,6477	0,7015	0,0335	7,00E-04
LOC653841	33391	2389,014	0,6963	0,0337	7,00E-04
RRM2B	41723	2391,696	0,7072	0,0337	7,00E-04
NOD1	36729	2389,5954	0,7077	0,0337	7,00E-04
RBM12B	40804	2391,3336	0,7143	0,0337	7,00E-04
MSH3	35761	2394,8821	0,7124	0,034	7,00E-04
SHROOM4	42669	2397,4502	0,698	0,0341	7,00E-04
DDX28	7076	2399,6572	0,7021	0,0343	7,00E-04
SIAH1	42676	2402,6243	0,6928	0,0344	7,00E-04
CHI3L2	5479	2416,1712	0,6953	0,0351	7,00E-04
LOC642947	29478	2417,4271	0,7104	0,0351	7,00E-04
HS.154336	13486	2416,1027	0,6967	0,0352	7,00E-04
LEMD3	27122	2421,7509	0,7084	0,0352	7,00E-04
DBNDD1	6929	2418,9623	0,7174	0,0352	7,00E-04
ZFP37	47939	2415,7814	0,7583	0,0352	7,00E-04
LOC400464	28363	2422,2802	0,6854	0,0353	7,00E-04
ADHFE1	531	2427,2739	0,7062	0,0355	7,00E-04
PLEKHA1	39123	2433,5	0,7176	0,0358	7,00E-04
RGPD1	41105	2434,7614	0,6773	0,0359	7,00E-04
ZC3H7A	47843	2442,7376	0,7046	0,0363	8,00E-04
CSNK1G3	6429	2442,136	0,7166	0,0363	8,00E-04
UIMC1	46866	2441,9424	0,7198	0,0363	8,00E-04
TMEM123	45426	2449,1341	0,6881	0,0364	8,00E-04
CCDC130	4610	2445,7954	0,7056	0,0364	8,00E-04
RABGAP1	40551	2448,5767	0,7174	0,0364	8,00E-04
ZBTB5	47819	2447,4502	0,7284	0,0364	8,00E-04
SNRP70	43575	2451,0865	0,6934	0,0366	8,00E-04
BBS2	2188	2452,5146	0,7053	0,0366	8,00E-04
ZBTB24	47797	2455,3485	0,7238	0,0367	8,00E-04
PLCH2	39095	2458,0767	0,7049	0,0368	8,00E-04
ST3GAL5	44165	2458,7076	0,7095	0,0369	8,00E-04
GIT2	10807	2460,5694	0,7041	0,037	8,00E-04
FOXO1	10158	2461,6975	0,7054	0,037	8,00E-04
PVRIG	40364	2461,4318	0,7056	0,037	8,00E-04
ROCK2	41443	2462,4022	0,7088	0,037	8,00E-04
ROR1	41457	2467,4432	0,7039	0,0371	8,00E-04
TTC17	46381	2465,7884	0,7219	0,0371	8,00E-04
CAPN3	4395	2466,49	0,7093	0,0372	8,00E-04
HS.354359	15116	2475,4836	0,6955	0,0374	8,00E-04
KIAA0831	26266	2471,6993	0,7162	0,0374	8,00E-04
LOC400721	28384	2473,3658	0,7216	0,0374	8,00E-04
HS.568676	22410	2472,6844	0,7231	0,0374	8,00E-04
TSPYL1	46345	2476,715	0,731	0,0374	8,00E-04
IL7	25507	2475,1372	0,6954	0,0375	8,00E-04
LOC653489	33229	2474,5202	0,6987	0,0375	8,00E-04
JMJD1C	25858	2471,663	0,706	0,0375	8,00E-04
BTN2A1	2593	2474,3497	0,7147	0,0375	8,00E-04

C14ORF102	2892	2479,0673	0,7234	0,0375	8,00E-04
FLJ20489	9637	2479,1321	0,7235	0,0375	8,00E-04
GNPTG	11018	2478,884	0,7014	0,0376	8,00E-04
SETD2	42431	2482,5831	0,7041	0,0378	8,00E-04
CDK5RAP2	5176	2484,7607	0,7124	0,0378	8,00E-04
OCEL1	37219	2484,9382	0,7205	0,0378	8,00E-04
GUSBL1	11578	2484,7596	0,7042	0,0379	8,00E-04
DGKD	7245	2485,4662	0,7132	0,0379	8,00E-04
LILRB3	27234	2494,7375	0,7177	0,0383	8,00E-04
ZNF177	48088	2497,7725	0,6962	0,0384	8,00E-04
ANAPC4	1008	2502,1186	0,6998	0,0385	8,00E-04
ZNF285A	48206	2501,7516	0,7567	0,0385	8,00E-04
C10ORF58	2699	2500,9174	0,7074	0,0386	8,00E-04
USP47	47047	2501,5289	0,719	0,0386	8,00E-04
WDR26	47388	2507,0447	0,7158	0,0387	8,00E-04
RN7SL1	41266	2504,882	0,7256	0,0387	8,00E-04
HS.130036	12845	2510,6676	0,7056	0,0389	8,00E-04
ZCCHC7	47868	2517,6744	0,7128	0,0393	9,00E-04
ITM2B	25810	2520,5011	0,7048	0,0395	9,00E-04
PHF21A	38782	2523,8732	0,7236	0,0396	9,00E-04
TCEA2	44872	2531,5934	0,6971	0,04	9,00E-04
KLHDC9	26613	2529,4173	0,7315	0,04	9,00E-04
C12ORF47	2846	2533,7393	0,7194	0,0401	9,00E-04
BMF	2391	2531,3954	0,7209	0,0401	9,00E-04
NADK	36136	2533,0912	0,7263	0,0401	9,00E-04
DMD	7506	2537,8609	0,694	0,0403	9,00E-04
PARP11	38124	2538,4253	0,7183	0,0403	9,00E-04
LCOR	27077	2539,104	0,7217	0,0403	9,00E-04
HS.483906	16242	2539,2026	0,7307	0,0403	9,00E-04
ARSD	1629	2536,7406	0,8152	0,0403	9,00E-04
PTP4A2	40237	2544,2528	0,727	0,0405	9,00E-04
FRAT1	10197	2547,0465	0,7083	0,0406	9,00E-04
TTLL3	46432	2548,7419	0,7093	0,0406	9,00E-04
CRIPAK	6282	2545,9869	0,7146	0,0406	9,00E-04
SDHA	42150	2546,9507	0,7228	0,0406	9,00E-04
PDE7A	38480	2549,8178	0,7229	0,0407	9,00E-04
LOC728565	33684	2551,6411	0,7186	0,0408	9,00E-04
PSCD1	40021	2557,6876	0,7346	0,0411	9,00E-04
SFRS14	42490	2560,2818	0,718	0,0412	9,00E-04
HS.211743	14187	2558,7752	0,7236	0,0412	9,00E-04
RGS2	41136	2562,7259	0,7144	0,0413	9,00E-04
LOC644250	30073	2563,7679	0,7108	0,0414	9,00E-04
LOC652458	32709	2566,5236	0,7448	0,0415	9,00E-04
GPSM3	11295	2567,7241	0,7107	0,0416	9,00E-04
SYVN1	44597	2568,1466	0,7275	0,0416	9,00E-04
ULK1	46869	2569,3155	0,718	0,0417	9,00E-04
CASP1	4451	2570,9313	0,7182	0,0417	9,00E-04

ANKRD28	1099	2573,0243	0,7171	0,0418	9,00E-04
SEPN1	42306	2577,4896	0,7155	0,042	9,00E-04
RPL7L1	41588	2578,5581	0,7185	0,042	9,00E-04
RNF38	41393	2581,7419	0,7155	0,0423	0,001
S100PBP	41887	2585,3523	0,7229	0,0425	0,001
SLC35E1	43089	2588,6083	0,7125	0,0427	0,001
LOC92497	34065	2592,8123	0,7306	0,043	0,001
CDC16	5058	2594,3659	0,7324	0,043	0,001
NT5C3	37022	2597,7796	0,7074	0,0431	0,001
TMEM63A	45611	2596,1337	0,7106	0,0431	0,001
C17ORF87	3146	2596,5889	0,7111	0,0431	0,001
MGEA5	35279	2597,8642	0,722	0,0431	0,001
ZNF767	48661	2598,9806	0,7233	0,0431	0,001
BMF	2394	2600,7486	0,7396	0,0432	0,001
NSUN7	37014	2604,2168	0,7197	0,0434	0,001
MLL5	35393	2604,9972	0,7253	0,0434	0,001
TMEM42	45585	2607,5621	0,722	0,0435	0,001
PLEKHA9	39135	2607,1775	0,7417	0,0435	0,001
SPIN4	43914	2611,4157	0,7143	0,0437	0,001
ZNF828	48704	2615,4904	0,7175	0,0439	0,001
ZNF816A	48698	2621,003	0,7134	0,0442	0,001
LOC728153	33622	2622,713	0,7198	0,0443	0,001
PPP1CB	39571	2626,976	0,7058	0,0444	0,001
AFTPH	615	2624,3065	0,7239	0,0444	0,001
TCIRG1	44938	2626,9696	0,7059	0,0445	0,001
MGC3207	35189	2631,6437	0,7241	0,0445	0,001
SCARB2	42015	2628,3956	0,7305	0,0445	0,001
LOC648622	31609	2629,9749	0,7306	0,0445	0,001
ZNF514	48409	2630,9666	0,7369	0,0445	0,001
AKAP7	764	2632,4444	0,7865	0,0445	0,001
STAG2	44214	2637,2691	0,7119	0,0446	0,001
BTN3A2	2600	2635,7804	0,7176	0,0446	0,001
GSTK1	11467	2637,0373	0,7184	0,0446	0,001
STK38	44318	2636,9639	0,7322	0,0446	0,001
TMEM143	45452	2639,5242	0,7169	0,0447	0,001
GTF2IRD2B	11526	2635,6758	0,7255	0,0447	0,001
CCDC84	4730	2634,5042	0,7288	0,0447	0,001
KIAA2026	26457	2641,3001	0,7175	0,0448	0,001
ARHGAP4	1444	2642,2208	0,7203	0,0448	0,001
KIAA0528	26229	2644,0309	0,7397	0,0449	0,001
HPS3	12241	2646,0109	0,7317	0,045	0,001
HS.20255	14067	2650,3918	0,7366	0,0452	0,0011
CAPN3	4396	2653,6983	0,7182	0,0454	0,0011
HS.516646	16501	2654,6404	0,7286	0,0454	0,0011
HS.163752	13669	2656,4326	0,7169	0,0455	0,0011
FRAT2	10199	2657,242	0,7173	0,0455	0,0011
MGST1	35290	2659,5284	0,7273	0,0456	0,0011

PPP2R5B	39651	2660,9808	0,7446	0,0456	0,0011
ASF1A	1706	2665,2558	0,7055	0,0457	0,0011
ZDHHC8	47904	2664,5556	0,7106	0,0457	0,0011
C17ORF48	3102	2668,1961	0,7184	0,0457	0,0011
TULP4	46546	2663,88	0,7201	0,0457	0,0011
DMTF1	7527	2666,1475	0,7258	0,0457	0,0011
ATP2B1	1867	2662,4885	0,7273	0,0457	0,0011
CALCOCO1	4316	2664,4192	0,7372	0,0457	0,0011
CYORF15A	6764	2666,5175	0,9041	0,0457	0,0011
ACSS1	335	2669,5361	0,7131	0,0458	0,0011
PLCG2	39093	2670,3158	0,7228	0,0458	0,0011
SERPINB2	42391	2668,0455	0,8351	0,0458	0,0011
LOC654103	33471	2676,207	0,7154	0,046	0,0011
C14ORF135	2922	2674,7474	0,726	0,046	0,0011
LOC441155	28737	2678,8038	0,7206	0,0461	0,0011
IL1RN	25446	2678,8092	0,7463	0,0461	0,0011
LIG1	27210	2683,1385	0,7049	0,0462	0,0011
CRYGS	6347	2681,5819	0,7242	0,0462	0,0011
KRCC1	26725	2685,7569	0,7276	0,0464	0,0011
CSF2RA	6381	2691,8658	0,7255	0,0465	0,0011
ARID4B	1499	2689,8412	0,7336	0,0465	0,0011
WEE1	47492	2689,4595	0,7323	0,0466	0,0011
ZNF175	48087	2690,7295	0,7347	0,0466	0,0011
AFAP1L2	594	2695,1261	0,7256	0,0467	0,0011
LOC642989	29498	2700,563	0,7097	0,047	0,0011
DBNDD1	6928	2700,7853	0,7252	0,047	0,0011
RHBDF2	41165	2699,9025	0,7306	0,047	0,0011
CAPN12	4389	2708,4946	0,6971	0,0475	0,0011
LOC400304	28355	2708,0589	0,7332	0,0475	0,0011
CRLF3	6300	2709,8699	0,7267	0,0476	0,0011
TAGAP	44674	2715,0033	0,7211	0,0478	0,0011
TSPAN3	46320	2713,8343	0,7231	0,0478	0,0011
ZCWPW1	47872	2713,0911	0,7251	0,0478	0,0011
HS.15956	13597	2721,0593	0,7315	0,0482	0,0012
AP3B1	1237	2723,2886	0,7165	0,0484	0,0012
HERC1	11797	2728,5404	0,7214	0,0486	0,0012
IFNAR1	25243	2725,9012	0,7387	0,0486	0,0012
HS.569104	22470	2726,7138	0,7808	0,0486	0,0012
ZNF483	48380	2731,7127	0,7285	0,0488	0,0012
CD79A	5008	2733,3812	0,7381	0,0488	0,0012
BRWD2	2543	2732,088	0,7987	0,0488	0,0012
RPS27A	41652	2735,186	0,7135	0,0489	0,0012
DEPDC5	7197	2734,0003	0,7351	0,0489	0,0012
KLHL3	26637	2740,2874	0,722	0,0491	0,0012
N4BP2	36119	2745,7217	0,7251	0,0494	0,0012
ICK	25165	2748,0599	0,7169	0,0495	0,0012
FLJ38717	9822	2748,3408	0,7245	0,0495	0,0012

OFD1	37247	2750,7644	0,7366	0,0497	0,0012
IL8	25510	2754,9447	0,7846	0,05	0,0012
