# Supplementary material for: CLL Cells Respond to B-Cell Receptor Stimulation with a MicroRNA/mRNA Signature Associated with MYC Activation and Cell Cycle Progression
Source: PLoS One. 2013 Apr 1;8(4):e60275. doi: 10.1371/journal.pone.0060275 (PMC3613353; doi:10.1371/journal.pone.0060275)
Supplement: Table S4 — Gene Set Enrichment Analysis of unstimulated versus stimulated sample categories. Table shows gene sets found significantly enriched in either sample category from gene set collections KEGG (shown in black characters), Gene Ontology Biological Process (shown in blue characters), and Transcription Factor Targets (shown in red characters), ranked according normalized enrichment score (NES). False discovery rate (FDR) q value, based on 1,000 permutations of category labels, was below 0.05. Category indicates sample type gene set is enriched in (negative scores of NES for stimulated samples). (PDF) [file pone.0060275.s011.pdf]

Gene Set	NES
KEGG_PROTEASOME	-2,7981236
KEGG_GLYCOLYSIS_GLUCONEOGENESIS	-2,5058157
KEGG_AMINOACYL_TRNA_BIOSYNTHESIS	-2,4161668
PROTEIN_FOLDING	-2,3515246
NITROGEN_COMPOUND_METABOLIC_PROCESS	-2,2858992
AMINE_METABOLIC_PROCESS	-2,2382627
AMINO_ACID_METABOLIC_PROCESS	-2,2372925
KEGG_PARKINSONS_DISEASE	-2,2234716
KEGG_PENTOSE_PHOSPHATE_PATHWAY	-2,1792994
AMINO_ACID_AND_DERIVATIVE_METABOLIC_PROCESS	-2,1588688
KEGG_CITRATE_CYCLE_TCA_CYCLE	-2,1531835
KEGG_CYSTEINE_AND_METHIONINE_METABOLISM	-2,1479754
KEGG_OXIDATIVE_PHOSPHORYLATION	-2,1261601
V$MYCMAX_01	-2,107117
COFACTOR_METABOLIC_PROCESS	-2,102321
MITOCHONDRION_ORGANIZATION_AND_BIOGENESIS	-2,1017635
TRNA_METABOLIC_PROCESS	-2,100583
KEGG_PROTEIN_EXPORT	-2,0880094
KEGG_ALZHEIMERS_DISEASE	-2,0857005
V$MYC_Q2	-2,0798883
KEGG_AMINO_SUGAR_AND_NUCLEOTIDE_SUGAR_METABOLISM	-2,0778565
CARBOXYLIC_ACID_METABOLIC_PROCESS	-2,0747218
ORGANIC_ACID_METABOLIC_PROCESS	-2,0744777
CELLULAR_BIOSYNTHETIC_PROCESS	-2,0706565
KEGG_FRUCTOSE_AND_MANNOSE_METABOLISM	-2,0652034
KEGG_PATHOGENIC_ESCHERICHIA_COLI_INFECTION	-2,0367074
HETEROCYCLE_METABOLIC_PROCESS	-2,0319421
KEGG_RNA_POLYMERASE	-2,0271745
MITOCHONDRIAL_TRANSPORT	-2,020482
BIOSYNTHETIC_PROCESS	-2,0054471
V$HIF1_Q3	-2,0014095
KEGG_ARGININE_AND_PROLINE_METABOLISM	-1,9881915
KEGG_TERPENOID_BACKBONE_BIOSYNTHESIS	-1,9849468
V$GABP_B	-1,9784379
ALCOHOL_METABOLIC_PROCESS	-1,9687159
KEGG_SELENOAMINO_ACID_METABOLISM	-1,9603868
KEGG_GLYOXYLATE_AND_DICARBOXYLATE_METABOLISM	-1,9559577
COENZYME_METABOLIC_PROCESS	-1,9552972
V$USF_C	-1,9532865
CELLULAR_CARBOHYDRATE_METABOLIC_PROCESS	-1,9428166
KEGG_CARDIAC_MUSCLE_CONTRACTION	-1,937909
KEGG_ALANINE_ASPARTATE_AND_GLUTAMATE_METABOLISM	-1,9321718
KEGG_HUNTINGTONS_DISEASE	-1,9298851

KEGG_SPLICEOSOME	-1,9281821
KEGG_VIBRIO_CHOLERAE_INFECTION	-1,9272383
RNA_PROCESSING	-1,9135625
POSITIVE_REGULATION_OF_CELL_CYCLE	-1,9089651
AROMATIC_COMPOUND_METABOLIC_PROCESS	-1,8972907
TTCNRGNNNNTTC_V$HSF_Q6	-1,891327
RIBONUCLEOPROTEIN_COMPLEX_BIOGENESIS_AND_ASSEMBLY	-1,890157
TGASTMAGC_V$NFE2_01	-1,8729239
CGGAARNGGCNG_UNKNOWN	-1,870343
AMINO_ACID_TRANSPORT	-1,8672116
KEGG_N_GLYCAN_BIOSYNTHESIS	-1,8670521
SECONDARY_METABOLIC_PROCESS	-1,8659898
RESPONSE_TO_OXIDATIVE_STRESS	-1,8634323
V$USF2_Q6	-1,8536484
CELLULAR_CARBOHYDRATE_CATABOLIC_PROCESS	-1,8493507
KEGG_GALACTOSE_METABOLISM	-1,8465056
PIGMENT_METABOLIC_PROCESS	-1,8455678
KEGG_RIBOSOME	-1,8419878
KEGG_PYRUVATE_METABOLISM	-1,834186
CARBOHYDRATE_BIOSYNTHETIC_PROCESS	-1,8299872
V$ATF_01	-1,8240834
V$MYCMAX_B	-1,8200685
V$ELK1_02	-1,817645
NITROGEN_COMPOUND_BIOSYNTHETIC_PROCESS	-1,8164427
V$SRF_01	-1,8150768
V$NMYC_01	-1,8149133
CCAWWNAAGG_V$SRF_Q4	-1,8016021
KEGG_PYRIMIDINE_METABOLISM	-1,793939
KEGG_RNA_DEGRADATION	-1,7912732
TRANSLATION	-1,7912457
TGANNYRGCA_V$TCF11MAFG_01	-1,7799392
KEGG_VALINE_LEUCINE_AND_ISOLEUCINE_DEGRADATION	-1,7790892
STEROID_BIOSYNTHETIC_PROCESS	-1,7782346
CARBOHYDRATE_CATABOLIC_PROCESS	-1,77568
V$NRF2_01	-1,7729795
KEGG_ONE_CARBON_POOL_BY_FOLATE	-1,7683319
KEGG_STEROID_BIOSYNTHESIS	-1,7608944
V$NRF2_Q4	-1,7556549
KEGG_PHENYLALANINE_METABOLISM	-1,752845
PIGMENT_BIOSYNTHETIC_PROCESS	-1,748059
V$NFE2_01	-1,7434299
REGULATION_OF_CELL_CYCLE	-1,7413938
CELL_CYCLE_GO_0007049	-1,7403742
V$ARNT_01	-1,7402039

CARBOHYDRATE_METABOLIC_PROCESS	-1,7397976
V$SRF_Q6	-1,7389747
NUCLEOBASENUCLEOSIDE_AND_NUCLEOTIDE_METABOLIC_PROCESS	-1,7330017
CARBOXYLIC_ACID_TRANSPORT	-1,7312112
AMINO_SUGAR_METABOLIC_PROCESS	-1,7307831
MITOTIC_CELL_CYCLE	-1,7293234
ORGANIC_ACID_TRANSPORT	-1,7221488
MRNA_METABOLIC_PROCESS	-1,7125987
MACROMOLECULE_BIOSYNTHETIC_PROCESS	-1,7048229
V$ATF6_01	-1,704472
AMINE_BIOSYNTHETIC_PROCESS	-1,7035104
KEGG_PROXIMAL_TUBULE_BICARBONATE_RECLAMATION	-1,7001636
V$CREB_Q3	-1,6983218
V$AP1_01	-1,6970884
KEGG_LYSOSOME	-1,6964374
V$XBP1_01	-1,6843419
V$HIF1_Q5	-1,675343
KEGG_PURINE_METABOLISM	-1,6592855
ACTAYRNNNCCCR_UNKNOWN	-1,6583364
V$MAX_01	-1,6516819
V$PPARG_01	-1,6481
V$USF_Q6	-1,641402
KEGG_PORPHYRIN_AND_CHLOROPHYLL_METABOLISM	-1,6348813
KEGG_TYROSINE_METABOLISM	-1,6328541
CGTSACG_V$PAX3_B	-1,623522
V$CREBP1_Q2	-1,6211461
GKCGCNNNNNNNTGAYG_UNKNOWN	-1,6192927
ACTWSNACTNY_UNKNOWN	-1,6143744
YYCATTCAWW_UNKNOWN	-1,6104406
KTGGYRSGAA_UNKNOWN	-1,6071126
RRCCGTTA_UNKNOWN	-1,6051728
TCCCRNNRTGC_UNKNOWN	-1,6040845
V$E2F_Q4_01	-1,6039628
V$SRF_C	-1,6000705
V$CREB_Q4_01	-1,5942569
KEGG_GLYCINE_SERINE_AND_THREONINE_METABOLISM	-1,5935856
V$E2F1_Q6_01	-1,5897303
V$TCF11MAFG_01	-1,5892336
V$USF_01	-1,5888231
KEGG_DNA_REPLICATION	-1,5873499
TCANNTGAY_V$SREBP1_01	-1,5869843
V$MYCMAX_03	-1,5839589
SGCGSSAAA_V$E2F1DP2_01	-1,5731711
GGAANCGGAANY_UNKNOWN	-1,5698109

V$HNF4_01	-1,5661803
TTCYRGAA_UNKNOWN	-1,5580405
V$E2F4DP2_01	-1,5575514
V$ATF_B	-1,5550233
V$E2F1DP1_01	-1,5546685
V$COUP_DR1_Q6	-1,5525135
V$CREB_02	-1,5479219
V$PPAR_DR1_Q2	-1,5470638
V$E2F1DP2_01	-1,5450486
V$E2F_02	-1,5439019
YGCGYRCGC_UNKNOWN	-1,5436958
V$ERR1_Q2	-1,5418429
V$E2F4DP1_01	-1,540896
V$CREB_Q2_01	-1,5376639
V$STAT1_02	-1,5354567
V$NRF1_Q6	-1,5287105
V$ALPHACP1_01	-1,5216415
V$E2F_01	-1,5203168
V$CREB_01	-1,5039147
SNACANNNYSYAGA_UNKNOWN	-1,4983753
V$ARNT_02	-1,4963322
V$E2F1_Q6	-1,4948996
V$AP1_Q4_01	-1,4919646
TGACCTTG_V$SF1_Q6	-1,4873445
V$CREBP1CJUN_01	-1,4849901
TGACGTCA_V$ATF3_Q6	-1,4822891
V$CETS1P54_01	-1,4821042
V$SRF_Q4	-1,4806056
V$SRF_Q5_01	-1,4759026
V$HEB_Q6	-1,4686565
V$USF_Q6_01	-1,4660532
V$BACH2_01	-1,4645076
V$HTF_01	-1,4476479
V$STAT5A_01	-1,4466268
V$E2F1_Q3	-1,4446814
V$USF_02	-1,4390981
V$AP1_Q6	-1,4389751
V$ZF5_01	-1,4388026
V$NFY_C	-1,4355828
CCAATNNSNNNGCG_UNKNOWN	-1,4352742
V$E2F_Q6_01	-1,4335244
V$E4F1_Q6	-1,4327898
TGCGCANK_UNKNOWN	-1,4315721
V$E2F1_Q4_01	-1,4304066

V$MYCMAX_02	-1,4160752
V$E2F1DP1RB_01	-1,4108465
V$E2F_03	-1,4065132
V$AP1_Q6_01	-1,4050207
V$DR1_Q3	-1,4045991
V$E2F_Q3_01	-1,4022812
V$AP1_C	-1,4021095
CTGRYYYNATT_UNKNOWN	-1,3944839
V$NFY_01	-1,3940098
V$BACH1_01	-1,3905578
V$PAX3_01	-1,3880159
V$CREL_01	1,5825131
V$IK3_01	1,5908895
V$PEA3_Q6	1,5917994
V$HP1SITEFACTOR_Q6	1,5944347
V$NFKAPPAB_01	1,6007775
V$ETS1_B	1,6039326
V$IRF7_01	1,6160452
V$GR_Q6_01	1,6333253
V$IRF1_Q6	1,6953967
V$PAX8_01	1,6971809
V$ELF1_Q6	1,7918807
STTTCRNTTT_V$IRF_Q6	1,8374724
POSITIVE_REGULATION_OF_MAP_KINASE_ACTIVITY	1,8825512
V$ICSBP_Q6	1,9820628
CYTOKINE_PRODUCTION	2,0034878
RESPONSE_TO_BACTERIUM	2,0225382
RESPONSE_TO_OTHER_ORGANISM	2,0245862
V$ISRE_01	2,0419588
V$IRF_Q6	2,1116283

FDR q-value	Category
0	stimulated
0	stimulated
0	stimulated
0	stimulated
0	stimulated
0	stimulated
0	stimulated
0	stimulated
0	stimulated
7,10E-04	stimulated
0	stimulated
0	stimulated
4,90E-04	stimulated
0,002037342	stimulated
0,001548958	stimulated
0,001327678	stimulated
0,001161719	stimulated
5,97E-04	stimulated
5,37E-04	stimulated
0,001018671	stimulated
4,88E-04	stimulated
0,001805034	stimulated
0,00162453	stimulated
0,001684934	stimulated
4,47E-04	stimulated
4,13E-04	stimulated
0,002426604	stimulated
3,84E-04	stimulated
0,002785516	stimulated
0,003262081	stimulated
0,001721177	stimulated
7,96E-04	stimulated
7,46E-04	stimulated
0,001826473	stimulated
0,005047221	stimulated
0,001251231	stimulated
0,001181718	stimulated
0,005889482	stimulated
0,001461179	stimulated
0,006783405	stimulated
0,001478017	stimulated
0,001464869	stimulated
0,001395113	stimulated

0,001331699	stimulated
0,001273799	stimulated
0,009773843	stimulated
0,009927046	stimulated
0,011101742	stimulated
0,002965855	stimulated
0,011402897	stimulated
0,002960752	stimulated
0,003099069	stimulated
0,014165985	stimulated
0,003084766	stimulated
0,013601732	stimulated
0,01332595	stimulated
0,003441172	stimulated
0,015626155	stimulated
0,003863811	stimulated
0,015339699	stimulated
0,003974493	stimulated
0,004520753	stimulated
0,018391995	stimulated
0,00487207	stimulated
0,004621004	stimulated
0,004323758	stimulated
0,020315342	stimulated
0,003991162	stimulated
0,003706079	stimulated
0,004091826	stimulated
0,007240905	stimulated
0,007082444	stimulated
0,025500683	stimulated
0,004351031	stimulated
0,007549192	stimulated
0,028080828	stimulated
0,027955119	stimulated
0,00434314	stimulated
0,00836569	stimulated
0,008875286	stimulated
0,005648669	stimulated
0,009345831	stimulated
0,03684092	stimulated
0,006165095	stimulated
0,038210336	stimulated
0,037395995	stimulated
0,006011244	stimulated

0,036594655	stimulated
0,005822219	stimulated
0,039075874	stimulated
0,038808268	stimulated
0,03787579	stimulated
0,03755456	stimulated
0,039760053	stimulated
0,042526867	stimulated
0,044826943	stimulated
0,007648897	stimulated
0,04413521	stimulated
0,015374668	stimulated
0,007858409	stimulated
0,007616193	stimulated
0,015587701	stimulated
0,008476602	stimulated
0,009015859	stimulated
0,021637624	stimulated
0,010701226	stimulated
0,011138468	stimulated
0,011152527	stimulated
0,011863454	stimulated
0,027140869	stimulated
0,02677902	stimulated
0,013613904	stimulated
0,01351077	stimulated
0,013348889	stimulated
0,013623857	stimulated
0,014051513	stimulated
0,014260245	stimulated
0,014205995	stimulated
0,013860437	stimulated
0,013556045	stimulated
0,013894982	stimulated
0,014440123	stimulated
0,036791228	stimulated
0,014824446	stimulated
0,014524722	stimulated
0,014194614	stimulated
0,037804887	stimulated
0,014268239	stimulated
0,014451876	stimulated
0,016017951	stimulated
0,016391682	stimulated

0,016627487	stimulated
0,017593732	stimulated
0,017490696	stimulated
0,017673567	stimulated
0,017358499	stimulated
0,017416928	stimulated
0,017978797	stimulated
0,017802743	stimulated
0,01768977	stimulated
0,01759534	stimulated
0,017366603	stimulated
0,01733372	stimulated
0,01716968	stimulated
0,017477369	stimulated
0,01762342	stimulated
0,018332748	stimulated
0,019517267	stimulated
0,019543128	stimulated
0,023087228	stimulated
0,02427849	stimulated
0,024529766	stimulated
0,02451279	stimulated
0,024900118	stimulated
0,026244564	stimulated
0,026462894	stimulated
0,026788438	stimulated
0,026474236	stimulated
0,02653413	stimulated
0,027753783	stimulated
0,029096507	stimulated
0,029543351	stimulated
0,029649477	stimulated
0,034573708	stimulated
0,034503996	stimulated
0,034882028	stimulated
0,036497507	stimulated
0,03614408	stimulated
0,035795085	stimulated
0,036578063	stimulated
0,036348674	stimulated
0,036490463	stimulated
0,0363473	stimulated
0,03641908	stimulated
0,03655361	stimulated

0,0419176	stimulated
0,043651555	stimulated
0,0451289	stimulated
0,045287594	stimulated
0,04501096	stimulated
0,0455493	stimulated
0,045163155	stimulated
0,0480993	stimulated
0,047865525	stimulated
0,04902835	stimulated
0,049768426	stimulated
0,047672007	unstimulated
0,046848867	unstimulated
0,04968197	unstimulated
0,05220317	unstimulated
0,052993134	unstimulated
0,05643395	unstimulated
0,05467267	unstimulated
0,052455034	unstimulated
0,029519286	unstimulated
0,033934437	unstimulated
0,01182026	unstimulated
0,008466081	unstimulated
0,04513918	unstimulated
0,001328023	unstimulated
0,009651061	unstimulated
0,010665851	unstimulated
0,02044814	unstimulated
9,98E-04	unstimulated
0	unstimulated
