# Supplementary material for: CLL Cells Respond to B-Cell Receptor Stimulation with a MicroRNA/mRNA Signature Associated with MYC Activation and Cell Cycle Progression
Source: PLoS One. 2013 Apr 1;8(4):e60275. doi: 10.1371/journal.pone.0060275 (PMC3613353; doi:10.1371/journal.pone.0060275)
Supplement: Table S5 — miRNAs detected in BCR stimulated CLL cells. (PDF) [file pone.0060275.s012.pdf]

miRNA (Alfabetical order)	Expression level in screening	miRNA (Sorted in decreasing abundance)	Expression level in screening
hsa-let-7a	15,84	hsa-mir-150	8181,10
hsa-let-7b	21,04	U6	6885,86
hsa-let-7c	0,27	RNU48	4417,19
hsa-let-7d	11,96	RNU44	647,65
hsa-let-7e	86,90	hsa-mir-768-3p	355,14
hsa-let-7f	2,29	hsa-mir-223	257,22
hsa-let-7g	36,48	hsa-mir-142-3p	206,08
hsa-mir-101	0,73	hsa-mir-19b	130,89
hsa-mir-103	4,95	hsa-mir-191	95,01
hsa-mir-106a	40,72	hsa-let-7e	86,90
hsa-mir-106b	10,90	RNU24	80,64
hsa-mir-125a-5p	0,02	hsa-mir-26a	75,12
hsa-mir-126	0,05	hsa-mir-92a	75,08
hsa-mir-126x	0,06	hsa-mir-20a	71,89
hsa-mir-127	0,01	hsa-mir-342-3p	71,80
hsa-mir-128a	0,29	hsa-mir-29a	69,64
hsa-mir-130b	0,24	hsa-mir-21	68,92
hsa-mir-132-3p	8,99	hsa-mir-30c	59,56
hsa-mir-132-5p	0,02	hsa-mir-30b	57,54
hsa-mir-135a	0,02	hsa-mir-135ax	56,96
hsa-mir-135ax	56,96	hsa-mir-17	45,05
hsa-mir-138-1x	0,84	hsa-mir-24	44,73
hsa-mir-140-3p	0,25	hsa-mir-923	43,28
hsa-mir-141	0,05	hsa-mir-222	42,35
hsa-mir-142-3p	206,08	hsa-mir-16	41,83
hsa-mir-142-5p	1,24	hsa-mir-106a	40,72
hsa-mir-146a	3,74	hsa-let-7g	36,48
hsa-mir-146b	7,55	RNU43	35,59
hsa-mir-146b-3p	0,08	hsa-mir-320	31,12
hsa-mir-148a	1,88	hsa-mir-26b	28,95
hsa-mir-148b	0,11	hsa-let-7b	21,04
hsa-mir-148bx	0,02	RNU6B	15,96
hsa-mir-150	8181,10	hsa-let-7a	15,84
hsa-mir-151-3p	0,94	hsa-mir-15b	14,01
hsa-mir-152	0,06	hsa-mir-331	12,33
hsa-mir-155-3p	0,15	hsa-let-7d	11,96
hsa-mir-15a	0,32	hsa-mir-484	11,91
hsa-mir-15ax	0,03	hsa-mir-30a-5p	11,23
hsa-mir-15b	14,01	hsa-mir-29c	11,13
hsa-mir-16	41,83	hsa-mir-106b	10,90
hsa-mir-16-1x	0,04	hsa-mir-132-3p	8,99
hsa-mir-17	45,05	hsa-mir-28-3p	8,50
hsa-mir-17x	0,02	hsa-mir-188-5p	8,16
hsa-mir-181a	0,02	hsa-mir-146b	7,55
hsa-mir-186	4,31	hsa-mir-28	7,42

hsa-mir-188-5p	8,16	hsa-mir-30e	6,63
hsa-mir-18a	0,93	hsa-mir-20b	5,94
hsa-mir-190b	2,22	hsa-mir-768-5p	5,75
hsa-mir-191	95,01	hsa-mir-25	5,59
hsa-mir-192	0,38	hsa-mir-34a	5,57
hsa-mir-193a-5p	0,13	hsa-mir-103	4,95
hsa-mir-193b	0,77	hsa-mir-374	4,82
hsa-mir-194	0,23	hsa-mir-625x	4,72
hsa-mir-195	2,97	hsa-mir-186	4,31
hsa-mir-197	3,48	hsa-mir-146a	3,74
hsa-mir-199a-3p	0,05	hsa-mir-197	3,48
hsa-mir-19a	3,30	hsa-mir-30e-3p	3,41
hsa-mir-19b	130,89	hsa-mir-19a	3,30
hsa-mir-19b-1x	0,15	hsa-mir-339-5p	3,16
hsa-mir-200b	0,18	hsa-mir-328	3,09
hsa-mir-200c	2,15	hsa-mir-195	2,97
hsa-mir-20a	71,89	hsa-mir-766	2,70
hsa-mir-20ax	0,06	hsa-mir-574-3p	2,60
hsa-mir-20b	5,94	hsa-mir-30d	2,53
hsa-mir-21	68,92	hsa-mir-660	2,35
hsa-mir-21x	0,21	hsa-let-7f	2,29
hsa-mir-212	0,13	hsa-mir-190b	2,22
hsa-mir-22x	0,07	hsa-mir-200c	2,15
hsa-mir-221	0,08	hsa-mir-532	2,12
hsa-mir-222	42,35	hsa-mir-532-3p	2,03
hsa-mir-223	257,22	hsa-mir-148a	1,88
hsa-mir-223x	1,06	hsa-mir-342-5p	1,85
hsa-mir-24	44,73	hsa-mir-520c-3p	1,75
hsa-mir-25	5,59	hsa-mir-610	1,63
hsa-mir-26a	75,12	hsa-mir-744	1,50
hsa-mir-26b	28,95	hsa-mir-423-5p	1,44
hsa-mir-26bx	0,03	hsa-mir-142-5p	1,24
hsa-mir-27a	0,68	hsa-mir-27ax	1,23
hsa-mir-27ax	1,23	hsa-mir-29b	1,21
hsa-mir-27b	0,10	hsa-mir-378	1,15
hsa-mir-28	7,42	hsa-mir-760	1,07
hsa-mir-28-3p	8,50	hsa-mir-223x	1,06
hsa-mir-296	0,46	hsa-mir-151-3p	0,94
hsa-mir-29a	69,64	hsa-mir-18a	0,93
hsa-mir-29ax	0,63	hsa-mir-590-5p	0,88
hsa-mir-29b	1,21	hsa-mir-526bx	0,86
hsa-mir-29b-1x	0,19	hsa-mir-138-1x	0,84
hsa-mir-29b-2x	0,12	hsa-mir-193b	0,77
hsa-mir-29c	11,13	hsa-mir-101	0,73
hsa-mir-301	0,14	hsa-mir-454	0,71
hsa-mir-30a-3p	0,19	hsa-mir-601	0,68
hsa-mir-30a-5p	11,23	hsa-mir-27a	0,68

hsa-mir-30b	57,54	hsa-mir-29ax	0,63
hsa-mir-30c	59,56	hsa-mir-339-3p	0,62
hsa-mir-30d	2,53	hsa-mir-324-3p	0,59
hsa-mir-30dx	0,32	hsa-mir-652	0,55
hsa-mir-30e	6,63	hsa-mir-92a-1x	0,47
hsa-mir-30e-3p	3,41	hsa-mir-296	0,46
hsa-mir-320	31,12	hsa-mir-513-3p	0,45
hsa-mir-324-3p	0,59	hsa-mir-769-5p	0,44
hsa-mir-324-5p	0,36	hsa-mir-516-3p	0,43
hsa-mir-328	3,09	hsa-mir-505x	0,40
hsa-mir-330	0,12	hsa-mir-192	0,38
hsa-mir-331	12,33	hsa-mir-886-3p	0,37
hsa-mir-335	0,25	hsa-mir-324-5p	0,36
hsa-mir-335x	0,08	hsa-mir-345	0,34
hsa-mir-339-3p	0,62	hsa-mir-15a	0,32
hsa-mir-339-5p	3,16	hsa-mir-30dx	0,32
hsa-mir-33ax	0,03	hsa-mir-34ax	0,29
hsa-mir-340	0,20	hsa-mir-128a	0,29
hsa-mir-340x	0,21	hsa-mir-486-3p	0,28
hsa-mir-342-3p	71,80	hsa-let-7c	0,27
hsa-mir-342-5p	1,85	hsa-mir-598	0,26
hsa-mir-345	0,34	hsa-mir-140-3p	0,25
hsa-mir-34a	5,57	hsa-mir-335	0,25
hsa-mir-34ax	0,29	hsa-mir-130b	0,24
hsa-mir-361	0,22	hsa-mir-671-3p	0,23
hsa-mir-362	0,07	hsa-mir-194	0,23
hsa-mir-362-3p	0,04	hsa-mir-632	0,22
hsa-mir-365	0,11	hsa-mir-361	0,22
hsa-mir-374	4,82	hsa-mir-21x	0,21
hsa-mir-378	1,15	hsa-mir-629	0,21
hsa-mir-422a	0,06	hsa-mir-340x	0,21
hsa-mir-423-5p	1,44	hsa-mir-340	0,20
hsa-mir-425x	0,06	hsa-mir-30a-3p	0,19
hsa-mir-454	0,71	hsa-mir-29b-1x	0,19
hsa-mir-483-5p	0,05	hsa-mir-625	0,19
hsa-mir-484	11,91	hsa-mir-200b	0,18
hsa-mir-486-3p	0,28	hsa-mir-155-3p	0,15
hsa-mir-489	0,07	hsa-mir-19b-1x	0,15
hsa-mir-494	0,10	hsa-mir-301	0,14
hsa-mir-500	0,10	hsa-mir-212	0,13
hsa-mir-502-3p	0,11	hsa-mir-193a-5p	0,13
hsa-mir-505x	0,40	hsa-mir-330	0,12
hsa-mir-509-3p	0,05	hsa-mir-95	0,12
hsa-mir-513-3p	0,45	hsa-mir-29b-2x	0,12
hsa-mir-516-3p	0,43	hsa-mir-502-3p	0,11
hsa-mir-520c-3p	1,75	hsa-mir-365	0,11
hsa-mir-520d-5p	0,02	hsa-mir-148b	0,11

hsa-mir-526bx	0,86	hsa-mir-642	0,10
hsa-mir-532	2,12	hsa-mir-500	0,10
hsa-mir-532-3p	2,03	hsa-mir-494	0,10
hsa-mir-574-3p	2,60	hsa-mir-27b	0,10
hsa-mir-576-3p	0,02	hsa-mir-146b-3p	0,08
hsa-mir-579	0,05	hsa-mir-93x	0,08
hsa-mir-590-5p	0,88	hsa-mir-875-5p	0,08
hsa-mir-597	0,04	U47	0,08
hsa-mir-598	0,26	hsa-mir-335x	0,08
hsa-mir-601	0,68	hsa-mir-221	0,08
hsa-mir-610	1,63	hsa-mir-638	0,07
hsa-mir-625	0,19	hsa-mir-22x	0,07
hsa-mir-625x	4,72	hsa-mir-489	0,07
hsa-mir-628-5p	0,04	hsa-mir-886-5p	0,07
hsa-mir-629	0,21	hsa-mir-362	0,07
hsa-mir-632	0,22	hsa-mir-942	0,06
hsa-mir-636	0,02	hsa-mir-425x	0,06
hsa-mir-638	0,07	hsa-mir-422a	0,06
hsa-mir-642	0,10	hsa-mir-152	0,06
hsa-mir-645	0,04	hsa-mir-20ax	0,06
hsa-mir-652	0,55	hsa-mir-126x	0,06
hsa-mir-660	2,35	hsa-mir-509-3p	0,05
hsa-mir-671-3p	0,23	hsa-mir-126	0,05
hsa-mir-744	1,50	hsa-mir-579	0,05
hsa-mir-760	1,07	hsa-mir-199a-3p	0,05
hsa-mir-766	2,70	hsa-mir-483-5p	0,05
hsa-mir-768-3p	355,14	hsa-mir-141	0,05
hsa-mir-768-5p	5,75	hsa-mir-597	0,04
hsa-mir-769-5p	0,44	hsa-mir-362-3p	0,04
hsa-mir-875-5p	0,08	hsa-mir-645	0,04
hsa-mir-886-3p	0,37	hsa-mir-16-1x	0,04
hsa-mir-886-5p	0,07	hsa-mir-628-5p	0,04
hsa-mir-9x	0,01	hsa-mir-15ax	0,03
hsa-mir-923	43,28	hsa-mir-26bx	0,03
hsa-mir-92a	75,08	hsa-mir-33ax	0,03
hsa-mir-92a-1x	0,47	hsa-mir-181a	0,02
hsa-mir-93x	0,08	hsa-mir-132-5p	0,02
hsa-mir-942	0,06	hsa-mir-520d-5p	0,02
hsa-mir-95	0,12	hsa-mir-17x	0,02
hsa-mir-99b	0,01	hsa-mir-125a-5p	0,02
RNU24	80,64	hsa-mir-576-3p	0,02
RNU43	35,59	hsa-mir-135a	0,02
RNU44	647,65	hsa-mir-636	0,02
RNU48	4417,19	hsa-mir-148bx	0,02
RNU6B	15,96	hsa-mir-9x	0,01
U47	0,08	hsa-mir-99b	0,01
U6	6885,86	hsa-mir-127	0,01
