# Supplementary material for: CLL Cells Respond to B-Cell Receptor Stimulation with a MicroRNA/mRNA Signature Associated with MYC Activation and Cell Cycle Progression
Source: PLoS One. 2013 Apr 1;8(4):e60275. doi: 10.1371/journal.pone.0060275 (PMC3613353; doi:10.1371/journal.pone.0060275)
Supplement: Table S6 — Rank-product analysis of detected miRNA, showing fold change FC in samples stimulated for 3 and 24 hours, ranked according to increasing percentage false positive. (PDF) [file pone.0060275.s013.pdf]

	3 hours			24 hours	
miRNA	pfp	FC	miRNA	pfp	FC
hsa-mir-212	0	17,708	hsa-mir-132-3p	0	12,46797
hsa-mir-132-3p	0	7,2312	hsa-mir-212	0	20,91016
hsa-mir-155-3p	0,0214	2,9114	hsa-mir-155-3p	0,0124	2,764777
hsa-mir-20ax	0,0255	2,4697	hsa-mir-27ax	0,061	2,249485
hsa-mir-132-5p	0,0293333	3,1096	hsa-mir-146a	0,172857	1,863078
hsa-mir-19b-1x	0,035	2,9036	hsa-mir-513-3p	0,210625	1,593694
hsa-mir-92a-1x	0,10875	2,511	hsa-mir-22x	0,22475	1,698288
hsa-mir-148a	0,3352	1,1824	hsa-mir-92a-1x	0,233	1,819702
hsa-mir-27ax	0,3757273	2,0994	hsa-mir-601	0,2388	1,583076
hsa-mir-17x	0,5401333	1,7245	hsa-mir-190b	0,255556	1,530512
hsa-mir-29b-1x	0,5491875	1,8077	hsa-mir-516-3p	0,261267	1,582274
hsa-mir-636	0,5577647	1,6385	hsa-mir-132-5p	0,271643	1,749023
hsa-mir-21x	0,5598571	1,9041	hsa-mir-19b-1x	0,284	1,707217
hsa-mir-126	0,5818462	1,0485	hsa-mir-188-5p	0,314563	1,255524
hsa-mir-221	0,60225	1,3216	hsa-mir-34ax	0,421647	1,516603
hsa-mir-33ax	0,6456111	1,4749	hsa-mir-671-3p	0,425105	1,568038
hsa-mir-127	0,9346842	1,1554	hsa-mir-148a	0,430667	1,204139
hsa-mir-766	0,9424167	0,8197	hsa-mir-21x	0,436091	1,449851
hsa-mir-330	0,9440279	0,855	hsa-mir-33ax	0,442143	1,354462
hsa-mir-331	0,9471703	0,8855	hsa-mir-18a	0,46105	1,404634
hsa-mir-484	0,9472955	0,8452	hsa-mir-489	0,486652	1,28718
hsa-mir-324-5p	0,9483842	0,923	hsa-mir-422a	0,546875	1,353562
hsa-mir-361	0,9493315	0,8201	hsa-mir-17x	0,57036	1,21315
hsa-mir-532-3p	0,9518057	0,887	hsa-mir-509-3p	0,648615	1,223682
hsa-mir-486-3p	0,951989	0,7549	hsa-mir-9x	0,708536	1,016985
hsa-mir-191	0,9521214	0,8554	hsa-mir-20ax	0,709889	1,037222
hsa-mir-625x	0,9533023	0,7529	hsa-mir-29b-1x	0,7177	1,230044
hsa-mir-769-5p	0,956092	0,8879	hsa-mir-135a	0,718793	1,131886
hsa-mir-339-3p	0,9576885	0,7997	hsa-mir-20b	0,793286	1,211465
hsa-let-7g	0,957807	0,9567	hsa-mir-17	0,813848	1,23746
hsa-mir-197	0,9604076	0,8005	hsa-mir-106a	0,814441	1,246792
RNU48	0,9604919	0,7785	hsa-mir-632	0,828432	1,166086
hsa-mir-768-3p	0,9621647	0,8644	hsa-mir-193b	0,831781	1,14467
hsa-mir-489	0,9676923	0,6906	hsa-mir-629	0,851389	1,204837
hsa-mir-222	0,9677917	0,9495	hsa-mir-610	0,85229	1,16358
hsa-mir-339-5p	0,9728323	0,7738	hsa-mir-486-3p	0,989332	0,486097
hsa-mir-128a	0,9769458	0,886	hsa-mir-25	0,994437	0,610173
RNU24	0,9789879	0,8806	hsa-mir-324-3p	0,995514	0,608823
hsa-mir-425x	0,983311	0,8135	hsa-mir-26a	0,99761	0,568853
hsa-mir-671-3p	0,9845645	0,7427	hsa-mir-769-5p	0,997968	0,483546
hsa-mir-652	0,9869571	0,7988	hsa-mir-345	0,999081	0,317731
hsa-mir-494	0,9897019	0,8274	hsa-mir-766	0,999883	0,650218
hsa-mir-744	0,9910062	0,8179	hsa-mir-26b	1,002687	0,57144
hsa-mir-24	0,994	0,9591	hsa-mir-15b	1,007831	0,601186

hsa-mir-875-5p	0,9969551	0,6356	hsa-mir-598	1,012638	0,562559
hsa-mir-28-3p	0,9983822	0,9086	hsa-let-7a	1,014057	0,56493
hsa-mir-342-3p	1,0000943	0,9204	hsa-let-7d	1,017971	0,645404
hsa-mir-502-3p	1,0024968	0,9685	hsa-let-7e	1,023753	0,63829
hsa-let-7e	1,0042595	0,9403	hsa-mir-197	1,029503	0,595364
hsa-mir-454	1,0080325	0,9951	hsa-mir-423-5p	1,030349	0,608936
hsa-mir-324-3p	1,0102876	0,9574	hsa-let-7g	1,036175	0,629758
hsa-mir-28	1,0135197	0,9648	hsa-mir-30b	1,040347	0,615677
hsa-mir-320	1,0187881	0,9138	hsa-mir-30dx	1,042474	1,114413
hsa-mir-378	1,02152	0,8735	hsa-mir-625	1,045544	0,617888
hsa-mir-93x	1,0282282	0,9835	hsa-mir-30c	1,049503	0,63463
hsa-mir-140-3p	1,0349054	0,9672	hsa-mir-28	1,050583	0,626626
hsa-mir-30d	1,0361497	1,0095	hsa-mir-103	1,052416	0,664028
hsa-mir-513-3p	1,0374384	0,4617	hsa-mir-652	1,055224	0,578305
hsa-mir-103	1,0436207	0,9941	hsa-mir-638	1,06122	0,54136
hsa-mir-15b	1,0502083	0,9817	hsa-mir-301	1,064847	0,622188
hsa-mir-92a	1,0504196	0,9798	hsa-mir-328	1,065716	0,582397
hsa-mir-342-5p	1,0535141	0,8469	hsa-mir-93x	1,071615	0,693178
hsa-mir-95	1,0572482	0,9153	hsa-mir-28-3p	1,07522	0,655729
hsa-mir-25	1,0634	1,0158	hsa-mir-26bx	1,078113	0,658282
hsa-mir-223	1,0672662	0,9715	hsa-mir-29b-2x	1,079823	0,567811
RNU44	1,072058	0,9192	hsa-mir-195	1,083277	0,633273
hsa-mir-17	1,0754963	1,0508	hsa-mir-30d	1,085987	0,705829
hsa-mir-505x	1,077375	0,9111	hsa-mir-186	1,08614	0,679737
hsa-mir-30a-5p	1,0781168	1,036	hsa-let-7f	1,088682	0,722207
hsa-let-7d	1,083291	0,9873	RNU48	1,093412	0,670175
hsa-mir-15a	1,0873864	0,9323	hsa-mir-768-5p	1,100072	0,584645
hsa-mir-148b	1,0886767	1,0091	hsa-mir-106b	1,106702	0,656901
hsa-mir-923	1,0894286	1,4345	hsa-mir-30a-5p	1,11192	0,715042
hsa-let-7a	1,0900534	0,9716	hsa-mir-500	1,118289	0,549484
hsa-mir-186	1,0921008	1,0459	hsa-mir-30e	1,123426	0,678067
hsa-mir-328	1,0975547	0,8514	hsa-mir-30e-3p	1,130551	0,728863
hsa-mir-29b-2x	1,0979923	0,8674	hsa-mir-342-3p	1,137856	0,70114
hsa-mir-532	1,098216	1,0196	hsa-mir-331	1,145655	0,746754
hsa-mir-576-3p	1,0992742	0,9486	hsa-mir-150	1,150417	0,697067
hsa-mir-598	1,1030157	1,0109	hsa-mir-128a	1,156993	0,700806
hsa-mir-22x	1,1043636	1,57	hsa-mir-361	1,160528	0,567033
hsa-mir-126x	1,10468	1,0815	hsa-mir-130b	1,162625	1,134357
hsa-mir-106a	1,106626	1,0772	hsa-mir-148b	1,167915	0,689324
hsa-mir-30c	1,1090164	1,0618	hsa-mir-27a	1,168615	1,006588
hsa-mir-579	1,1103254	0,9907	hsa-mir-340x	1,173199	0,713683
hsa-mir-106b	1,1149504	1,0283	hsa-mir-660	1,176058	0,646157
hsa-mir-340x	1,11785	1,0482	hsa-mir-140-3p	1,176236	0,684584
hsa-mir-335x	1,1193051	0,948	hsa-mir-127	1,17707	0,732713
hsa-mir-574-3p	1,1211513	0,9583	hsa-mir-142-3p	1,179933	0,593012
hsa-mir-362	1,1259145	1,0738	hsa-mir-454	1,180459	0,721006
hsa-mir-483-5p	1,1294	1,3036	hsa-mir-223	1,180576	0,624352

hsa-let-7f	1,1337931	1,0723	hsa-mir-15a	1,181321	0,679653
hsa-mir-301	1,1352609	1,042	hsa-mir-152	1,183576	0,933966
hsa-mir-135a	1,13725	1,196	hsa-let-7c	1,184196	0,621466
hsa-mir-422a	1,1387281	0,9034	hsa-mir-19b	1,184516	0,93277
hsa-mir-625	1,1401182	0,9862	hsa-mir-374	1,184646	0,676863
hsa-mir-20b	1,1432018	1,1279	hsa-mir-342-5p	1,187318	0,604775
hsa-mir-26bx	1,1441504	0,9417	hsa-mir-597	1,189176	1,009662
hsa-mir-146a	1,145	1,1824	hsa-mir-24	1,190119	0,977427
hsa-mir-192	1,1473796	1,0799	hsa-mir-148bx	1,190214	0,755589
hsa-mir-30e-3p	1,1483929	1,1049	hsa-mir-532-3p	1,19158	0,76816
hsa-mir-597	1,1546542	1,0892	hsa-mir-221	1,194317	0,668969
hsa-mir-200c	1,156018	0,9603	hsa-mir-194	1,194563	0,671858
hsa-mir-27b	1,1629528	0,8328	hsa-mir-142-5p	1,195154	0,647894
hsa-mir-423-5p	1,1677429	1,0136	RNU24	1,196923	0,92452
hsa-mir-150	1,1708077	1,7205	hsa-mir-502-3p	1,199368	0,79738
hsa-mir-190b	1,1763689	0,589	hsa-mir-365	1,201316	1,039124
hsa-mir-26a	1,1788846	1,0842	hsa-mir-16	1,201331	0,650323
hsa-mir-500	1,1818039	0,9467	hsa-mir-335x	1,202279	0,54669
hsa-mir-15ax	1,1832079	0,9662	U6	1,202619	1,088179
hsa-mir-142-3p	1,1892088	1,0598	hsa-mir-20a	1,203098	1,039815
hsa-mir-181a	1,1921481	0,9611	hsa-mir-126	1,204318	0,558182
hsa-mir-30a-3p	1,19375	1,1349	hsa-mir-324-5p	1,204602	0,742154
hsa-mir-30e	1,1979889	1,1275	hsa-mir-29a	1,207779	0,669921
hsa-mir-20a	1,2046869	1,1017	hsa-mir-16-1x	1,208696	0,768848
hsa-mir-130b	1,204764	1,0519	hsa-mir-923	1,2123	0,896693
hsa-mir-516-3p	1,2048261	0,6916	hsa-mir-483-5p	1,212435	0,883971
hsa-mir-30b	1,2058977	1,1384	RNU43	1,213871	0,918812
hsa-mir-223x	1,208051	1,0206	hsa-mir-768-3p	1,215438	0,70327
hsa-mir-146b-3p	1,2089787	0,9399	hsa-mir-15ax	1,215557	0,872792
hsa-mir-629	1,2121474	1,0883	hsa-let-7b	1,222333	0,654244
hsa-mir-296	1,2133021	0,8284	hsa-mir-628-5p	1,225667	0,970527
hsa-mir-660	1,2162069	1,1167	hsa-mir-875-5p	1,225844	1,066108
hsa-mir-16	1,217093	1,0523	hsa-mir-95	1,226176	0,674189
hsa-mir-148bx	1,2180206	1,1285	hsa-mir-34a	1,228255	0,855748
hsa-mir-628-5p	1,2195591	1,09	hsa-mir-378	1,229576	1,097297
hsa-mir-362-3p	1,2303214	1,3437	hsa-mir-520d-5p	1,230042	0,917124
hsa-mir-151-3p	1,2306	0,9245	hsa-mir-138-1x	1,232714	1,073849
hsa-mir-34a	1,2317976	0,9353	hsa-mir-339-3p	1,232743	0,999446
hsa-mir-138-1x	1,2354337	0,9676	hsa-mir-340	1,23341	0,676084
hsa-mir-195	1,2446951	1,0617	hsa-mir-526bx	1,233986	0,961345
U47	1,2497308	0,9783	hsa-mir-505x	1,235983	0,786705
hsa-mir-188-5p	1,2559041	0,7302	hsa-mir-942	1,238027	0,898691
hsa-mir-135ax	1,2569375	0,9844	hsa-mir-193a-5p	1,24086	1,016276
hsa-let-7c	1,2574074	0,9031	hsa-mir-494	1,241431	1,02849
hsa-mir-152	1,2585065	1,0975	hsa-mir-19a	1,242815	0,97003
hsa-mir-200b	1,2592639	1,0513	hsa-mir-320	1,243431	0,813227
RNU6B	1,2611266	1,0894	hsa-mir-92a	1,245104	1,103009

hsa-mir-194	1,2636933	1,1277	hsa-mir-126x	1,247245	0,59167
hsa-let-7b	1,2673382	0,9346	hsa-mir-141	1,247287	0,718345
hsa-mir-142-5p	1,273	1,1342	hsa-mir-146b	1,24831	0,862686
U6	1,2737162	1,0203	hsa-mir-199a-3p	1,248473	0,474679
hsa-mir-26b	1,2744737	1,1343	hsa-mir-532	1,255307	0,737118
hsa-mir-141	1,2752754	1,1746	hsa-mir-191	1,256265	0,758581
hsa-mir-34ax	1,2752817	1,0655	hsa-mir-579	1,258982	0,819928
hsa-mir-632	1,2769692	0,8691	U47	1,260215	0,706529
hsa-mir-193b	1,2776212	0,9532	hsa-mir-590-5p	1,26308	0,675336
hsa-mir-29c	1,2898	1,0932	hsa-mir-29c	1,267176	0,63206
hsa-mir-16-1x	1,2900328	1,0806	RNU6B	1,267885	1,064096
hsa-mir-768-5p	1,2924857	1,0355	hsa-mir-625x	1,268383	0,935981
hsa-mir-29a	1,2954063	1,1406	hsa-mir-223x	1,270906	0,675385
hsa-mir-590-5p	1,3033333	1,1267	hsa-mir-101	1,273784	0,627857
hsa-mir-638	1,3042333	0,9747	hsa-mir-744	1,274452	0,801434
hsa-mir-526bx	1,3077937	1,0998	hsa-mir-222	1,276233	0,822618
hsa-mir-146b	1,3160377	1,0646	hsa-mir-760	1,276638	0,832594
hsa-mir-125a-5p	1,3219153	0,9005	hsa-mir-29b	1,284343	0,642922
hsa-mir-601	1,3225968	0,6456	hsa-mir-335	1,287554	0,634917
hsa-mir-18a	1,3290192	1,2892	hsa-mir-27b	1,295043	0,995371
hsa-mir-99b	1,3367679	0,8794	hsa-mir-151-3p	1,29856	0,562164
hsa-mir-335	1,3385517	1,048	hsa-mir-576-3p	1,306755	0,840212
hsa-mir-645	1,3470784	1,0122	hsa-mir-362	1,309707	0,882644
hsa-mir-29b	1,3506333	1,2287	hsa-mir-192	1,314237	0,730469
hsa-mir-760	1,3566491	1,0367	hsa-mir-21	1,317935	0,713729
hsa-mir-9x	1,36976	1,0315	hsa-mir-645	1,320088	0,809214
hsa-mir-374	1,3830612	1,18	hsa-mir-520c-3p	1,32456	0,904319
hsa-mir-520d-5p	1,3842414	1,2933	hsa-mir-339-5p	1,327427	0,781536
hsa-mir-19a	1,3861951	1,2702	hsa-mir-29ax	1,329043	0,777857
hsa-mir-365	1,3914359	1,2218	hsa-mir-484	1,334033	0,848225
hsa-mir-642	1,4042826	0,9584	hsa-mir-574-3p	1,335579	0,743465
hsa-mir-340	1,4071136	1,2367	hsa-mir-296	1,339068	0,728576
hsa-mir-520c-3p	1,4083333	1,069	hsa-mir-425x	1,339609	0,840965
hsa-mir-610	1,4135	0,7802	hsa-mir-125a-5p	1,342228	0,755106
hsa-mir-193a-5p	1,4201489	1,0379	hsa-mir-200b	1,342531	0,75431
hsa-mir-19b	1,4246	1,2339	hsa-mir-362-3p	1,344085	0,763326
hsa-mir-345	1,4251935	1,4287	hsa-mir-181a	1,345463	0,628702
hsa-mir-509-3p	1,4252105	0,6839	hsa-mir-146b-3p	1,346663	0,755628
hsa-mir-101	1,4340233	1,1072	hsa-mir-135ax	1,347977	0,909521
hsa-mir-29ax	1,4413571	1,2176	hsa-mir-200c	1,349397	0,848916
hsa-mir-21	1,4434722	1,177	hsa-mir-99b	1,355792	0,775674
hsa-mir-27a	1,4446486	1,2382	hsa-mir-642	1,356176	0,656602
hsa-mir-942	1,4787429	1,3101	hsa-mir-636	1,356913	0,887892
hsa-mir-30dx	1,5084706	1,3937	hsa-mir-30a-3p	1,366947	0,942309
hsa-mir-199a-3p	1,5155625	0,9268	RNU44	1,369857	0,859945
RNU43	1,5279091	1,2505	hsa-mir-330	1,381783	0,841806
