# Supplementary material for: CLL Cells Respond to B-Cell Receptor Stimulation with a MicroRNA/mRNA Signature Associated with MYC Activation and Cell Cycle Progression
Source: PLoS One. 2013 Apr 1;8(4):e60275. doi: 10.1371/journal.pone.0060275 (PMC3613353; doi:10.1371/journal.pone.0060275)
Supplement: Table S7 — Canonical pathways associated with modulated genes after 3 hours or 24 hours of BCR stimulation. (PDF) [file pone.0060275.s014.pdf]

Table S7: Canonical pathways associated with modulated genes after 3 hours or 24 hours of BCR stimulation.

3 hours of stimulation		24 hours of stimulation	
B-cell receptor signaling	$1.34\ 10^{-6}$	Purine Metabolism	$3.94\ 10^{-8}$
PI3K Signaling in B lymphocytes	$1.75\ 10^{-6}$	TREM1 Signaling	$1.11\ 10^{-6}$
IL-4 Signaling	$5.37\ 10^{-6}$	Glycolysis/Gluconeogenesis	$6.91\ 10^{-6}$
Molecular Mechanisms of Cancer	$5.66\ 10^{-6}$	Pyrimidine Metabolism	$1.06\ 10^{-5}$
Interferon Signaling	$8.02\ 10^{-6}$	Protein Ubiquitination Pathway	$2.33\ 10^{-5}$

Canonical pathways most significantly enriched for genes modulated after BCR stimulation for 3 or 24 hours. Significance p value according to Ingenuity IPA® software
