# Supplementary material for: CLL Cells Respond to B-Cell Receptor Stimulation with a MicroRNA/mRNA Signature Associated with MYC Activation and Cell Cycle Progression
Source: PLoS One. 2013 Apr 1;8(4):e60275. doi: 10.1371/journal.pone.0060275 (PMC3613353; doi:10.1371/journal.pone.0060275)
Supplement: Table S8 — Correlation coefficient between gene and hsa-miR-132-3p or hsa-miR-212 miRNA expression. (PDF) [file pone.0060275.s015.pdf]

Correlation coefficient between gene and miRNA expression

hsa-miR-132-3p		hsa-miR-212	
VHL	-0,875	EDG1	-0,889
ANKRD28	-0,865	KIAA0831	-0,879
USPL1	-0,857	VHL	-0,875
MAPK8IP3	-0,847	LOC731985	-0,853
KIAA1545	-0,838	GDPD3	-0,850
CBL	-0,836	ANKRD28	-0,843
LAMA5	-0,836	CXCR4	-0,832
EDG1	-0,835	LOC648526	-0,829
ANKRD10	-0,833	CYP2U1	-0,825
NPHP3	-0,832	CHD6	-0,818
ZNF193	-0,827	SYF2	-0,818
CAPRIN2	-0,826	KTELC1	-0,811
EFHC1	-0,820	USPL1	-0,804
LHPP	-0,820	MAPK8IP3	-0,800
CYP2U1	-0,818	TSPAN3	-0,800
CXCR4	-0,815	LOC643382	-0,793
CDK5RAP2	-0,814	ARRDC2	-0,789
LOC727820	-0,814	ASTE1	-0,789
LCOR	-0,814	GUCY2C	-0,786
FLJ39639	-0,809	ADRB2	-0,786
TULP4	-0,808	SCARA5	-0,779
PRKRIP1	-0,805	ANKRD49	-0,775
LOC730256	-0,803	THAP2	-0,768
TMEM188	-0,800	ZNF193	-0,768
MGC24039	-0,800	LOC728481	-0,768
C17ORF56	-0,797	PGBD2	-0,764
KIAA0999	-0,797	LRIG1	-0,764
C1ORF63	-0,795	FAM129C	-0,764
TRABD	-0,794	FADS3	-0,761
ADRB2	-0,792	PRKRIP1	-0,761
KIAA1267	-0,792	CRLF3	-0,761
SLC44A2	-0,789	IFNGR2	-0,761
THSD1P	-0,788	ZNF260	-0,757
ZNF260	-0,786	LOC644935	-0,757
CECR1	-0,782	SAMD9	-0,757
PDCD6IP	-0,780	ARRB1	-0,754
ZNF154	-0,780	MSL3L1	-0,754
HNRNPH2	-0,779	B3GALT4	-0,754
RALGPS1	-0,779	LOC652071	-0,754
EP400	-0,776	NUF2	-0,750

KIAA0892	-0,774	ZBTB1	-0,750
P2RY8	-0,774	CPNE5	-0,750
GLTSCR2	-0,774	IGBP1	-0,750
ATM	-0,773	EP400	-0,750
ADAM28	-0,773	LOC727820	-0,746
ZNF688	-0,771	ZNF615	-0,746
YPEL3	-0,770	LILRB1	-0,743
ZCCHC11	-0,768	CPEB2	-0,739
UIMC1	-0,765	ABCA7	-0,739
C6ORF151	-0,765	DET1	-0,739
REM2	-0,764	KIAA0999	-0,739
ABCA7	-0,764	LYSMD2	-0,739
SULT1A4	-0,764	ELF2	-0,736
TRIM33	-0,764	TNRC18	-0,732
ZNF83	-0,764	UIMC1	-0,732
ANKRA2	-0,764	LOC728518	-0,729
KLHDC9	-0,762	CCDC106	-0,729
PAN2	-0,762	CRBN	-0,729
TMEM50B	-0,762	SYPL1	-0,725
GON4L	-0,761	PAN2	-0,725
MKLN1	-0,761	PNRC2	-0,725
KIAA0494	-0,759	NDC80	-0,721
TRAK1	-0,758	ADD3	-0,721
LOC202181	-0,756	ZNF277	-0,721
WBP1	-0,756	ZNF33A	-0,718
LOC653853	-0,755	CD46	-0,718
LOC730994	-0,755	LCOR	-0,718
AHR	-0,755	PCMTD1	-0,718
WDR23	-0,753	LOC651324	-0,714
UNC84A	-0,753	LOC641700	-0,714
LILRB1	-0,753	IFIT5	-0,714
N4BP1	-0,752	GPR18	-0,714
CREBBP	-0,752	ZNF816A	-0,714
CD37	-0,750	TMEM50B	-0,711
ADHFE1	-0,749	MGC12760	-0,707
MCM8	-0,749	HSD17B11	-0,707
C9ORF103	-0,749	LAIR1	-0,707
ADD3	-0,747	LOC646200	-0,707
FNBP4	-0,747	ZNF480	-0,704
RPL32P3	-0,746	CAPRIN2	-0,704
FLJ25778	-0,746	CDC42SE2	-0,704
WDR73	-0,743	HSBP1	-0,702
PAN3	-0,743	ZNF540	-0,700
ZFP90	-0,743	SMAD5	-0,700

C21ORF55	-0,741	ZNF33B	-0,700
IL18	-0,741	TMEM106B	-0,700
WDR22	-0,741	BCL11A	-0,700
RERE	-0,740	LOC653853	-0,696
ZBTB4	-0,740	FLJ25778	-0,696
CRYZL1	-0,738	LHPP	-0,696
SHCBP1	-0,738	LOC648251	-0,693
ADAM19	-0,737	LOC729446	-0,693
CRLF3	-0,737	MCM8	-0,693
RBL2	-0,737	P2RY8	-0,693
PTCD3	-0,735	LOC389671	-0,689
C5ORF25	-0,735	LOC644222	-0,689
DMTF1	-0,734	ZNF561	-0,689
XPC	-0,734	FLJ20021	-0,689
LOC642333	-0,734	ADAM19	-0,689
C20ORF195	-0,732	HMHA1	-0,689
ANKRD49	-0,732	ZBTB5	-0,689
AKR1D1	-0,732	EVI2B	-0,689
TSPYL4	-0,731	TTC7B	-0,686
ABR	-0,731	MFSD11	-0,686
AP1G2	-0,731	SEC62	-0,686
CDAN1	-0,731	ANKRA2	-0,686
CCDC97	-0,731	FKTN	-0,686
GGA2	-0,731	MBD4	-0,686
LOC644935	-0,729	RPS15A	-0,686
FKTN	-0,729	SPPL2B	-0,682
AIRE	-0,728	ZNF688	-0,682
C7ORF23	-0,728	C16ORF74	-0,682
NAG18	-0,726	EEF1D	-0,682
RHBDL1	-0,725	BIN2	-0,679
CRYGS	-0,725	CLSTN2	-0,679
FAM39DP	-0,725	TRIM23	-0,679
LOC146517	-0,725	AMN1	-0,679
CD47	-0,725	ATMIN	-0,679
KIAA0240	-0,725	CHST14	-0,679
ZNF486	-0,725	CDK5RAP2	-0,679
SETX	-0,723	RALA	-0,677
OMA1	-0,723	LOC388532	-0,676
TCF3	-0,722	ATG9B	-0,675
ZNF235	-0,720	GABRD	-0,675
CHST14	-0,719	FLJ39639	-0,675
LARP5	-0,718	TMEM188	-0,675
KIAA0247	-0,717	DAPP1	-0,675
SLC15A4	-0,717	FALZ	-0,671

KLHL20	-0,714	LOC651309	-0,671
DPH4	-0,714	CRYZL1	-0,671
SYF2	-0,714	FAM89B	-0,671
RPS11	-0,713	LOC731640	-0,671
ZNF23	-0,713	ING4	-0,668
ANGEL2	-0,713	DCLRE1C	-0,668
IFT88	-0,711	NPHP3	-0,668
ZBTB5	-0,711	TRIM33	-0,668
STK4	-0,711	KIAA0182	-0,668
PFKFB2	-0,710	IL18	-0,668
NBPF3	-0,710	HOM-TES-103	-0,664
SSBP4	-0,710	P2RY10	-0,664
NKTR	-0,710	WDR22	-0,661
IL7	-0,708	RNPEPL1	-0,661
CTDSP1	-0,708	NBPF3	-0,661
B3GALT4	-0,708	SCYL1BP1	-0,661
TSPAN3	-0,708	NBPF11	-0,661
SMC1A	-0,707	OMA1	-0,661
ZNF230	-0,707	PFDN1	-0,661
TTC21A	-0,707	KIAA0256	-0,661
ZNF211	-0,707	ANKRD30B	-0,661
HECTD1	-0,707	CDO1	-0,657
ANKRD30B	-0,707	ATP10B	-0,657
LIN54	-0,705	RAB37	-0,657
LOC729446	-0,705	DDX60	-0,657
ALOX5	-0,705	AIRE	-0,657
ACD	-0,705	TRIM68	-0,657
CIAO1	-0,705	UPF2	-0,657
VEZF1	-0,705	PTPRCAP	-0,657
POGK	-0,705	RPS14	-0,657
CPNE5	-0,704	LOC652840	-0,654
PGBD2	-0,704	TTC29	-0,654
PUM1	-0,704	KCNA3	-0,654
ZNF821	-0,702	GPR87	-0,654
AMN1	-0,702	C6ORF78	-0,654
ZNF319	-0,702	LOC202181	-0,654
PIAS1	-0,702	CEP290	-0,654
ZNF549	-0,702	ZNF613	-0,654
SOBP	-0,701	FAM26F	-0,654
DKFZP586I14	-0,701	C17ORF44	-0,654
RBM33	-0,701	LOC90586	-0,654
MBD4	-0,701	ANKRD10	-0,654
TNK2	-0,699	TNFRSF13C	-0,654
KIAA0831	-0,699	C3ORF52	-0,650

RBM17	-0,699	PFKFB2	-0,650
BCL11A	-0,698	KBTBD7	-0,650
ZFP3	-0,696	NR2C1	-0,650
AGGF1	-0,696	VEZF1	-0,650
AASDH	-0,696	RPS11	-0,649
SNX1	-0,695	C1QB	-0,646
SEC14L1	-0,695	SOBP	-0,646
NCF4	-0,695	TRIM67	-0,646
RYK	-0,695	FLJ37396	-0,646
WASPIP	-0,695	LIPT1	-0,646
NASP	-0,693	DOPEY2	-0,646
DDX17	-0,693	KIAA1545	-0,646
LOC643031	-0,693	PIAS1	-0,646
ZNF540	-0,692	LOC285941	-0,643
CCDC14	-0,692	TLR4	-0,643
GDPD3	-0,690	REM2	-0,643
PHF12	-0,690	FUT6	-0,643
PUM2	-0,690	ZNF187	-0,643
TOP2B	-0,690	SEC14L1	-0,643
RPL38	-0,689	ADAM28	-0,643
ZNF137	-0,689	ALOX5	-0,643
LOC727762	-0,689	STXBP3	-0,643
CROP	-0,689	LAMA5	-0,643
EVI2B	-0,689	MAP3K1	-0,642
WDR19	-0,689	LOC653907	-0,639
C9ORF45	-0,689	C1ORF157	-0,639
COX19	-0,688	RHBDL1	-0,639
TARSL2	-0,687	R3HDM2	-0,639
LOC728565	-0,687	BEST4	-0,639
MED23	-0,687	WDR73	-0,639
MGC29891	-0,687	RNF13	-0,639
MYST3	-0,687	DYRK2	-0,639
MITD1	-0,687	MRFAP1L1	-0,639
EHD1	-0,687	BCL3	-0,639
DHRS12	-0,686	JMJD1B	-0,639
BICD2	-0,686	LOC730256	-0,639
PDE4B	-0,686	RBL2	-0,639
NBPF11	-0,686	LOC729900	-0,636
SETDB1	-0,686	C6ORF195	-0,636
GALNAC4S-6S	-0,686	FSIP1	-0,636
MTX3	-0,684	ZNF512B	-0,636
SDCCAG3	-0,684	LOC54103	-0,636
ZBTB1	-0,683	FLJ20125	-0,636
FRAT1	-0,683	HYLS1	-0,636

LOC153561	-0,683	ATM	-0,636
DET1	-0,683	KIAA0892	-0,636
CCDC84	-0,683	LOC388621	-0,636
SGSM2	-0,683	LOC653658	-0,633
MRFAP1L1	-0,681	PROCA1	-0,632
STXBP3	-0,681	ZMAT1	-0,632
TNFRSF13C	-0,681	ARHGAP27	-0,632
PDE4C	-0,681	PRSS3	-0,632
C20ORF72	-0,681	ZNF254	-0,632
LOC729603	-0,680	MGC24039	-0,632
DICER1	-0,680	LOC730994	-0,632
RNF38	-0,680	CYSLTR1	-0,632
ZMAT3	-0,680	SYS1	-0,632
TOMM7	-0,680	SEPW1	-0,632
LOC388969	-0,678	VPREB3	-0,632
ST3GAL5	-0,678	PIM1	-0,632
SLC16A12	-0,678	ELK3	-0,629
LOC729900	-0,677	PGCP	-0,629
ZNF107	-0,677	ZMYM1	-0,629
ZNF512B	-0,677	CCDC66	-0,629
TAGLN	-0,677	ULK1	-0,629
SH3GLB2	-0,677	NUAK2	-0,629
BBS2	-0,675	FRAT2	-0,629
GUCY2C	-0,674	IQGAP1	-0,629
POLR3GL	-0,674	ZBED5	-0,629
LOC652071	-0,673	ABCB11	-0,625
LOC728518	-0,672	RRM2	-0,625
CYP2E1	-0,672	THSD1P	-0,625
ZNF350	-0,672	ZNF420	-0,625
ODF2L	-0,671	C10ORF73	-0,625
RNPEPL1	-0,671	C5ORF25	-0,625
TRIM23	-0,671	ZNF235	-0,625
ZNF45	-0,671	PAQR8	-0,625
ADA	-0,671	SEPHS1	-0,625
SPSB3	-0,671	TCTN1	-0,625
LOC643509	-0,671	RSBN1	-0,625
PDCD7	-0,671	LSM14A	-0,622
LOC644222	-0,669	RPL38	-0,622
MBTPS1	-0,669	LOC646447	-0,621
FAM76B	-0,669	PAX5	-0,621
LYSMD2	-0,669	LOC653829	-0,621
SDHALP1	-0,668	RRM2B	-0,621
ARRDC2	-0,668	FAM116A	-0,621
SULT1A1	-0,668	CLK4	-0,621

FAM129C	-0,668	ACD	-0,621
LOC399900	-0,667	TMEM59	-0,621
KIAA0355	-0,666	LOC648622	-0,621
CREB1	-0,666	LOC653806	-0,618
ZNF33A	-0,665	C20ORF195	-0,618
R3HDM2	-0,665	PDE7A	-0,618
PGM2L1	-0,665	CMIP	-0,618
6/sep	-0,665	FAM98C	-0,618
PDE7B	-0,665	SFRS2IP	-0,618
EVI5	-0,665	HERC6	-0,618
NUAK2	-0,665	GTF2IRD2B	-0,618
SYS1	-0,665	NAE1	-0,618
PNPLA7	-0,665	CREB1	-0,618
LOC401152	-0,665	ZNF486	-0,618
BEST4	-0,663	LOC645138	-0,618
MFSD11	-0,663	LOC650822	-0,614
C14ORF102	-0,663	LOC728032	-0,614
SIAH1	-0,663	LOC732425	-0,614
TCFL5	-0,663	LOC90925	-0,614
C1ORF66	-0,663	CDC25B	-0,614
LRDD	-0,662	AGGF1	-0,614
ZNF225	-0,662	IFT57	-0,614
ELF2	-0,662	PRICKLE1	-0,614
PYHIN1	-0,662	AES	-0,614
SAMD9	-0,662	C5ORF29	-0,614
SRRM2	-0,662	GVIN1	-0,614
GNPTG	-0,660	MTPN	-0,614
LOC642701	-0,660	TOMM7	-0,614
ZNF425	-0,660	LOC643809	-0,611
LOC54103	-0,660	BFSP2	-0,611
MLL5	-0,660	CNOT8	-0,611
LOC90586	-0,660	C3ORF19	-0,611
IGBP1	-0,660	SLC15A4	-0,611
ROCK2	-0,660	LOC644411	-0,607
SPOCK2	-0,660	OR11H12	-0,607
ALS2CR16	-0,659	ZNF425	-0,607
CLK2	-0,659	TLR1	-0,607
ST3GAL1	-0,659	TNFSF10	-0,607
ZNF329	-0,659	CECR1	-0,607
RGL2	-0,657	ACAA2	-0,607
C1ORF186	-0,657	EVI5	-0,607
CLK4	-0,657	HHEX	-0,607
HYPK	-0,657	RYK	-0,607
CTGLF3	-0,657	HBXIP	-0,607

ZNF395	-0,657	ZNF518B	-0,607
GAB3	-0,656	ZNF69	-0,607
IFFO	-0,656	USP24	-0,607
ZNF33B	-0,656	CD79A	-0,607
ABLIM1	-0,656	MITD1	-0,607
EIF3F	-0,655	RPL17	-0,607
LOC646144	-0,654	GARNL4	-0,604
C1ORF220	-0,654	CENPQ	-0,604
CHD6	-0,654	ZNF821	-0,604
SMA4	-0,654	TULP4	-0,604
NCOA1	-0,654	TRAK1	-0,604
SLC25A28	-0,654	C9ORF103	-0,604
DBF4B	-0,653	ZNF671	-0,604
TAF1L	-0,653	SNRPN	-0,604
TRIM38	-0,653	KIAA1267	-0,604
ZNF318	-0,653	CYBA	-0,604
CEP110	-0,653	C6ORF151	-0,601
C19ORF22	-0,652	RAB27B	-0,600
ARMC2	-0,651	ZBED2	-0,600
C11ORF61	-0,651	MPP3	-0,600
TGIF1	-0,651	RAB11FIP4	-0,600
LIPT1	-0,651	LOC644117	-0,600
ERMN	-0,650	FMNL3	-0,600
DACT1	-0,650	SULT1A4	-0,600
SKP2	-0,650	PDCD6IP	-0,600
C21ORF24	-0,650	LOC653489	-0,600
CBX7	-0,650	RERE	-0,600
LOC400721	-0,650	ZNF154	-0,600
ADAMTS6	-0,648	TP53RK	-0,600
OCIAD1	-0,648	RAB3IP	-0,600
CSAD	-0,647	ZNF549	-0,600
AMT	-0,647	ZMAT3	-0,600
SLC9A9	-0,647	MKLN1	-0,600
AK1	-0,647	TRIM22	-0,600
DCLRE1C	-0,647	LOC649557	-0,596
SNRPN	-0,647	CSNK1A1L	-0,596
QSOX2	-0,647	NGFRAP1	-0,596
SHISA5	-0,647	C1ORF120	-0,596
MORC3	-0,646	ANXA2P3	-0,596
PCMTD1	-0,645	C1ORF220	-0,596
ARHGAP27	-0,645	ZNF57	-0,596
SDHAP3	-0,645	TUBD1	-0,596
IL12A	-0,645	WHDC1	-0,596
NPIP	-0,645	ARHGAP12	-0,596

LOC654103	-0,645	C1ORF83	-0,596
SLC25A35	-0,644	ZNF350	-0,596
C4ORF41	-0,644	IFIT2	-0,596
ADD1	-0,644	MTIF3	-0,596
RBM5	-0,644	YPEL3	-0,596
BBS1	-0,642	KLF13	-0,596
ZNF302	-0,642	SELL	-0,596
POLM	-0,642	EPSTI1	-0,596
LRCH4	-0,642	C4ORF34	-0,596
TAF3	-0,641	OR3A2	-0,593
CMAH	-0,641	ST8SIA4	-0,593
PHACS	-0,641	1/mrt	-0,593
ZNF133	-0,641	FCGR2B	-0,593
GPX1	-0,641	RCSD1	-0,593
YPEL5	-0,641	LRRFIP1	-0,593
LOC643977	-0,639	IK	-0,593
BRD8	-0,639	SLC25A28	-0,593
NUB1	-0,639	YPEL5	-0,593
C5ORF39	-0,639	AQP12A	-0,589
KLF13	-0,639	SLFN12	-0,589
KIAA2026	-0,638	ZNRF2	-0,589
DDX60	-0,638	TTC21A	-0,589
NIP30	-0,638	ZNF250	-0,589
GOLGA8B	-0,638	CBL	-0,589
GTF2IRD2P	-0,636	NIP30	-0,589
NLRP1	-0,636	ZSCAN16	-0,589
MZF1	-0,636	C21ORF24	-0,589
PIM1	-0,636	CDKN1B	-0,589
KIAA0430	-0,636	EHD1	-0,589
LAPTM5	-0,636	TRABD	-0,589
FARSLB	-0,636	RPL15	-0,589
PRDM2	-0,635	RPS27A	-0,589
SLC6A16	-0,635	LOC643284	-0,589
MS4A1	-0,635	PRSS2	-0,586
YTHDC1	-0,635	ITGA9	-0,586
SNURF	-0,635	FADS6	-0,586
SETD2	-0,635	OR2B2	-0,586
LOC644033	-0,635	KLHDC9	-0,586
RNF166	-0,633	RAB12	-0,586
KLRA1	-0,633	MNDA	-0,586
CAPS	-0,633	CIAO1	-0,586
PLEKHA2	-0,633	TMEM77	-0,586
ADNP	-0,633	TM9SF2	-0,586
CD46	-0,633	HSD17B7	-0,586

ZNF337	-0,633	LAPTM5	-0,586
IFNGR2	-0,633	PA2G4P4	-0,582
LSM14A	-0,633	LOC647928	-0,582
ITIH5	-0,632	LOC650657	-0,582
ASTE1	-0,632	SH3BGR	-0,582
CCDC106	-0,632	ZNF335	-0,582
DMAP1	-0,632	MED11	-0,582
TTC31	-0,632	LOC647346	-0,582
VPREB3	-0,632	C4ORF41	-0,582
CRKL	-0,632	C5ORF5	-0,582
DAG1	-0,630	FGF11	-0,579
VPS8	-0,630	LOC652663	-0,579
F8A1	-0,630	LOC138652	-0,579
DPEP2	-0,630	LOC286467	-0,579
LOC400890	-0,630	ZNF436	-0,579
LYN	-0,630	ARHGAP11B	-0,579
FRAT2	-0,629	PROK2	-0,579
SFRS14	-0,629	LOC729837	-0,579
FOXJ3	-0,629	ZNF230	-0,579
FAM39E	-0,627	IL16	-0,579
BIN3	-0,627	EBAG9	-0,579
C11ORF35	-0,627	SELO	-0,579
CENPJ	-0,627	SLC44A2	-0,579
BTN2A1	-0,627	CD37	-0,579
ZNF217	-0,627	C14ORF85	-0,579
TGOLN2	-0,627	LOC653867	-0,575
TXNDC16	-0,626	LOC644090	-0,575
TBC1D23	-0,626	DEGS2	-0,575
AFG3L1	-0,626	LOC643977	-0,575
DOPEY1	-0,626	TLR6	-0,575
FAM98C	-0,626	PLEKHG1	-0,575
SYNJ2BP	-0,626	C14ORF102	-0,575
ZSCAN18	-0,626	LOC440704	-0,575
C9ORF38	-0,624	TXNIP	-0,575
S100PBP	-0,624	LOC651635	-0,571
GSDML	-0,624	LOC730809	-0,571
TOP1MT	-0,624	DZIP1L	-0,571
C14ORF24	-0,624	LOC441120	-0,571
GVIN1	-0,624	FOXD2	-0,571
MX2	-0,624	ZNF510	-0,571
LOC728481	-0,624	SMA4	-0,571
ARL2BP	-0,624	KIAA1683	-0,571
RAB26	-0,623	ABHD3	-0,571
GNRH1	-0,623	PYHIN1	-0,571

GUSBL1	-0,623	PIK3IP1	-0,571
C14ORF28	-0,623	GALNAC4S-6S	-0,571
SNRP70	-0,623	AKR1D1	-0,571
MYD88	-0,623	C20ORF111	-0,571
CDKN2AIPNL	-0,623	EIF3F	-0,571
THAP11	-0,623	LOC728956	-0,568
LOC653081	-0,621	FCGR3A	-0,568
ZNF187	-0,621	C9ORF109	-0,568
RNF216	-0,621	LOC652887	-0,568
UPF2	-0,621	PTCD3	-0,568
PNN	-0,621	MTERFD2	-0,568
ZNF514	-0,620	TBC1D1	-0,568
C20ORF107	-0,620	LENG1	-0,568
AHSA2	-0,620	FRAT1	-0,568
ZMYM4	-0,620	CNNM4	-0,568
C6ORF111	-0,620	DPP8	-0,568
AGBL5	-0,618	KIAA1407	-0,568
TGFBI	-0,618	RNF38	-0,568
GLOD4	-0,618	CDAN1	-0,568
EVL	-0,618	NUB1	-0,568
RAB12	-0,617	ITM2B	-0,568
KMO	-0,617	C6ORF192	-0,568
BCOR	-0,617	SSH2	-0,568
ABTB1	-0,617	ZNF107	-0,564
PGS1	-0,617	CRABP1	-0,564
AES	-0,617	FGF20	-0,564
ZBED5	-0,617	ALOX15	-0,564
NISCH	-0,617	PCDH9	-0,564
LRAP	-0,617	CNTN6	-0,564
PLA2G4B	-0,616	KLRA1	-0,564
HESX1	-0,615	FLJ20489	-0,564
LOC654335	-0,615	CCBE1	-0,564
MIA3	-0,615	ZNF175	-0,564
FLJ39653	-0,615	SDCCAG3	-0,564
NADK	-0,615	STAT5B	-0,564
RAB11FIP2	-0,615	PRDM8	-0,564
BRWD2	-0,615	AASDH	-0,564
KIAA0256	-0,615	ZNF545	-0,564
C11ORF47	-0,614	15/sep	-0,564
CCDC100	-0,614	LOC642277	-0,561
PCBD2	-0,614	PLXDC2	-0,561
TLR6	-0,614	C8ORF30A	-0,561
GNG7	-0,614	LOC644944	-0,561
BAGE5	-0,612	CASC5	-0,561

FMNL3	-0,612	LOC653197	-0,561
NBPF14	-0,612	DKFZP686E24	-0,561
ZNF451	-0,612	LOC644642	-0,561
CBX4	-0,612	XRCC1	-0,561
VAMP1	-0,612	ZNF32	-0,561
ATMIN	-0,612	ZNF791	-0,561
EPM2AIP1	-0,612	MED23	-0,561
LRWD1	-0,612	C1ORF63	-0,561
TMEM77	-0,612	ZCCHC11	-0,561
CHMP1B	-0,612	HSPC268	-0,561
LOC144983	-0,611	RGS19	-0,561
LZTS2	-0,611	SHCBP1	-0,561
BCL3	-0,611	SLC7A7	-0,561
WDR5B	-0,609	ELOVL5	-0,561
PGCP	-0,609	PUM2	-0,561
ASCC3L1	-0,609	DUSP19	-0,561
SEC62	-0,609	OR4K15	-0,557
SCARB2	-0,609	CDH1	-0,557
PCMTD2	-0,609	FLJ11235	-0,557
C5ORF41	-0,609	CSNK1G1	-0,557
ANAPC4	-0,609	KLHL20	-0,557
NMT2	-0,608	PURA	-0,557
TMEM80	-0,608	CNN2	-0,557
PDE7A	-0,608	TRMT12	-0,557
ULK1	-0,608	AHR	-0,557
LIG1	-0,608	GGA2	-0,557
KIAA0182	-0,608	LOC130773	-0,554
PRKCE	-0,608	LOC340113	-0,554
ZFYVE20	-0,608	LOC647802	-0,554
SSH2	-0,608	FSTL3	-0,554
RAB37	-0,606	TBC1D23	-0,554
FLT3LG	-0,606	CRK	-0,554
ARHGAP12	-0,606	C17ORF48	-0,554
SETD1B	-0,606	BBS4	-0,554
HERC6	-0,606	HBP1	-0,554
CD5	-0,606	RIPK1	-0,554
OVGP1	-0,606	RAD21	-0,554
PRICKLE1	-0,606	SLC16A12	-0,554
ZNF439	-0,606	SF3A3	-0,554
ST6GAL1	-0,606	PDCD7	-0,554
LPP	-0,606	KIAA0430	-0,554
TXLNB	-0,605	RPL13A	-0,554
TRPC6	-0,605	CFL2	-0,550
FXYD7	-0,605	LOC650938	-0,550

TBC1D1	-0,605	CLDN22	-0,550
NR2C1	-0,605	CRCT1	-0,550
FCGRT	-0,605	SLC44A5	-0,550
ZBTB24	-0,605	TAS2R39	-0,550
MGC16703	-0,605	LOC338750	-0,550
CAMK1D	-0,603	CNP	-0,550
COL9A3	-0,603	HNRNPH2	-0,550
CALCOCO1	-0,603	MGC16075	-0,550
BBS4	-0,603	ZNF239	-0,550
TPMT	-0,603	KIAA2026	-0,550
NAGPA	-0,603	C3ORF58	-0,550
TAF4	-0,603	AGTPBP1	-0,550
IK	-0,603	METTL7A	-0,550
TOR1AIP1	-0,603	TCF3	-0,550
C1ORF152	-0,602	PARP12	-0,550
ANKRD26	-0,602	PDSS2	-0,550
IKBKB	-0,602	C8ORF59	-0,550
PITPNM1	-0,602	RGL2	-0,547
MAN1C1	-0,600	LOC653081	-0,546
INADL	-0,600	ZNF280B	-0,546
WBSCR18	-0,600	RNF166	-0,546
HKR1	-0,600	LBR	-0,546
NEK8	-0,600	LOC153561	-0,546
CNP	-0,598	CTDSP1	-0,546
FANCF	-0,598	FCHO1	-0,546
TUBD1	-0,598	SETX	-0,546
C6ORF136	-0,598	PRUNE	-0,546
AKT1	-0,598	TDRD7	-0,546
RHBDD2	-0,598	CCDC97	-0,546
LOC440704	-0,598	CTCF	-0,546
PHF15	-0,598	KIAA0355	-0,546
FAM55C	-0,597	PAN3	-0,546
LOC441124	-0,597	CHMP1B	-0,546
CASD1	-0,597	C12ORF47	-0,546
KRIT1	-0,597	GLTSCR2	-0,546
LOC197135	-0,597	LOC222967	-0,543
FLJ11286	-0,597	ZNF385A	-0,543
CTCF	-0,597	LOC347364	-0,543
LOC731985	-0,596	MBD5	-0,543
KLHL24	-0,595	LOC644421	-0,543
ANKRD12	-0,595	MGC27165	-0,543
DEGS2	-0,594	ARL4C	-0,543
ZNF280B	-0,594	KBTBD3	-0,543
LOC153684	-0,594	WDR23	-0,543

ZNF517	-0,594	USP33	-0,543
MSL3L1	-0,594	PARD6A	-0,543
YY1AP1	-0,594	SETD1B	-0,543
LOC221442	-0,594	PHF20	-0,543
DUSP18	-0,594	ARID4B	-0,543
CDC25B	-0,594	ZNF266	-0,543
C8ORF37	-0,594	FAM45A	-0,543
RPL15	-0,593	SNX10	-0,543
SART1	-0,592	CTSS	-0,543
ZNF500	-0,592	PVRIG	-0,543
ZNF34	-0,592	ZFP90	-0,543
C20ORF191	-0,592	KIAA0240	-0,543
CNPY3	-0,592	PDLIM1	-0,543
MYO9B	-0,592	C20ORF72	-0,543
TMEM17	-0,592	TOP2B	-0,543
LOC729843	-0,592	EIF3EIP	-0,543
SUMO3	-0,592	FLJ12334	-0,539
EIF4A2	-0,591	LOC646552	-0,539
HAL	-0,591	ZNF596	-0,539
SULT1A2	-0,591	C17ORF67	-0,539
NDC80	-0,591	LOC653093	-0,539
STX16	-0,591	LOC646573	-0,539
HSD17B7	-0,591	RAB23	-0,539
RBM22	-0,591	LOC728519	-0,539
RNF32	-0,589	LOC648059	-0,539
CCDC146	-0,589	KMO	-0,539
FAM117A	-0,589	GSDML	-0,539
TTC32	-0,589	TIFA	-0,539
KIAA0323	-0,589	TGFBI	-0,539
HSPC268	-0,589	DMAP1	-0,539
PRIC285	-0,589	ZNF430	-0,539
DNMT1	-0,589	RAB31	-0,539
LOC645895	-0,589	SERTAD2	-0,536
ARGLU1	-0,589	RASSF3	-0,536
TMEM86B	-0,588	LOC390378	-0,536
SNHG11	-0,588	LOC647003	-0,536
XRCC1	-0,588	LOC643665	-0,536
SCYL1BP1	-0,588	B4GALT2	-0,536
SLFN11	-0,588	BAGE5	-0,536
IRS2	-0,588	NR1D2	-0,536
SYPL1	-0,588	FOXO4	-0,536
ZNF615	-0,588	NR3C2	-0,536
RABGAP1	-0,588	SLC25A20	-0,536
CXCR5	-0,588	BRD8	-0,536

EP300	-0,586	SKP2	-0,536
ITCH	-0,586	GOLGA7	-0,536
USP24	-0,586	SSTR2	-0,536
CCDC130	-0,586	PDE4B	-0,536
LOC644391	-0,585	RALGPS1	-0,536
LOC652726	-0,585	MYO9B	-0,536
ZNF224	-0,585	TMEM17	-0,536
LOC651309	-0,585	ZNF721	-0,536
PLEKHG1	-0,585	KIAA0494	-0,536
AKAP11	-0,585	LRAP	-0,536
RNASET2	-0,584	LOC654114	-0,532
LOC653080	-0,583	GPM6B	-0,532
C16ORF74	-0,583	LOC652076	-0,532
KIAA1407	-0,583	FXYD7	-0,532
AGXT2L2	-0,583	CLOCK	-0,532
LOC645522	-0,582	SLC39A10	-0,532
RUNDC2A	-0,582	WDFY2	-0,532
C20ORF12	-0,582	ZBTB34	-0,532
LRIG1	-0,582	LOC728565	-0,532
RUFY1	-0,582	ZBP1	-0,532
KIAA1128	-0,582	AFF3	-0,532
SLC44A4	-0,582	CEP135	-0,532
ZNF430	-0,582	SESN1	-0,532
PDCD4	-0,582	GCA	-0,532
TBL1X	-0,582	CSNK1G2	-0,532
DKFZP686E24	-0,580	ISG20	-0,532
TLR1	-0,580	OR52E5	-0,529
SNTB2	-0,580	SPIN2A	-0,529
SMAD5	-0,580	BHLHB8	-0,529
BRPF3	-0,580	CENPC1	-0,529
TXNDC13	-0,580	LOC642574	-0,529
CCNDBP1	-0,580	MDM1	-0,529
TRRAP	-0,580	FAM55C	-0,529
BFSP2	-0,579	HDAC5	-0,529
ZNF320	-0,579	F11R	-0,529
POMT2	-0,579	SELP	-0,529
FAM104B	-0,579	ZNF136	-0,529
GGA1	-0,579	ZBTB39	-0,529
CENTB2	-0,579	C17ORF39	-0,529
ZYG11B	-0,579	GON4L	-0,529
C14ORF85	-0,579	C7ORF36	-0,529
IRF9	-0,579	PPM1K	-0,529
HEY1	-0,577	BLCAP	-0,529
ZNF229	-0,577	KIAA1370	-0,529

GKAP1	-0,577	LOC146517	-0,529
OSBPL7	-0,577	LOC648852	-0,529
ZNF791	-0,577	DPEP2	-0,529
SMCHD1	-0,577	CDC40	-0,529
RUNDC1	-0,577	LOC642333	-0,529
C3ORF19	-0,577	RP9	-0,525
RAB2B	-0,577	ATP5O	-0,525
PCID2	-0,577	TXLNB	-0,525
LOC728519	-0,576	DMXL2	-0,525
C1ORF83	-0,576	OR4N5	-0,525
SIGLEC10	-0,576	SPESP1	-0,525
TBCC	-0,576	LOC642423	-0,525
LOC728499	-0,576	ZNF592	-0,525
RSBN1	-0,576	FLJ14213	-0,525
TMEM149	-0,576	CYP2E1	-0,525
NUF2	-0,574	EFHC1	-0,525
CLDN15	-0,574	CASD1	-0,525
PPP2R5B	-0,574	ZFP3	-0,525
LUC7L	-0,574	BRDG1	-0,525
CUTL1	-0,574	COX19	-0,525
LOC653778	-0,574	EID2B	-0,525
ZNF69	-0,574	PCMTD2	-0,525
CDC40	-0,574	CBX7	-0,525
PDLIM1	-0,574	VPS4B	-0,525
LOC441087	-0,574	KRTAP19-4	-0,521
FAM155A	-0,573	AMBRA1	-0,521
PPM1A	-0,573	LOC649030	-0,521
RAI16	-0,573	FLJ35934	-0,521
C10ORF73	-0,573	NXF3	-0,521
TAF1C	-0,573	PIK3R5	-0,521
SMG1	-0,573	IL7	-0,521
DHRS1	-0,573	TAGLN	-0,521
ZFP36L2	-0,573	RBBP9	-0,521
LOC730744	-0,573	SULT1A1	-0,521
HSBP1	-0,572	ZNF45	-0,521
C1ORF132	-0,571	C1ORF186	-0,521
FLJ10404	-0,571	RTP4	-0,521
SPG7	-0,571	WBP1	-0,521
CCDC101	-0,571	CNOT7	-0,521
CRAMP1L	-0,571	HIVEP1	-0,521
ZNF652	-0,571	ZFP36L2	-0,521
RCSD1	-0,571	CCNDBP1	-0,521
HVCN1	-0,571	SNORD16	-0,521
C17ORF62	-0,571	XPC	-0,521

DUSP19	-0,571	PPP1CC	-0,521
LEAP-2	-0,570	EIF4A2	-0,518
LOC554206	-0,570	ASAH3L	-0,518
RFFL	-0,570	DAGLA	-0,518
LOC649864	-0,570	C8ORF74	-0,518
SEPN1	-0,570	WFDC5	-0,518
PPAPDC2	-0,570	CDR2L	-0,518
APPL2	-0,570	KIAA1984	-0,518
EID2B	-0,570	C11ORF77	-0,518
HNRPDL	-0,570	LOC649456	-0,518
ASB12	-0,568	TARSL2	-0,518
MAPKBP1	-0,568	UBE3B	-0,518
BAIAP3	-0,568	MS4A1	-0,518
DEPDC5	-0,568	CEP192	-0,518
FLJ35220	-0,568	TM2D2	-0,518
GALC	-0,568	PSIP1	-0,518
CABIN1	-0,568	ATG5	-0,518
POFUT1	-0,568	SYNJ2BP	-0,518
SLC46A3	-0,568	CHRNA5	-0,518
ARID3B	-0,568	TBCC	-0,518
YPEL2	-0,568	KLHL22	-0,518
PTPRCAP	-0,568	TMEM134	-0,518
LOC728032	-0,567	DEDD2	-0,518
F11R	-0,567	ZBTB24	-0,518
RG9MTD3	-0,567	LOC441087	-0,518
KIAA0556	-0,567	RPS10	-0,517
LOC286208	-0,567	LOC730919	-0,514
WWC3	-0,567	ELOVL2	-0,514
C7ORF26	-0,567	CRSP2	-0,514
BAT2D1	-0,566	LOC644852	-0,514
LOC642995	-0,565	BNIPL	-0,514
MBD5	-0,565	RPL23AP13	-0,514
MAN2A2	-0,565	DACT1	-0,514
ZNF550	-0,565	CCDC102A	-0,514
C20ORF67	-0,565	SPG3A	-0,514
RAD21	-0,565	LOC339804	-0,514
AKAP8	-0,565	LYST	-0,514
TMED4	-0,565	DOPEY1	-0,514
CYFIP2	-0,565	NASP	-0,514
LOC51035	-0,565	ADAM8	-0,514
ALKBH5	-0,565	LOC26010	-0,514
NIPSNAP3B	-0,564	SIAH1	-0,514
BRWD3	-0,564	CD47	-0,514
LOC285908	-0,564	CDKN2AIPNL	-0,514

GOLGA1	-0,564	RPL22	-0,514
SIN3B	-0,564	RPS27	-0,514
PRKD2	-0,564	LOC650005	-0,511
LOC729559	-0,564	GPR174	-0,511
ZNF641	-0,564	LOC646134	-0,511
TTC3	-0,564	COL1A2	-0,511
IFIT2	-0,564	COX11P	-0,511
DEDD2	-0,564	CITED4	-0,511
ZNF512	-0,564	WDR86	-0,511
ZNF510	-0,562	GPA33	-0,511
LOC90379	-0,562	EPB41L3	-0,511
BMF	-0,562	ZNF443	-0,511
CCBE1	-0,562	C11ORF61	-0,511
LIN7B	-0,562	PBXIP1	-0,511
NHLRC3	-0,562	LOC651957	-0,511
RIPK1	-0,562	BICD2	-0,511
PPWD1	-0,562	CREBBP	-0,511
P2RY10	-0,562	ALPP	-0,511
TRIM22	-0,562	ICAM3	-0,511
LOC389671	-0,561	LOC646003	-0,507
OMG	-0,561	LOC642616	-0,507
C3ORF42	-0,561	LOC651695	-0,507
LOC732425	-0,561	KIF27	-0,507
ASB6	-0,561	ALX3	-0,507
MST1	-0,561	LOC388327	-0,507
KIAA0460	-0,561	LOC653878	-0,507
C3ORF63	-0,561	VNN1	-0,507
SLC9A8	-0,561	CCDC122	-0,507
GPR18	-0,561	TSPYL4	-0,507
PPP3CC	-0,561	LOC729196	-0,507
C18ORF8	-0,561	CNR1	-0,507
LOC653197	-0,559	PWWP2B	-0,507
LOC91664	-0,559	C17ORF56	-0,507
DMWD	-0,559	PDE7B	-0,507
ELAVL1	-0,559	USP47	-0,507
FLJ46309	-0,559	GPR132	-0,507
SIN3A	-0,559	PXMP3	-0,507
MAP3K1	-0,559	DPH4	-0,507
BSN	-0,558	FLJ46309	-0,507
ZNF321	-0,558	KIAA0247	-0,507
SBK1	-0,558	CWF19L2	-0,507
ATP2A3	-0,558	SP110	-0,507
ZNF559	-0,558	FAM104A	-0,507
HSPBAP1	-0,558	TEAD2	-0,507

ZNF700	-0,558	CCDC109B	-0,507
PHF3	-0,558	TINP1	-0,507
TP53I13	-0,558	RPS29	-0,507
RN7SL1	-0,558	HRH1	-0,504
LGI4	-0,556	LOC388080	-0,504
HOM-TES-103	-0,556	LOC731895	-0,504
GUCA1B	-0,556	FAM114A1	-0,504
HDAC5	-0,556	LOC645966	-0,504
PLGLB1	-0,556	SLITRK3	-0,504
ARL3	-0,556	DYNC2H1	-0,504
FOXP4	-0,556	DBNDD1	-0,504
ZNF18	-0,556	LRDD	-0,504
NPEPL1	-0,556	ZNF133	-0,504
WDR59	-0,556	NCR3	-0,504
JAK1	-0,556	TMEM30A	-0,504
UNC50	-0,556	C21ORF55	-0,504
FCHSD1	-0,555	ZNF134	-0,504
STK35	-0,555	HECA	-0,504
LOC653829	-0,555	PACS1	-0,504
LOC729985	-0,555	FAM39DP	-0,504
PPM1K	-0,555	YPEL2	-0,504
DYRK1A	-0,555	DEF8	-0,504
NCOA6	-0,555	SCARB2	-0,504
EEF1D	-0,555	U2AF1L2	-0,504
GPM6B	-0,553	MGC29891	-0,504
CCDC24	-0,553	IL10RA	-0,504
ZNF493	-0,553	C7ORF23	-0,504
ZNF346	-0,553	CD300LB	-0,500
DPP8	-0,553	LOC285307	-0,500
FLJ45244	-0,553	LOC401431	-0,500
CES2	-0,553	FLJ45256	-0,500
TNFSF10	-0,553	TIE1	-0,500
RBM16	-0,553	DMD	-0,500
KTELC1	-0,553	AP4M1	-0,500
MAGT1	-0,553	ZNF225	-0,500
CPEB2	-0,552	TAF1L	-0,500
C1ORF56	-0,552	IFT52	-0,500
APPBP2	-0,552	TNK2	-0,500
CP110	-0,552	SEMA4B	-0,500
RRAGA	-0,552	FLJ40142	-0,500
TUG1	-0,552	ZNF564	-0,500
ALS2CR8	-0,550	FAM65A	-0,500
MLL3	-0,550	SPINT2	-0,500
CHST12	-0,550	ZBTB4	-0,500

RFXDC2	-0,550	C6ORF32	-0,500
DVL3	-0,550	PITPNM1	-0,500
ZNF786	-0,550	TBC1D9	-0,500
PSCD1	-0,550	LMBRD1	-0,500
KLF2	-0,550		
LOC652541	-0,549		
ZNF248	-0,549		
DTWD2	-0,549		
CNOT6	-0,549		
MANBA	-0,549		
C3ORF34	-0,549		
SIX5	-0,547		
ARL17P1	-0,547		
PLEKHA1	-0,547		
CHRNA5	-0,547		
ZNF434	-0,547		
PIGC	-0,547		
AK3	-0,547		
IFIT5	-0,547		
LOC649095	-0,546		
ZNF57	-0,546		
DTX3	-0,546		
TACC3	-0,546		
LOC400304	-0,546		
ZNF266	-0,546		
LMF2	-0,546		
HDAC1	-0,546		
ZNF658	-0,544		
LOC644891	-0,544		
PRKAR2A	-0,544		
C6ORF64	-0,544		
CCDC16	-0,544		
ERCC5	-0,544		
PPP1R16B	-0,544		
SEMA3C	-0,543		
FLJ10120	-0,543		
LOC650883	-0,543		
CDO1	-0,543		
VSIG1	-0,543		
ZNF607	-0,543		
GNB5	-0,543		
LYG1	-0,543		
LOC92497	-0,543		
ZNF763	-0,543		

HERPUD2	-0,543
SENP6	-0,543
PLCH2	-0,543
C14ORF173	-0,543
STAT5B	-0,543
TMEM42	-0,543
DUSP28	-0,543
LRRFIP1	-0,543
YWHAZ	-0,543
FAIM3	-0,543
OR4K15	-0,541
LOC644450	-0,541
PLEC1	-0,541
LOC285359	-0,541
SMARCC2	-0,541
ZC3H3	-0,541
C19ORF31	-0,541
LOC728888	-0,541
ANKRD13A	-0,541
KIF27	-0,540
RAP2B	-0,540
LOC338799	-0,540
ANKRD36B	-0,540
SF3A1	-0,540
C7ORF41	-0,540
TRA2A	-0,540
MKRN1	-0,539
CFL2	-0,538
LOC440030	-0,538
IFT122	-0,538
GTF2IRD2	-0,538
TSGA10	-0,538
CDC42EP4	-0,538
FAM48A	-0,538
ZBTB39	-0,538
IL11RA	-0,538
SIDT1	-0,538
CLCN6	-0,538
GAS8	-0,537
RPLP2	-0,537
C10ORF46	-0,537
FLJ20309	-0,537
DBNDD1	-0,537
RNF26	-0,537

WDR37	-0,537
C2ORF64	-0,537
CTSS	-0,537
PIK3IP1	-0,537
SDHA	-0,537
ADAR	-0,537
SPEN	-0,537
GLTPD2	-0,535
LTBP1	-0,535
CBFA2T2	-0,535
LOC653086	-0,535
KIAA1147	-0,535
CDK5RAP3	-0,535
PEX11B	-0,535
FGF11	-0,534
SLC14A1	-0,534
WHDC1	-0,534
KIAA0226	-0,534
SH2B1	-0,534
POFUT2	-0,534
CORO1B	-0,534
DNAJB2	-0,534
CTSO	-0,534
LOC654123	-0,532
MGC12760	-0,532
CEND1	-0,532
LRRC56	-0,532
C3ORF17	-0,532
MGC52000	-0,532
LAX1	-0,532
ARIH2	-0,532
PGRMC2	-0,532
ZNF345	-0,531
ZNF335	-0,531
TRIM41	-0,531
RC3H2	-0,531
FAM113A	-0,531
FAM73A	-0,531
ARID4B	-0,531
SFRS17A	-0,531
ZDHHC8	-0,531
GYLTL1B	-0,529
NPHP4	-0,529
RCBTB2	-0,529

ZNF543	-0,529
RRM2B	-0,529
ZBTB34	-0,529
CRY2	-0,529
ABHD3	-0,529
LOC647389	-0,529
ZZEF1	-0,529
PLXDC2	-0,528
LOC653468	-0,528
FSTL3	-0,528
PKN1	-0,528
COL4A3	-0,528
RILPL1	-0,528
LOC400464	-0,528
FOXO3	-0,528
USP47	-0,528
FKBP14	-0,528
SMC3	-0,528
AQR	-0,528
C7ORF47	-0,528
KIAA0174	-0,527
DDX26B	-0,526
C1QTNF6	-0,526
RIC3	-0,526
SCAPER	-0,526
LOC729776	-0,526
C14ORF153	-0,526
RDH14	-0,526
USP49	-0,526
FAM104A	-0,526
TMEM66	-0,526
COX4I1	-0,526
LOC648251	-0,525
TTLL3	-0,525
UHRF2	-0,525
BLR1	-0,525
ZMYM6	-0,525
ZNF10	-0,525
PRDM8	-0,525
FAM65A	-0,525
GOLGA3	-0,525
TMEM131	-0,525
CCDC7	-0,523
LOC653867	-0,523

C20ORF106	-0,523
GARNL3	-0,523
LOC643011	-0,523
MTERFD2	-0,523
LOC158830	-0,523
HCN3	-0,523
DNAJC4	-0,523
LOC389517	-0,523
TSC1	-0,523
GRIPAP1	-0,523
SSTR2	-0,523
NAPB	-0,523
RBM4B	-0,523
BLZF1	-0,523
IL10RA	-0,523
COL4A4	-0,522
TBC1D3B	-0,522
C10ORF33	-0,522
LOC644642	-0,522
EBAG9	-0,522
FAM116A	-0,522
ZNF689	-0,522
TSPYL1	-0,522
C20ORF111	-0,522
C5ORF5	-0,520
SCARA5	-0,520
TMOD4	-0,520
LOC123688	-0,520
GOLGA8A	-0,520
DENND1C	-0,520
SF1	-0,520
ZMYM2	-0,520
RABL2B	-0,520
LOC653103	-0,520
LEMD2	-0,520
SP110	-0,520
CCR6	-0,520
LOC652076	-0,519
LOC728411	-0,519
COX11	-0,519
HPS4	-0,519
LOC641710	-0,519
C17ORF65	-0,519
CNNM4	-0,519

TRAPPC2	-0,519
PRPF4B	-0,519
ZNF250	-0,519
ZNF480	-0,519
RANBP10	-0,519
LOC339344	-0,519
ZBTB40	-0,519
FAM89B	-0,519
TJAP1	-0,519
KCNA3	-0,517
NR1D2	-0,517
DIS3	-0,517
FANCD2	-0,517
C6ORF170	-0,517
PDPR	-0,517
LBA1	-0,517
RAXL1	-0,517
SLU7	-0,517
FAM3A	-0,517
FN3KRP	-0,517
TEAD2	-0,517
GNB1	-0,517
ZNF621	-0,517
E2F5	-0,516
DOCK9	-0,516
LOC645550	-0,516
CDIPT	-0,516
CLDN14	-0,516
SMYD2	-0,516
LOC653066	-0,516
TM2D2	-0,516
ZNF767	-0,516
JMJD1B	-0,516
C5ORF29	-0,516
MON2	-0,516
USP34	-0,516
RPS27	-0,516
LOC652663	-0,514
RBED1	-0,514
CTPS2	-0,514
RCP9	-0,514
RBM38	-0,514
RGS19	-0,514
DDX24	-0,514

LOC646200	-0,513
FALZ	-0,513
RALGPS2	-0,513
SLC25A36	-0,513
TRAM2	-0,513
ZNF746	-0,513
ZC3H12A	-0,513
LOC644411	-0,511
PARD6B	-0,511
GH1	-0,511
GCC2	-0,511
1/mrt	-0,511
SPG11	-0,511
KRTAP21-1	-0,510
VPS13D	-0,510
NOXA1	-0,510
PEX1	-0,510
DGKQ	-0,510
ARID1A	-0,510
SF3B1	-0,510
MPHOSPH8	-0,510
TTC14	-0,510
KIAA1737	-0,510
DAPP1	-0,510
C14ORF135	-0,510
TPRG1L	-0,510
ARHGAP4	-0,510
ALPP	-0,510
KRTAP19-4	-0,508
LOC654000	-0,508
C11ORF77	-0,508
ARRB1	-0,508
RAD17	-0,508
ZNF160	-0,508
C10ORF26	-0,508
ASXL2	-0,508
FAM120B	-0,508
CNNM2	-0,507
C15ORF28	-0,507
CCDC88B	-0,507
FGR	-0,507
LOC730432	-0,507
FOXD2	-0,505
C6ORF26	-0,505

FLJ38717	-0,505
CSF2RA	-0,505
TMEM175	-0,505
STK36	-0,505
ZC3H7A	-0,505
XPNPEP3	-0,505
TXNIP	-0,505
F2R	-0,505
FNBP1	-0,505
HERC3	-0,504
IRS1	-0,504
CHI3L2	-0,504
RHBDL2	-0,504
HPS3	-0,504
PNPLA2	-0,504
ZNF76	-0,504
JARID1D	-0,504
CNOT8	-0,504
HDAC6	-0,504
EPS15	-0,504
LOC648852	-0,504
U2AF1L2	-0,504
PFAAP5	-0,504
HLA-DMA	-0,503
C21ORF74	-0,502
EMR4	-0,502
FLJ20489	-0,502
MYO5C	-0,502
AFF3	-0,502
DDX28	-0,502
UPF3A	-0,502
ZNF274	-0,502
CIRBP	-0,502
RPL36AL	-0,502
TMEM59	-0,501
FLJ11235	-0,501
LOC648189	-0,501
FLJ38969	-0,501
C1ORF107	-0,501
MBTD1	-0,501
CLSTN1	-0,501
ZBP1	-0,501
DOPEY2	-0,501
MOAP1	-0,501

HBP1	-0,501
TMEM134	-0,501
NME3	-0,501
SHROOM4	-0,501
GJC1	-0,501
