# Supplementary material for: CLL Cells Respond to B-Cell Receptor Stimulation with a MicroRNA/mRNA Signature Associated with MYC Activation and Cell Cycle Progression
Source: PLoS One. 2013 Apr 1;8(4):e60275. doi: 10.1371/journal.pone.0060275 (PMC3613353; doi:10.1371/journal.pone.0060275)
Supplement: Table S9 — Gene sets enriched for genes negatively correlating with hsa-miR-132-3p or hsa-miR-212 miRNA expression. (PDF) [file pone.0060275.s016.pdf]

Table S9: Gene sets enriched for genes negatively correlating with hsa-miR-132/212 expression

Reference	Subject	miR-132	miR-212
Mori et al., Cancer Res. 2008, Table S4	Down-regulated genes in the B lymphocyte developmental signature, expression profiling of lymphomas from Emu-myc transgenic mice: the Pre-BI stage.	0.0000	0.0000
Moserle et al., Cancer Res. 2008, Table S2	Top 50 genes up-regulated in ovarian cancer progenitor cells (also known as side population, SP, cells) in response to interferon alpha (IFNA).	0.0000	0.0000
O'Donnell et al., Mol Cell Biol. 2006, Table S7B	Genes up-regulated in P493-6 cells (B lymphocyte) by MYC and down-regulated by RNAi knockdown of TFRC .	0.0000	0.0012
Sotiriou et al., J Natl Cancer Inst. 2006, Table S1	Down-regulated genes whose expression correlated with histologic grade of invasive breast cancer tumors: comparison of grade 1 vs grade 3.	0.0000	0.0354
Yu et al., Ann N Y Acad Sci. 2005, Table 2	Genes down-regulated in B cell lymphoma tumors expressing an activated form of MYC.	0.0000	0.0019
Dazard et al., Oncogene 2003, Table S2	Genes down-regulated in NHEK cells (normal keratinocytes) by UV-B irradiation.	0.0010	0.0156
Klein et al., Blood 2003, Table S2	Genes down-regulated in AIDS-related primary effusion lymphoma (PEL) samples compared to other tumor subtypes and normal B lymphocytes.	0.0016	0.0014
Lee et al., Int Immunol. 2004, Table S1	Genes enriched at every T lymphocyte differentiation stage compared to the early passage fetal thymic stromal cultures (TSC).	0.0045	0.0189
Osman et al., Clin Cancer Res. 2006, Table S1	Genes down-regulated in blood samples from bladder cancer patients.	0.0045	0.0232
Winter et al., Cancer Res. 2007, Table S3	Genes down-regulated in head and neck tumor samples which clustered around known hypoxia genes.	0.0049	0.0056
Browne et al., J. Virol. 2001, Table S1	Genes down-regulated in primary fibroblast cell culture with at 6 h time point that were not down-regulated at the previous time point, 4 h.	0.0050	0.0184
Haddad et al., Blood 2004, Table S3	Genes up-regulated in hematopoietic progenitor cells (HPC) of B lymphocyte lineage CD34+CD45RA+CD10+.	0.0051	0.0024
Zhan et al., Blood 2006, Table S3	Top 50 down-regulated genes in cluster PR of multiple myeloma samples characterized by increased expression of proliferation and cell cycle genes.	0.0071	0.0147
Theilgaard et al., J Immunol. 2004, Table 3	Genes down-regulated in polymorphonuclear neutrophilic granulocytes (PMNs) attracted to skin wounds.	0.0072	0.0335
Tarte et al., Blood 2003, Table S4	Genes down-regulated in plasma cells compared with B lymphocytes.	0.0088	0.0232
Ginestier et al., Clin Cancer Res. 2006, Table S4	Genes up-regulated in non-metastatic breast cancer tumors having type 1 amplification in the 20q13 region; involves ZNF217 locus only.	0.0093	0.0031
Mori et al., Cancer Res. 2008, Table S4	Up-regulated genes in the B lymphocyte developmental signature, expression profiling of lymphomas from the Emu-myc transgenic mice: the mature B	0.0094	0.0359
Kim et al., Oncogene 2006, Table S3	Genes negatively correlated with amplifications of MYCN in the SCLC (small cell lung cancer) cell lines.	0.0095	0.0000
Rutella et al., Blood 2006, Table S1	Genes down-regulated in peripheral blood monocytes by HGF .	0.0223	0.0174
Zhan et al., Blood 2006, Table S3	Top 50 down-regulated genes in cluster LB of multiple myeloma samples belonging to the low bone disease group.	0.0244	0.0234
Zhou et al., J. Immunol. 2007, Table S2	Genes down-regulated in macrophages by P.gingivalis FimA pathogen.	0.0244	0.0170
Mori et al., Cancer Res. 2008, Table S4	Up-regulated genes in the B lymphocyte developmental signature expression profiling of lymphomas from Emu-myc transgenic mice: the immature B stage.	0.0245	0.0350
Pasqualucci et al., Nat Genet 2008, Table S2	Genes down-regulated in post-GC, BCL6 dependent B cell non-Hodgkin's lymphoma (B-NHL) vs MYC driven pre-GC lymphoma; GC = germinal center.	0.0281	0.0164
Gargalovic et al., Proc Natl Acad Sci USA 2006, Table S1	Genes which are down-regulated in primary aortic endothelium after exposure to the oxidized 1-palmitoyl-2-arachidonyl-sn-3-glycerophosphorylcholine	0.0365	0.0016
Krasnoselskaya et al., AIDS Res Hum Retroviruses 2002, Table 2	Up-regulated in GHOST CXCR4 cells (osteosarcoma) upon ectopic expression of ILF3.	0.0367	0.0498

Reference of publication, gene set description are listed, p values, for the miRNA indicated. Data generated through www.mirnabodymap.org
